# Pathway-based, reaction-specific annotation of disease variants for elucidation of molecular phenotypes

**DOI:** 10.1093/database/baae031

**Published:** 2024-05-07

**Authors:** Marija Orlic-Milacic, Karen Rothfels, Lisa Matthews, Adam Wright, Bijay Jassal, Veronica Shamovsky, Quang Trinh, Marc E Gillespie, Cristoffer Sevilla, Krishna Tiwari, Eliot Ragueneau, Chuqiao Gong, Ralf Stephan, Bruce May, Robin Haw, Joel Weiser, Deidre Beavers, Patrick Conley, Henning Hermjakob, Lincoln D Stein, Peter D’Eustachio, Guanming Wu

**Affiliations:** Adaptive Oncology, Ontario Institute for Cancer Research, 661 University Avenue Suite 510, Toronto, ON M5G 0A3, Canada; Adaptive Oncology, Ontario Institute for Cancer Research, 661 University Avenue Suite 510, Toronto, ON M5G 0A3, Canada; Department of Biochemistry and Molecular Pharmacology, New York University Grossman School of Medicine, 550 First Avenue, New York, NY 10016, USA; Adaptive Oncology, Ontario Institute for Cancer Research, 661 University Avenue Suite 510, Toronto, ON M5G 0A3, Canada; Adaptive Oncology, Ontario Institute for Cancer Research, 661 University Avenue Suite 510, Toronto, ON M5G 0A3, Canada; Department of Biochemistry and Molecular Pharmacology, New York University Grossman School of Medicine, 550 First Avenue, New York, NY 10016, USA; Adaptive Oncology, Ontario Institute for Cancer Research, 661 University Avenue Suite 510, Toronto, ON M5G 0A3, Canada; Adaptive Oncology, Ontario Institute for Cancer Research, 661 University Avenue Suite 510, Toronto, ON M5G 0A3, Canada; College of Pharmacy and Health Sciences, St. John’s University, 8000 Utopia Parkway, Queens, NY 11439, USA; European Molecular Biology Laboratory, European Bioinformatics Institute (EMBL-EBI), Wellcome Genome Campus, Hinxton, Cambridgeshire CB10 1SD, UK; Open Targets, Wellcome Genome Campus, Hinxton, Cambridgeshire CB10 1SD, UK; European Molecular Biology Laboratory, European Bioinformatics Institute (EMBL-EBI), Wellcome Genome Campus, Hinxton, Cambridgeshire CB10 1SD, UK; European Molecular Biology Laboratory, European Bioinformatics Institute (EMBL-EBI), Wellcome Genome Campus, Hinxton, Cambridgeshire CB10 1SD, UK; European Molecular Biology Laboratory, European Bioinformatics Institute (EMBL-EBI), Wellcome Genome Campus, Hinxton, Cambridgeshire CB10 1SD, UK; Adaptive Oncology, Ontario Institute for Cancer Research, 661 University Avenue Suite 510, Toronto, ON M5G 0A3, Canada; Institute for Globally Distributed Open Research and Education (IGDORE); Adaptive Oncology, Ontario Institute for Cancer Research, 661 University Avenue Suite 510, Toronto, ON M5G 0A3, Canada; Adaptive Oncology, Ontario Institute for Cancer Research, 661 University Avenue Suite 510, Toronto, ON M5G 0A3, Canada; Adaptive Oncology, Ontario Institute for Cancer Research, 661 University Avenue Suite 510, Toronto, ON M5G 0A3, Canada; Department of Medical Informatics and Clinical Epidemiology, Oregon Health and Science University, 3181 S.W. Sam Jackson Park Rd., Portland, OR 97239, USA; Department of Medical Informatics and Clinical Epidemiology, Oregon Health and Science University, 3181 S.W. Sam Jackson Park Rd., Portland, OR 97239, USA; European Molecular Biology Laboratory, European Bioinformatics Institute (EMBL-EBI), Wellcome Genome Campus, Hinxton, Cambridgeshire CB10 1SD, UK; Open Targets, Wellcome Genome Campus, Hinxton, Cambridgeshire CB10 1SD, UK; Adaptive Oncology, Ontario Institute for Cancer Research, 661 University Avenue Suite 510, Toronto, ON M5G 0A3, Canada; Department of Molecular Genetics, University of Toronto, 1 King’s College Circle, Room 4386, Toronto, ON M5S 1A8, Canada; Department of Biochemistry and Molecular Pharmacology, New York University Grossman School of Medicine, 550 First Avenue, New York, NY 10016, USA; Department of Medical Informatics and Clinical Epidemiology, Oregon Health and Science University, 3181 S.W. Sam Jackson Park Rd., Portland, OR 97239, USA

## Abstract

Germline and somatic mutations can give rise to proteins with altered activity, including both gain and loss-of-function. The effects of these variants can be captured in disease-specific reactions and pathways that highlight the resulting changes to normal biology. A disease reaction is defined as an aberrant reaction in which a variant protein participates. A disease pathway is defined as a pathway that contains a disease reaction. Annotation of disease variants as participants of disease reactions and disease pathways can provide a standardized overview of molecular phenotypes of pathogenic variants that is amenable to computational mining and mathematical modeling. Reactome (https://reactome.org/), an open source, manually curated, peer-reviewed database of human biological pathways, in addition to providing annotations for >11 000 unique human proteins in the context of ∼15 000 wild-type reactions within more than 2000 wild-type pathways, also provides annotations for >4000 disease variants of close to 400 genes as participants of ∼800 disease reactions in the context of ∼400 disease pathways. Functional annotation of disease variants proceeds from normal gene functions, described in wild-type reactions and pathways, through disease variants whose divergence from normal molecular behaviors has been experimentally verified, to extrapolation from molecular phenotypes of characterized variants to variants of unknown significance using criteria of the American College of Medical Genetics and Genomics and the Association for Molecular Pathology. Reactome’s data model enables mapping of disease variant datasets to specific disease reactions within disease pathways, providing a platform to infer pathway output impacts of numerous human disease variants and model organism orthologs, complementing computational predictions of variant pathogenicity.

**Database URL**: https://reactome.org/

## Introduction

Reactome is an open-source, manually curated, peer-reviewed knowledgebase of human biological pathways and an omics data analysis platform initially focused on normal processes (1–3). An expanded data model enables annotation of protein variants as participants in disease reactions and pathways (4, 5), supporting the intended use of Reactome as a disease mechanism elucidation tool (6). Wild-type biological reactions can only contain wild-type human protein participants, and wild-type biological pathways consist only of wild-type reactions. Any reaction involving a disease variant participant as either an input, a catalyst or a regulator is a disease reaction. Any pathway containing a disease reaction is a disease pathway. As a hierarchical pathway database, Reactome groups disease pathways based on the normal biological processes that they affect.

The American College of Medical Genetics and Genomics (ACMG)/Association for Molecular Pathology (AMP) Standards and Guidelines for the interpretation of sequence variants (7) provide the evidence framework to classify variants as benign or pathogenic based on (i) population, (ii) computational and predictive, (iii) functional, (iv) segregation, (v) de novo, (vi) allelic, (vii) another database-derived and (viii) other data. Criteria for assessing the evidence strength in these eight categories can be supporting (BP1–7) or strong (BS1–4) for benign variants and supporting (PP1–5), moderate (PM1–6), strong (PS1–4) or very strong (PVS1) for pathogenic variants. Adopting ACMG/AMP is the first step toward alignment and exchange of Reactome disease variant annotations with ACMG/AMP-compliant variant databases such as ClinGen and ClinVar (8).

Rather than comprehensively cataloging disease variants, Reactome describes the impact of representative protein variants on pathway activity through disease pathway-based, disease reaction-specific functional annotations. Reactome disease variants, whenever possible, cross-reference external open-source resources that provide DNA-level annotations and relevant clinical information: Online Mendelian Inheritance in Man (OMIM) (9), ClinGen Allele Registry (10), ClinVar (11) and disease-specific databases, such as RettBASE (12) for Mendelian disorders; Catalogue of Somatic Mutations in Cancer (COSMIC) (13), ClinGen Allele Registry (10) or ClinVar (11), and Leiden Open Variation Database (14) for cancer variants. Reactome adheres to the Human Genome Variation Society (HGVS) protein variant nomenclature (15) when available, with the exception of using (i) one-letter amino acid abbreviations and (ii) common protein isoform and cleaved protein fragment names.

Recent years saw a great progress of computational tools developed to assess the functional impact of variants, such as Sorting Intolerant From Tolerant (SIFT) (16), PolyPhen-2 (17), Mutation Assessor (18) and, very recently, AlphaMissense (19). However, these tools may produce false-negative and false-positive predictions. For example, pathogenic hotspot variants PIK3CA H1047L and PIK3CA H1047R (20, 21) are assessed as ‘neutral’, ‘tolerated’, ‘benign’ and ‘ambiguous’ by Mutation Assessor, SIFT, PolyPhen-2 and AlphaMissense, respectively. Furthermore, these tools cannot provide information on the functional contexts (e.g. pathways) impacted by the variants, and they mainly focus on missense variants, which represent only a fraction of disease variants. Therefore, manual curation of protein variants is still highly needed.

Here, we present a protocol we have developed to annotate variants in Reactome pathways and a manyfold expanded Reactome disease variant dataset that includes novel classes of protein variants such as fusion proteins. We also describe how Reactome’s pathway-based, reaction-specific disease variant annotations can be used to identify and fill in gaps in computational predictions, aiding in the interpretation and modeling of clinically relevant variants (22).

## Materials and methods

### Determining the scope of Reactome disease variant curation

**Figure 1.**
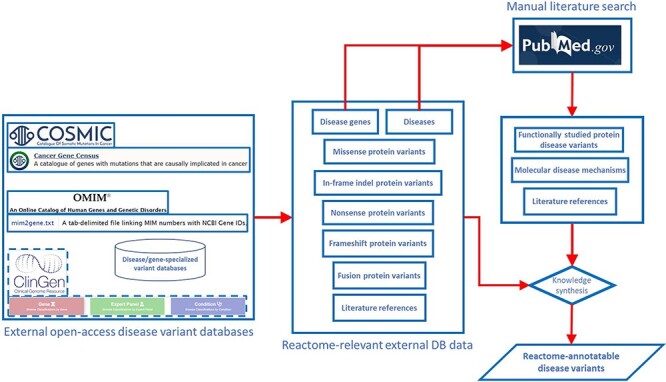
Pipeline for determining the scope of Reactome disease variant curation. ClinGen is shown with dashed borders as it is still to be routinely incorporated in the pipeline.

Annotation of disease variants in Reactome builds on annotation of wild-type biochemical functions of disease genes, meaning that in order to annotate a disease variant in Reactome, it is necessary for the wild-type function of the corresponding gene to be annotated in the context of a wild-type pathway. Disease variants of the gene of interest are retrieved from disease variant databases and from published literature (Figure 1). In the case of cancer driver genes, COSMIC database (13) is searched for missense, nonsense, in-frame insertion/deletion (indel) and frameshift mutations that can be traced back to published literature via COSMIC-provided PubMed (23) links. In the case of Mendelian disorders, OMIM (9) and ClinGen (24) are consulted to identify, if available, the most prominent disease variants in the target gene associated with the disorder or the initial relevant literature references from which such disease variants can be obtained. PubMed is also queried for any additional published, open-source, specialized variant databases dedicated to the Mendelian disorder in question, and if such databases are available, relevant disease variants are obtained from them. Once a draft list of disease variants for a gene of interest is established, PubMed (23) is manually searched for studies that provide functional descriptions of disease variants from the draft list, starting with the most frequent variants, and the search results are used to prune and append the list. Any functionally studied protein variants of interest identified in original research articles are included in the draft list of disease variants even if they have not been retrieved from the relevant databases. If the literature search shows that disease variants that lead to formation of fusion proteins are prominent in the disease etiology, then fusion variants are also included in the draft list, besides missense, nonsense, in-frame indels and frameshift variants.

### Aligning Reactome annotations with ACMG/AMP Standards and Guidelines

The ACMG/AMP Standards and Guidelines (7) framework for the interpretation of sequence variants based on evidence strength, pathogenicity and evidence type was evaluated for its ability to describe pathogenicity with Reactome-suitable evidence type and quality level (Table 1).

**Table 1.** ACMG/AMP evidence framework utilized by Reactome

Criterion	Evidence strength	Pathogenicity	Evidence type	Evidence definition	Suitability as direct Reactome evidence
BS1	Strong	Benign	Population data	Variant represents minor allele whose frequency is too high for disorder	−
BS2	Strong	Benign	Population data	Variant incidence in controls is inconsistent with disease penetrance	−
BS3	Strong	Benign	Functional data	Well-established functional studies show no deleterious effect for variant	−
BS4	Strong	Benign	Segregation data	Variant does not segregate with disease	−
BP1	Supporting	Benign	Computational and predictive data	Missense variant is found in gene in which only truncating variants cause disease	−
BP2	Supporting	Benign	Allelic data	Variant is observed in trans with dominant variant or in cis with pathogenic variant	−
BP3	Supporting	Benign	Computational and predictive evidence data	Variant is an in-frame indel in repeat without known function	−
BP4	Supporting	Benign	Computational and predictive evidence data	Multiple lines of computational evidence suggest that variant has no impact on gene or gene product	−
BP5	Supporting	Benign	Other data	Variant is found in disease case with an alternate cause	−
BP6	Supporting	Benign	Other database	A reputable source without shared data defines variant as benign	−
BP7	Supporting	Benign	Computational and predictive evidence data	Variant is silent variant with non-predicted splice impact	−
PP1	Supporting	Pathogenic	Segregation data	Variant co-segregates with disease in multiple affected family members	−
PP2	Supporting	Pathogenic	Functional data	Variant represents missense variant in gene in which there is low rate of benign missense variants and in which pathogenic missense variants are common	−
PP3	Supporting	Pathogenic	Computational and predictive data	Multiple lines of computational evidence support a deleterious effect of variant on gene/gene product	−
PP4	Supporting	Pathogenic	Other data	Variant is detected in a patient whose phenotype or family history are highly specific for disease gene	−
PP5	Supporting	Pathogenic	Other database	A reputable source identifies variant as pathogenic	−
PM1	Moderate	Pathogenic	Functional data	Variant represents mutational hotspot or is within functional domain without benign variation	✓
PM2	Moderate	Pathogenic	Population data	Variant is absent in population databases	−
PM3	Moderate	Pathogenic	Allelic data	For recessive disorders variant is detected in trans with known pathogenic variant	−
PM4	Moderate	Pathogenic	Computational and predictive data	Variant represents protein length changing variant	✓
PM5	Moderate	Pathogenic	Computational and predictive data	Variant represents novel missense change at amino acid residue previously reported to be affected by pathogenic missense change	✓✓
PM6	Moderate	Pathogenic	De novo data	De novo variant is detected (without paternity or maternity confirmed)	−
PS1	Strong	Pathogenic	Computational and predictive data	Variant leads to same amino acid change as an established pathogenic variant	✓✓✓
PS2	Strong	Pathogenic	De novo data	De novo variant is detected (with paternity and maternity confirmed)	−
PS3	Strong	Pathogenic	Functional data	Well-established functional studies show variant’s deleterious effect	✓✓✓
PS4	Strong	Pathogenic	Population data	Prevalence of variant in affected patients is statistically significantly increased over controls	−
PVS1	Very strong	Pathogenic	Computational and predictive data	Variant is predicted to be null variant in gene where LOF is known disease mechanism	✓✓

A minus (−) indicates that a criterion cannot be used as direct Reactome evidence. A checkmark (✓) indicates that a criterion can be used as direct evidence, with the number of checkmarks indicating the criterion’s strength from the perspective of Reactome’s data model. This table represents an edited version of a figure published in Genetics in Medicine, Vol. 17, by Richards et al., “Standards and guidelines for the interpretation of sequence variants: a joint consensus recommendation of the American College of Medical Genetics and Genomics and the Association for Molecular Pathology”, Page 415, Copyright Elsevier (2015) (7), reproduced and edited with authors’ and publisher’s permission.

### Reactome data model and curator tool

**Figure 2.**
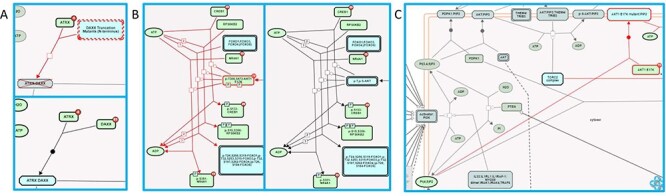
Disease variant-associated disease reactions in Reactome pathway diagrams. (A) Automated overlay of a LOF reaction ‘Defective DAXX does not bind ATRX’ (upper panel) onto normal reaction ‘ATRX binds DAXX’ (bottom panel) (28). (B) Automated overlay of GOF reactions showing phosphorylation of nuclear proteins by oncogenic AKT1 E17K variant (left panel) onto normal reactions in the subpathway ‘AKT phosphorylates targets in the nucleus’ (right panel) (29). (C) Manually added GOF reaction ‘AKT1 E17K mutant binds PIP2’, with no normal reaction counterpart (29).

As previously described (4, 5), Reactome annotates disease variants in the context of disease reactions tagged with Disease Ontology (DO) (25) disease terms. Two main types of disease reactions involving disease variants can be distinguished: (i) failed reactions, in which somatic or germline mutations lead to non-functional, i.e. loss-of-function (LOF) variant gene products, so reactions that normally depend on the wild-type gene product do not take place; these reaction-like events are represented as having inputs but no outputs; (ii) reactions where somatic or germline mutations lead to variant gene products with novel functions, i.e. gain-of-function (GOF) or abnormally enhanced wild-type-like functions, producing qualitatively different outcomes. In addition to the characteristic lack of outputs in failed reactions, the two types of disease variant-associated reactions can be distinguished by their ‘entity functional status’, an attribute that combines molecular phenotype terms (e.g. gain_of_function and loss_of_function) and sequence variant terms (e.g. missense_variant, frameshift_variant and stop_gained) from the Sequence Ontology (26) to describe molecular pathophysiological phenomena of disease variants at the reaction level. Association of the entity functional status with disease reactions rather than variants circumvents the problem arising when a variant differentially affects different functional aspects of a protein (27), enabling the Reactome data model to accurately define the behavior of disease variants in different contexts/reactions. Both types of disease variant-related disease reactions can be tagged with a ‘normal reaction’ attribute. This attribute allows the automated overlay of disease reactions onto corresponding wild-type reactions in Reactome pathway diagrams, allowing visualization of the perturbation of the wild-type biological process in the presence of disease variants. An automated overlay of cancer-derived LOF variants of Death domain-associated protein 6 (DAXX) in disease reaction ‘Defective DAXX does not bind ATRX’, belonging to the disease pathway ‘Defective Inhibition of DNA Recombination at Telomere Due to DAXX Mutations’, onto normal reaction ‘ATRX binds DAXX’, belonging to the wild-type pathway ‘Telomere Maintenance’, is shown in Figure 2A (28). An automated overlay of cancer-derived GOF variant of AKT1 in disease reactions ‘AKT1 E17K mutant phosphorylates forkhead box transcription factors’, ‘AKT1 E17K mutant phosphorylates CREB1’, ‘AKT1 E17K mutant phosphorylates RSK’ and ‘AKT1 E17K mutant phosphorylates NR4A1 (NUR77)’, belonging to the disease pathway ‘Constitutive Signaling by AKT1 E17K in Cancer’, onto normal reactions ‘AKT phosphorylates FOXO transcription factors’, ‘AKT phosphorylates CREB1’, ‘AKT can phosphorylate RSK’ and ‘AKT can phosphorylate NR4A1 (NUR77)’, respectively, belonging to the wild-type pathway ‘PIP3 activates AKT signaling’, is shown in Figure 2B (29). For GOF disease reactions in which disease variants participate in molecular interactions that do not occur between corresponding wild-type proteins, the normal reaction attribute is not applied, and these disease reactions are manually added to the pathway diagram. A manually drawn disease reaction involving a cancer-derived AKT1 variant, ‘AKT1 E17K mutant binds PIP2’, belonging to the disease pathway ‘Constitutive Signaling by AKT1 E17K in Cancer’, is shown in Figure 2C (29). Functionally analogous variants, whose biological function is perturbed in a qualitatively indistinguishable manner, are grouped into disease variant sets. Detailed instructions for annotation of disease variants can be found in the Supplementary Methods.

### Accessing Reactome disease variant data

Reactome disease variant data are stored in the central repository. Only reviewed data are available on Reactome webpages and accessible (https://reactome.org/dev/graph-database#Resources) through a Neo4j Graph Database (30).

Reactome disease variants can be accessed on the Reactome website (www.reactome.org) as previously published (31) (the section ‘EXPLORING REACTOME ANNOTATIONS OF DISEASE AND DRUGS’, pages 15–23 of Rothfels *et al*. 2023, subsections 8 and 9, and related figures 17 and 18, in particular, provide disease variant-relevant walk-through examples) and described in the User Guide (https://reactome.org/userguide) (the ‘diseases’ button on the main page of the User Guide leads to a detailed diseases page https://reactome.org/userguide/diseases with walk-through disease variant examples). Detailed descriptions of annotated disease processes and variants are also available in Reactome’s electronic textbook (https://reactome.org/download/current/TheReactomeBook.pdf.tgz).

### Analysis of disease genes and disease terms in Reactome

Unique Human Genome Organisation - Gene Nomenclature Committee (32) genes with Reactome-annotated disease variants were analyzed for pathway enrichment (Reactome web tool ‘Analyse gene list function’; Reactome Release 84, March 2023) and overlap with external disease gene databases (BioVenn web tool (33); InteractiVenn web tool (34); COSMIC Cancer Gene Census, COSMIC edition v97, November 2022; ClinGen genes: 5 April 2023 download; OMIM genes: Online Mendelian Inheritance in Man, OMIM®. McKusick-Nathans Institute of Genetic Medicine, Johns Hopkins University (Baltimore, MD), (5 April 2023). URL: https://omim.org/). Disease variant-associated DO terms (DO March 2023 release) were analyzed for prevalence and their relationship to the DO tree (constructed using Cytoscape 3.9.1 (35)) was analyzed using PathLinker (36).

## Results

### Disease genes and variants in Reactome

Reactome (version 84 March 2023) includes disease variants for 372 genes (disease genes). Each protein disease variant is annotated as an EntityWithAccessionedSequence (EWAS) instance, characterized by UniProt-derived referenceEntity (37), start and end coordinates, cellular compartment and a hasModifiedResidue attribute indicating post-translational modifications and mutated residues. One disease variant can have multiple EWASes. Reactome’s disease variant dataset is summarized in Table 2 and provided in Supplementary Table S1.

**Table 2.** Disease variant types in Reactome

Variant type	Variant EWASs	Unique variants
Missense	1955	1624
Missense in translation initiation codon	2	2
Frameshift	1869	1858
Nonsense	1096	1087
Nonsense leading to usage of an alternative START codon	1	1
Fusion	244	122
Fusion (fused to promoter of another gene with in-frame deletion)	1	1
In-frame deletion	91	68
In-frame duplication	36	21
In-frame insertion	33	18
In-frame indel	21	12
Multiple in-cis mutations	12	9
Splicing	6	6
Inversion	2	2
Extension	1	1
Total	5370	4832

**Figure 3.**
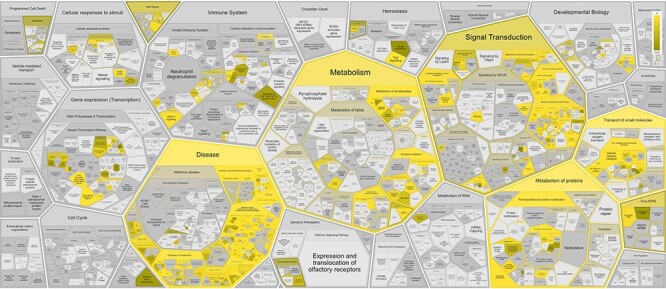
Reactome pathway enrichment analysis results shown in the Reactome Voronoi map for 372 disease variant genes based on Reactome Release 84.

Overrepresented pathways for Reactome disease genes (Supplementary Table S2, Figure 3) are often related to cancer hallmarks (38) and Mendelian disorders of metabolism, reflecting curation and experimental bias for a small fraction of protein-coding genes involved in particular biological processes (39). The overrepresentation analysis shows that 88% (326/372) of the disease variant genes are captured in the following six top-level wild type pathways: Metabolism (127/372; 34%), Signal Transduction (92/372; 25%), Metabolism of proteins (91/372; 24%), Immune System (68/372; 18%), Transport of small molecules (80/372; 22%) and Gene expression (37/372; 10%), of which Metabolism, Metabolism of proteins and Transport of small molecules are significantly enriched (corrected *P*-value < 0.05).

**Figure 4.**
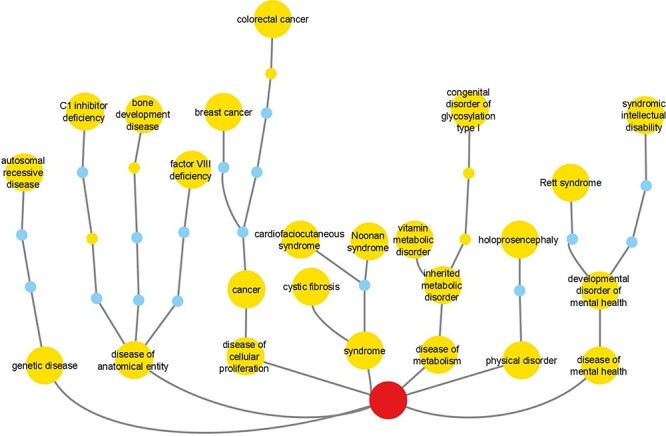
Reactome cross-references to DO branches with most frequently cross-referenced terms for each branch shown. Labeled large nodes and small unlabeled nodes of the same hue are DO terms annotated in the context of Reactome disease variants. Small unlabeled nodes of a different hue are linker nodes, connecting Reactome disease variant-annotated DO terms with the top-level DO term Disease (DOID:4), shown as the centrally positioned root node at the bottom of the picture.

Reactome disease variants cross-reference 421/11 254 DO terms (Supplementary Table S3). Of the 5370 disease variant EWASs, 2096 cross-reference more than one DO term. The most frequently cross-referenced DO terms (Figure 4, Supplementary Table S3) belong to the branches ‘disease of cellular proliferation’ (DOID:14566), ‘disease of anatomical entity’ (DOID:7) and ‘disease of metabolism’ (DOID:0014667). The top three cross-referenced terms for ‘disease of cellular proliferation’ are ‘cancer’ (DOID:162), ‘breast cancer’ (DOID:1612) and ‘colorectal cancer’ (DOID:9256). For ‘disease of anatomical entity’, the top three cross-referenced terms are ‘factor VIII deficiency’ (DOID:12134), ‘C1 inhibitor deficiency’ (DOID:0060002) and ‘bone development disease’ (DOID:0080006). For ‘disease of metabolism’, the top three cross-referenced terms are ‘congenital disorder of glycosylation type I’ (DOID:0050570), ‘inherited metabolic disorder’ (DOID:655) and ‘vitamin metabolic disorder’ (DOID:0050718).

Reactome variant annotations can lead to novel biological insights by creating explicit connections between disease terms and pathway/process-based molecular phenotypes (Supplementary Table S1), as described in the Discussion.

**Figure 5.**
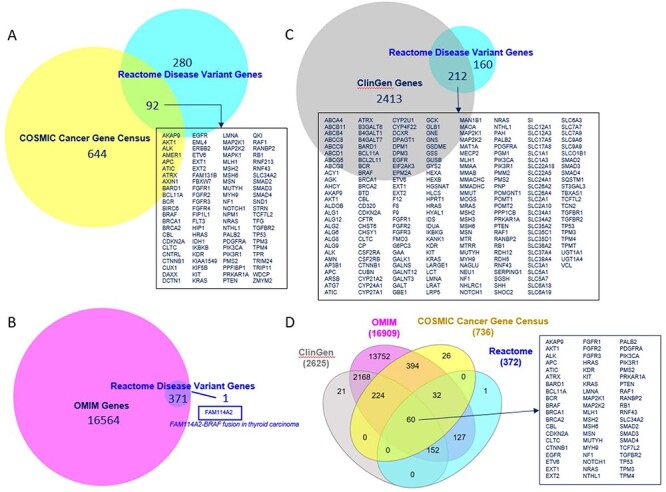
Overlap of Reactome disease variant genes with (A) COSMIC Cancer Gene Census genes, (B) OMIM disease genes, (C) ClinGen disease genes and (D) COSMIC, OMIM and ClinGen combined. Overlapping COSMIC/Reactome, ClinGen/Reactome and COSMIC/ClinGen/OMIM/Reactome genes are shown. A single disease gene present in Reactome but not in OMIM is shown.

The overlap of Reactome disease genes with COSMIC v97 Cancer Gene Census List (40), OMIM (9) and ClinGen (24) is shown in Figure 5. Sixty disease genes overlapped between all four resources. Reactome version 84 included 3804 COSMIC, 199 ClinGen, 11 ClinVar, 2 OMIM and 132 Leiden Open Variation Database identifiers. Reactome variant EWASes map to 547 disease variant sets, 801 disease reactions (cross-referencing 506 normal reactions) and 403 diagram-level disease pathways (cross-referencing 73 normal pathways linked to 51 Gene Ontology (GO) Biological Processes) (Supplementary Table S1). The average number of disease reactions a disease gene participates in is 4 (1–54 range, median 1) overall, 8 (1–54 range, median 6) for genes with GOF variants (110/372) and 2 (1–16 range, median 1) for genes with LOF variants (262/372). The discrepancy is due to the curation strategy. Reactome disease pathways start with disease reactions diverging from the wild-type and end with disease reactions with all wild-type outputs. With LOF variants, a single disease reaction is frequently the starting and ending reaction in a single disease pathway (e.g. PAH S40L in ‘Phenylketonuria’ (41)). GOF variants frequently engage in a cascade of events (e.g. AKT1 E17K in ‘PI3K/AKT Signaling in Cancer’ (29)).

### Reactome-applicable ACMG/AMP Standards and Guidelines evidence framework

Two Reactome driving principles are the annotation of disease variants (i) whose molecular functionality has been characterized and (ii) which are clinically relevant/pathogenic.

Benign variants are outside Reactome’s scope. Reactome disease variants correspond to only 6/27 ACMG/AMP criteria (PM1, PM4, PM5, PS1, PS3 and PVS1) (Table 1). ACMG/AMP criteria BS1–4 and BP1–7 can be used for the exclusion of potentially pathogenic variants. ACMG/AMP criteria PP1–PP5, PM2, PM3, PM6, PS2 and PS4 are insufficient for reaction-specific annotation but can corroborate the pathogenicity of variants conforming to PM1, PM4, PM5, PS1, PS3 or PVS1.

Currently, the ACMG/AMP-derived designations, provided in Supplementary Table S1, are not visible on the website but will be included as part of improved user interface.

#### PS3, PVS1 and PS1

**Figure 6.**
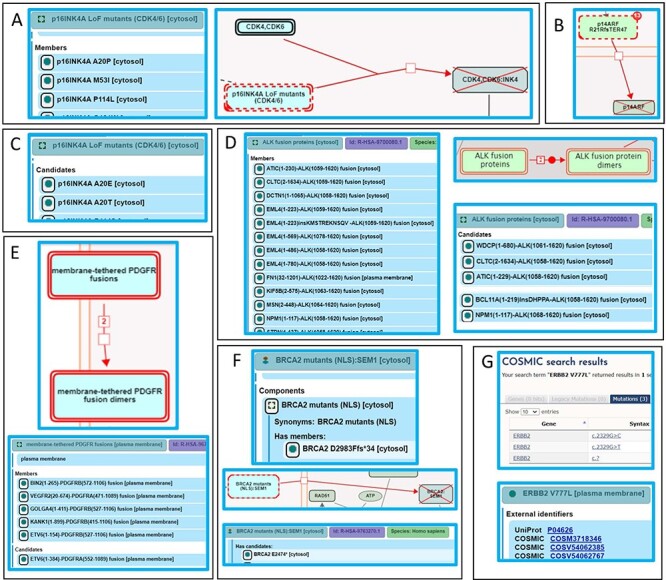
Alignment of Reactome disease variant annotations with ACMG/AMP-specified criteria. (A) PS3 variants are annotated as members of disease variant sets. Cancer variant p16INK4 A20P, unable to bind to and inhibit cyclin-dependent kinases CDK4 and CDK6, is annotated as a member of the ‘p16INK4A LoF mutants (CDK4/6)’ variant set (44). (B) PS3 variants are annotated as direct disease reaction participants. Cancer variant p14ARF R21Rfs*47 (p14ARF 60ins16) is unable to translocate to the nucleus (46). (C) PM5 variants are annotated as candidates of disease variant sets. Cancer variants p16INK4A A20E and p16INK4A A20T, sharing similarity with the functionally studied p16INK4A A20P, are candidates of the ‘p16INK4A LoF mutants (CDK4/6)’ variant set (44). (D) Extrapolation of PS3 and PM5 criteria to fusion variants. Functionally studied (PS3) cancer-associated fusions EML4(1–496)-ALK(1058–1620) and NPM1(1–117)-ALK(1058–1620) undergo ligand-independent dimerization and are annotated as direct members of the disease set ‘ALK fusion proteins’. Fusion proteins that have not been functionally characterized but are expected to behave in a similar manner based on conservation of functional domains in each fusion partner (PM5) are annotated as candidates of the ‘ALK fusion protein’ set (53). (E) Further extrapolation of PM5 criterion to fusion variants to include related family members. Fusion variant ETV6(1–154)-PDGFRB(527–1106), known to dimerize independently of ligand stimulation, is a member of the ‘membrane-tethered PDGFR fusions’ variant set, while the analogous, functionally uncharacterized fusion variant ETV6(1–384)-PDGFRA(552–1089), involving PDGFR family member PDGFRA, is a candidate (56). (F) Nonsense and frameshift variants not directly functionally studied that conform to both criteria PM1 and PM4 are annotated as candidates of disease variant sets. Cancer variant BRCA2 F2058Lfs*12 is cytosolic and, in accordance with PS3, annotated as a member of the variant set ‘BRCA2 mutants (NLS)’. Cancer variant BRCA2 E2474*, not functionally studied but similarly lacking the NLS, is annotated as a candidate (60). (G) Protein disease variants in Reactome cross-reference any PS1 disease DNA variants from relevant reference databases available at the time of annotation. Cancer variant ERBB2 V777L cross-references three applicable COSMIC records: COSV54062385, COSV54062767 and COSM3718346 (47).

PS3 variants are the gold standard for Reactome—deleterious effect(s) supported by well-established functional studies—with an additional Reactome requirement for studies to be at the molecular mechanism resolution level. PS3 variants are annotated as functionally analogous members of disease variant sets or as direct disease reaction participants. For example, a cancer-associated missense mutation in the *CDKN2A* gene produces a protein variant of one of its protein products, p16INK4A, in which alanine at position 20 is substituted with proline (p16INK4A A20P). The variant protein p16INK4 A20P is unable to bind to and inhibit cyclin-dependent kinases CDK4 and CDK6 (42, 43), thus enabling cancer cell division to proceed unchecked. As there are multiple p16INK4A variants shown to bind neither CDK4 nor CDK6, p16INK4A A20P is annotated as a member of the ‘p16INK4A LoF mutants (CDK4/6)’ variant set in the disease reaction ‘p16INK4A mutants do not bind CDK4, CDK6’, belonging to the disease pathway ‘Evasion of oncogene-induced senescence due to defective p16INK4A binding to CDK4 and CDK6’ (44) (Figure 6A). A germline mutation affecting exon 1β of the CDKN2A locus, specific to the p14ARF protein product of CDKN2A, is associated with familial melanoma syndrome. The mutation represents an insertion of 16 nucleotides (CGGCCCGCCGCGAGTG) between coding bases 60 and 61 in exon 1β. This insertion results in a frameshift, starting at arginine codon at position 21 of p14ARF and ending with a premature stop codon at position 67. The mutant protein p14ARF R21Rfs*47 (p14ARF 60ins16) is unable to translocate to the nucleus and stabilize TP53 (45). As no other similar variants of p14ARF have been reported, the p14ARF R21Rfs*47 variant is annotated as a direct participant in the disease reaction ‘p14ARF mutant does not translocate to the nucleus’, belonging to the disease pathway ‘Evasion of Oncogene Induced Senescence Due to p14ARF Defects’ (46) (Figure 6B).

Only PVS1 variants that can be annotated at the protein level are included. Unless PS3 criterion is satisfied, PVS1 variants are annotated as disease variant set candidates.

Reactome disease protein variants cross-reference available PS1 DNA variants from reference databases. For example, ERBB2 (HER2) cancer variant ERBB2 V777L is connected with three different gene mutation identifiers in COSMIC: COSV54062385 (coding DNA sequence—CDS—mutation c.2329G > C—substitution, Position 2329, G➞C), COSV54062767 (CDS mutation c.2329G > T—substitution, Position 2329, G➞T) and one legacy identifier COSM3718346 for which CDS mutation is not specified (CDS mutation c.? Unknown). Reactome links out to all three COSMIC records on the disease variant webpage in the pathway ‘Signaling by ERBB2 in Cancer’ (47), Figure 6G).

#### PM5

Reactome extends PM5 variants—novel missense changes at amino acids previously reported to be affected by pathogenic missense changes—to include variants of any type (e.g. fusion, in-frame indel, etc.) that contain mutations analogous to those in PS3 variants as Reactome disease variant set ‘candidates’. For example, Variants p16INK4A A20E and p16INK4A A20T, sharing similarity with the functionally studied p16INK4A A20P (Figure 6A), are annotated as candidates of the ‘p16INK4A LoF mutants (CDK4/6)’ variant set (Figure 6C) in the disease reaction ‘p16INK4A mutants do not bind CDK4,CDK6’, belonging to the disease pathway ‘Evasion of oncogene-induced senescence due to defective p16INK4A binding to CDK4 and CDK6’ (44).

The *ALK* locus is subject to chromosomal translocations and inversions that give rise to >25 oncogenic fusion proteins in a number of different cancers. These fusion proteins form constitutive, ligand-independent dimers on the basis of an N-terminal dimerization domain, contributed by the fusion partner and the intracellular portion of the ALK receptor tyrosine kinase that contains the catalytic domain. ‘ALK fusion proteins’ set members include EML4(1–496)-ALK(1058–1620), NPM1(1–117)-ALK(1058–1620) (48, 49) and others (48, 50, 51), which undergo ligand-independent dimerization, as described in the disease reaction ‘Ligand-independent dimerization of ALK fusions’, belonging to the disease pathway ‘Signaling by ALK in cancer’, meeting the PS3 criterion. PM5 candidates of this set include different breakpoints between PS3 fusion protein pairs, e.g. NPM1(1–117)-ALK(1068–1620) (52), or novel fusion partners, e.g. WDCP(1–680)-ALK(1061–1620), containing functional domains analogous to those in PS3 variants (Figure 6D) (53). While these ALK fusion proteins have not been functionally characterized, they can reasonably be expected to behave in a similar manner to ‘ALK fusion protein’ set members based on the conservation of functional domains in each fusion partner (that is, a known dimerization domain provided by the N-terminal partner and the kinase domain provided by the ALK protein). In the case of PS3 fusion variant of *PDGFRB*, ETV6(1–154)-PDGFRB(527–1106) (54), a member of the ‘membrane-tethered PDGFR fusions’ set, the PM5 criterion was extended to the PDGFR family member PDGFRA to include ETV6(1–384)-PDGFRA(552–1089) (55) as a candidate (Figure 6E) (56). As no HGVS guidelines exist for the naming of fusion proteins, Reactome proposes nomenclature based on the controlled vocabulary for protein fragments (57), described in Supplementary Methods.

#### PM1 and PM4

Nonsense and frameshift variants not directly functionally studied that conform to both ACMG/AMP PM1 (truncation or complete ablation of functional domains key to a particular protein function) and PM4 (protein length changing) criteria are annotated as disease variant set candidates. For example, truncating mutations are the most frequent mutations seen in *BRCA2*, a tumor-suppressor gene that localizes to the nucleus and takes part in repair of DNA double-strand breaks. Of two functional BRCA2 nuclear localization signals, NLS1 and NLS2 (Positions 3263–3269 and 3381–3385, respectively), only NLS1 is essential (58, 59). A common cancer variant, BRCA2 F2058Lfs*12 (c.6174delT), is cytosolic (58) and, in accordance with the PS3 criterion, annotated as the member of the ‘BRCA2 mutants (NLS)’ set (Figure 6F), in the disease reaction ‘Defective BRCA2 does not translocate to the nucleus’, belonging to the disease pathway ‘Defective homologous recombination repair (HRR) due to BRCA2 loss of function’ (60). Polymorphisms with intact NLS1, BRCA2 K3326* and BRCA2 E3342*, not cancer-associated (BS2) and properly localized (BS3) (58), are outside Reactome’s scope. Functionally uncharacterized cancer mutants BRCA2 E2474*, BRCA2 R2502Lfs*24 and BRCA2 N2553Tfs*95, with truncations upstream of NLS1, satisfying criteria PM1 and PM4, are annotated as ‘BRCA2 mutants (NLS)’ set candidates (60).

#### Conflicting evidence

When evidence is conflicting, functional data takes precedence in Reactome. If a variant can be categorized as benign by BP4 (computational evidence) and pathogenic by PS3 (functional evidence), as is the case with PIK3CA H1047L and PIK3CA H1047R (20, 21), it is annotated as a PS3 variant (29). If a variant can be categorized as benign by BS3 (functional evidence) and pathogenic by PM5 (computational evidence), it is excluded from Reactome. PS3 variant p16INK4A D74Y, unable to bind and inhibit CDK4 (61) and restrict cellular proliferation (62), has a PM5 variant p16INK4A D74N which retains the ability to bind CDK4 and CDK6 (63) (BS3), although with diminished ability to inhibit cellular proliferation and CDK4/6 catalytic activity (63) (PS3). As the LOF mechanism is uncertain, p16INK4A D74N is waitlisted for future research community contributions (64) and updates (44).

### Retrieval of Reactome disease variant knowledge

**Figure 7.**
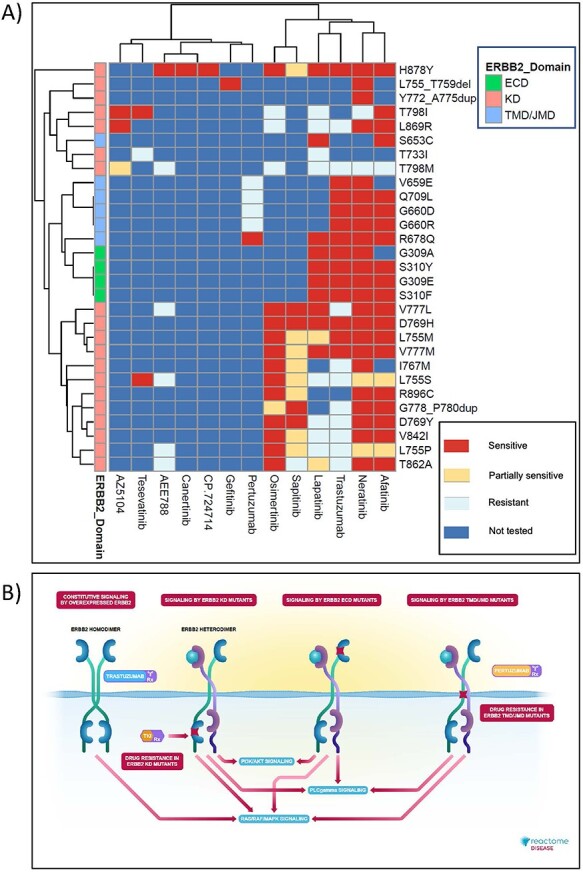
High-level graphical summary of Reactome’s ERBB2 cancer variants content. (A) Heatmap representation of Reactome electronic textbook knowledge on the sensitivity of different ERBB2 cancer variants to ERBB2-targeted anti-cancer therapeutics. The heatmap was generated using the R package pheatmap with default settings. (B) Interactive textbook style diagram for ‘Signaling by ERBB2 in Cancer’ pathway.

Reactome’s disease variant knowledge can be retrieved in three formats: interactive (based on the Reactome’s homepage search function (31)), electronic textbook (https://reactome.org/download/current/TheReactomeBook.pdf.tgz, with heatmap and textbook-style illustration representations (65) shown in Figure 7) and comprehensive tabular (via the Cypher query provided in the Supplementary Methods of the Reactome Graph Database available from the Downloads page, https://reactome.org/download-data, along with Neo4J installation instructions; after Neo4J installation, Awesome Procedures on Cypher plugin needs to be installed to run the Cypher query).

## Discussion

The greatest challenges in predicting pathogenic effects of newly discovered disease-associated variants are the scarcity of mechanistic protein-level evidence, and conflicting predictions by different algorithms. Reactome’s reaction-specific, pathway-based, experimentally supported, peer-reviewed disease variant annotations can be used as a gold-standard dataset for computational pathogenicity assessment based on machine learning or similar approaches, e.g. AlphaMissense (19).

Truncating variants are commonly interpreted as LOF, but mutation position (66) and the surrounding sequence landscape (67) affect the likelihood of nonsense-mediated decay. The role of truncated/obliterated protein domains also has to be considered. NOTCH1 PEST domain truncations enhance NOTCH1 oncogenic signaling by interfering with ubiquitin-mediated degradation (68–71).

Many variants of unknown significance have been annotated as Reactome disease variant set candidates by extrapolating wild-type protein domain knowledge and experimental findings from PS3 to analogous variants. The Reactome pipeline for bulk automated annotation of pre-selected candidate variants (Supplementary Methods) will enable future automated variants of unknown significance projection onto Reactome pathways. This requires higher resolution annotations of wild-type protein domains and critical amino acid residues. The large-scale, highly accurate protein 3D structures predicted by AlphaFold (72) may facilitate this type of annotation.

Reactome relies on external variant databases and disease/phenotype ontologies for comprehensive clinical information. While continuous communication across databases is necessary for maintaining data integrity, and while open-source databases strive to adhere to FAIR principles (73), lack of standardization hampers FAIRness. Large disease variant repositories do not mandate compliance of disease terms with standardized ontologies, such as DO (25), Monarch Disease Ontology (74) and Human Phenotype Ontology (75). Scientific journals largely do not mandate HGVS nomenclature (15). Some HGVS guidelines are complex and hard to follow, e.g. nomenclature of initiating methionine missense variants with many synonymous alternatives. For some groups of variants, especially fusion proteins, HGVS guidelines are lacking. Reactome’s fusion protein nomenclature system can improve the standardization of fusion protein annotations by HGVS and emerging fusion protein databases FusionGDB (76), CIViC (77) and FPIA (78).

To improve the organization of disease variant content and its FAIR compliance, Reactome will provide standardized molecular phenotypes for annotated variants, derived from the entity functional status of relevant disease reactions (e.g. LOF or GOF), and the GO Biological Process of the relevant normal pathway counterpart (Supplementary Table S1), thus bridging GO Biological Processes and terms from disease/phenotype ontologies. In addition, Reactome is developing more sophisticated User Guide GraphQL protocols (https://reactome.org/userguide), planning a disease filter for search results, adding the comprehensive disease variant dataset table to its Downloads page, working to systematically apply ClinGen/ClinVar cross-references to all applicable disease variants along with ACMG/AMP pathogenicity criteria tags, developing a protocol for contributing its disease variant annotations to open-source disease variant databases, improving its pipeline for periodic updates of external cross-references and taking part in disease gene curation community initiatives, such as Gene Curation Coalition (79) and Database of Chromosomal Imbalance and Phenotype in Humans Using Ensembl Resources (80).

## Supplementary Material

baae031_Supp

## Data Availability

Supplementary Tables and Supplementary Methods, the latter including the script used to generate the table of human diseases variants in Reactome, are available at Database online as Word documents (please refer to the Supplementary Data section). Supplementary Tables as Excel files and Supplementary Methods as PDF file are available at Zenodo: https://zenodo.org/records/8069693. Reactome data is available at https://reactome.org/.
