# Supplementary material for: Pathway-based, reaction-specific annotation of disease variants for elucidation of molecular phenotypes
Source: Database (Oxford). 2024 May 7;2024:baae031. doi: 10.1093/database/baae031 (PMC11184451; doi:10.1093/database/baae031)
Supplement: baae031_Supp [file baae031_supp.zip › suppl_data/Database_Orlic_Milacic_Reactome_of_Disease_Variants_SupplementaryTable1.docx]

ACMG_AMP_Criterion Genename displayName stable_id referenceEntity_name referenceEntity_id hasModifiedResidue_displayName modifiedResidue_class cross_reference disease disease_identifier entityWithAccessionedSequence_literatureReference_pubMedIdentifier first_entitySet entityWithAccessionedSequence_reactionLikeEvent_stable_id entityWithAccessionedSequence_reactionLikeEvent_displayName entityWithAccessionedSequence_reactionLikeEvent_entityFunctionalStatus_functionalStatus_functionalStatusType_displayName reactionLikeEvent_literatureReference_pubMedIdentifier entityWithAccessionedSequence_pathway_stable_id entityWithAccessionedSequence_pathway_displayName entityWithAccessionedSequence_reactionLikeEvent_normalReaction_stable_id entityWithAccessionedSequence_reactionLikeEvent_normalReaction_displayName entityWithAccessionedSequence_pathway_normalPathway_displayName entityWithAccessionedSequence_pathway_normalPathway_stable_id normal_reaction_like_event_go_biological_process_accession normal_reaction_like_event_go_biological_process_displayName entityWithAccessionedSequence_pathway_normalPathway_goBiologicalProcess_accession entityWithAccessionedSequence_pathway_normalPathway_goBiologicalProcess_displayName Disease Variant Type Molecular Phenotype

PS3 ABCA1 ABCA1 W590S [plasma membrane] R-HSA-5682201 ABCA1 UniProt:O95477 L-tryptophan 590 replaced with L-serine ReplacedResidue Tangier disease DOID:1388 R-HSA-5682194-ABCA1 mutants [plasma membrane]-DefinedSet-hasMember R-HSA-5682111 Defective ABCA1 does not transport CHOL from transport vesicle membrane to plasma membrane loss_of_function pubmed:12509412|pubmed:10431236|pubmed:11476961|pubmed:12111381 R-HSA-5682113 Defective ABCA1 causes TGD R-HSA-216723 4xPALM-C-p-2S-ABCA1 tetramer transports CHOL from transport vesicle membrane to plasma membrane "Plasma lipoprotein assembly, remodeling, and clearance" R-HSA-174824 71827 plasma lipoprotein particle organization missense loss_of_function of plasma lipoprotein particle organization

PS3 ABCA1 ABCA1 N935S [plasma membrane] R-HSA-5682200 ABCA1 UniProt:O95477 L-asparagine 935 replaced with L-serine ReplacedResidue Tangier disease DOID:1388 R-HSA-5682194-ABCA1 mutants [plasma membrane]-DefinedSet-hasMember R-HSA-5682111 Defective ABCA1 does not transport CHOL from transport vesicle membrane to plasma membrane loss_of_function pubmed:12509412|pubmed:10431236|pubmed:11476961|pubmed:12111381 R-HSA-5682113 Defective ABCA1 causes TGD R-HSA-216723 4xPALM-C-p-2S-ABCA1 tetramer transports CHOL from transport vesicle membrane to plasma membrane "Plasma lipoprotein assembly, remodeling, and clearance" R-HSA-174824 71827 plasma lipoprotein particle organization missense loss_of_function of plasma lipoprotein particle organization

PS3 ABCA1 ABCA1 C1417R [plasma membrane] R-HSA-5682190 ABCA1 UniProt:O95477 L-cysteine 1417 replaced with L-arginine ReplacedResidue Tangier disease DOID:1388 R-HSA-5682194-ABCA1 mutants [plasma membrane]-DefinedSet-hasMember R-HSA-5682111 Defective ABCA1 does not transport CHOL from transport vesicle membrane to plasma membrane loss_of_function pubmed:12509412|pubmed:10431236|pubmed:11476961|pubmed:12111381 R-HSA-5682113 Defective ABCA1 causes TGD R-HSA-216723 4xPALM-C-p-2S-ABCA1 tetramer transports CHOL from transport vesicle membrane to plasma membrane "Plasma lipoprotein assembly, remodeling, and clearance" R-HSA-174824 71827 plasma lipoprotein particle organization missense loss_of_function of plasma lipoprotein particle organization

PS3 ABCA1 ABCA1 S1446L [plasma membrane] R-HSA-5682195 ABCA1 UniProt:O95477 L-serine 1446 replaced with L-leucine ReplacedResidue Tangier disease DOID:1388 R-HSA-5682194-ABCA1 mutants [plasma membrane]-DefinedSet-hasMember R-HSA-5682111 Defective ABCA1 does not transport CHOL from transport vesicle membrane to plasma membrane loss_of_function pubmed:12509412|pubmed:10431236|pubmed:11476961|pubmed:12111381 R-HSA-5682113 Defective ABCA1 causes TGD R-HSA-216723 4xPALM-C-p-2S-ABCA1 tetramer transports CHOL from transport vesicle membrane to plasma membrane "Plasma lipoprotein assembly, remodeling, and clearance" R-HSA-174824 71827 plasma lipoprotein particle organization missense loss_of_function of plasma lipoprotein particle organization

PS3 ABCA1 ABCA1 R587W [plasma membrane] R-HSA-5682197 ABCA1 UniProt:O95477 L-arginine 587 replaced with L-tryptophan ReplacedResidue Tangier disease DOID:1388 R-HSA-5682194-ABCA1 mutants [plasma membrane]-DefinedSet-hasMember R-HSA-5682111 Defective ABCA1 does not transport CHOL from transport vesicle membrane to plasma membrane loss_of_function pubmed:12509412|pubmed:10431236|pubmed:11476961|pubmed:12111381 R-HSA-5682113 Defective ABCA1 causes TGD R-HSA-216723 4xPALM-C-p-2S-ABCA1 tetramer transports CHOL from transport vesicle membrane to plasma membrane "Plasma lipoprotein assembly, remodeling, and clearance" R-HSA-174824 71827 plasma lipoprotein particle organization missense loss_of_function of plasma lipoprotein particle organization

PS3 ABCA1 ABCA1 Q537R [plasma membrane] R-HSA-5682199 ABCA1 UniProt:O95477 L-glutamine 537 replaced with L-arginine ReplacedResidue Tangier disease DOID:1388 R-HSA-5682194-ABCA1 mutants [plasma membrane]-DefinedSet-hasMember R-HSA-5682111 Defective ABCA1 does not transport CHOL from transport vesicle membrane to plasma membrane loss_of_function pubmed:12509412|pubmed:10431236|pubmed:11476961|pubmed:12111381 R-HSA-5682113 Defective ABCA1 causes TGD R-HSA-216723 4xPALM-C-p-2S-ABCA1 tetramer transports CHOL from transport vesicle membrane to plasma membrane "Plasma lipoprotein assembly, remodeling, and clearance" R-HSA-174824 71827 plasma lipoprotein particle organization missense loss_of_function of plasma lipoprotein particle organization

PS3 ABCA12 ABCA12 V2442Sfs*22 [plasma membrane] R-HSA-5682350 ABCA12 UniProt:Q86UK0 Replacement of residues 2442 to 2462 by SSSHLTAWKNVKLSVPGWPLW FragmentReplacedModification autosomal recessive congenital ichthyosis DOID:0060655 R-HSA-5682322-ABCA12 mutants [plasma membrane]-DefinedSet-hasMember R-HSA-5682311 Defective ABCA12 does not transport lipids from cytosol to extracellular region loss_of_function pubmed:16902423|pubmed:15756637|pubmed:19664001 R-HSA-5682294 Defective ABCA12 causes ARCI4B R-HSA-5682285 ABCA12 transports lipids from cytosol to extracellular region ABC-family proteins mediated transport R-HSA-382556 55085 transmembrane transport frameshift loss_of_function of transmembrane transport

PS3 ABCA12 ABCA12 K1671Ifs*4 [plasma membrane] R-HSA-5682320 ABCA12 UniProt:Q86UK0 Replacement of residues 1671 to 1673 by IVL FragmentReplacedModification autosomal recessive congenital ichthyosis DOID:0060655 R-HSA-5682322-ABCA12 mutants [plasma membrane]-DefinedSet-hasMember R-HSA-5682311 Defective ABCA12 does not transport lipids from cytosol to extracellular region loss_of_function pubmed:16902423|pubmed:15756637|pubmed:19664001 R-HSA-5682294 Defective ABCA12 causes ARCI4B R-HSA-5682285 ABCA12 transports lipids from cytosol to extracellular region ABC-family proteins mediated transport R-HSA-382556 55085 transmembrane transport frameshift loss_of_function of transmembrane transport

PS3 ABCA12 ABCA12 D2363N [plasma membrane] R-HSA-5682291 ABCA12 UniProt:Q86UK0 L-aspartic acid 2363 replaced with L-asparagine ReplacedResidue autosomal recessive congenital ichthyosis DOID:0060655 R-HSA-5682322-ABCA12 mutants [plasma membrane]-DefinedSet-hasMember R-HSA-5682311 Defective ABCA12 does not transport lipids from cytosol to extracellular region loss_of_function pubmed:16902423|pubmed:15756637|pubmed:19664001 R-HSA-5682294 Defective ABCA12 causes ARCI4B R-HSA-5682285 ABCA12 transports lipids from cytosol to extracellular region ABC-family proteins mediated transport R-HSA-382556 55085 transmembrane transport missense loss_of_function of transmembrane transport

PS3 ABCA12 ABCA12 R287* [plasma membrane] R-HSA-5682308 ABCA12 UniProt:Q86UK0 Nonsense mutation at L-arginine 287 NonsenseMutation autosomal recessive congenital ichthyosis DOID:0060655 R-HSA-5682322-ABCA12 mutants [plasma membrane]-DefinedSet-hasMember R-HSA-5682311 Defective ABCA12 does not transport lipids from cytosol to extracellular region loss_of_function pubmed:16902423|pubmed:15756637|pubmed:19664001 R-HSA-5682294 Defective ABCA12 causes ARCI4B R-HSA-5682285 ABCA12 transports lipids from cytosol to extracellular region ABC-family proteins mediated transport R-HSA-382556 55085 transmembrane transport nonsense loss_of_function of transmembrane transport

PS3 ABCA12 ABCA12 G1179R [plasma membrane] R-HSA-5682356 ABCA12 UniProt:Q86UK0 glycine 1179 replaced with L-arginine ReplacedResidue autosomal recessive congenital ichthyosis DOID:0060655 R-HSA-5682322-ABCA12 mutants [plasma membrane]-DefinedSet-hasMember R-HSA-5682311 Defective ABCA12 does not transport lipids from cytosol to extracellular region loss_of_function pubmed:16902423|pubmed:15756637|pubmed:19664001 R-HSA-5682294 Defective ABCA12 causes ARCI4B R-HSA-5682285 ABCA12 transports lipids from cytosol to extracellular region ABC-family proteins mediated transport R-HSA-382556 55085 transmembrane transport missense loss_of_function of transmembrane transport

PS3 ABCA3 ABCA3 T1173R [lamellar body membrane] R-HSA-5683705 ABCA3 UniProt:Q99758 L-threonine 1173 replaced with L-arginine ReplacedResidue newborn respiratory distress syndrome DOID:12716 R-HSA-5683704-ABCA3 mutants [lamellar body membrane]-DefinedSet-hasMember R-HSA-5683672 "Defective ABCA3 does not transport PC, PG from ER membrane to lamellar body" loss_of_function pubmed:17719949|pubmed:22068586|pubmed:15044640 R-HSA-5683678 Defective ABCA3 causes SMDP3 R-HSA-5683714 "ABCA3 transports PC, PG from ER membrane to lamellar body" ABC-family proteins mediated transport R-HSA-382556 55085 transmembrane transport missense loss_of_function of transmembrane transport

PS3 ABCA3 ABCA3 W1142* [lamellar body membrane] R-HSA-5683691 ABCA3 UniProt:Q99758 Nonsense mutation at L-tryptophan 1142 NonsenseMutation newborn respiratory distress syndrome DOID:12716 R-HSA-5683704-ABCA3 mutants [lamellar body membrane]-DefinedSet-hasMember R-HSA-5683672 "Defective ABCA3 does not transport PC, PG from ER membrane to lamellar body" loss_of_function pubmed:17719949|pubmed:22068586|pubmed:15044640 R-HSA-5683678 Defective ABCA3 causes SMDP3 R-HSA-5683714 "ABCA3 transports PC, PG from ER membrane to lamellar body" ABC-family proteins mediated transport R-HSA-382556 55085 transmembrane transport nonsense loss_of_function of transmembrane transport

PS3 ABCA3 ABCA3 L101P [lamellar body membrane] R-HSA-5683658 ABCA3 UniProt:Q99758 L-leucine 101 replaced with L-proline ReplacedResidue newborn respiratory distress syndrome DOID:12716 R-HSA-5683704-ABCA3 mutants [lamellar body membrane]-DefinedSet-hasMember R-HSA-5683672 "Defective ABCA3 does not transport PC, PG from ER membrane to lamellar body" loss_of_function pubmed:17719949|pubmed:22068586|pubmed:15044640 R-HSA-5683678 Defective ABCA3 causes SMDP3 R-HSA-5683714 "ABCA3 transports PC, PG from ER membrane to lamellar body" ABC-family proteins mediated transport R-HSA-382556 55085 transmembrane transport missense loss_of_function of transmembrane transport

PS3 ABCA3 ABCA3 D253H [lamellar body membrane] R-HSA-5683697 ABCA3 UniProt:Q99758 L-aspartic acid 253 replaced with L-histidine ReplacedResidue newborn respiratory distress syndrome DOID:12716 R-HSA-5683704-ABCA3 mutants [lamellar body membrane]-DefinedSet-hasMember R-HSA-5683672 "Defective ABCA3 does not transport PC, PG from ER membrane to lamellar body" loss_of_function pubmed:17719949|pubmed:22068586|pubmed:15044640 R-HSA-5683678 Defective ABCA3 causes SMDP3 R-HSA-5683714 "ABCA3 transports PC, PG from ER membrane to lamellar body" ABC-family proteins mediated transport R-HSA-382556 55085 transmembrane transport missense loss_of_function of transmembrane transport

PS3 ABCA3 ABCA3 L326P [lamellar body membrane] R-HSA-5683666 ABCA3 UniProt:Q99758 L-leucine 326 replaced with L-proline ReplacedResidue newborn respiratory distress syndrome DOID:12716 R-HSA-5683704-ABCA3 mutants [lamellar body membrane]-DefinedSet-hasMember R-HSA-5683672 "Defective ABCA3 does not transport PC, PG from ER membrane to lamellar body" loss_of_function pubmed:17719949|pubmed:22068586|pubmed:15044640 R-HSA-5683678 Defective ABCA3 causes SMDP3 R-HSA-5683714 "ABCA3 transports PC, PG from ER membrane to lamellar body" ABC-family proteins mediated transport R-HSA-382556 55085 transmembrane transport missense loss_of_function of transmembrane transport

PS3 ABCA3 ABCA3 L1553P [lamellar body membrane] R-HSA-5683671 ABCA3 UniProt:Q99758 L-leucine 1553 replaced with L-proline ReplacedResidue newborn respiratory distress syndrome DOID:12716 R-HSA-5683704-ABCA3 mutants [lamellar body membrane]-DefinedSet-hasMember R-HSA-5683672 "Defective ABCA3 does not transport PC, PG from ER membrane to lamellar body" loss_of_function pubmed:17719949|pubmed:22068586|pubmed:15044640 R-HSA-5683678 Defective ABCA3 causes SMDP3 R-HSA-5683714 "ABCA3 transports PC, PG from ER membrane to lamellar body" ABC-family proteins mediated transport R-HSA-382556 55085 transmembrane transport missense loss_of_function of transmembrane transport

PS3 ABCA3 ABCA3 E292V [lamellar body membrane] R-HSA-5688394 ABCA3 UniProt:Q99758 L-glutamic acid 292 replaced with L-valine ReplacedResidue newborn respiratory distress syndrome|interstitial lung disease DOID:12716|DOID:3082 R-HSA-5688403-ABCA3 mutants [lamellar body membrane]-DefinedSet-hasMember R-HSA-5688397 "Defective ABCA3 does not transport PC, PG from ER membrane to lamellar body" loss_of_function pubmed:18676873|pubmed:22434821|pubmed:16959783|pubmed:15044640 R-HSA-5688399 Defective ABCA3 causes SMDP3 R-HSA-5683714 "ABCA3 transports PC, PG from ER membrane to lamellar body" Surfactant metabolism R-HSA-5683826 missense NA

PS3 ABCA3 ABCA3 E690K [lamellar body membrane] R-HSA-5688389 ABCA3 UniProt:Q99758 L-glutamic acid 690 replaced with L-lysine ReplacedResidue newborn respiratory distress syndrome|interstitial lung disease DOID:12716|DOID:3082 R-HSA-5688403-ABCA3 mutants [lamellar body membrane]-DefinedSet-hasMember R-HSA-5688397 "Defective ABCA3 does not transport PC, PG from ER membrane to lamellar body" loss_of_function pubmed:18676873|pubmed:22434821|pubmed:16959783|pubmed:15044640 R-HSA-5688399 Defective ABCA3 causes SMDP3 R-HSA-5683714 "ABCA3 transports PC, PG from ER membrane to lamellar body" Surfactant metabolism R-HSA-5683826 missense NA

PS3 ABCA3 ABCA3 N568D [lamellar body membrane] R-HSA-5688388 ABCA3 UniProt:Q99758 L-asparagine 568 replaced with L-aspartic acid ReplacedResidue newborn respiratory distress syndrome|interstitial lung disease DOID:12716|DOID:3082 R-HSA-5688403-ABCA3 mutants [lamellar body membrane]-DefinedSet-hasMember R-HSA-5688397 "Defective ABCA3 does not transport PC, PG from ER membrane to lamellar body" loss_of_function pubmed:18676873|pubmed:22434821|pubmed:16959783|pubmed:15044640 R-HSA-5688399 Defective ABCA3 causes SMDP3 R-HSA-5683714 "ABCA3 transports PC, PG from ER membrane to lamellar body" Surfactant metabolism R-HSA-5683826 missense NA

PS3 ABCA3 ABCA3 T1114M [lamellar body membrane] R-HSA-5688390 ABCA3 UniProt:Q99758 L-threonine 1114 replaced with L-methionine ReplacedResidue newborn respiratory distress syndrome|interstitial lung disease DOID:12716|DOID:3082 R-HSA-5688403-ABCA3 mutants [lamellar body membrane]-DefinedSet-hasMember R-HSA-5688397 "Defective ABCA3 does not transport PC, PG from ER membrane to lamellar body" loss_of_function pubmed:18676873|pubmed:22434821|pubmed:16959783|pubmed:15044640 R-HSA-5688399 Defective ABCA3 causes SMDP3 R-HSA-5683714 "ABCA3 transports PC, PG from ER membrane to lamellar body" Surfactant metabolism R-HSA-5683826 missense NA

PS3 ABCA4 ABCA4 R943Q [photoreceptor disc membrane] R-HSA-2466827 ABCA4 UniProt:P78363 L-arginine 943 replaced with L-glutamine ReplacedResidue macular degeneration DOID:4448 R-HSA-2466752-ABCA4 mutants [photoreceptor disc membrane]-DefinedSet-hasMember R-HSA-2466802 Defective ABCA4 does not transport NRPE from disc membranes loss_of_function pubmed:19553623|pubmed:9054934|pubmed:19304658|pubmed:9781034 R-HSA-2453864 Retinoid cycle disease events R-HSA-2466749 ABCA4 transports NRPE from photoreceptor outer segment membrane to cytosol Visual phototransduction R-HSA-2187338 7603 "phototransduction, visible light" missense "loss_of_function of phototransduction, visible light"

PS3 ABCA4 ABCA4 G1961E [photoreceptor disc membrane] R-HSA-2466780 ABCA4 UniProt:P78363 glycine 1961 replaced with L-glutamic acid ReplacedResidue macular degeneration DOID:4448 R-HSA-2466752-ABCA4 mutants [photoreceptor disc membrane]-DefinedSet-hasMember R-HSA-2466802 Defective ABCA4 does not transport NRPE from disc membranes loss_of_function pubmed:19553623|pubmed:9054934|pubmed:19304658|pubmed:9781034 R-HSA-2453864 Retinoid cycle disease events R-HSA-2466749 ABCA4 transports NRPE from photoreceptor outer segment membrane to cytosol Visual phototransduction R-HSA-2187338 7603 "phototransduction, visible light" missense "loss_of_function of phototransduction, visible light"

PS3 ABCA4 ABCA4 A1028V [photoreceptor disc membrane] R-HSA-2466850 ABCA4 UniProt:P78363 L-alanine 1028 replaced with L-valine ReplacedResidue macular degeneration DOID:4448 R-HSA-2466752-ABCA4 mutants [photoreceptor disc membrane]-DefinedSet-hasMember R-HSA-2466802 Defective ABCA4 does not transport NRPE from disc membranes loss_of_function pubmed:19553623|pubmed:9054934|pubmed:19304658|pubmed:9781034 R-HSA-2453864 Retinoid cycle disease events R-HSA-2466749 ABCA4 transports NRPE from photoreceptor outer segment membrane to cytosol Visual phototransduction R-HSA-2187338 7603 "phototransduction, visible light" missense "loss_of_function of phototransduction, visible light"

PS3 ABCA4 ABCA4 G863A [photoreceptor disc membrane] R-HSA-2466821 ABCA4 UniProt:P78363 glycine 863 replaced with L-alanine ReplacedResidue macular degeneration DOID:4448 R-HSA-2466752-ABCA4 mutants [photoreceptor disc membrane]-DefinedSet-hasMember R-HSA-2466802 Defective ABCA4 does not transport NRPE from disc membranes loss_of_function pubmed:19553623|pubmed:9054934|pubmed:19304658|pubmed:9781034 R-HSA-2453864 Retinoid cycle disease events R-HSA-2466749 ABCA4 transports NRPE from photoreceptor outer segment membrane to cytosol Visual phototransduction R-HSA-2187338 7603 "phototransduction, visible light" missense "loss_of_function of phototransduction, visible light"

PS3 ABCB11 ABCB11 R1057* [plasma membrane] R-HSA-5678521 ABCB11 UniProt:O95342 Nonsense mutation at L-arginine 1057 NonsenseMutation intrahepatic cholestasis DOID:1852 R-HSA-5678515-ABCB11 mutants [plasma membrane]-DefinedSet-hasMember R-HSA-5678517 Defective ABCB11 does not transport bile salts from cytosol to extracellular region loss_of_function pubmed:9806540|pubmed:16039748 R-HSA-5678520 Defective ABCB11 causes PFIC2 and BRIC2 R-HSA-193362 ABCB11 transports bile salts from cytosol to extracellular region Bile acid and bile salt metabolism R-HSA-194068 8206 bile acid metabolic process nonsense loss_of_function of bile acid metabolic process

PS3 ABCB11 ABCB11 R575* [plasma membrane] R-HSA-5678506 ABCB11 UniProt:O95342 Nonsense mutation at L-arginine 575 NonsenseMutation intrahepatic cholestasis DOID:1852 R-HSA-5678515-ABCB11 mutants [plasma membrane]-DefinedSet-hasMember R-HSA-5678517 Defective ABCB11 does not transport bile salts from cytosol to extracellular region loss_of_function pubmed:9806540|pubmed:16039748 R-HSA-5678520 Defective ABCB11 causes PFIC2 and BRIC2 R-HSA-193362 ABCB11 transports bile salts from cytosol to extracellular region Bile acid and bile salt metabolism R-HSA-194068 8206 bile acid metabolic process nonsense loss_of_function of bile acid metabolic process

PS3 ABCB11 ABCB11 R432T [plasma membrane] R-HSA-5678509 ABCB11 UniProt:O95342 L-arginine 432 replaced with L-threonine ReplacedResidue intrahepatic cholestasis DOID:1852 R-HSA-5678515-ABCB11 mutants [plasma membrane]-DefinedSet-hasMember R-HSA-5678517 Defective ABCB11 does not transport bile salts from cytosol to extracellular region loss_of_function pubmed:9806540|pubmed:16039748 R-HSA-5678520 Defective ABCB11 causes PFIC2 and BRIC2 R-HSA-193362 ABCB11 transports bile salts from cytosol to extracellular region Bile acid and bile salt metabolism R-HSA-194068 8206 bile acid metabolic process missense loss_of_function of bile acid metabolic process

PS3 ABCB11 ABCB11 E297G [plasma membrane] R-HSA-5678500 ABCB11 UniProt:O95342 L-glutamic acid 297 replaced with glycine ReplacedResidue intrahepatic cholestasis DOID:1852 R-HSA-5678515-ABCB11 mutants [plasma membrane]-DefinedSet-hasMember R-HSA-5678517 Defective ABCB11 does not transport bile salts from cytosol to extracellular region loss_of_function pubmed:9806540|pubmed:16039748 R-HSA-5678520 Defective ABCB11 causes PFIC2 and BRIC2 R-HSA-193362 ABCB11 transports bile salts from cytosol to extracellular region Bile acid and bile salt metabolism R-HSA-194068 8206 bile acid metabolic process missense loss_of_function of bile acid metabolic process

PS3 ABCB4 ABCB4 P1161S [plasma membrane] R-HSA-5678777 ABCB4 UniProt:P21439 L-proline 1161 replaced with L-serine ReplacedResidue cholelithiasis DOID:10211 R-HSA-5678836-ABCB4 mutants [plasma membrane]-DefinedSet-hasMember R-HSA-5678749 Defective ABCB4 does not transport PC from plasma membrane to extracellular region loss_of_function pubmed:17726488|pubmed:11313316|pubmed:10767346|pubmed:9923886|pubmed:18482588|pubmed:19584064|pubmed:9419367 R-HSA-5678771 "Defective ABCB4 causes PFIC3, ICP3 and GBD1" R-HSA-5678706 ABCB4 transports PC from plasma membrane to extracellular region ABC-family proteins mediated transport R-HSA-382556 55085 transmembrane transport missense loss_of_function of transmembrane transport

PS3 ABCB4 ABCB4 R590Q [plasma membrane] R-HSA-5678907 ABCB4 UniProt:P21439 L-arginine 590 replaced with L-glutamine ReplacedResidue intrahepatic cholestasis DOID:1852 R-HSA-5678836-ABCB4 mutants [plasma membrane]-DefinedSet-hasMember R-HSA-5678749 Defective ABCB4 does not transport PC from plasma membrane to extracellular region loss_of_function pubmed:17726488|pubmed:11313316|pubmed:10767346|pubmed:9923886|pubmed:18482588|pubmed:19584064|pubmed:9419367 R-HSA-5678771 "Defective ABCB4 causes PFIC3, ICP3 and GBD1" R-HSA-5678706 ABCB4 transports PC from plasma membrane to extracellular region ABC-family proteins mediated transport R-HSA-382556 55085 transmembrane transport missense loss_of_function of transmembrane transport

PS3 ABCB4 ABCB4 Y403H [plasma membrane] R-HSA-5678826 ABCB4 UniProt:P21439 L-tyrosine 403 replaced with L-histidine ReplacedResidue intrahepatic cholestasis DOID:1852 R-HSA-5678836-ABCB4 mutants [plasma membrane]-DefinedSet-hasMember R-HSA-5678749 Defective ABCB4 does not transport PC from plasma membrane to extracellular region loss_of_function pubmed:17726488|pubmed:11313316|pubmed:10767346|pubmed:9923886|pubmed:18482588|pubmed:19584064|pubmed:9419367 R-HSA-5678771 "Defective ABCB4 causes PFIC3, ICP3 and GBD1" R-HSA-5678706 ABCB4 transports PC from plasma membrane to extracellular region ABC-family proteins mediated transport R-HSA-382556 55085 transmembrane transport missense loss_of_function of transmembrane transport

PS3 ABCB4 ABCB4 R957* [plasma membrane] R-HSA-5678748 ABCB4 UniProt:P21439 Nonsense mutation at L-arginine 957 NonsenseMutation intrahepatic cholestasis DOID:1852 R-HSA-5678836-ABCB4 mutants [plasma membrane]-DefinedSet-hasMember R-HSA-5678749 Defective ABCB4 does not transport PC from plasma membrane to extracellular region loss_of_function pubmed:17726488|pubmed:11313316|pubmed:10767346|pubmed:9923886|pubmed:18482588|pubmed:19584064|pubmed:9419367 R-HSA-5678771 "Defective ABCB4 causes PFIC3, ICP3 and GBD1" R-HSA-5678706 ABCB4 transports PC from plasma membrane to extracellular region ABC-family proteins mediated transport R-HSA-382556 55085 transmembrane transport nonsense loss_of_function of transmembrane transport

PS3 ABCB4 ABCB4 V571Dfs*16 [plasma membrane] R-HSA-5678814 ABCB4 UniProt:P21439 Replacement of residues 571 to 585 by DRQLWIRPEKAGPPL FragmentReplacedModification intrahepatic cholestasis DOID:1852 R-HSA-5678836-ABCB4 mutants [plasma membrane]-DefinedSet-hasMember R-HSA-5678749 Defective ABCB4 does not transport PC from plasma membrane to extracellular region loss_of_function pubmed:17726488|pubmed:11313316|pubmed:10767346|pubmed:9923886|pubmed:18482588|pubmed:19584064|pubmed:9419367 R-HSA-5678771 "Defective ABCB4 causes PFIC3, ICP3 and GBD1" R-HSA-5678706 ABCB4 transports PC from plasma membrane to extracellular region ABC-family proteins mediated transport R-HSA-382556 55085 transmembrane transport frameshift loss_of_function of transmembrane transport

PS3 ABCB4 ABCB4 T175V [plasma membrane] R-HSA-5678865 ABCB4 UniProt:P21439 L-threonine 175 replaced with L-valine ReplacedResidue cholelithiasis DOID:10211 R-HSA-5678836-ABCB4 mutants [plasma membrane]-DefinedSet-hasMember R-HSA-5678749 Defective ABCB4 does not transport PC from plasma membrane to extracellular region loss_of_function pubmed:17726488|pubmed:11313316|pubmed:10767346|pubmed:9923886|pubmed:18482588|pubmed:19584064|pubmed:9419367 R-HSA-5678771 "Defective ABCB4 causes PFIC3, ICP3 and GBD1" R-HSA-5678706 ABCB4 transports PC from plasma membrane to extracellular region ABC-family proteins mediated transport R-HSA-382556 55085 transmembrane transport missense loss_of_function of transmembrane transport

PS3 ABCB4 ABCB4 R545G [plasma membrane] R-HSA-5678946 ABCB4 UniProt:P21439 L-arginine 545 replaced with glycine ReplacedResidue cholelithiasis DOID:10211 R-HSA-5678836-ABCB4 mutants [plasma membrane]-DefinedSet-hasMember R-HSA-5678749 Defective ABCB4 does not transport PC from plasma membrane to extracellular region loss_of_function pubmed:17726488|pubmed:11313316|pubmed:10767346|pubmed:9923886|pubmed:18482588|pubmed:19584064|pubmed:9419367 R-HSA-5678771 "Defective ABCB4 causes PFIC3, ICP3 and GBD1" R-HSA-5678706 ABCB4 transports PC from plasma membrane to extracellular region ABC-family proteins mediated transport R-HSA-382556 55085 transmembrane transport missense loss_of_function of transmembrane transport

PS3 ABCB4 ABCB4 A546D [plasma membrane] R-HSA-5678948 ABCB4 UniProt:P21439 L-alanine 546 replaced with L-aspartic acid ReplacedResidue intrahepatic cholestasis DOID:1852 R-HSA-5678836-ABCB4 mutants [plasma membrane]-DefinedSet-hasMember R-HSA-5678749 Defective ABCB4 does not transport PC from plasma membrane to extracellular region loss_of_function pubmed:17726488|pubmed:11313316|pubmed:10767346|pubmed:9923886|pubmed:18482588|pubmed:19584064|pubmed:9419367 R-HSA-5678771 "Defective ABCB4 causes PFIC3, ICP3 and GBD1" R-HSA-5678706 ABCB4 transports PC from plasma membrane to extracellular region ABC-family proteins mediated transport R-HSA-382556 55085 transmembrane transport missense loss_of_function of transmembrane transport

PS3 ABCB4 ABCB4 R144* [plasma membrane] R-HSA-5678839 ABCB4 UniProt:P21439 Nonsense mutation at L-arginine 144 NonsenseMutation intrahepatic cholestasis DOID:1852 R-HSA-5678836-ABCB4 mutants [plasma membrane]-DefinedSet-hasMember R-HSA-5678749 Defective ABCB4 does not transport PC from plasma membrane to extracellular region loss_of_function pubmed:17726488|pubmed:11313316|pubmed:10767346|pubmed:9923886|pubmed:18482588|pubmed:19584064|pubmed:9419367 R-HSA-5678771 "Defective ABCB4 causes PFIC3, ICP3 and GBD1" R-HSA-5678706 ABCB4 transports PC from plasma membrane to extracellular region ABC-family proteins mediated transport R-HSA-382556 55085 transmembrane transport nonsense loss_of_function of transmembrane transport

PS3 ABCB6 ABCB6 A57T [mitochondrial outer membrane] R-HSA-5683344 ABCB6 UniProt:Q9NP58 L-alanine 57 replaced with L-threonine ReplacedResidue microphthalmia DOID:10629 R-HSA-5683361-ABCB6 mutants [mitochondrial outer membrane]-DefinedSet-hasMember R-HSA-5683355 Defective ABCB6 does not transport porphyrin from cytosol into mitochondria matrix loss_of_function pubmed:22226084 R-HSA-5683371 Defective ABCB6 causes MCOPCB7 R-HSA-1369065 ABCB6 transports porphyrin from cytosol to mitchondrial matrix ABC-family proteins mediated transport R-HSA-382556 55085 transmembrane transport missense loss_of_function of transmembrane transport

PS3 ABCB6 ABCB6 L811V [mitochondrial outer membrane] R-HSA-5683347 ABCB6 UniProt:Q9NP58 L-leucine 811 replaced with L-valine ReplacedResidue microphthalmia DOID:10629 R-HSA-5683361-ABCB6 mutants [mitochondrial outer membrane]-DefinedSet-hasMember R-HSA-5683355 Defective ABCB6 does not transport porphyrin from cytosol into mitochondria matrix loss_of_function pubmed:22226084 R-HSA-5683371 Defective ABCB6 causes MCOPCB7 R-HSA-1369065 ABCB6 transports porphyrin from cytosol to mitchondrial matrix ABC-family proteins mediated transport R-HSA-382556 55085 transmembrane transport missense loss_of_function of transmembrane transport

PS3 ABCC2 ABCC2 R768W [plasma membrane] R-HSA-5679033 ABCC2 UniProt:Q92887 L-arginine 768 replaced with L-tryptophan ReplacedResidue Dubin-Johnson syndrome DOID:12308 R-HSA-5679025-ABCC2 mutants [plasma membrane]-DefinedSet-hasMember R-HSA-5679031 "Defective ABCC2 does not transport BMG,BDG from cytosol to extracellular region" loss_of_function pubmed:11477083|pubmed:21044052|pubmed:9425227 R-HSA-5679001 Defective ABCC2 causes DJS R-HSA-5679041 "ABCC2 transports BMG,BDG from cytosol to extracellular region" Metabolism of porphyrins R-HSA-189445 6778 porphyrin-containing compound metabolic process missense loss_of_function of porphyrin-containing compound metabolic process

PS3 ABCC2 ABCC2 R1066* [plasma membrane] R-HSA-5679021 ABCC2 UniProt:Q92887 Nonsense mutation at L-arginine 1066 NonsenseMutation Dubin-Johnson syndrome DOID:12308 R-HSA-5679025-ABCC2 mutants [plasma membrane]-DefinedSet-hasMember R-HSA-5679031 "Defective ABCC2 does not transport BMG,BDG from cytosol to extracellular region" loss_of_function pubmed:11477083|pubmed:21044052|pubmed:9425227 R-HSA-5679001 Defective ABCC2 causes DJS R-HSA-5679041 "ABCC2 transports BMG,BDG from cytosol to extracellular region" Metabolism of porphyrins R-HSA-189445 6778 porphyrin-containing compound metabolic process nonsense loss_of_function of porphyrin-containing compound metabolic process

PS3 ABCC2 ABCC2 R1150H [plasma membrane] R-HSA-5679024 ABCC2 UniProt:Q92887 L-arginine 1150 replaced with L-histidine ReplacedResidue Dubin-Johnson syndrome DOID:12308 R-HSA-5679025-ABCC2 mutants [plasma membrane]-DefinedSet-hasMember R-HSA-5679031 "Defective ABCC2 does not transport BMG,BDG from cytosol to extracellular region" loss_of_function pubmed:11477083|pubmed:21044052|pubmed:9425227 R-HSA-5679001 Defective ABCC2 causes DJS R-HSA-5679041 "ABCC2 transports BMG,BDG from cytosol to extracellular region" Metabolism of porphyrins R-HSA-189445 6778 porphyrin-containing compound metabolic process missense loss_of_function of porphyrin-containing compound metabolic process

PS3 ABCC2 ABCC2 I1173F [plasma membrane] R-HSA-5679030 ABCC2 UniProt:Q92887 L-isoleucine 1173 replaced with L-phenylalanine ReplacedResidue Dubin-Johnson syndrome DOID:12308 R-HSA-5679025-ABCC2 mutants [plasma membrane]-DefinedSet-hasMember R-HSA-5679031 "Defective ABCC2 does not transport BMG,BDG from cytosol to extracellular region" loss_of_function pubmed:11477083|pubmed:21044052|pubmed:9425227 R-HSA-5679001 Defective ABCC2 causes DJS R-HSA-5679041 "ABCC2 transports BMG,BDG from cytosol to extracellular region" Metabolism of porphyrins R-HSA-189445 6778 porphyrin-containing compound metabolic process missense loss_of_function of porphyrin-containing compound metabolic process

PS3 ABCC6 ABCC6 R1141* [plasma membrane] R-HSA-5690367 ABCC6 UniProt:O95255 Nonsense mutation at L-arginine 1141 NonsenseMutation pseudoxanthoma elasticum DOID:2738 R-HSA-5690340 Defective ABCC6 does not transport organic anion from cytosol to extracellular region loss_of_function pubmed:12714611|pubmed:10835642|pubmed:11536079 R-HSA-5690338 Defective ABCC6 causes PXE R-HSA-1454916 The ABCC family mediates organic anion transport ABC-family proteins mediated transport R-HSA-382556 55085 transmembrane transport nonsense loss_of_function of transmembrane transport

PS3 ABCC8 ABCC8 E1507K [plasma membrane] R-HSA-5683146 ABCC8 UniProt:Q09428 L-glutamic acid 1507 replaced with L-lysine ReplacedResidue hyperinsulinemic hypoglycemia DOID:13317 R-HSA-5683203-ABCC8 mutants (HHF1) [plasma membrane]-DefinedSet-hasMember R-HSA-5683113 "Defective ABCC8 does not form functional KATP channels, causing hyperinsulinemic hypoglycemia" loss_of_function pubmed:8923011|pubmed:7847376|pubmed:10334322 R-HSA-5683177 Defective ABCC8 can cause hypo- and hyper-glycemias R-HSA-265682 "KCNJ11 tetramer:ABCC8 tetramer binds 4xATP, closing the channel" Integration of energy metabolism R-HSA-163685 43467 regulation of generation of precursor metabolites and energy missense loss_of_function of regulation of generation of precursor metabolites and energy

PS3 ABCC8 ABCC8 F1388del [plasma membrane] R-HSA-5683137 ABCC8 UniProt:Q09428 Deletion of residues 1388 to 1388 FragmentDeletionModification hyperinsulinemic hypoglycemia DOID:13317 R-HSA-5683203-ABCC8 mutants (HHF1) [plasma membrane]-DefinedSet-hasMember R-HSA-5683113 "Defective ABCC8 does not form functional KATP channels, causing hyperinsulinemic hypoglycemia" loss_of_function pubmed:8923011|pubmed:7847376|pubmed:10334322 R-HSA-5683177 Defective ABCC8 can cause hypo- and hyper-glycemias R-HSA-265682 "KCNJ11 tetramer:ABCC8 tetramer binds 4xATP, closing the channel" Integration of energy metabolism R-HSA-163685 43467 regulation of generation of precursor metabolites and energy in-frame indel: deletion loss_of_function of regulation of generation of precursor metabolites and energy

PS3 ABCC8 ABCC8 A1330Gfs*35 [plasma membrane] R-HSA-5683122 ABCC8 UniProt:Q09428 Replacement of residues 1330 to 1363 FragmentReplacedModification hyperinsulinemic hypoglycemia DOID:13317 R-HSA-5683203-ABCC8 mutants (HHF1) [plasma membrane]-DefinedSet-hasMember R-HSA-5683113 "Defective ABCC8 does not form functional KATP channels, causing hyperinsulinemic hypoglycemia" loss_of_function pubmed:8923011|pubmed:7847376|pubmed:10334322 R-HSA-5683177 Defective ABCC8 can cause hypo- and hyper-glycemias R-HSA-265682 "KCNJ11 tetramer:ABCC8 tetramer binds 4xATP, closing the channel" Integration of energy metabolism R-HSA-163685 43467 regulation of generation of precursor metabolites and energy frameshift loss_of_function of regulation of generation of precursor metabolites and energy

PS3 ABCC8 ABCC8 V187D [plasma membrane] R-HSA-5683140 ABCC8 UniProt:Q09428 L-valine 187 replaced with L-aspartic acid ReplacedResidue hyperinsulinemic hypoglycemia DOID:13317 R-HSA-5683203-ABCC8 mutants (HHF1) [plasma membrane]-DefinedSet-hasMember R-HSA-5683113 "Defective ABCC8 does not form functional KATP channels, causing hyperinsulinemic hypoglycemia" loss_of_function pubmed:8923011|pubmed:7847376|pubmed:10334322 R-HSA-5683177 Defective ABCC8 can cause hypo- and hyper-glycemias R-HSA-265682 "KCNJ11 tetramer:ABCC8 tetramer binds 4xATP, closing the channel" Integration of energy metabolism R-HSA-163685 43467 regulation of generation of precursor metabolites and energy missense loss_of_function of regulation of generation of precursor metabolites and energy

PS3 ABCC8 ABCC8 N72S [plasma membrane] R-HSA-5683257 ABCC8 UniProt:Q09428 L-asparagine 72 replaced with L-serine ReplacedResidue neonatal diabetes mellitus DOID:11717 "R-HSA-5683210-ABCC8 mutants (PNDM, TNDM2) [plasma membrane]-DefinedSet-hasMember" R-HSA-5683209 Activating ABCC8 mutants cause hyperglycemia in permanent neonatal diabetes mellitus (PNDM) and transient neonatal DM (TNDM). gain_of_function pubmed:24843477|pubmed:16613899|pubmed:16885549|pubmed:17668386 R-HSA-5683177 Defective ABCC8 can cause hypo- and hyper-glycemias Integration of energy metabolism R-HSA-163685 43467 regulation of generation of precursor metabolites and energy missense gain_of_function of regulation of generation of precursor metabolites and energy

PS3 ABCC8 ABCC8 E382K [plasma membrane] R-HSA-5683256 ABCC8 UniProt:Q09428 L-glutamic acid 382 replaced with L-lysine ReplacedResidue neonatal diabetes mellitus DOID:11717 "R-HSA-5683210-ABCC8 mutants (PNDM, TNDM2) [plasma membrane]-DefinedSet-hasMember" R-HSA-5683209 Activating ABCC8 mutants cause hyperglycemia in permanent neonatal diabetes mellitus (PNDM) and transient neonatal DM (TNDM). gain_of_function pubmed:24843477|pubmed:16613899|pubmed:16885549|pubmed:17668386 R-HSA-5683177 Defective ABCC8 can cause hypo- and hyper-glycemias Integration of energy metabolism R-HSA-163685 43467 regulation of generation of precursor metabolites and energy missense gain_of_function of regulation of generation of precursor metabolites and energy

PS3 ABCC8 ABCC8 R1379C [plasma membrane] R-HSA-5683307 ABCC8 UniProt:Q09428 L-arginine 1379 replaced with L-cysteine ReplacedResidue neonatal diabetes mellitus DOID:11717 "R-HSA-5683210-ABCC8 mutants (PNDM, TNDM2) [plasma membrane]-DefinedSet-hasMember" R-HSA-5683209 Activating ABCC8 mutants cause hyperglycemia in permanent neonatal diabetes mellitus (PNDM) and transient neonatal DM (TNDM). gain_of_function pubmed:24843477|pubmed:16613899|pubmed:16885549|pubmed:17668386 R-HSA-5683177 Defective ABCC8 can cause hypo- and hyper-glycemias Integration of energy metabolism R-HSA-163685 43467 regulation of generation of precursor metabolites and energy missense gain_of_function of regulation of generation of precursor metabolites and energy

PS3 ABCC8 ABCC8 L582V [plasma membrane] R-HSA-5683311 ABCC8 UniProt:Q09428 L-leucine 582 replaced with L-valine ReplacedResidue neonatal diabetes mellitus DOID:11717 "R-HSA-5683210-ABCC8 mutants (PNDM, TNDM2) [plasma membrane]-DefinedSet-hasMember" R-HSA-5683209 Activating ABCC8 mutants cause hyperglycemia in permanent neonatal diabetes mellitus (PNDM) and transient neonatal DM (TNDM). gain_of_function pubmed:24843477|pubmed:16613899|pubmed:16885549|pubmed:17668386 R-HSA-5683177 Defective ABCC8 can cause hypo- and hyper-glycemias Integration of energy metabolism R-HSA-163685 43467 regulation of generation of precursor metabolites and energy missense gain_of_function of regulation of generation of precursor metabolites and energy

PS3 ABCC8 ABCC8 P132L [plasma membrane] R-HSA-5683247 ABCC8 UniProt:Q09428 L-proline 132 replaced with L-leucine ReplacedResidue neonatal diabetes mellitus DOID:11717 "R-HSA-5683210-ABCC8 mutants (PNDM, TNDM2) [plasma membrane]-DefinedSet-hasMember" R-HSA-5683209 Activating ABCC8 mutants cause hyperglycemia in permanent neonatal diabetes mellitus (PNDM) and transient neonatal DM (TNDM). gain_of_function pubmed:24843477|pubmed:16613899|pubmed:16885549|pubmed:17668386 R-HSA-5683177 Defective ABCC8 can cause hypo- and hyper-glycemias Integration of energy metabolism R-HSA-163685 43467 regulation of generation of precursor metabolites and energy missense gain_of_function of regulation of generation of precursor metabolites and energy

PS3 ABCC8 ABCC8 A1185E [plasma membrane] R-HSA-5683251 ABCC8 UniProt:Q09428 L-alanine 1185 replaced with L-glutamic acid ReplacedResidue neonatal diabetes mellitus DOID:11717 "R-HSA-5683210-ABCC8 mutants (PNDM, TNDM2) [plasma membrane]-DefinedSet-hasMember" R-HSA-5683209 Activating ABCC8 mutants cause hyperglycemia in permanent neonatal diabetes mellitus (PNDM) and transient neonatal DM (TNDM). gain_of_function pubmed:24843477|pubmed:16613899|pubmed:16885549|pubmed:17668386 R-HSA-5683177 Defective ABCC8 can cause hypo- and hyper-glycemias Integration of energy metabolism R-HSA-163685 43467 regulation of generation of precursor metabolites and energy missense gain_of_function of regulation of generation of precursor metabolites and energy

PS3 ABCC8 ABCC8 L213R [plasma membrane] R-HSA-5683253 ABCC8 UniProt:Q09428 L-leucine 213 replaced with L-arginine ReplacedResidue neonatal diabetes mellitus DOID:11717 "R-HSA-5683210-ABCC8 mutants (PNDM, TNDM2) [plasma membrane]-DefinedSet-hasMember" R-HSA-5683209 Activating ABCC8 mutants cause hyperglycemia in permanent neonatal diabetes mellitus (PNDM) and transient neonatal DM (TNDM). gain_of_function pubmed:24843477|pubmed:16613899|pubmed:16885549|pubmed:17668386 R-HSA-5683177 Defective ABCC8 can cause hypo- and hyper-glycemias Integration of energy metabolism R-HSA-163685 43467 regulation of generation of precursor metabolites and energy missense gain_of_function of regulation of generation of precursor metabolites and energy

PS3 ABCC9 ABCC9 T1547I [plasma membrane] R-HSA-5678458 ABCC9 UniProt:O60706 L-threonine 1547 replaced with L-isoleucine ReplacedResidue familial atrial fibrillation DOID:0050650 R-HSA-5678456-ABCC9 mutants [plasma membrane]-DefinedSet-hasMember R-HSA-5678418 Defective ABCC9 (in KCNJ11:ABCC9) does not transport K+ from extracellular region to cytosol loss_of_function pubmed:22610116|pubmed:22608503|pubmed:17245405|pubmed:15034580 R-HSA-5678420 "Defective ABCC9 causes CMD10, ATFB12 and Cantu syndrome" R-HSA-5678261 KCNJ11:ABCC9 transports K+ from extracellular region to cytosol ABC-family proteins mediated transport R-HSA-382556 55085 transmembrane transport missense loss_of_function of transmembrane transport

PS3 ABCC9 ABCC9 R1154W [plasma membrane] R-HSA-5678781 ABCC9 UniProt:O60706 L-arginine 1154 replaced with L-tryptophan ReplacedResidue hypertrichosis|osteochondrodysplasia DOID:420|DOID:2256 R-HSA-5678456-ABCC9 mutants [plasma membrane]-DefinedSet-hasMember R-HSA-5678418 Defective ABCC9 (in KCNJ11:ABCC9) does not transport K+ from extracellular region to cytosol loss_of_function pubmed:22610116|pubmed:22608503|pubmed:17245405|pubmed:15034580 R-HSA-5678420 "Defective ABCC9 causes CMD10, ATFB12 and Cantu syndrome" R-HSA-5678261 KCNJ11:ABCC9 transports K+ from extracellular region to cytosol ABC-family proteins mediated transport R-HSA-382556 55085 transmembrane transport missense loss_of_function of transmembrane transport

PS3 ABCC9 ABCC9 S1020P [plasma membrane] R-HSA-5678912 ABCC9 UniProt:O60706 L-serine 1020 replaced with L-proline ReplacedResidue hypertrichosis|osteochondrodysplasia DOID:420|DOID:2256 R-HSA-5678456-ABCC9 mutants [plasma membrane]-DefinedSet-hasMember R-HSA-5678418 Defective ABCC9 (in KCNJ11:ABCC9) does not transport K+ from extracellular region to cytosol loss_of_function pubmed:22610116|pubmed:22608503|pubmed:17245405|pubmed:15034580 R-HSA-5678420 "Defective ABCC9 causes CMD10, ATFB12 and Cantu syndrome" R-HSA-5678261 KCNJ11:ABCC9 transports K+ from extracellular region to cytosol ABC-family proteins mediated transport R-HSA-382556 55085 transmembrane transport missense loss_of_function of transmembrane transport

PS3 ABCC9 ABCC9 A1513T [plasma membrane] R-HSA-5678947 ABCC9 UniProt:O60706 L-alanine 1513 replaced with L-threonine ReplacedResidue dilated cardiomyopathy DOID:12930 R-HSA-5678456-ABCC9 mutants [plasma membrane]-DefinedSet-hasMember R-HSA-5678418 Defective ABCC9 (in KCNJ11:ABCC9) does not transport K+ from extracellular region to cytosol loss_of_function pubmed:22610116|pubmed:22608503|pubmed:17245405|pubmed:15034580 R-HSA-5678420 "Defective ABCC9 causes CMD10, ATFB12 and Cantu syndrome" R-HSA-5678261 KCNJ11:ABCC9 transports K+ from extracellular region to cytosol ABC-family proteins mediated transport R-HSA-382556 55085 transmembrane transport missense loss_of_function of transmembrane transport

PS3 ABCC9 ABCC9 R1154Q [plasma membrane] R-HSA-5678930 ABCC9 UniProt:O60706 L-arginine 1154 replaced with L-glutamine ReplacedResidue hypertrichosis|osteochondrodysplasia DOID:420|DOID:2256 R-HSA-5678456-ABCC9 mutants [plasma membrane]-DefinedSet-hasMember R-HSA-5678418 Defective ABCC9 (in KCNJ11:ABCC9) does not transport K+ from extracellular region to cytosol loss_of_function pubmed:22610116|pubmed:22608503|pubmed:17245405|pubmed:15034580 R-HSA-5678420 "Defective ABCC9 causes CMD10, ATFB12 and Cantu syndrome" R-HSA-5678261 KCNJ11:ABCC9 transports K+ from extracellular region to cytosol ABC-family proteins mediated transport R-HSA-382556 55085 transmembrane transport missense loss_of_function of transmembrane transport

PS3 ABCC9 ABCC9 H60Y [plasma membrane] R-HSA-5678818 ABCC9 UniProt:O60706 L-histidine 60 replaced with L-tyrosine ReplacedResidue hypertrichosis|osteochondrodysplasia DOID:420|DOID:2256 R-HSA-5678456-ABCC9 mutants [plasma membrane]-DefinedSet-hasMember R-HSA-5678418 Defective ABCC9 (in KCNJ11:ABCC9) does not transport K+ from extracellular region to cytosol loss_of_function pubmed:22610116|pubmed:22608503|pubmed:17245405|pubmed:15034580 R-HSA-5678420 "Defective ABCC9 causes CMD10, ATFB12 and Cantu syndrome" R-HSA-5678261 KCNJ11:ABCC9 transports K+ from extracellular region to cytosol ABC-family proteins mediated transport R-HSA-382556 55085 transmembrane transport missense loss_of_function of transmembrane transport

PS3 ABCC9 ABCC9 C1043Y [plasma membrane] R-HSA-5678850 ABCC9 UniProt:O60706 L-cysteine 1043 replaced with L-tyrosine ReplacedResidue hypertrichosis|osteochondrodysplasia DOID:420|DOID:2256 R-HSA-5678456-ABCC9 mutants [plasma membrane]-DefinedSet-hasMember R-HSA-5678418 Defective ABCC9 (in KCNJ11:ABCC9) does not transport K+ from extracellular region to cytosol loss_of_function pubmed:22610116|pubmed:22608503|pubmed:17245405|pubmed:15034580 R-HSA-5678420 "Defective ABCC9 causes CMD10, ATFB12 and Cantu syndrome" R-HSA-5678261 KCNJ11:ABCC9 transports K+ from extracellular region to cytosol ABC-family proteins mediated transport R-HSA-382556 55085 transmembrane transport missense loss_of_function of transmembrane transport

PS3 ABCC9 ABCC9 L1524Kfs*5 [plasma membrane] R-HSA-5678421 ABCC9 UniProt:O60706 Replacement of residues 1524 to 1527 by KCGV FragmentReplacedModification dilated cardiomyopathy DOID:12930 R-HSA-5678456-ABCC9 mutants [plasma membrane]-DefinedSet-hasMember R-HSA-5678418 Defective ABCC9 (in KCNJ11:ABCC9) does not transport K+ from extracellular region to cytosol loss_of_function pubmed:22610116|pubmed:22608503|pubmed:17245405|pubmed:15034580 R-HSA-5678420 "Defective ABCC9 causes CMD10, ATFB12 and Cantu syndrome" R-HSA-5678261 KCNJ11:ABCC9 transports K+ from extracellular region to cytosol ABC-family proteins mediated transport R-HSA-382556 55085 transmembrane transport frameshift loss_of_function of transmembrane transport

PS3 ABCC9 ABCC9 R1116H [plasma membrane] R-HSA-5678820 ABCC9 UniProt:O60706 L-arginine 1116 replaced with L-histidine ReplacedResidue hypertrichosis|osteochondrodysplasia DOID:420|DOID:2256 R-HSA-5678456-ABCC9 mutants [plasma membrane]-DefinedSet-hasMember R-HSA-5678418 Defective ABCC9 (in KCNJ11:ABCC9) does not transport K+ from extracellular region to cytosol loss_of_function pubmed:22610116|pubmed:22608503|pubmed:17245405|pubmed:15034580 R-HSA-5678420 "Defective ABCC9 causes CMD10, ATFB12 and Cantu syndrome" R-HSA-5678261 KCNJ11:ABCC9 transports K+ from extracellular region to cytosol ABC-family proteins mediated transport R-HSA-382556 55085 transmembrane transport missense loss_of_function of transmembrane transport

PS3 ABCD1 ABCD1 R617C [peroxisomal membrane] R-HSA-5685069 ABCD1 UniProt:P33897 L-arginine 617 replaced with L-cysteine ReplacedResidue adrenoleukodystrophy DOID:10588 R-HSA-5685092-ABCD1 mutants [peroxisomal membrane]-DefinedSet-hasMember R-HSA-5684043 Defective ABCD1 does not transfer LCFAs from cytosol to peroxisomal matrix loss_of_function pubmed:15811009|pubmed:24719134|pubmed:8040304|pubmed:22176151|pubmed:8566952 R-HSA-5684045 Defective ABCD1 causes ALD R-HSA-382575 ABCD1-3 dimers transfer LCFAs from cytosol to peroxisomal matrix ABC-family proteins mediated transport R-HSA-382556 55085 transmembrane transport missense loss_of_function of transmembrane transport

PS3 ABCD1 ABCD1 T77_L82del [peroxisomal membrane] R-HSA-5685088 ABCD1 UniProt:P33897 Deletion of residues 77 to 82 FragmentDeletionModification adrenoleukodystrophy DOID:10588 R-HSA-5685092-ABCD1 mutants [peroxisomal membrane]-DefinedSet-hasMember R-HSA-5684043 Defective ABCD1 does not transfer LCFAs from cytosol to peroxisomal matrix loss_of_function pubmed:15811009|pubmed:24719134|pubmed:8040304|pubmed:22176151|pubmed:8566952 R-HSA-5684045 Defective ABCD1 causes ALD R-HSA-382575 ABCD1-3 dimers transfer LCFAs from cytosol to peroxisomal matrix ABC-family proteins mediated transport R-HSA-382556 55085 transmembrane transport in-frame indel: deletion loss_of_function of transmembrane transport

PS3 ABCD1 ABCD1 S606L [peroxisomal membrane] R-HSA-5685104 ABCD1 UniProt:P33897 L-serine 606 replaced with L-leucine ReplacedResidue adrenoleukodystrophy DOID:10588 R-HSA-5685092-ABCD1 mutants [peroxisomal membrane]-DefinedSet-hasMember R-HSA-5684043 Defective ABCD1 does not transfer LCFAs from cytosol to peroxisomal matrix loss_of_function pubmed:15811009|pubmed:24719134|pubmed:8040304|pubmed:22176151|pubmed:8566952 R-HSA-5684045 Defective ABCD1 causes ALD R-HSA-382575 ABCD1-3 dimers transfer LCFAs from cytosol to peroxisomal matrix ABC-family proteins mediated transport R-HSA-382556 55085 transmembrane transport missense loss_of_function of transmembrane transport

PS3 ABCD1 ABCD1 R554H [peroxisomal membrane] R-HSA-5685072 ABCD1 UniProt:P33897 L-arginine 554 replaced with L-histidine ReplacedResidue adrenoleukodystrophy DOID:10588 R-HSA-5685092-ABCD1 mutants [peroxisomal membrane]-DefinedSet-hasMember R-HSA-5684043 Defective ABCD1 does not transfer LCFAs from cytosol to peroxisomal matrix loss_of_function pubmed:15811009|pubmed:24719134|pubmed:8040304|pubmed:22176151|pubmed:8566952 R-HSA-5684045 Defective ABCD1 causes ALD R-HSA-382575 ABCD1-3 dimers transfer LCFAs from cytosol to peroxisomal matrix ABC-family proteins mediated transport R-HSA-382556 55085 transmembrane transport missense loss_of_function of transmembrane transport

PS3 ABCD1 ABCD1 M1V [peroxisomal membrane] R-HSA-5685059 ABCD1 UniProt:P33897 L-methionine 1 replaced with L-valine ReplacedResidue adrenoleukodystrophy DOID:10588 R-HSA-5685092-ABCD1 mutants [peroxisomal membrane]-DefinedSet-hasMember R-HSA-5684043 Defective ABCD1 does not transfer LCFAs from cytosol to peroxisomal matrix loss_of_function pubmed:15811009|pubmed:24719134|pubmed:8040304|pubmed:22176151|pubmed:8566952 R-HSA-5684045 Defective ABCD1 causes ALD R-HSA-382575 ABCD1-3 dimers transfer LCFAs from cytosol to peroxisomal matrix ABC-family proteins mediated transport R-HSA-382556 55085 transmembrane transport missense loss_of_function of transmembrane transport

PS3 ABCD1 ABCD1 G277R [peroxisomal membrane] R-HSA-5685058 ABCD1 UniProt:P33897 glycine 277 replaced with L-arginine ReplacedResidue adrenoleukodystrophy DOID:10588 R-HSA-5685092-ABCD1 mutants [peroxisomal membrane]-DefinedSet-hasMember R-HSA-5684043 Defective ABCD1 does not transfer LCFAs from cytosol to peroxisomal matrix loss_of_function pubmed:15811009|pubmed:24719134|pubmed:8040304|pubmed:22176151|pubmed:8566952 R-HSA-5684045 Defective ABCD1 causes ALD R-HSA-382575 ABCD1-3 dimers transfer LCFAs from cytosol to peroxisomal matrix ABC-family proteins mediated transport R-HSA-382556 55085 transmembrane transport missense loss_of_function of transmembrane transport

PS3 ABCD4 ABCD4 E583Lfs*9 [lysosomal membrane] R-HSA-5683320 ABCD4 UniProt:O14678 Replacement of residues 583 to 590 by LRSFIPWF FragmentReplacedModification methylmalonic acidemia|homocystinuria DOID:14749|DOID:9263 R-HSA-5683323-ABCD4 mutant:LBRD1 complexes [lysosomal membrane]-DefinedSet-hasMember R-HSA-5683325 Defective ABCD4:LMBRD1 does not transport Cbl from lysosomal lumen to cytosol loss_of_function pubmed:22922874 R-HSA-5683329 Defective ABCD4 causes MAHCJ R-HSA-5223313 ABCD4:LMBRD1 transports RCbl from lysosomal lumen to cytosol (gut mucosal cells) Metabolism of water-soluble vitamins and cofactors R-HSA-196849 6767 water-soluble vitamin metabolic process frameshift loss_of_function of water-soluble vitamin metabolic process

PS3 ABCD4 ABCD4 Y319C [lysosomal membrane] R-HSA-5683326 ABCD4 UniProt:O14678 L-tyrosine 319 replaced with L-cysteine ReplacedResidue methylmalonic acidemia|homocystinuria DOID:14749|DOID:9263 R-HSA-5683323-ABCD4 mutant:LBRD1 complexes [lysosomal membrane]-DefinedSet-hasMember R-HSA-5683325 Defective ABCD4:LMBRD1 does not transport Cbl from lysosomal lumen to cytosol loss_of_function pubmed:22922874 R-HSA-5683329 Defective ABCD4 causes MAHCJ R-HSA-5223313 ABCD4:LMBRD1 transports RCbl from lysosomal lumen to cytosol (gut mucosal cells) Metabolism of water-soluble vitamins and cofactors R-HSA-196849 6767 water-soluble vitamin metabolic process missense loss_of_function of water-soluble vitamin metabolic process

PS3 ABCG5 ABCG5 R389H [plasma membrane] R-HSA-5679176 ABCG5 UniProt:Q9H222 L-arginine 389 replaced with L-histidine ReplacedResidue lipid metabolism disorder DOID:3146 R-HSA-5679155-ABCG5 mutants [plasma membrane]-DefinedSet-hasMember R-HSA-5679145 Defective ABCG5 (in ABCG5:ABCG8) does not transport sterols from cytosol to extracellular region loss_of_function pubmed:11099417|pubmed:17976197|pubmed:11138003|pubmed:20719861 R-HSA-5679096 Defective ABCG5 causes sitosterolemia R-HSA-265783 ABCG5:ABCG8 transports sterols from cytosol to extracellular region ABC-family proteins mediated transport R-HSA-382556 55085 transmembrane transport missense loss_of_function of transmembrane transport

PS3 ABCG5 ABCG5 R408* [plasma membrane] R-HSA-5679117 ABCG5 UniProt:Q9H222 Nonsense mutation at L-arginine 408 NonsenseMutation lipid metabolism disorder DOID:3146 R-HSA-5679155-ABCG5 mutants [plasma membrane]-DefinedSet-hasMember R-HSA-5679145 Defective ABCG5 (in ABCG5:ABCG8) does not transport sterols from cytosol to extracellular region loss_of_function pubmed:11099417|pubmed:17976197|pubmed:11138003|pubmed:20719861 R-HSA-5679096 Defective ABCG5 causes sitosterolemia R-HSA-265783 ABCG5:ABCG8 transports sterols from cytosol to extracellular region ABC-family proteins mediated transport R-HSA-382556 55085 transmembrane transport nonsense loss_of_function of transmembrane transport

PS3 ABCG5 ABCG5 Q16* [plasma membrane] R-HSA-5679132 ABCG5 UniProt:Q9H222 Nonsense mutation at L-glutamine 16 NonsenseMutation lipid metabolism disorder DOID:3146 R-HSA-5679155-ABCG5 mutants [plasma membrane]-DefinedSet-hasMember R-HSA-5679145 Defective ABCG5 (in ABCG5:ABCG8) does not transport sterols from cytosol to extracellular region loss_of_function pubmed:11099417|pubmed:17976197|pubmed:11138003|pubmed:20719861 R-HSA-5679096 Defective ABCG5 causes sitosterolemia R-HSA-265783 ABCG5:ABCG8 transports sterols from cytosol to extracellular region ABC-family proteins mediated transport R-HSA-382556 55085 transmembrane transport nonsense loss_of_function of transmembrane transport

PS3 ABCG5 ABCG5 R419H [plasma membrane] R-HSA-5679073 ABCG5 UniProt:Q9H222 L-arginine 419 replaced with L-histidine ReplacedResidue lipid metabolism disorder DOID:3146 R-HSA-5679155-ABCG5 mutants [plasma membrane]-DefinedSet-hasMember R-HSA-5679145 Defective ABCG5 (in ABCG5:ABCG8) does not transport sterols from cytosol to extracellular region loss_of_function pubmed:11099417|pubmed:17976197|pubmed:11138003|pubmed:20719861 R-HSA-5679096 Defective ABCG5 causes sitosterolemia R-HSA-265783 ABCG5:ABCG8 transports sterols from cytosol to extracellular region ABC-family proteins mediated transport R-HSA-382556 55085 transmembrane transport missense loss_of_function of transmembrane transport

PS3 ABCG5 ABCG5 R243* [plasma membrane] R-HSA-5679089 ABCG5 UniProt:Q9H222 Nonsense mutation at L-arginine 243 NonsenseMutation lipid metabolism disorder DOID:3146 R-HSA-5679155-ABCG5 mutants [plasma membrane]-DefinedSet-hasMember R-HSA-5679145 Defective ABCG5 (in ABCG5:ABCG8) does not transport sterols from cytosol to extracellular region loss_of_function pubmed:11099417|pubmed:17976197|pubmed:11138003|pubmed:20719861 R-HSA-5679096 Defective ABCG5 causes sitosterolemia R-HSA-265783 ABCG5:ABCG8 transports sterols from cytosol to extracellular region ABC-family proteins mediated transport R-HSA-382556 55085 transmembrane transport nonsense loss_of_function of transmembrane transport

PS3 ABCG5 ABCG5 R446* [plasma membrane] R-HSA-5679142 ABCG5 UniProt:Q9H222 Nonsense mutation at L-arginine 446 NonsenseMutation lipid metabolism disorder DOID:3146 R-HSA-5679155-ABCG5 mutants [plasma membrane]-DefinedSet-hasMember R-HSA-5679145 Defective ABCG5 (in ABCG5:ABCG8) does not transport sterols from cytosol to extracellular region loss_of_function pubmed:11099417|pubmed:17976197|pubmed:11138003|pubmed:20719861 R-HSA-5679096 Defective ABCG5 causes sitosterolemia R-HSA-265783 ABCG5:ABCG8 transports sterols from cytosol to extracellular region ABC-family proteins mediated transport R-HSA-382556 55085 transmembrane transport nonsense loss_of_function of transmembrane transport

PS3 ABCG8 ABCG8 Y658* [plasma membrane] R-HSA-5679080 ABCG8 UniProt:Q9H221 Nonsense mutation at L-tyrosine 658 NonsenseMutation lipid metabolism disorder DOID:3146 R-HSA-5679186-ABCG8 mutants [plasma membrane]-DefinedSet-hasMember R-HSA-5679101 Defective ABCG8 (in ABCG5:ABCG8) does not transport sterols from cytosol to extracellular region loss_of_function pubmed:11099417|pubmed:17632509 R-HSA-5679090 Defective ABCG8 causes GBD4 and sitosterolemia R-HSA-265783 ABCG5:ABCG8 transports sterols from cytosol to extracellular region ABC-family proteins mediated transport R-HSA-382556 55085 transmembrane transport nonsense loss_of_function of transmembrane transport

PS3 ABCG8 ABCG8 G574R [plasma membrane] R-HSA-5679144 ABCG8 UniProt:Q9H221 glycine 574 replaced with L-arginine ReplacedResidue lipid metabolism disorder DOID:3146 R-HSA-5679186-ABCG8 mutants [plasma membrane]-DefinedSet-hasMember R-HSA-5679101 Defective ABCG8 (in ABCG5:ABCG8) does not transport sterols from cytosol to extracellular region loss_of_function pubmed:11099417|pubmed:17632509 R-HSA-5679090 Defective ABCG8 causes GBD4 and sitosterolemia R-HSA-265783 ABCG5:ABCG8 transports sterols from cytosol to extracellular region ABC-family proteins mediated transport R-HSA-382556 55085 transmembrane transport missense loss_of_function of transmembrane transport

PS3 ABCG8 ABCG8 P231T [plasma membrane] R-HSA-5679100 ABCG8 UniProt:Q9H221 L-proline 231 replaced with L-threonine ReplacedResidue lipid metabolism disorder DOID:3146 R-HSA-5679186-ABCG8 mutants [plasma membrane]-DefinedSet-hasMember R-HSA-5679101 Defective ABCG8 (in ABCG5:ABCG8) does not transport sterols from cytosol to extracellular region loss_of_function pubmed:11099417|pubmed:17632509 R-HSA-5679090 Defective ABCG8 causes GBD4 and sitosterolemia R-HSA-265783 ABCG5:ABCG8 transports sterols from cytosol to extracellular region ABC-family proteins mediated transport R-HSA-382556 55085 transmembrane transport missense loss_of_function of transmembrane transport

PS3 ABCG8 ABCG8 W361* [plasma membrane] R-HSA-5679166 ABCG8 UniProt:Q9H221 Nonsense mutation at L-tryptophan 361 NonsenseMutation lipid metabolism disorder DOID:3146 R-HSA-5679186-ABCG8 mutants [plasma membrane]-DefinedSet-hasMember R-HSA-5679101 Defective ABCG8 (in ABCG5:ABCG8) does not transport sterols from cytosol to extracellular region loss_of_function pubmed:11099417|pubmed:17632509 R-HSA-5679090 Defective ABCG8 causes GBD4 and sitosterolemia R-HSA-265783 ABCG5:ABCG8 transports sterols from cytosol to extracellular region ABC-family proteins mediated transport R-HSA-382556 55085 transmembrane transport nonsense loss_of_function of transmembrane transport

PS3 ABCG8 ABCG8 R263Q [plasma membrane] R-HSA-5679185 ABCG8 UniProt:Q9H221 L-arginine 263 replaced with L-glutamine ReplacedResidue lipid metabolism disorder DOID:3146 R-HSA-5679186-ABCG8 mutants [plasma membrane]-DefinedSet-hasMember R-HSA-5679101 Defective ABCG8 (in ABCG5:ABCG8) does not transport sterols from cytosol to extracellular region loss_of_function pubmed:11099417|pubmed:17632509 R-HSA-5679090 Defective ABCG8 causes GBD4 and sitosterolemia R-HSA-265783 ABCG5:ABCG8 transports sterols from cytosol to extracellular region ABC-family proteins mediated transport R-HSA-382556 55085 transmembrane transport missense loss_of_function of transmembrane transport

PS3 ABCG8 ABCG8 D19H [plasma membrane] R-HSA-5679104 ABCG8 UniProt:Q9H221 L-aspartic acid 19 replaced with L-histidine ReplacedResidue cholelithiasis DOID:10211 R-HSA-5679186-ABCG8 mutants [plasma membrane]-DefinedSet-hasMember R-HSA-5679101 Defective ABCG8 (in ABCG5:ABCG8) does not transport sterols from cytosol to extracellular region loss_of_function pubmed:11099417|pubmed:17632509 R-HSA-5679090 Defective ABCG8 causes GBD4 and sitosterolemia R-HSA-265783 ABCG5:ABCG8 transports sterols from cytosol to extracellular region ABC-family proteins mediated transport R-HSA-382556 55085 transmembrane transport missense loss_of_function of transmembrane transport

PS3 ACY1 ACY1 R393H [cytosol] R-HSA-5579106 ACY1 UniProt:Q03154 L-arginine 393 replaced with L-histidine ReplacedResidue toxic encephalopathy DOID:3602 R-HSA-5579044-ACY1 mutants [cytosol]-DefinedSet-hasMember R-HSA-5579081 Defective ACY1 does not hydrolyse mercapturic acids loss_of_function pubmed:16465618|pubmed:17562838|pubmed:12933810 R-HSA-5579007 Defective ACY1 causes encephalopathy R-HSA-5433074 ACY1:Zn2+ dimer hydrolyses mercapturic acids Aflatoxin activation and detoxification R-HSA-5423646 46222 aflatoxin metabolic process missense loss_of_function of aflatoxin metabolic process

PS3 ACY1 ACY1 E233D [cytosol] R-HSA-5579040 ACY1 UniProt:Q03154 L-glutamic acid 233 replaced with L-aspartic acid ReplacedResidue toxic encephalopathy DOID:3602 R-HSA-5579044-ACY1 mutants [cytosol]-DefinedSet-hasMember R-HSA-5579081 Defective ACY1 does not hydrolyse mercapturic acids loss_of_function pubmed:16465618|pubmed:17562838|pubmed:12933810 R-HSA-5579007 Defective ACY1 causes encephalopathy R-HSA-5433074 ACY1:Zn2+ dimer hydrolyses mercapturic acids Aflatoxin activation and detoxification R-HSA-5423646 46222 aflatoxin metabolic process missense loss_of_function of aflatoxin metabolic process

PS3 ACY1 ACY1 R197W [cytosol] R-HSA-5579041 ACY1 UniProt:Q03154 L-arginine 197 replaced with L-tryptophan ReplacedResidue toxic encephalopathy DOID:3602 R-HSA-5579044-ACY1 mutants [cytosol]-DefinedSet-hasMember R-HSA-5579081 Defective ACY1 does not hydrolyse mercapturic acids loss_of_function pubmed:16465618|pubmed:17562838|pubmed:12933810 R-HSA-5579007 Defective ACY1 causes encephalopathy R-HSA-5433074 ACY1:Zn2+ dimer hydrolyses mercapturic acids Aflatoxin activation and detoxification R-HSA-5423646 46222 aflatoxin metabolic process missense loss_of_function of aflatoxin metabolic process

PS3 ACY1 ACY1 P369Hfs*47 [cytosol] R-HSA-5579105 ACY1 UniProt:Q03154 Replacement of residues 369 to 414 by HLCCCTTTMNGCMRLCSSVGWTYIHACCLPLPVCLPCPVTAEPWNS FragmentReplacedModification toxic encephalopathy DOID:3602 R-HSA-5579044-ACY1 mutants [cytosol]-DefinedSet-hasMember R-HSA-5579081 Defective ACY1 does not hydrolyse mercapturic acids loss_of_function pubmed:16465618|pubmed:17562838|pubmed:12933810 R-HSA-5579007 Defective ACY1 causes encephalopathy R-HSA-5433074 ACY1:Zn2+ dimer hydrolyses mercapturic acids Aflatoxin activation and detoxification R-HSA-5423646 46222 aflatoxin metabolic process frameshift loss_of_function of aflatoxin metabolic process

PS3 ACY1 ACY1 R353C [cytosol] R-HSA-5579111 ACY1 UniProt:Q03154 L-arginine 353 replaced with L-cysteine ReplacedResidue toxic encephalopathy DOID:3602 R-HSA-5579044-ACY1 mutants [cytosol]-DefinedSet-hasMember R-HSA-5579081 Defective ACY1 does not hydrolyse mercapturic acids loss_of_function pubmed:16465618|pubmed:17562838|pubmed:12933810 R-HSA-5579007 Defective ACY1 causes encephalopathy R-HSA-5433074 ACY1:Zn2+ dimer hydrolyses mercapturic acids Aflatoxin activation and detoxification R-HSA-5423646 46222 aflatoxin metabolic process missense loss_of_function of aflatoxin metabolic process

PM5 AGGF1 AGGF(1-290)-p-RAF1(279-648) fusion [cytosol] R-HSA-6802507 AGGF1 UniProt:Q8N302 Insertion of residues 279 to 648 at 291 from UniProt:P04049 RAF1 FragmentInsertionModification prostate adenocarcinoma|thyroid carcinoma|cancer DOID:2526|DOID:3963|DOID:162 pubmed:25204415 R-HSA-6802698-p-BRAF/RAF fusion dimers [cytosol]-CandidateSet-hasCandidate R-HSA-6802935|R-HSA-6802933|R-HSA-6802934|R-HSA-6802927|R-HSA-6802932 MAPKs are phosphorylated downstream of BRAF and RAF fusion dimers|p-BRAF and RAF fusion dimers phosphorylate MAP2Ks|p-BRAF and RAF fusion dimers bind MAP2Ks and MAPKs|BRAF and RAF fusion mutant dimers are phosphorylated|Dissociation of BRAF/RAF fusion complex gain_of_function pubmed:24345920|pubmed:15630448|pubmed:25907612|pubmed:21424530|pubmed:20526349|pubmed:18974108|pubmed:24135138|pubmed:25204415|pubmed:22745804|pubmed:23583981 R-HSA-6802952 Signaling by BRAF and RAF1 fusions R-HSA-5672973|R-HSA-5672978|R-HSA-5672972|R-HSA-5672969|R-HSA-5672980 MAP2Ks phosphorylate MAPKs|RAF phosphorylates MAP2K dimer|MAP2Ks and MAPKs bind to the activated RAF complex|Phosphorylation of RAF|Dissociation of RAS:RAF complex RAF/MAP kinase cascade R-HSA-5673001 165 MAPK cascade fusion gain_of_function of MAPK cascade

PM5 AGGF1 AGGF(1-290)-RAF1(279-648) fusion [cytosol] R-HSA-6802255 AGGF1 UniProt:Q8N302 Insertion of residues 279 to 648 at 291 from UniProt:P04049 RAF1 FragmentInsertionModification prostate adenocarcinoma|thyroid carcinoma|cancer DOID:2526|DOID:3963|DOID:162 pubmed:25204415 R-HSA-6802720-BRAF/RAF fusion dimers [cytosol]-CandidateSet-hasCandidate R-HSA-6802927 BRAF and RAF fusion mutant dimers are phosphorylated gain_of_function pubmed:24345920|pubmed:15630448|pubmed:25907612|pubmed:21424530|pubmed:20526349|pubmed:18974108|pubmed:24135138|pubmed:25204415|pubmed:22745804|pubmed:23583981 R-HSA-6802952 Signaling by BRAF and RAF1 fusions R-HSA-5672969 Phosphorylation of RAF RAF/MAP kinase cascade R-HSA-5673001 165 MAPK cascade fusion gain_of_function of MAPK cascade

PS3 AGK AGK(1-33)InsW-p-BRAF(328-766) fusion [cytosol] R-HSA-6802510 AGK UniProt:Q53H12 Replacement of residues 33 to 33 by HW|Insertion of residues 328 to 766 at 34 from UniProt:P15056 BRAF FragmentReplacedModification|FragmentInsertionModification thyroid cancer DOID:1781 pubmed:24135138 R-HSA-6802698-p-BRAF/RAF fusion dimers [cytosol]-CandidateSet-hasMember R-HSA-6802935|R-HSA-6802933|R-HSA-6802934|R-HSA-6802927|R-HSA-6802932 MAPKs are phosphorylated downstream of BRAF and RAF fusion dimers|p-BRAF and RAF fusion dimers phosphorylate MAP2Ks|p-BRAF and RAF fusion dimers bind MAP2Ks and MAPKs|BRAF and RAF fusion mutant dimers are phosphorylated|Dissociation of BRAF/RAF fusion complex gain_of_function pubmed:24345920|pubmed:15630448|pubmed:25907612|pubmed:21424530|pubmed:20526349|pubmed:18974108|pubmed:24135138|pubmed:25204415|pubmed:22745804|pubmed:23583981 R-HSA-6802952 Signaling by BRAF and RAF1 fusions R-HSA-5672973|R-HSA-5672978|R-HSA-5672972|R-HSA-5672969|R-HSA-5672980 MAP2Ks phosphorylate MAPKs|RAF phosphorylates MAP2K dimer|MAP2Ks and MAPKs bind to the activated RAF complex|Phosphorylation of RAF|Dissociation of RAS:RAF complex RAF/MAP kinase cascade R-HSA-5673001 165 MAPK cascade fusion gain_of_function of MAPK cascade

PS3 AGK AGK(1-33)InsW-BRAF(328-766) fusion [cytosol] R-HSA-6802260 AGK UniProt:Q53H12 Replacement of residues 33 to 33 by HW|Insertion of residues 328 to 766 at 34 from UniProt:P15056 BRAF FragmentReplacedModification|FragmentInsertionModification thyroid cancer DOID:1781 pubmed:24135138 R-HSA-6802720-BRAF/RAF fusion dimers [cytosol]-CandidateSet-hasMember R-HSA-6802927 BRAF and RAF fusion mutant dimers are phosphorylated gain_of_function pubmed:24345920|pubmed:15630448|pubmed:25907612|pubmed:21424530|pubmed:20526349|pubmed:18974108|pubmed:24135138|pubmed:25204415|pubmed:22745804|pubmed:23583981 R-HSA-6802952 Signaling by BRAF and RAF1 fusions R-HSA-5672969 Phosphorylation of RAF RAF/MAP kinase cascade R-HSA-5673001 165 MAPK cascade fusion gain_of_function of MAPK cascade

PM5 AGTRAP AGTRAP(1-159)-p-BRAF(288-545) fusion [cytosol] R-HSA-6802511 AGTRAP UniProt:Q6RW13 Insertion of residues 288 to 545 at 160 from UniProt:P15056 BRAF FragmentInsertionModification gastric adenocarcinoma|cancer DOID:3717|DOID:162 pubmed:20526349 R-HSA-6802698-p-BRAF/RAF fusion dimers [cytosol]-CandidateSet-hasCandidate R-HSA-6802935|R-HSA-6802933|R-HSA-6802934|R-HSA-6802927|R-HSA-6802932 MAPKs are phosphorylated downstream of BRAF and RAF fusion dimers|p-BRAF and RAF fusion dimers phosphorylate MAP2Ks|p-BRAF and RAF fusion dimers bind MAP2Ks and MAPKs|BRAF and RAF fusion mutant dimers are phosphorylated|Dissociation of BRAF/RAF fusion complex gain_of_function pubmed:24345920|pubmed:15630448|pubmed:25907612|pubmed:21424530|pubmed:20526349|pubmed:18974108|pubmed:24135138|pubmed:25204415|pubmed:22745804|pubmed:23583981 R-HSA-6802952 Signaling by BRAF and RAF1 fusions R-HSA-5672973|R-HSA-5672978|R-HSA-5672972|R-HSA-5672969|R-HSA-5672980 MAP2Ks phosphorylate MAPKs|RAF phosphorylates MAP2K dimer|MAP2Ks and MAPKs bind to the activated RAF complex|Phosphorylation of RAF|Dissociation of RAS:RAF complex RAF/MAP kinase cascade R-HSA-5673001 165 MAPK cascade fusion gain_of_function of MAPK cascade

PM5 AGTRAP AGTRAP(1-159)-BRAF(288-545) fusion [cytosol] R-HSA-6802262 AGTRAP UniProt:Q6RW13 Insertion of residues 288 to 545 at 160 from UniProt:P15056 BRAF FragmentInsertionModification gastric adenocarcinoma|cancer DOID:3717|DOID:162 pubmed:20526349 R-HSA-6802720-BRAF/RAF fusion dimers [cytosol]-CandidateSet-hasCandidate R-HSA-6802927 BRAF and RAF fusion mutant dimers are phosphorylated gain_of_function pubmed:24345920|pubmed:15630448|pubmed:25907612|pubmed:21424530|pubmed:20526349|pubmed:18974108|pubmed:24135138|pubmed:25204415|pubmed:22745804|pubmed:23583981 R-HSA-6802952 Signaling by BRAF and RAF1 fusions R-HSA-5672969 Phosphorylation of RAF RAF/MAP kinase cascade R-HSA-5673001 165 MAPK cascade fusion gain_of_function of MAPK cascade

PS3 AHCY AHCY Y143C [cytosol] R-HSA-5579098 AHCY UniProt:P23526 L-tyrosine 143 replaced with L-cysteine ReplacedResidue hypermethioninemia DOID:0050544 R-HSA-5579035-AHCY mutants [cytosol]-DefinedSet-hasMember R-HSA-5579084 Defective AHCY does not hydrolyse AdoHcy loss_of_function pubmed:12590576|pubmed:9586999|pubmed:15024124 R-HSA-5578997 Defective AHCY causes HMAHCHD R-HSA-174401 AHCY:NAD+ tetramer hydrolyses AdoHcy Phase II - Conjugation of compounds R-HSA-156580 6805 xenobiotic metabolic process missense loss_of_function of xenobiotic metabolic process

PS3 AHCY AHCY W112* [cytosol] R-HSA-5579042 AHCY UniProt:P23526 Nonsense mutation at L-tryptophan 112 NonsenseMutation hypermethioninemia DOID:0050544 R-HSA-5579035-AHCY mutants [cytosol]-DefinedSet-hasMember R-HSA-5579084 Defective AHCY does not hydrolyse AdoHcy loss_of_function pubmed:12590576|pubmed:9586999|pubmed:15024124 R-HSA-5578997 Defective AHCY causes HMAHCHD R-HSA-174401 AHCY:NAD+ tetramer hydrolyses AdoHcy Phase II - Conjugation of compounds R-HSA-156580 6805 xenobiotic metabolic process nonsense loss_of_function of xenobiotic metabolic process

PS3 AKAP9 AKAP9(1-1118)-p-BRAF(381-766) fusion [cytosol] R-HSA-6802514 AKAP9 UniProt:Q99996 Insertion of residues 381 to 766 at 1119 from UniProt:P15056 BRAF FragmentInsertionModification thyroid cancer|cancer DOID:1781|DOID:162 pubmed:15630448 R-HSA-6802698-p-BRAF/RAF fusion dimers [cytosol]-CandidateSet-hasMember R-HSA-6802935|R-HSA-6802933|R-HSA-6802934|R-HSA-6802927|R-HSA-6802932 MAPKs are phosphorylated downstream of BRAF and RAF fusion dimers|p-BRAF and RAF fusion dimers phosphorylate MAP2Ks|p-BRAF and RAF fusion dimers bind MAP2Ks and MAPKs|BRAF and RAF fusion mutant dimers are phosphorylated|Dissociation of BRAF/RAF fusion complex gain_of_function pubmed:24345920|pubmed:15630448|pubmed:25907612|pubmed:21424530|pubmed:20526349|pubmed:18974108|pubmed:24135138|pubmed:25204415|pubmed:22745804|pubmed:23583981 R-HSA-6802952 Signaling by BRAF and RAF1 fusions R-HSA-5672973|R-HSA-5672978|R-HSA-5672972|R-HSA-5672969|R-HSA-5672980 MAP2Ks phosphorylate MAPKs|RAF phosphorylates MAP2K dimer|MAP2Ks and MAPKs bind to the activated RAF complex|Phosphorylation of RAF|Dissociation of RAS:RAF complex RAF/MAP kinase cascade R-HSA-5673001 165 MAPK cascade fusion gain_of_function of MAPK cascade

PS3 AKAP9 AKAP9(1-1118)-BRAF(381-766) fusion [cytosol] R-HSA-6802267 AKAP9 UniProt:Q99996 Insertion of residues 381 to 766 at 1119 from UniProt:P15056 BRAF FragmentInsertionModification thyroid cancer|cancer DOID:1781|DOID:162 pubmed:15630448 R-HSA-6802720-BRAF/RAF fusion dimers [cytosol]-CandidateSet-hasMember R-HSA-6802927 BRAF and RAF fusion mutant dimers are phosphorylated gain_of_function pubmed:24345920|pubmed:15630448|pubmed:25907612|pubmed:21424530|pubmed:20526349|pubmed:18974108|pubmed:24135138|pubmed:25204415|pubmed:22745804|pubmed:23583981 R-HSA-6802952 Signaling by BRAF and RAF1 fusions R-HSA-5672969 Phosphorylation of RAF RAF/MAP kinase cascade R-HSA-5673001 165 MAPK cascade fusion gain_of_function of MAPK cascade

PS3-PS1 AKT1 p-S473-AKT1 E17K [plasma membrane] R-HSA-2243944 AKT1 UniProt:P31749 L-glutamic acid 17 replaced with L-lysine ReplacedResidue COSMIC:COSM34142|COSMIC:COSV62571334 ovarian cancer|cancer|breast cancer|large intestine cancer DOID:2394|DOID:162|DOID:1612|DOID:5672 pubmed:17611497 R-HSA-2243938|R-HSA-2243942|R-HSA-2243937 AKT1 E17K mutant is phosphorylated by TORC2 complex|PDPK1 phosphorylates AKT1 E17K mutant|PIP2-bound p-S473-AKT1 mutant binds PIP2-bound PDPK1 gain_of_function pubmed:18954143|pubmed:17611497|pubmed:9895304 R-HSA-5674400 Constitutive Signaling by AKT1 E17K in Cancer PIP3 activates AKT signaling R-HSA-1257604 51897 positive regulation of protein kinase B signaling missense gain_of_function of positive regulation of protein kinase B signaling

PS3-PS1 AKT1 AKT1 E17K [plasma membrane] R-HSA-1500573 AKT1 UniProt:P31749 L-glutamic acid 17 replaced with L-lysine ReplacedResidue COSMIC:COSM34142|COSMIC:COSV62571334 ovarian cancer|cancer|breast cancer|large intestine cancer DOID:2394|DOID:162|DOID:1612|DOID:5672 pubmed:17611497 R-HSA-2243938|R-HSA-2219536 AKT1 E17K mutant is phosphorylated by TORC2 complex|AKT1 E17K mutant binds PIP2 gain_of_function pubmed:18954143|pubmed:17611497 R-HSA-5674400 Constitutive Signaling by AKT1 E17K in Cancer PIP3 activates AKT signaling R-HSA-1257604 51897 positive regulation of protein kinase B signaling missense gain_of_function of positive regulation of protein kinase B signaling

PS3-PS1 AKT1 AKT1 E17K [cytosol] R-HSA-2219526 AKT1 UniProt:P31749 L-glutamic acid 17 replaced with L-lysine ReplacedResidue COSMIC:COSM34142|COSMIC:COSV62571334 ovarian cancer|cancer|breast cancer|large intestine cancer DOID:2394|DOID:162|DOID:1612|DOID:5672 pubmed:17611497 |R-HSA-2400013-AKT/AKT1 E17K mutant [cytosol]-CandidateSet-hasCandidate R-HSA-2219536|R-HSA-2400010 AKT1 E17K mutant binds PIP2|AKT inhibitors block AKT membrane recruitment gain_of_function pubmed:18954143|pubmed:17611497|pubmed:19644473|pubmed:14617782|pubmed:22025163|pubmed:20489726 R-HSA-5674400 Constitutive Signaling by AKT1 E17K in Cancer PIP3 activates AKT signaling R-HSA-1257604 51897 positive regulation of protein kinase B signaling missense gain_of_function of positive regulation of protein kinase B signaling

PS3-PS1 AKT1 "p-T308,S473-AKT1 E17K [cytosol]" R-HSA-2243935 AKT1 UniProt:P31749 L-glutamic acid 17 replaced with L-lysine ReplacedResidue COSMIC:COSM34142|COSMIC:COSV62571334 ovarian cancer|cancer|breast cancer|large intestine cancer DOID:2394|DOID:162|DOID:1612|DOID:5672 pubmed:17611497 R-HSA-2399941|R-HSA-2243942|R-HSA-2399969|R-HSA-2400001|R-HSA-2399981|R-HSA-2399982|R-HSA-2399977|R-HSA-2399997|R-HSA-2399985|R-HSA-2399966 "AKT1 E17K mutant phosphorylates BAD|PDPK1 phosphorylates AKT1 E17K mutant|AKT1 E17K mutant phosphorylates p21Cip1 and p27Kip1|AKT1 E17K mutant phosphorylates CHUK (IKKalpha)|AKT1 E17K mutant phosphorylates MDM2|AKT1 E17K mutant phosphorylates TSC2, inhibiting it|AKT1 E17K mutant phosphorylates AKT1S1 (PRAS40)|AKT1 E17K mutant translocates to the nucleus|AKT1 E17K mutant phosphorylates caspase-9|AKT1 E17K mutant phosphorylates GSK3" gain_of_function pubmed:20440266|pubmed:9381178|pubmed:9895304|pubmed:18954143|pubmed:17611497|pubmed:18256540|pubmed:12244303|pubmed:10485710|pubmed:15527798|pubmed:15169778|pubmed:11715018|pubmed:12172553|pubmed:12150915|pubmed:12524439|pubmed:12767043|pubmed:9812896|pubmed:12668767|pubmed:9478990 R-HSA-5674400 Constitutive Signaling by AKT1 E17K in Cancer R-HSA-198347|R-HSA-198613|R-HSA-198611|R-HSA-198599|R-HSA-198609|R-HSA-200143|R-HSA-198298|R-HSA-198621|R-HSA-198371 "AKT phosphorylates BAD|AKT phosphorylates p21Cip1 and p27Kip1|AKT phosphorylates IKKalpha|AKT phosphorylates MDM2|AKT phosphorylates TSC2, inhibiting it|AKT phosphorylates AKT1S1 (PRAS40)|AKT translocates to the nucleus|AKT phosphorylates caspase-9|AKT phosphorylates GSK3" PIP3 activates AKT signaling R-HSA-1257604 43491 protein kinase B signaling 51897 positive regulation of protein kinase B signaling missense gain_of_function of positive regulation of protein kinase B signaling

PS3-PS1 AKT1 "p-T308,S473-AKT1 E17K [nucleoplasm]" R-HSA-2399994 AKT1 UniProt:P31749 L-glutamic acid 17 replaced with L-lysine ReplacedResidue COSMIC:COSM34142|COSMIC:COSV62571334 ovarian cancer|cancer|breast cancer|large intestine cancer DOID:2394|DOID:162|DOID:1612|DOID:5672 pubmed:17611497 R-HSA-2399988|R-HSA-2399997|R-HSA-2399996|R-HSA-2399999|R-HSA-2399992 AKT1 E17K mutant phosphorylates NR4A1 (NUR77)|AKT1 E17K mutant translocates to the nucleus|AKT1 E17K mutant phosphorylates CREB1|AKT1 E17K mutant phosphorylates RSK|AKT1 E17K mutant phosphorylates forkhead box transcription factors gain_of_function pubmed:11274386|pubmed:12767043|pubmed:9829964|pubmed:10490848|pubmed:10102273|pubmed:10358075|pubmed:17611497|pubmed:16272144 R-HSA-5674400 Constitutive Signaling by AKT1 E17K in Cancer R-HSA-199863|R-HSA-198298|R-HSA-199298|R-HSA-199839|R-HSA-199299 AKT can phosphorylate NR4A1 (NUR77)|AKT translocates to the nucleus|AKT phosphorylates CREB1|AKT can phosphorylate RSK|AKT phosphorylates FOXO transcription factors PIP3 activates AKT signaling R-HSA-1257604 43491 protein kinase B signaling 51897 positive regulation of protein kinase B signaling missense gain_of_function of positive regulation of protein kinase B signaling

PS3 ALDOB ALDOB A174D [cytosol] R-HSA-5656456 ALDOB UniProt:P05062 L-alanine 174 replaced with L-aspartic acid ReplacedResidue hereditary fructose intolerance syndrome DOID:9869 pubmed:1967768 R-HSA-5656450-ALDOB mutant proteins [cytosol]-DefinedSet-hasMember R-HSA-5656438 Defective ALDOB does not cleave Fru 1-P to GA and DHAP loss_of_function pubmed:8535439|pubmed:2889861|pubmed:15733923|pubmed:10625657|pubmed:1967768|pubmed:20033295|pubmed:12464284|pubmed:20848650|pubmed:3383242|pubmed:9610797|pubmed:25637246 R-HSA-5657560 Hereditary fructose intolerance R-HSA-70342 ALDOB tetramer cleaves Fru-1-P to GA and DHAP Fructose metabolism R-HSA-5652084 6000 fructose metabolic process missense loss_of_function of fructose metabolic process

PS3 ALDOB ALDOB A149P [cytosol] R-HSA-5656457 ALDOB UniProt:P05062 L-alanine 149 replaced with L-proline ReplacedResidue hereditary fructose intolerance syndrome DOID:9869 pubmed:10625657|pubmed:3383242 R-HSA-5656450-ALDOB mutant proteins [cytosol]-DefinedSet-hasMember R-HSA-5656438 Defective ALDOB does not cleave Fru 1-P to GA and DHAP loss_of_function pubmed:8535439|pubmed:2889861|pubmed:15733923|pubmed:10625657|pubmed:1967768|pubmed:20033295|pubmed:12464284|pubmed:20848650|pubmed:3383242|pubmed:9610797|pubmed:25637246 R-HSA-5657560 Hereditary fructose intolerance R-HSA-70342 ALDOB tetramer cleaves Fru-1-P to GA and DHAP Fructose metabolism R-HSA-5652084 6000 fructose metabolic process missense loss_of_function of fructose metabolic process

PS3 ALG1 ALG1 M377V [endoplasmic reticulum membrane] R-HSA-4549402 ALG1 UniProt:Q9BT22 L-methionine 377 replaced with L-valine ReplacedResidue congenital disorder of glycosylation type I DOID:0050570 R-HSA-4549394-ALG1 mutants [endoplasmic reticulum membrane]-DefinedSet-hasMember R-HSA-4549382 Defective ALG1 does not transfer the first Man to the N-glycan precursor loss_of_function pubmed:14709599|pubmed:14973782|pubmed:20679665|pubmed:14973778 R-HSA-4549380 Defective ALG1 causes CDG-1k R-HSA-446218 Addition of the first mannose to the N-glycan precursor by ALG1 Asparagine N-linked glycosylation R-HSA-446203 18279 protein N-linked glycosylation via asparagine missense loss_of_function of protein N-linked glycosylation via asparagine

PS3 ALG1 ALG1 E342P [endoplasmic reticulum membrane] R-HSA-4549371 ALG1 UniProt:Q9BT22 L-glutamic acid 342 replaced with L-proline ReplacedResidue congenital disorder of glycosylation type I DOID:0050570 R-HSA-4549394-ALG1 mutants [endoplasmic reticulum membrane]-DefinedSet-hasMember R-HSA-4549382 Defective ALG1 does not transfer the first Man to the N-glycan precursor loss_of_function pubmed:14709599|pubmed:14973782|pubmed:20679665|pubmed:14973778 R-HSA-4549380 Defective ALG1 causes CDG-1k R-HSA-446218 Addition of the first mannose to the N-glycan precursor by ALG1 Asparagine N-linked glycosylation R-HSA-446203 18279 protein N-linked glycosylation via asparagine missense loss_of_function of protein N-linked glycosylation via asparagine

PS3 ALG1 ALG1 S258L [endoplasmic reticulum membrane] R-HSA-4549385 ALG1 UniProt:Q9BT22 L-serine 258 replaced with L-leucine ReplacedResidue congenital disorder of glycosylation type I DOID:0050570 R-HSA-4549394-ALG1 mutants [endoplasmic reticulum membrane]-DefinedSet-hasMember R-HSA-4549382 Defective ALG1 does not transfer the first Man to the N-glycan precursor loss_of_function pubmed:14709599|pubmed:14973782|pubmed:20679665|pubmed:14973778 R-HSA-4549380 Defective ALG1 causes CDG-1k R-HSA-446218 Addition of the first mannose to the N-glycan precursor by ALG1 Asparagine N-linked glycosylation R-HSA-446203 18279 protein N-linked glycosylation via asparagine missense loss_of_function of protein N-linked glycosylation via asparagine

PS3 ALG1 ALG1 R276W [endoplasmic reticulum membrane] R-HSA-4549401 ALG1 UniProt:Q9BT22 L-arginine 276 replaced with L-tryptophan ReplacedResidue congenital disorder of glycosylation type I DOID:0050570 R-HSA-4549394-ALG1 mutants [endoplasmic reticulum membrane]-DefinedSet-hasMember R-HSA-4549382 Defective ALG1 does not transfer the first Man to the N-glycan precursor loss_of_function pubmed:14709599|pubmed:14973782|pubmed:20679665|pubmed:14973778 R-HSA-4549380 Defective ALG1 causes CDG-1k R-HSA-446218 Addition of the first mannose to the N-glycan precursor by ALG1 Asparagine N-linked glycosylation R-HSA-446203 18279 protein N-linked glycosylation via asparagine missense loss_of_function of protein N-linked glycosylation via asparagine

PS3 ALG1 ALG1 S150R [endoplasmic reticulum membrane] R-HSA-4549311 ALG1 UniProt:Q9BT22 L-serine 150 replaced with L-arginine ReplacedResidue congenital disorder of glycosylation type I DOID:0050570 R-HSA-4549394-ALG1 mutants [endoplasmic reticulum membrane]-DefinedSet-hasMember R-HSA-4549382 Defective ALG1 does not transfer the first Man to the N-glycan precursor loss_of_function pubmed:14709599|pubmed:14973782|pubmed:20679665|pubmed:14973778 R-HSA-4549380 Defective ALG1 causes CDG-1k R-HSA-446218 Addition of the first mannose to the N-glycan precursor by ALG1 Asparagine N-linked glycosylation R-HSA-446203 18279 protein N-linked glycosylation via asparagine missense loss_of_function of protein N-linked glycosylation via asparagine

PS3 ALG1 ALG1 C396* [endoplasmic reticulum membrane] R-HSA-4549314 ALG1 UniProt:Q9BT22 Nonsense mutation at L-cysteine 396 NonsenseMutation congenital disorder of glycosylation type I DOID:0050570 R-HSA-4549394-ALG1 mutants [endoplasmic reticulum membrane]-DefinedSet-hasMember R-HSA-4549382 Defective ALG1 does not transfer the first Man to the N-glycan precursor loss_of_function pubmed:14709599|pubmed:14973782|pubmed:20679665|pubmed:14973778 R-HSA-4549380 Defective ALG1 causes CDG-1k R-HSA-446218 Addition of the first mannose to the N-glycan precursor by ALG1 Asparagine N-linked glycosylation R-HSA-446203 18279 protein N-linked glycosylation via asparagine nonsense loss_of_function of protein N-linked glycosylation via asparagine

PS3 ALG1 ALG1 G145D [endoplasmic reticulum membrane] R-HSA-4549398 ALG1 UniProt:Q9BT22 glycine 145 replaced with L-aspartic acid ReplacedResidue congenital disorder of glycosylation type I DOID:0050570 R-HSA-4549394-ALG1 mutants [endoplasmic reticulum membrane]-DefinedSet-hasMember R-HSA-4549382 Defective ALG1 does not transfer the first Man to the N-glycan precursor loss_of_function pubmed:14709599|pubmed:14973782|pubmed:20679665|pubmed:14973778 R-HSA-4549380 Defective ALG1 causes CDG-1k R-HSA-446218 Addition of the first mannose to the N-glycan precursor by ALG1 Asparagine N-linked glycosylation R-HSA-446203 18279 protein N-linked glycosylation via asparagine missense loss_of_function of protein N-linked glycosylation via asparagine

PS3 ALG11 ALG11 Y279S [endoplasmic reticulum membrane] R-HSA-4570575 ALG11 UniProt:Q2TAA5 L-tyrosine 279 replaced with L-serine ReplacedResidue congenital disorder of glycosylation type I DOID:0050570 R-HSA-4570572-ALG11 mutants [endoplasmic reticulum membrane]-DefinedSet-hasMember R-HSA-4551297 Defective ALG11 does not transfer Man to the N-glycan precursor loss_of_function pubmed:20080937|pubmed:22213132 R-HSA-4551295 Defective ALG11 causes CDG-1p R-HSA-446187 ALG11 transfers the fourth and fifth Man to the N-glycan precursor Asparagine N-linked glycosylation R-HSA-446203 18279 protein N-linked glycosylation via asparagine missense loss_of_function of protein N-linked glycosylation via asparagine

PS3 ALG11 ALG11 E398K [endoplasmic reticulum membrane] R-HSA-4570576 ALG11 UniProt:Q2TAA5 L-glutamic acid 398 replaced with L-lysine ReplacedResidue congenital disorder of glycosylation type I DOID:0050570 R-HSA-4570572-ALG11 mutants [endoplasmic reticulum membrane]-DefinedSet-hasMember R-HSA-4551297 Defective ALG11 does not transfer Man to the N-glycan precursor loss_of_function pubmed:20080937|pubmed:22213132 R-HSA-4551295 Defective ALG11 causes CDG-1p R-HSA-446187 ALG11 transfers the fourth and fifth Man to the N-glycan precursor Asparagine N-linked glycosylation R-HSA-446203 18279 protein N-linked glycosylation via asparagine missense loss_of_function of protein N-linked glycosylation via asparagine

PS3 ALG11 ALG11 Q318P [endoplasmic reticulum membrane] R-HSA-4570582 ALG11 UniProt:Q2TAA5 L-glutamine 318 replaced with L-proline ReplacedResidue congenital disorder of glycosylation type I DOID:0050570 R-HSA-4570572-ALG11 mutants [endoplasmic reticulum membrane]-DefinedSet-hasMember R-HSA-4551297 Defective ALG11 does not transfer Man to the N-glycan precursor loss_of_function pubmed:20080937|pubmed:22213132 R-HSA-4551295 Defective ALG11 causes CDG-1p R-HSA-446187 ALG11 transfers the fourth and fifth Man to the N-glycan precursor Asparagine N-linked glycosylation R-HSA-446203 18279 protein N-linked glycosylation via asparagine missense loss_of_function of protein N-linked glycosylation via asparagine

PS3 ALG11 ALG11 L86S [endoplasmic reticulum membrane] R-HSA-4570580 ALG11 UniProt:Q2TAA5 L-leucine 86 replaced with L-serine ReplacedResidue congenital disorder of glycosylation type I DOID:0050570 R-HSA-4570572-ALG11 mutants [endoplasmic reticulum membrane]-DefinedSet-hasMember R-HSA-4551297 Defective ALG11 does not transfer Man to the N-glycan precursor loss_of_function pubmed:20080937|pubmed:22213132 R-HSA-4551295 Defective ALG11 causes CDG-1p R-HSA-446187 ALG11 transfers the fourth and fifth Man to the N-glycan precursor Asparagine N-linked glycosylation R-HSA-446203 18279 protein N-linked glycosylation via asparagine missense loss_of_function of protein N-linked glycosylation via asparagine

PS3 ALG11 ALG11 L381S [endoplasmic reticulum membrane] R-HSA-4570581 ALG11 UniProt:Q2TAA5 L-leucine 381 replaced with L-serine ReplacedResidue congenital disorder of glycosylation type I DOID:0050570 R-HSA-4570572-ALG11 mutants [endoplasmic reticulum membrane]-DefinedSet-hasMember R-HSA-4551297 Defective ALG11 does not transfer Man to the N-glycan precursor loss_of_function pubmed:20080937|pubmed:22213132 R-HSA-4551295 Defective ALG11 causes CDG-1p R-HSA-446187 ALG11 transfers the fourth and fifth Man to the N-glycan precursor Asparagine N-linked glycosylation R-HSA-446203 18279 protein N-linked glycosylation via asparagine missense loss_of_function of protein N-linked glycosylation via asparagine

PS3 ALG12 ALG12 T67M [endoplasmic reticulum membrane] R-HSA-4722118 ALG12 UniProt:Q9BV10 L-threonine 67 replaced with L-methionine ReplacedResidue congenital disorder of glycosylation type I DOID:0050570 R-HSA-4722114-ALG12 mutants [endoplasmic reticulum membrane]-DefinedSet-hasMember R-HSA-4720497 Defective ALG12 does not add mannose to the N-glycan precursor loss_of_function pubmed:11983712|pubmed:12217961 R-HSA-4720489 Defective ALG12 causes CDG-1g R-HSA-446198 ALG12 transfers Man to N-glycan precursor (GlcNAc)2 (Man)7 (PP-Dol)1 Asparagine N-linked glycosylation R-HSA-446203 18279 protein N-linked glycosylation via asparagine missense loss_of_function of protein N-linked glycosylation via asparagine

PS3 ALG12 ALG12 G101R [endoplasmic reticulum membrane] R-HSA-4722101 ALG12 UniProt:Q9BV10 glycine 101 replaced with L-arginine ReplacedResidue congenital disorder of glycosylation type I DOID:0050570 R-HSA-4722114-ALG12 mutants [endoplasmic reticulum membrane]-DefinedSet-hasMember R-HSA-4720497 Defective ALG12 does not add mannose to the N-glycan precursor loss_of_function pubmed:11983712|pubmed:12217961 R-HSA-4720489 Defective ALG12 causes CDG-1g R-HSA-446198 ALG12 transfers Man to N-glycan precursor (GlcNAc)2 (Man)7 (PP-Dol)1 Asparagine N-linked glycosylation R-HSA-446203 18279 protein N-linked glycosylation via asparagine missense loss_of_function of protein N-linked glycosylation via asparagine

PS3 ALG12 ALG12 L158P [endoplasmic reticulum membrane] R-HSA-4722115 ALG12 UniProt:Q9BV10 L-leucine 158 replaced with L-proline ReplacedResidue congenital disorder of glycosylation type I DOID:0050570 R-HSA-4722114-ALG12 mutants [endoplasmic reticulum membrane]-DefinedSet-hasMember R-HSA-4720497 Defective ALG12 does not add mannose to the N-glycan precursor loss_of_function pubmed:11983712|pubmed:12217961 R-HSA-4720489 Defective ALG12 causes CDG-1g R-HSA-446198 ALG12 transfers Man to N-glycan precursor (GlcNAc)2 (Man)7 (PP-Dol)1 Asparagine N-linked glycosylation R-HSA-446203 18279 protein N-linked glycosylation via asparagine missense loss_of_function of protein N-linked glycosylation via asparagine

PS3 ALG12 ALG12 R146Q [endoplasmic reticulum membrane] R-HSA-4722098 ALG12 UniProt:Q9BV10 L-arginine 146 replaced with L-glutamine ReplacedResidue congenital disorder of glycosylation type I DOID:0050570 R-HSA-4722114-ALG12 mutants [endoplasmic reticulum membrane]-DefinedSet-hasMember R-HSA-4720497 Defective ALG12 does not add mannose to the N-glycan precursor loss_of_function pubmed:11983712|pubmed:12217961 R-HSA-4720489 Defective ALG12 causes CDG-1g R-HSA-446198 ALG12 transfers Man to N-glycan precursor (GlcNAc)2 (Man)7 (PP-Dol)1 Asparagine N-linked glycosylation R-HSA-446203 18279 protein N-linked glycosylation via asparagine missense loss_of_function of protein N-linked glycosylation via asparagine

PS3 ALG12 ALG12 F142V [endoplasmic reticulum membrane] R-HSA-4722108 ALG12 UniProt:Q9BV10 L-phenylalanine 142 replaced with L-valine ReplacedResidue congenital disorder of glycosylation type I DOID:0050570 R-HSA-4722114-ALG12 mutants [endoplasmic reticulum membrane]-DefinedSet-hasMember R-HSA-4720497 Defective ALG12 does not add mannose to the N-glycan precursor loss_of_function pubmed:11983712|pubmed:12217961 R-HSA-4720489 Defective ALG12 causes CDG-1g R-HSA-446198 ALG12 transfers Man to N-glycan precursor (GlcNAc)2 (Man)7 (PP-Dol)1 Asparagine N-linked glycosylation R-HSA-446203 18279 protein N-linked glycosylation via asparagine missense loss_of_function of protein N-linked glycosylation via asparagine

PS3 ALG12 ALG12 Y414* [endoplasmic reticulum membrane] R-HSA-4722121 ALG12 UniProt:Q9BV10 Nonsense mutation at L-tyrosine 414 NonsenseMutation congenital disorder of glycosylation type I DOID:0050570 R-HSA-4722114-ALG12 mutants [endoplasmic reticulum membrane]-DefinedSet-hasMember R-HSA-4720497 Defective ALG12 does not add mannose to the N-glycan precursor loss_of_function pubmed:11983712|pubmed:12217961 R-HSA-4720489 Defective ALG12 causes CDG-1g R-HSA-446198 ALG12 transfers Man to N-glycan precursor (GlcNAc)2 (Man)7 (PP-Dol)1 Asparagine N-linked glycosylation R-HSA-446203 18279 protein N-linked glycosylation via asparagine nonsense loss_of_function of protein N-linked glycosylation via asparagine

PS3 ALG14 ALG14 R104* [endoplasmic reticulum membrane] R-HSA-5633229 ALG14 UniProt:Q96F25 Nonsense mutation at L-arginine 104 NonsenseMutation congenital myasthenic syndrome DOID:3635 R-HSA-5633238-ALG14 mutants [endoplasmic reticulum membrane]-DefinedSet-hasMember R-HSA-5633241 Defective ALG14 does not transfer GlcNAc from UDP-GlcNAc to GlcNAcDOLP loss_of_function pubmed:16100110|pubmed:23404334 R-HSA-5633231 Defective ALG14 causes ALG14-CMS R-HSA-446207 ALG13:ALG14 transfers GlcNAc from UDP-GlcNAc to GlcNAcDOLP Asparagine N-linked glycosylation R-HSA-446203 18279 protein N-linked glycosylation via asparagine nonsense loss_of_function of protein N-linked glycosylation via asparagine

PS3 ALG14 ALG14 P65L [endoplasmic reticulum membrane] R-HSA-5633218 ALG14 UniProt:Q96F25 L-proline 65 replaced with L-leucine ReplacedResidue congenital myasthenic syndrome DOID:3635 R-HSA-5633238-ALG14 mutants [endoplasmic reticulum membrane]-DefinedSet-hasMember R-HSA-5633241 Defective ALG14 does not transfer GlcNAc from UDP-GlcNAc to GlcNAcDOLP loss_of_function pubmed:16100110|pubmed:23404334 R-HSA-5633231 Defective ALG14 causes ALG14-CMS R-HSA-446207 ALG13:ALG14 transfers GlcNAc from UDP-GlcNAc to GlcNAcDOLP Asparagine N-linked glycosylation R-HSA-446203 18279 protein N-linked glycosylation via asparagine missense loss_of_function of protein N-linked glycosylation via asparagine

PS3 ALG2 ALG2 G347Vfs*27 [endoplasmic reticulum membrane] R-HSA-4549361 ALG2 UniProt:Q9H553 Replacement of residues 347 to 372 by VDPWSPLTTVSQGFCVSLTRCTSQKQ FragmentReplacedModification congenital disorder of glycosylation type I DOID:0050570 R-HSA-5633219-ALG2 mutants [endoplasmic reticulum membrane]-DefinedSet-hasMember R-HSA-4549368 Defective ALG2 does not transfer a second Man to N-glycan precursor loss_of_function pubmed:12684507 R-HSA-4549349 Defective ALG2 causes CDG-1i R-HSA-446208 Addition of a second mannose to the N-glycan precursor by ALG2 Asparagine N-linked glycosylation R-HSA-446203 18279 protein N-linked glycosylation via asparagine frameshift loss_of_function of protein N-linked glycosylation via asparagine

PS3 ALG2 ALG2 72_75delinsSPR [endoplasmic reticulum membrane] R-HSA-5633223 ALG2 UniProt:Q9H553 Replacement of residues 72 to 75 by SPR FragmentReplacedModification congenital myasthenic syndrome DOID:3635 R-HSA-5633219-ALG2 mutants [endoplasmic reticulum membrane]-DefinedSet-hasMember R-HSA-4549368 Defective ALG2 does not transfer a second Man to N-glycan precursor loss_of_function pubmed:12684507 R-HSA-4549349 Defective ALG2 causes CDG-1i R-HSA-446208 Addition of a second mannose to the N-glycan precursor by ALG2 Asparagine N-linked glycosylation R-HSA-446203 18279 protein N-linked glycosylation via asparagine in-frame indel: indel loss_of_function of protein N-linked glycosylation via asparagine

PS3 ALG2 ALG2 V68G [endoplasmic reticulum membrane] R-HSA-5633236 ALG2 UniProt:Q9H553 L-valine 68 replaced with glycine ReplacedResidue congenital myasthenic syndrome DOID:3635 R-HSA-5633219-ALG2 mutants [endoplasmic reticulum membrane]-DefinedSet-hasMember R-HSA-4549368 Defective ALG2 does not transfer a second Man to N-glycan precursor loss_of_function pubmed:12684507 R-HSA-4549349 Defective ALG2 causes CDG-1i R-HSA-446208 Addition of a second mannose to the N-glycan precursor by ALG2 Asparagine N-linked glycosylation R-HSA-446203 18279 protein N-linked glycosylation via asparagine missense loss_of_function of protein N-linked glycosylation via asparagine

PS3 ALG3 ALG3 M157K [endoplasmic reticulum membrane] R-HSA-4720484 ALG3 UniProt:Q92685 L-methionine 157 replaced with L-lysine ReplacedResidue congenital disorder of glycosylation type I DOID:0050570 R-HSA-4720485-ALG3 mutants [endoplasmic reticulum membrane]-DefinedSet-hasMember R-HSA-4720473 Defective ALG3 does not add mannose to the N-glycan precursor loss_of_function pubmed:10581255|pubmed:17551933|pubmed:15840742 R-HSA-4720475 Defective ALG3 causes CDG-1d R-HSA-446188 ALG3 transfers Man to N-glycan precursor (GlcNAc)2 (Man)5 (PP-Dol)1 Asparagine N-linked glycosylation R-HSA-446203 18279 protein N-linked glycosylation via asparagine missense loss_of_function of protein N-linked glycosylation via asparagine

PS3 ALG3 ALG3 G118D [endoplasmic reticulum membrane] R-HSA-4720468 ALG3 UniProt:Q92685 glycine 118 replaced with L-aspartic acid ReplacedResidue congenital disorder of glycosylation type I DOID:0050570 R-HSA-4720485-ALG3 mutants [endoplasmic reticulum membrane]-DefinedSet-hasMember R-HSA-4720473 Defective ALG3 does not add mannose to the N-glycan precursor loss_of_function pubmed:10581255|pubmed:17551933|pubmed:15840742 R-HSA-4720475 Defective ALG3 causes CDG-1d R-HSA-446188 ALG3 transfers Man to N-glycan precursor (GlcNAc)2 (Man)5 (PP-Dol)1 Asparagine N-linked glycosylation R-HSA-446203 18279 protein N-linked glycosylation via asparagine missense loss_of_function of protein N-linked glycosylation via asparagine

PS3 ALG3 ALG3 W71R [endoplasmic reticulum membrane] R-HSA-4720466 ALG3 UniProt:Q92685 L-tryptophan 71 replaced with L-arginine ReplacedResidue congenital disorder of glycosylation type I DOID:0050570 R-HSA-4720485-ALG3 mutants [endoplasmic reticulum membrane]-DefinedSet-hasMember R-HSA-4720473 Defective ALG3 does not add mannose to the N-glycan precursor loss_of_function pubmed:10581255|pubmed:17551933|pubmed:15840742 R-HSA-4720475 Defective ALG3 causes CDG-1d R-HSA-446188 ALG3 transfers Man to N-glycan precursor (GlcNAc)2 (Man)5 (PP-Dol)1 Asparagine N-linked glycosylation R-HSA-446203 18279 protein N-linked glycosylation via asparagine missense loss_of_function of protein N-linked glycosylation via asparagine

PS3 ALG6 ALG6 A333V [endoplasmic reticulum membrane] R-HSA-4724295 ALG6 UniProt:Q9Y672 L-alanine 333 replaced with L-valine ReplacedResidue congenital disorder of glycosylation type I DOID:0050570 R-HSA-4724300-ALG6 mutants [endoplasmic reticulum membrane]-DefinedSet-hasMember R-HSA-4724291 Defective ALG6 does not add glucose to the N-glycan precursor loss_of_function pubmed:10359825|pubmed:23430515|pubmed:16007612|pubmed:10914684|pubmed:10924277 R-HSA-4724289 Defective ALG6 causes CDG-1c R-HSA-446202 Addition of the first glucose to the N-glycan precursor by ALG6 Asparagine N-linked glycosylation R-HSA-446203 18279 protein N-linked glycosylation via asparagine missense loss_of_function of protein N-linked glycosylation via asparagine

PS3 ALG6 ALG6 S478P [endoplasmic reticulum membrane] R-HSA-4724296 ALG6 UniProt:Q9Y672 L-alanine 333 replaced with L-valine|L-serine 478 replaced with L-proline ReplacedResidue congenital disorder of glycosylation type I DOID:0050570 R-HSA-4724300-ALG6 mutants [endoplasmic reticulum membrane]-DefinedSet-hasMember R-HSA-4724291 Defective ALG6 does not add glucose to the N-glycan precursor loss_of_function pubmed:10359825|pubmed:23430515|pubmed:16007612|pubmed:10914684|pubmed:10924277 R-HSA-4724289 Defective ALG6 causes CDG-1c R-HSA-446202 Addition of the first glucose to the N-glycan precursor by ALG6 Asparagine N-linked glycosylation R-HSA-446203 18279 protein N-linked glycosylation via asparagine missense loss_of_function of protein N-linked glycosylation via asparagine

PS3 ALG8 ALG8 G275D [endoplasmic reticulum membrane] R-HSA-4724304 ALG8 UniProt:Q9BVK2 glycine 275 replaced with L-aspartic acid ReplacedResidue congenital disorder of glycosylation type I DOID:0050570 R-HSA-4724315-ALG8 mutants [endoplasmic reticulum membrane]-DefinedSet-hasMember R-HSA-4724330 Defective ALG8 does not add glucose to the N-glycan precursor loss_of_function pubmed:12480927|pubmed:15235028 R-HSA-4724325 Defective ALG8 causes CDG-1h R-HSA-446189 Addition of a second glucose to the N-glycan precursor by ALG8 Asparagine N-linked glycosylation R-HSA-446203 18279 protein N-linked glycosylation via asparagine missense loss_of_function of protein N-linked glycosylation via asparagine

PS3 ALG8 ALG8 T47P [endoplasmic reticulum membrane] R-HSA-4724334 ALG8 UniProt:Q9BVK2 L-threonine 47 replaced with L-proline ReplacedResidue congenital disorder of glycosylation type I DOID:0050570 R-HSA-4724315-ALG8 mutants [endoplasmic reticulum membrane]-DefinedSet-hasMember R-HSA-4724330 Defective ALG8 does not add glucose to the N-glycan precursor loss_of_function pubmed:12480927|pubmed:15235028 R-HSA-4724325 Defective ALG8 causes CDG-1h R-HSA-446189 Addition of a second glucose to the N-glycan precursor by ALG8 Asparagine N-linked glycosylation R-HSA-446203 18279 protein N-linked glycosylation via asparagine missense loss_of_function of protein N-linked glycosylation via asparagine

PS3 ALG8 ALG8 V133Sfs*3 [endoplasmic reticulum membrane] R-HSA-4724313 ALG8 UniProt:Q9BVK2 Replacement of residues 133 to 134 by SG FragmentReplacedModification congenital disorder of glycosylation type I DOID:0050570 R-HSA-4724315-ALG8 mutants [endoplasmic reticulum membrane]-DefinedSet-hasMember R-HSA-4724330 Defective ALG8 does not add glucose to the N-glycan precursor loss_of_function pubmed:12480927|pubmed:15235028 R-HSA-4724325 Defective ALG8 causes CDG-1h R-HSA-446189 Addition of a second glucose to the N-glycan precursor by ALG8 Asparagine N-linked glycosylation R-HSA-446203 18279 protein N-linked glycosylation via asparagine frameshift loss_of_function of protein N-linked glycosylation via asparagine

PS3 ALG8 ALG8 T138Kfs*19 [endoplasmic reticulum membrane] R-HSA-4724312 ALG8 UniProt:Q9BVK2 Replacement of residues 138 to 155 by KKSQNLFCRYYFCGTSGY FragmentReplacedModification congenital disorder of glycosylation type I DOID:0050570 R-HSA-4724315-ALG8 mutants [endoplasmic reticulum membrane]-DefinedSet-hasMember R-HSA-4724330 Defective ALG8 does not add glucose to the N-glycan precursor loss_of_function pubmed:12480927|pubmed:15235028 R-HSA-4724325 Defective ALG8 causes CDG-1h R-HSA-446189 Addition of a second glucose to the N-glycan precursor by ALG8 Asparagine N-linked glycosylation R-HSA-446203 18279 protein N-linked glycosylation via asparagine frameshift loss_of_function of protein N-linked glycosylation via asparagine

PS3 ALG9 ALG9 E523K [endoplasmic reticulum membrane] R-HSA-4720482 ALG9 UniProt:Q9H6U8 L-glutamic acid 523 replaced with L-lysine ReplacedResidue congenital disorder of glycosylation type I DOID:0050570 R-HSA-4720499-ALG9 mutants [endoplasmic reticulum membrane]-DefinedSet-hasMember R-HSA-9035514|R-HSA-4720478 Defective ALG9 does not add the last mannose to the N-glycan precursor|Defective ALG9 does not add the seventh mannose to the N-glycan precursor loss_of_function pubmed:15148656|pubmed:15945070 R-HSA-4720454 Defective ALG9 causes CDG-1l R-HSA-446216|R-HSA-446215 ALG9 transfers Man to N-glycan precursor (GlcNAc)2 (Man)8 (PP-Dol)1|ALG9 transfers Man to N-glycan precursor (GlcNAc)2 (Man)6 (PP-Dol)1 Asparagine N-linked glycosylation R-HSA-446203 18279 protein N-linked glycosylation via asparagine missense loss_of_function of protein N-linked glycosylation via asparagine

PS3 ALG9 ALG9 Y286C [endoplasmic reticulum membrane] R-HSA-4720474 ALG9 UniProt:Q9H6U8 L-tyrosine 286 replaced with L-cysteine ReplacedResidue congenital disorder of glycosylation type I DOID:0050570 R-HSA-4720499-ALG9 mutants [endoplasmic reticulum membrane]-DefinedSet-hasMember R-HSA-9035514|R-HSA-4720478 Defective ALG9 does not add the last mannose to the N-glycan precursor|Defective ALG9 does not add the seventh mannose to the N-glycan precursor loss_of_function pubmed:15148656|pubmed:15945070 R-HSA-4720454 Defective ALG9 causes CDG-1l R-HSA-446216|R-HSA-446215 ALG9 transfers Man to N-glycan precursor (GlcNAc)2 (Man)8 (PP-Dol)1|ALG9 transfers Man to N-glycan precursor (GlcNAc)2 (Man)6 (PP-Dol)1 Asparagine N-linked glycosylation R-HSA-446203 18279 protein N-linked glycosylation via asparagine missense loss_of_function of protein N-linked glycosylation via asparagine

PS3-PS1 ALK ALK F1174I [plasma membrane] R-HSA-9699794 ALK UniProt:Q9UM73 L-phenylalanine 1174 replaced with L-isoleucine ReplacedResidue COSMIC:COSM4169861|COSMIC:COSV66568713 neuroblastoma DOID:769 R-HSA-9700555-alectinib sensitve ALK mutants [plasma membrane]-DefinedSet-hasMember|R-HSA-9700528-ceritinib-sensitive ALK mutants [plasma membrane]-DefinedSet-hasMember|R-HSA-9700082-kinase domain ALK mutant dimers [plasma membrane]-CandidateSet-hasCandidate|R-HSA-9700083-kinase domain ALK mutants [plasma membrane]-CandidateSet-hasCandidate R-HSA-9700131|R-HSA-9700186|R-HSA-9700184 ALK mutants bind type I TKIs|Autophosphorylation of point mutants of ALK|Ligand-independent dimerization of point mutants of ALK pubmed:24613930|pubmed:21494621|pubmed:21948233|pubmed:28404650|pubmed:22570254|pubmed:26301689|pubmed:24496003|pubmed:27245569|pubmed:26939704|pubmed:25393796|pubmed:25421750|pubmed:25228534|pubmed:24675041|pubmed:26464158|pubmed:18923525|pubmed:26001147|pubmed:23239810|pubmed:24518094|pubmed:17185414|pubmed:31388026|pubmed:21502504|pubmed:18724359|pubmed:29455642|pubmed:24060861|pubmed:21847362|pubmed:18923524|pubmed:18923523|pubmed:21596819|pubmed:21838707|pubmed:27573755|pubmed:23104988|pubmed:23201355 R-HSA-9700645|R-HSA-9725370 ALK mutants bind TKIs|Signaling by ALK fusions and activated point mutants R-HSA-9700662|R-HSA-201521|R-HSA-201519 (PTN/MDK):ALK dimer binds type I ALK-binding TKIs|ALK autophosphorylation downstream of PTN and MDK|ALK:PTN dimerization Signaling by ALK R-HSA-201556 missense NA

PS3-PS1 ALK ALK F1174L [plasma membrane] R-HSA-9699801 ALK UniProt:Q9UM73 L-phenylalanine 1174 replaced with L-leucine ReplacedResidue COSMIC:COSM88796|COSMIC:COSV66555460|COSMIC:COSV66556166|COSMIC:COSV66556325 neuroblastoma DOID:769 pubmed:18724359|pubmed:18923525|pubmed:20632993 R-HSA-9700555-alectinib sensitve ALK mutants [plasma membrane]-DefinedSet-hasMember|R-HSA-9700081-NVP-TAE684-sensitive ALK mutants [cytosol]-DefinedSet-hasMember|R-HSA-9717270-ASP3026-resistant ALK mutants [plasma membrane]-DefinedSet-hasMember|R-HSA-9700564-crizotinib resistant ALK mutants [plasma membrane]-DefinedSet-hasMember|R-HSA-9700553-ceritinib-resistant ALK mutants [plasma membrane]-DefinedSet-hasMember|R-HSA-9700082-kinase domain ALK mutant dimers [plasma membrane]-CandidateSet-hasMember|R-HSA-9700083-kinase domain ALK mutants [plasma membrane]-CandidateSet-hasMember R-HSA-9700131|R-HSA-9700646|R-HSA-9715357|R-HSA-9700658|R-HSA-9700186|R-HSA-9700184 ALK mutants bind type I TKIs|ASP-3026- resistant ALK mutants don't bind ASP-3026|crizotinib-resistant ALK mutants don't bind crizotinib|ceritinib-resistant ALK mutants don't bind ceritinib|Autophosphorylation of point mutants of ALK|Ligand-independent dimerization of point mutants of ALK loss_of_function pubmed:24613930|pubmed:21494621|pubmed:21948233|pubmed:28404650|pubmed:22570254|pubmed:26301689|pubmed:24496003|pubmed:27245569|pubmed:26939704|pubmed:25393796|pubmed:25421750|pubmed:25228534|pubmed:24675041|pubmed:26464158|pubmed:18923525|pubmed:26001147|pubmed:23239810|pubmed:24518094|pubmed:17185414|pubmed:31388026|pubmed:21502504|pubmed:27009859|pubmed:31712133|pubmed:25393798|pubmed:22277784|pubmed:30089600|pubmed:27045755|pubmed:20979473|pubmed:21030459|pubmed:27432227|pubmed:23434628|pubmed:21791641|pubmed:30675302|pubmed:22235099|pubmed:24736079|pubmed:23344087|pubmed:31749991|pubmed:30683630|pubmed:18724359|pubmed:29455642|pubmed:24060861|pubmed:21847362|pubmed:18923524|pubmed:18923523|pubmed:21596819|pubmed:21838707|pubmed:27573755|pubmed:23104988|pubmed:23201355 R-HSA-9700645|R-HSA-9717264|R-HSA-9717326|R-HSA-9717323|R-HSA-9725370 ALK mutants bind TKIs|ASP-3026-resistant ALK mutants|crizotinib-resistant ALK mutants|ceritinib-resistant ALK mutants|Signaling by ALK fusions and activated point mutants R-HSA-9700662|R-HSA-201521|R-HSA-201519 (PTN/MDK):ALK dimer binds type I ALK-binding TKIs|ALK autophosphorylation downstream of PTN and MDK|ALK:PTN dimerization Signaling by ALK R-HSA-201556 missense NA

PS3-PS1 ALK ALK F1174V [plasma membrane] R-HSA-9699818 ALK UniProt:Q9UM73 L-phenylalanine 1174 replaced with L-valine ReplacedResidue COSMIC:COSV66559314|COSMIC:COSM4422791 non-small cell lung carcinoma|neuroblastoma DOID:3908|DOID:769 R-HSA-9700555-alectinib sensitve ALK mutants [plasma membrane]-DefinedSet-hasMember|R-HSA-9700552-brigatinib-sensitive ALK mutants [plasma membrane]-DefinedSet-hasMember|R-HSA-9700529-crizotinib-sensitive ALK mutants [cytosol]-DefinedSet-hasMember|R-HSA-9700528-ceritinib-sensitive ALK mutants [plasma membrane]-DefinedSet-hasMember|R-HSA-9700564-crizotinib resistant ALK mutants [plasma membrane]-DefinedSet-hasMember|R-HSA-9700553-ceritinib-resistant ALK mutants [plasma membrane]-DefinedSet-hasMember|R-HSA-9700082-kinase domain ALK mutant dimers [plasma membrane]-CandidateSet-hasCandidate|R-HSA-9700083-kinase domain ALK mutants [plasma membrane]-CandidateSet-hasCandidate R-HSA-9700131|R-HSA-9715357|R-HSA-9700658|R-HSA-9700186|R-HSA-9700184 ALK mutants bind type I TKIs|crizotinib-resistant ALK mutants don't bind crizotinib|ceritinib-resistant ALK mutants don't bind ceritinib|Autophosphorylation of point mutants of ALK|Ligand-independent dimerization of point mutants of ALK loss_of_function pubmed:24613930|pubmed:21494621|pubmed:21948233|pubmed:28404650|pubmed:22570254|pubmed:26301689|pubmed:24496003|pubmed:27245569|pubmed:26939704|pubmed:25393796|pubmed:25421750|pubmed:25228534|pubmed:24675041|pubmed:26464158|pubmed:18923525|pubmed:26001147|pubmed:23239810|pubmed:24518094|pubmed:17185414|pubmed:31388026|pubmed:21502504|pubmed:31712133|pubmed:25393798|pubmed:22277784|pubmed:30089600|pubmed:27045755|pubmed:20979473|pubmed:21030459|pubmed:27432227|pubmed:23434628|pubmed:21791641|pubmed:30675302|pubmed:22235099|pubmed:24736079|pubmed:23344087|pubmed:31749991|pubmed:30683630|pubmed:27009859|pubmed:18724359|pubmed:29455642|pubmed:24060861|pubmed:21847362|pubmed:18923524|pubmed:18923523|pubmed:21596819|pubmed:21838707|pubmed:27573755|pubmed:23104988|pubmed:23201355 R-HSA-9700645|R-HSA-9717326|R-HSA-9717323|R-HSA-9725370 ALK mutants bind TKIs|crizotinib-resistant ALK mutants|ceritinib-resistant ALK mutants|Signaling by ALK fusions and activated point mutants R-HSA-9700662|R-HSA-201521|R-HSA-201519 (PTN/MDK):ALK dimer binds type I ALK-binding TKIs|ALK autophosphorylation downstream of PTN and MDK|ALK:PTN dimerization Signaling by ALK R-HSA-201556 missense NA

PS3 ALK ALK L1196Q [plasma membrane] R-HSA-9700472 ALK UniProt:Q9UM73 L-leucine 1196 replaced with L-asparagine ReplacedResidue COSMIC:COSV66565358 anaplastic large cell lymphoma DOID:0050744 R-HSA-9700552-brigatinib-sensitive ALK mutants [plasma membrane]-DefinedSet-hasMember|R-HSA-9700081-NVP-TAE684-sensitive ALK mutants [cytosol]-DefinedSet-hasMember|R-HSA-9700564-crizotinib resistant ALK mutants [plasma membrane]-DefinedSet-hasMember|R-HSA-9700082-kinase domain ALK mutant dimers [plasma membrane]-CandidateSet-hasMember|R-HSA-9700083-kinase domain ALK mutants [plasma membrane]-CandidateSet-hasMember R-HSA-9700131|R-HSA-9715357|R-HSA-9700186|R-HSA-9700184 ALK mutants bind type I TKIs|crizotinib-resistant ALK mutants don't bind crizotinib|Autophosphorylation of point mutants of ALK|Ligand-independent dimerization of point mutants of ALK loss_of_function pubmed:24613930|pubmed:21494621|pubmed:21948233|pubmed:28404650|pubmed:22570254|pubmed:26301689|pubmed:24496003|pubmed:27245569|pubmed:26939704|pubmed:25393796|pubmed:25421750|pubmed:25228534|pubmed:24675041|pubmed:26464158|pubmed:18923525|pubmed:26001147|pubmed:23239810|pubmed:24518094|pubmed:17185414|pubmed:31388026|pubmed:21502504|pubmed:31712133|pubmed:25393798|pubmed:22277784|pubmed:30089600|pubmed:27045755|pubmed:20979473|pubmed:21030459|pubmed:27432227|pubmed:23434628|pubmed:21791641|pubmed:30675302|pubmed:22235099|pubmed:24736079|pubmed:23344087|pubmed:31749991|pubmed:18724359|pubmed:29455642|pubmed:24060861|pubmed:21847362|pubmed:18923524|pubmed:18923523|pubmed:21596819|pubmed:21838707|pubmed:27573755|pubmed:23104988|pubmed:23201355 R-HSA-9700645|R-HSA-9717326|R-HSA-9725370 ALK mutants bind TKIs|crizotinib-resistant ALK mutants|Signaling by ALK fusions and activated point mutants R-HSA-9700662|R-HSA-201521|R-HSA-201519 (PTN/MDK):ALK dimer binds type I ALK-binding TKIs|ALK autophosphorylation downstream of PTN and MDK|ALK:PTN dimerization Signaling by ALK R-HSA-201556 missense NA

PS3 ALK ALK V1180L [plasma membrane] R-HSA-9715261 ALK UniProt:Q9UM73 L-valine 1180 replaced with L-leucine ReplacedResidue COSMIC:COSV66587894 lung squamous cell carcinoma DOID:3907 pubmed:25228534 R-HSA-9700552-brigatinib-sensitive ALK mutants [plasma membrane]-DefinedSet-hasMember|R-HSA-9716604-ASP-3026-sensitive ALK mutants [plasma membrane]-DefinedSet-hasMember|R-HSA-9700081-NVP-TAE684-sensitive ALK mutants [cytosol]-DefinedSet-hasMember|R-HSA-9700528-ceritinib-sensitive ALK mutants [plasma membrane]-DefinedSet-hasMember|R-HSA-9700560-alectinib resistant ALK mutants [plasma membrane]-DefinedSet-hasMember|R-HSA-9700564-crizotinib resistant ALK mutants [plasma membrane]-DefinedSet-hasMember|R-HSA-9700082-kinase domain ALK mutant dimers [plasma membrane]-CandidateSet-hasMember|R-HSA-9700083-kinase domain ALK mutants [plasma membrane]-CandidateSet-hasMember R-HSA-9700131|R-HSA-9700656|R-HSA-9715357|R-HSA-9700186|R-HSA-9700184 ALK mutants bind type I TKIs|alectinib resistant ALK mutants don't bind alectinib|crizotinib-resistant ALK mutants don't bind crizotinib|Autophosphorylation of point mutants of ALK|Ligand-independent dimerization of point mutants of ALK loss_of_function pubmed:24613930|pubmed:21494621|pubmed:21948233|pubmed:28404650|pubmed:22570254|pubmed:26301689|pubmed:24496003|pubmed:27245569|pubmed:26939704|pubmed:25393796|pubmed:25421750|pubmed:25228534|pubmed:24675041|pubmed:26464158|pubmed:18923525|pubmed:26001147|pubmed:23239810|pubmed:24518094|pubmed:17185414|pubmed:31388026|pubmed:21502504|pubmed:24509625|pubmed:31374369|pubmed:25393798|pubmed:27432227|pubmed:27130468|pubmed:31712133|pubmed:22277784|pubmed:30089600|pubmed:27045755|pubmed:20979473|pubmed:21030459|pubmed:23434628|pubmed:21791641|pubmed:30675302|pubmed:22235099|pubmed:24736079|pubmed:23344087|pubmed:31749991|pubmed:18724359|pubmed:29455642|pubmed:24060861|pubmed:21847362|pubmed:18923524|pubmed:18923523|pubmed:21596819|pubmed:21838707|pubmed:27573755|pubmed:23104988|pubmed:23201355 R-HSA-9700645|R-HSA-9717316|R-HSA-9717326|R-HSA-9725370 ALK mutants bind TKIs|alectinib-resistant ALK mutants|crizotinib-resistant ALK mutants|Signaling by ALK fusions and activated point mutants R-HSA-9700662|R-HSA-201521|R-HSA-201519 (PTN/MDK):ALK dimer binds type I ALK-binding TKIs|ALK autophosphorylation downstream of PTN and MDK|ALK:PTN dimerization Signaling by ALK R-HSA-201556 missense NA

PS3-PS1 ALK ALK L1196M [plasma membrane] R-HSA-9700470 ALK UniProt:Q9UM73 L-leucine 1196 replaced with L-methionine ReplacedResidue COSMIC:COSV66556435|COSMIC:COSM4422786 lung adenocarcinoma|neuroblastoma DOID:3910|DOID:769 R-HSA-9700081-NVP-TAE684-sensitive ALK mutants [cytosol]-DefinedSet-hasMember|R-HSA-9700560-alectinib resistant ALK mutants [plasma membrane]-DefinedSet-hasMember|R-HSA-9714046-brigatinib-resistant ALK mutants [plasma membrane]-DefinedSet-hasMember|R-HSA-9714053-lorlatinib-resistant ALK mutants [plasma membrane]-DefinedSet-hasMember|R-HSA-9700564-crizotinib resistant ALK mutants [plasma membrane]-DefinedSet-hasMember|R-HSA-9700553-ceritinib-resistant ALK mutants [plasma membrane]-DefinedSet-hasMember|R-HSA-9700082-kinase domain ALK mutant dimers [plasma membrane]-CandidateSet-hasMember|R-HSA-9700083-kinase domain ALK mutants [plasma membrane]-CandidateSet-hasMember R-HSA-9700131|R-HSA-9700656|R-HSA-9715356|R-HSA-9715355|R-HSA-9715357|R-HSA-9700658|R-HSA-9700186|R-HSA-9700184 ALK mutants bind type I TKIs|alectinib resistant ALK mutants don't bind alectinib|brigatinib-resistant ALK mutants don't bind brigatinib|lorlatinib-resistant ALK mutants don't bind lorlatinib|crizotinib-resistant ALK mutants don't bind crizotinib|ceritinib-resistant ALK mutants don't bind ceritinib|Autophosphorylation of point mutants of ALK|Ligand-independent dimerization of point mutants of ALK loss_of_function pubmed:24613930|pubmed:21494621|pubmed:21948233|pubmed:28404650|pubmed:22570254|pubmed:26301689|pubmed:24496003|pubmed:27245569|pubmed:26939704|pubmed:25393796|pubmed:25421750|pubmed:25228534|pubmed:24675041|pubmed:26464158|pubmed:18923525|pubmed:26001147|pubmed:23239810|pubmed:24518094|pubmed:17185414|pubmed:31388026|pubmed:21502504|pubmed:24509625|pubmed:31374369|pubmed:25393798|pubmed:27432227|pubmed:27130468|pubmed:27009859|pubmed:31668326|pubmed:31943796|pubmed:31616196|pubmed:29650534|pubmed:31749991|pubmed:31712133|pubmed:22277784|pubmed:30089600|pubmed:27045755|pubmed:20979473|pubmed:21030459|pubmed:23434628|pubmed:21791641|pubmed:30675302|pubmed:22235099|pubmed:24736079|pubmed:23344087|pubmed:30683630|pubmed:18724359|pubmed:29455642|pubmed:24060861|pubmed:21847362|pubmed:18923524|pubmed:18923523|pubmed:21596819|pubmed:21838707|pubmed:27573755|pubmed:23104988|pubmed:23201355 R-HSA-9700645|R-HSA-9717316|R-HSA-9717319|R-HSA-9717329|R-HSA-9717326|R-HSA-9717323|R-HSA-9725370 ALK mutants bind TKIs|alectinib-resistant ALK mutants|brigatinib-resistant ALK mutants|lorlatinib-resistant ALK mutants|crizotinib-resistant ALK mutants|ceritinib-resistant ALK mutants|Signaling by ALK fusions and activated point mutants R-HSA-9700662|R-HSA-201521|R-HSA-201519 (PTN/MDK):ALK dimer binds type I ALK-binding TKIs|ALK autophosphorylation downstream of PTN and MDK|ALK:PTN dimerization Signaling by ALK R-HSA-201556 missense NA

PS3-PS1 ALK ALK R1275Q [plasma membrane] R-HSA-9699853 ALK UniProt:Q9UM73 L-arginine 1275 replaced with L-glutamine ReplacedResidue COSMIC:COSM88797|COSMIC:COSV66555567 neuroblastoma DOID:769 pubmed:18724359|pubmed:18923525|pubmed:20632993 R-HSA-9700081-NVP-TAE684-sensitive ALK mutants [cytosol]-DefinedSet-hasMember|R-HSA-9700082-kinase domain ALK mutant dimers [plasma membrane]-CandidateSet-hasMember|R-HSA-9700083-kinase domain ALK mutants [plasma membrane]-CandidateSet-hasMember R-HSA-9700131|R-HSA-9700186|R-HSA-9700184 ALK mutants bind type I TKIs|Autophosphorylation of point mutants of ALK|Ligand-independent dimerization of point mutants of ALK pubmed:24613930|pubmed:21494621|pubmed:21948233|pubmed:28404650|pubmed:22570254|pubmed:26301689|pubmed:24496003|pubmed:27245569|pubmed:26939704|pubmed:25393796|pubmed:25421750|pubmed:25228534|pubmed:24675041|pubmed:26464158|pubmed:18923525|pubmed:26001147|pubmed:23239810|pubmed:24518094|pubmed:17185414|pubmed:31388026|pubmed:21502504|pubmed:18724359|pubmed:29455642|pubmed:24060861|pubmed:21847362|pubmed:18923524|pubmed:18923523|pubmed:21596819|pubmed:21838707|pubmed:27573755|pubmed:23104988|pubmed:23201355 R-HSA-9700645|R-HSA-9725370 ALK mutants bind TKIs|Signaling by ALK fusions and activated point mutants R-HSA-9700662|R-HSA-201521|R-HSA-201519 (PTN/MDK):ALK dimer binds type I ALK-binding TKIs|ALK autophosphorylation downstream of PTN and MDK|ALK:PTN dimerization Signaling by ALK R-HSA-201556 missense NA

PS3-PS1 ALK ALK L1198F [plasma membrane] R-HSA-9701434 ALK UniProt:Q9UM73 L-leucine 1198 replaced with L-phenylalanine ReplacedResidue COSMIC:COSV66556391|COSMIC:COSM9311666 thyroid carcinoma|lung squamous cell carcinoma DOID:3963|DOID:3907 pubmed:21596819|pubmed:24675041 R-HSA-9700529-crizotinib-sensitive ALK mutants [cytosol]-DefinedSet-hasMember|R-HSA-9700560-alectinib resistant ALK mutants [plasma membrane]-DefinedSet-hasMember|R-HSA-9714046-brigatinib-resistant ALK mutants [plasma membrane]-DefinedSet-hasMember|R-HSA-9714053-lorlatinib-resistant ALK mutants [plasma membrane]-DefinedSet-hasMember|R-HSA-9700553-ceritinib-resistant ALK mutants [plasma membrane]-DefinedSet-hasMember|R-HSA-9700082-kinase domain ALK mutant dimers [plasma membrane]-CandidateSet-hasMember|R-HSA-9700083-kinase domain ALK mutants [plasma membrane]-CandidateSet-hasMember R-HSA-9700131|R-HSA-9700656|R-HSA-9715356|R-HSA-9715355|R-HSA-9700658|R-HSA-9700186|R-HSA-9700184 ALK mutants bind type I TKIs|alectinib resistant ALK mutants don't bind alectinib|brigatinib-resistant ALK mutants don't bind brigatinib|lorlatinib-resistant ALK mutants don't bind lorlatinib|ceritinib-resistant ALK mutants don't bind ceritinib|Autophosphorylation of point mutants of ALK|Ligand-independent dimerization of point mutants of ALK loss_of_function pubmed:24613930|pubmed:21494621|pubmed:21948233|pubmed:28404650|pubmed:22570254|pubmed:26301689|pubmed:24496003|pubmed:27245569|pubmed:26939704|pubmed:25393796|pubmed:25421750|pubmed:25228534|pubmed:24675041|pubmed:26464158|pubmed:18923525|pubmed:26001147|pubmed:23239810|pubmed:24518094|pubmed:17185414|pubmed:31388026|pubmed:21502504|pubmed:24509625|pubmed:31374369|pubmed:25393798|pubmed:27432227|pubmed:27130468|pubmed:27009859|pubmed:31668326|pubmed:31943796|pubmed:31616196|pubmed:29650534|pubmed:31749991|pubmed:30683630|pubmed:18724359|pubmed:29455642|pubmed:24060861|pubmed:21847362|pubmed:18923524|pubmed:18923523|pubmed:21596819|pubmed:21838707|pubmed:27573755|pubmed:23104988|pubmed:23201355 R-HSA-9700645|R-HSA-9717316|R-HSA-9717319|R-HSA-9717329|R-HSA-9717323|R-HSA-9725370 ALK mutants bind TKIs|alectinib-resistant ALK mutants|brigatinib-resistant ALK mutants|lorlatinib-resistant ALK mutants|ceritinib-resistant ALK mutants|Signaling by ALK fusions and activated point mutants R-HSA-9700662|R-HSA-201521|R-HSA-201519 (PTN/MDK):ALK dimer binds type I ALK-binding TKIs|ALK autophosphorylation downstream of PTN and MDK|ALK:PTN dimerization Signaling by ALK R-HSA-201556 missense NA

PS3 ALK ALK S1206Y [plasma membrane] R-HSA-9701426 ALK UniProt:Q9UM73 L-serine 1206 replaced with L-tyrosine ReplacedResidue COSMIC:COSV66566818 lung adenocarcinoma DOID:3910 pubmed:24675041 R-HSA-9700528-ceritinib-sensitive ALK mutants [plasma membrane]-DefinedSet-hasMember|R-HSA-9700564-crizotinib resistant ALK mutants [plasma membrane]-DefinedSet-hasMember R-HSA-9700131|R-HSA-9715357 ALK mutants bind type I TKIs|crizotinib-resistant ALK mutants don't bind crizotinib loss_of_function pubmed:24613930|pubmed:21494621|pubmed:21948233|pubmed:28404650|pubmed:22570254|pubmed:26301689|pubmed:24496003|pubmed:27245569|pubmed:26939704|pubmed:25393796|pubmed:25421750|pubmed:25228534|pubmed:24675041|pubmed:26464158|pubmed:18923525|pubmed:26001147|pubmed:23239810|pubmed:24518094|pubmed:17185414|pubmed:31388026|pubmed:21502504|pubmed:31712133|pubmed:25393798|pubmed:22277784|pubmed:30089600|pubmed:27045755|pubmed:20979473|pubmed:21030459|pubmed:27432227|pubmed:23434628|pubmed:21791641|pubmed:30675302|pubmed:22235099|pubmed:24736079|pubmed:23344087|pubmed:31749991 R-HSA-9700645|R-HSA-9717326 ALK mutants bind TKIs|crizotinib-resistant ALK mutants R-HSA-9700662 (PTN/MDK):ALK dimer binds type I ALK-binding TKIs Signaling by ALK R-HSA-201556 missense NA

PS3-PS1 ALK ALK I1171T [plasma membrane] R-HSA-9699891 ALK UniProt:Q9UM73 L-isoleucine 1171 replaced with L-threonine ReplacedResidue COSMIC:COSV66564577|COSMIC:COSM9306547 non-small cell lung carcinoma|lung adenocarcinoma DOID:3908|DOID:3910 R-HSA-9700528-ceritinib-sensitive ALK mutants [plasma membrane]-DefinedSet-hasMember|R-HSA-9700560-alectinib resistant ALK mutants [plasma membrane]-DefinedSet-hasMember|R-HSA-9714046-brigatinib-resistant ALK mutants [plasma membrane]-DefinedSet-hasMember|R-HSA-9717270-ASP3026-resistant ALK mutants [plasma membrane]-DefinedSet-hasMember|R-HSA-9700564-crizotinib resistant ALK mutants [plasma membrane]-DefinedSet-hasMember|R-HSA-9700082-kinase domain ALK mutant dimers [plasma membrane]-CandidateSet-hasCandidate|R-HSA-9700083-kinase domain ALK mutants [plasma membrane]-CandidateSet-hasCandidate R-HSA-9700131|R-HSA-9700656|R-HSA-9715356|R-HSA-9700646|R-HSA-9715357|R-HSA-9700186|R-HSA-9700184 ALK mutants bind type I TKIs|alectinib resistant ALK mutants don't bind alectinib|brigatinib-resistant ALK mutants don't bind brigatinib|ASP-3026- resistant ALK mutants don't bind ASP-3026|crizotinib-resistant ALK mutants don't bind crizotinib|Autophosphorylation of point mutants of ALK|Ligand-independent dimerization of point mutants of ALK loss_of_function pubmed:24613930|pubmed:21494621|pubmed:21948233|pubmed:28404650|pubmed:22570254|pubmed:26301689|pubmed:24496003|pubmed:27245569|pubmed:26939704|pubmed:25393796|pubmed:25421750|pubmed:25228534|pubmed:24675041|pubmed:26464158|pubmed:18923525|pubmed:26001147|pubmed:23239810|pubmed:24518094|pubmed:17185414|pubmed:31388026|pubmed:21502504|pubmed:24509625|pubmed:31374369|pubmed:25393798|pubmed:27432227|pubmed:27130468|pubmed:27009859|pubmed:31668326|pubmed:31712133|pubmed:22277784|pubmed:30089600|pubmed:27045755|pubmed:20979473|pubmed:21030459|pubmed:23434628|pubmed:21791641|pubmed:30675302|pubmed:22235099|pubmed:24736079|pubmed:23344087|pubmed:31749991|pubmed:18724359|pubmed:29455642|pubmed:24060861|pubmed:21847362|pubmed:18923524|pubmed:18923523|pubmed:21596819|pubmed:21838707|pubmed:27573755|pubmed:23104988|pubmed:23201355 R-HSA-9700645|R-HSA-9717316|R-HSA-9717319|R-HSA-9717264|R-HSA-9717326|R-HSA-9725370 ALK mutants bind TKIs|alectinib-resistant ALK mutants|brigatinib-resistant ALK mutants|ASP-3026-resistant ALK mutants|crizotinib-resistant ALK mutants|Signaling by ALK fusions and activated point mutants R-HSA-9700662|R-HSA-201521|R-HSA-201519 (PTN/MDK):ALK dimer binds type I ALK-binding TKIs|ALK autophosphorylation downstream of PTN and MDK|ALK:PTN dimerization Signaling by ALK R-HSA-201556 missense NA

PS3-PS1 ALK ALK G1269A [plasma membrane] R-HSA-9701427 ALK UniProt:Q9UM73 glycine 1269 replaced with L-alanine ReplacedResidue COSMIC:COSM4422787|COSMIC:COSV66557991 lung squamous cell carcinoma DOID:3907 pubmed:24675041 R-HSA-9700528-ceritinib-sensitive ALK mutants [plasma membrane]-DefinedSet-hasMember|R-HSA-9717270-ASP3026-resistant ALK mutants [plasma membrane]-DefinedSet-hasMember|R-HSA-9714053-lorlatinib-resistant ALK mutants [plasma membrane]-DefinedSet-hasMember|R-HSA-9700564-crizotinib resistant ALK mutants [plasma membrane]-DefinedSet-hasMember|R-HSA-9700553-ceritinib-resistant ALK mutants [plasma membrane]-DefinedSet-hasMember R-HSA-9700131|R-HSA-9700646|R-HSA-9715355|R-HSA-9715357|R-HSA-9700658 ALK mutants bind type I TKIs|ASP-3026- resistant ALK mutants don't bind ASP-3026|lorlatinib-resistant ALK mutants don't bind lorlatinib|crizotinib-resistant ALK mutants don't bind crizotinib|ceritinib-resistant ALK mutants don't bind ceritinib loss_of_function pubmed:24613930|pubmed:21494621|pubmed:21948233|pubmed:28404650|pubmed:22570254|pubmed:26301689|pubmed:24496003|pubmed:27245569|pubmed:26939704|pubmed:25393796|pubmed:25421750|pubmed:25228534|pubmed:24675041|pubmed:26464158|pubmed:18923525|pubmed:26001147|pubmed:23239810|pubmed:24518094|pubmed:17185414|pubmed:31388026|pubmed:21502504|pubmed:27009859|pubmed:31943796|pubmed:31616196|pubmed:29650534|pubmed:31749991|pubmed:31712133|pubmed:25393798|pubmed:22277784|pubmed:30089600|pubmed:27045755|pubmed:20979473|pubmed:21030459|pubmed:27432227|pubmed:23434628|pubmed:21791641|pubmed:30675302|pubmed:22235099|pubmed:24736079|pubmed:23344087|pubmed:30683630 R-HSA-9700645|R-HSA-9717264|R-HSA-9717329|R-HSA-9717326|R-HSA-9717323 ALK mutants bind TKIs|ASP-3026-resistant ALK mutants|lorlatinib-resistant ALK mutants|crizotinib-resistant ALK mutants|ceritinib-resistant ALK mutants R-HSA-9700662 (PTN/MDK):ALK dimer binds type I ALK-binding TKIs Signaling by ALK R-HSA-201556 missense NA

PS3-PS1 ALK ALK G1202R [plasma membrane] R-HSA-9701428 ALK UniProt:Q9UM73 glycine 1202 replaced with L-arginine ReplacedResidue COSMIC:COSV66555808|COSMIC:COSV104692577|COSMIC:COSM4422789 lung squamous cell carcinoma DOID:3907 R-HSA-9700560-alectinib resistant ALK mutants [plasma membrane]-DefinedSet-hasMember|R-HSA-9714046-brigatinib-resistant ALK mutants [plasma membrane]-DefinedSet-hasMember|R-HSA-9714053-lorlatinib-resistant ALK mutants [plasma membrane]-DefinedSet-hasMember|R-HSA-9700564-crizotinib resistant ALK mutants [plasma membrane]-DefinedSet-hasMember|R-HSA-9700553-ceritinib-resistant ALK mutants [plasma membrane]-DefinedSet-hasMember R-HSA-9700656|R-HSA-9715356|R-HSA-9715355|R-HSA-9715357|R-HSA-9700658 alectinib resistant ALK mutants don't bind alectinib|brigatinib-resistant ALK mutants don't bind brigatinib|lorlatinib-resistant ALK mutants don't bind lorlatinib|crizotinib-resistant ALK mutants don't bind crizotinib|ceritinib-resistant ALK mutants don't bind ceritinib loss_of_function pubmed:25228534|pubmed:24509625|pubmed:31374369|pubmed:26464158|pubmed:25393798|pubmed:27432227|pubmed:25421750|pubmed:25393796|pubmed:27130468|pubmed:27009859|pubmed:23239810|pubmed:31668326|pubmed:31943796|pubmed:31616196|pubmed:29650534|pubmed:31749991|pubmed:31712133|pubmed:21948233|pubmed:22277784|pubmed:30089600|pubmed:27045755|pubmed:20979473|pubmed:24675041|pubmed:21030459|pubmed:23434628|pubmed:21791641|pubmed:30675302|pubmed:21502504|pubmed:22235099|pubmed:24736079|pubmed:23344087|pubmed:30683630 R-HSA-9717316|R-HSA-9717319|R-HSA-9717329|R-HSA-9717326|R-HSA-9717323 alectinib-resistant ALK mutants|brigatinib-resistant ALK mutants|lorlatinib-resistant ALK mutants|crizotinib-resistant ALK mutants|ceritinib-resistant ALK mutants R-HSA-9700662 (PTN/MDK):ALK dimer binds type I ALK-binding TKIs Signaling by ALK R-HSA-201556 missense NA

PS3-PS1 ALK ALK I1171S [plasma membrane] R-HSA-9714054 ALK UniProt:Q9UM73 L-isoleucine 1171 replaced with L-serine ReplacedResidue COSMIC:COSV66567934|COSMIC:COSM5352215 non-small cell lung carcinoma DOID:3908 pubmed:31943796 R-HSA-9700560-alectinib resistant ALK mutants [plasma membrane]-DefinedSet-hasMember|R-HSA-9714046-brigatinib-resistant ALK mutants [plasma membrane]-DefinedSet-hasMember|R-HSA-9717270-ASP3026-resistant ALK mutants [plasma membrane]-DefinedSet-hasMember|R-HSA-9714053-lorlatinib-resistant ALK mutants [plasma membrane]-DefinedSet-hasMember|R-HSA-9700564-crizotinib resistant ALK mutants [plasma membrane]-DefinedSet-hasMember|R-HSA-9700553-ceritinib-resistant ALK mutants [plasma membrane]-DefinedSet-hasMember|R-HSA-9700082-kinase domain ALK mutant dimers [plasma membrane]-CandidateSet-hasCandidate|R-HSA-9700083-kinase domain ALK mutants [plasma membrane]-CandidateSet-hasCandidate R-HSA-9700656|R-HSA-9715356|R-HSA-9700646|R-HSA-9715355|R-HSA-9715357|R-HSA-9700658|R-HSA-9700186|R-HSA-9700184 alectinib resistant ALK mutants don't bind alectinib|brigatinib-resistant ALK mutants don't bind brigatinib|ASP-3026- resistant ALK mutants don't bind ASP-3026|lorlatinib-resistant ALK mutants don't bind lorlatinib|crizotinib-resistant ALK mutants don't bind crizotinib|ceritinib-resistant ALK mutants don't bind ceritinib|Autophosphorylation of point mutants of ALK|Ligand-independent dimerization of point mutants of ALK loss_of_function pubmed:25228534|pubmed:24509625|pubmed:31374369|pubmed:26464158|pubmed:25393798|pubmed:27432227|pubmed:25421750|pubmed:25393796|pubmed:27130468|pubmed:27009859|pubmed:23239810|pubmed:31668326|pubmed:31943796|pubmed:31616196|pubmed:29650534|pubmed:31749991|pubmed:31712133|pubmed:21948233|pubmed:22277784|pubmed:30089600|pubmed:27045755|pubmed:20979473|pubmed:24675041|pubmed:21030459|pubmed:23434628|pubmed:21791641|pubmed:30675302|pubmed:21502504|pubmed:22235099|pubmed:24736079|pubmed:23344087|pubmed:30683630|pubmed:18724359|pubmed:29455642|pubmed:18923525|pubmed:24060861|pubmed:21847362|pubmed:18923524|pubmed:18923523|pubmed:21596819|pubmed:21838707|pubmed:27573755|pubmed:23104988|pubmed:23201355 R-HSA-9717316|R-HSA-9717319|R-HSA-9717264|R-HSA-9717329|R-HSA-9717326|R-HSA-9717323|R-HSA-9725370 alectinib-resistant ALK mutants|brigatinib-resistant ALK mutants|ASP-3026-resistant ALK mutants|lorlatinib-resistant ALK mutants|crizotinib-resistant ALK mutants|ceritinib-resistant ALK mutants|Signaling by ALK fusions and activated point mutants R-HSA-9700662|R-HSA-201521|R-HSA-201519 (PTN/MDK):ALK dimer binds type I ALK-binding TKIs|ALK autophosphorylation downstream of PTN and MDK|ALK:PTN dimerization Signaling by ALK R-HSA-201556 missense NA

PS3 ALK ALK S1206C [plasma membrane] R-HSA-9715268 ALK UniProt:Q9UM73 L-serine 1206 replaced with L-cysteine ReplacedResidue COSMIC:COSM9311656 non-small cell lung carcinoma DOID:3908 pubmed:27432227|pubmed:25421750 R-HSA-9700560-alectinib resistant ALK mutants [plasma membrane]-DefinedSet-hasMember|R-HSA-9700564-crizotinib resistant ALK mutants [plasma membrane]-DefinedSet-hasMember|R-HSA-9700553-ceritinib-resistant ALK mutants [plasma membrane]-DefinedSet-hasMember|R-HSA-9700082-kinase domain ALK mutant dimers [plasma membrane]-CandidateSet-hasMember|R-HSA-9700083-kinase domain ALK mutants [plasma membrane]-CandidateSet-hasMember R-HSA-9700656|R-HSA-9715357|R-HSA-9700658|R-HSA-9700186|R-HSA-9700184 alectinib resistant ALK mutants don't bind alectinib|crizotinib-resistant ALK mutants don't bind crizotinib|ceritinib-resistant ALK mutants don't bind ceritinib|Autophosphorylation of point mutants of ALK|Ligand-independent dimerization of point mutants of ALK loss_of_function pubmed:25228534|pubmed:24509625|pubmed:31374369|pubmed:26464158|pubmed:25393798|pubmed:27432227|pubmed:25421750|pubmed:25393796|pubmed:27130468|pubmed:31712133|pubmed:21948233|pubmed:22277784|pubmed:30089600|pubmed:27045755|pubmed:20979473|pubmed:24675041|pubmed:21030459|pubmed:23434628|pubmed:21791641|pubmed:23239810|pubmed:30675302|pubmed:21502504|pubmed:22235099|pubmed:24736079|pubmed:23344087|pubmed:31749991|pubmed:30683630|pubmed:27009859|pubmed:18724359|pubmed:29455642|pubmed:18923525|pubmed:24060861|pubmed:21847362|pubmed:18923524|pubmed:18923523|pubmed:21596819|pubmed:21838707|pubmed:27573755|pubmed:23104988|pubmed:23201355 R-HSA-9717316|R-HSA-9717326|R-HSA-9717323|R-HSA-9725370 alectinib-resistant ALK mutants|crizotinib-resistant ALK mutants|ceritinib-resistant ALK mutants|Signaling by ALK fusions and activated point mutants R-HSA-9700662|R-HSA-201521|R-HSA-201519 (PTN/MDK):ALK dimer binds type I ALK-binding TKIs|ALK autophosphorylation downstream of PTN and MDK|ALK:PTN dimerization Signaling by ALK R-HSA-201556 missense NA

PS3-PS1 ALK ALK F1174C [plasma membrane] R-HSA-9699908 ALK UniProt:Q9UM73 L-phenylalanine 1174 replaced with L-cysteine ReplacedResidue COSMIC:COSV66560921|COSMIC:COSM4422790 non-small cell lung carcinoma|neuroblastoma DOID:3908|DOID:769 R-HSA-9700560-alectinib resistant ALK mutants [plasma membrane]-DefinedSet-hasMember|R-HSA-9700564-crizotinib resistant ALK mutants [plasma membrane]-DefinedSet-hasMember|R-HSA-9700553-ceritinib-resistant ALK mutants [plasma membrane]-DefinedSet-hasMember|R-HSA-9700082-kinase domain ALK mutant dimers [plasma membrane]-CandidateSet-hasCandidate|R-HSA-9700083-kinase domain ALK mutants [plasma membrane]-CandidateSet-hasCandidate R-HSA-9700656|R-HSA-9715357|R-HSA-9700658|R-HSA-9700186|R-HSA-9700184 alectinib resistant ALK mutants don't bind alectinib|crizotinib-resistant ALK mutants don't bind crizotinib|ceritinib-resistant ALK mutants don't bind ceritinib|Autophosphorylation of point mutants of ALK|Ligand-independent dimerization of point mutants of ALK loss_of_function pubmed:25228534|pubmed:24509625|pubmed:31374369|pubmed:26464158|pubmed:25393798|pubmed:27432227|pubmed:25421750|pubmed:25393796|pubmed:27130468|pubmed:31712133|pubmed:21948233|pubmed:22277784|pubmed:30089600|pubmed:27045755|pubmed:20979473|pubmed:24675041|pubmed:21030459|pubmed:23434628|pubmed:21791641|pubmed:23239810|pubmed:30675302|pubmed:21502504|pubmed:22235099|pubmed:24736079|pubmed:23344087|pubmed:31749991|pubmed:30683630|pubmed:27009859|pubmed:18724359|pubmed:29455642|pubmed:18923525|pubmed:24060861|pubmed:21847362|pubmed:18923524|pubmed:18923523|pubmed:21596819|pubmed:21838707|pubmed:27573755|pubmed:23104988|pubmed:23201355 R-HSA-9717316|R-HSA-9717326|R-HSA-9717323|R-HSA-9725370 alectinib-resistant ALK mutants|crizotinib-resistant ALK mutants|ceritinib-resistant ALK mutants|Signaling by ALK fusions and activated point mutants R-HSA-9700662|R-HSA-201521|R-HSA-201519 (PTN/MDK):ALK dimer binds type I ALK-binding TKIs|ALK autophosphorylation downstream of PTN and MDK|ALK:PTN dimerization Signaling by ALK R-HSA-201556 missense NA

PS3-PS1 ALK ALK I1171N [plasma membrane] R-HSA-9699828 ALK UniProt:Q9UM73 L-isoleucine 1171 replaced with L-asparagine ReplacedResidue COSMIC:COSV66556242|COSMIC:COSM9278044 non-small cell lung carcinoma|lung adenocarcinoma|neuroblastoma DOID:3908|DOID:3910|DOID:769 pubmed:23239810 R-HSA-9700560-alectinib resistant ALK mutants [plasma membrane]-DefinedSet-hasMember|R-HSA-9700568-NVP-TAE684-resistant ALK mutants [plasma membrane]-DefinedSet-hasMember|R-HSA-9714046-brigatinib-resistant ALK mutants [plasma membrane]-DefinedSet-hasMember|R-HSA-9714053-lorlatinib-resistant ALK mutants [plasma membrane]-DefinedSet-hasMember|R-HSA-9700564-crizotinib resistant ALK mutants [plasma membrane]-DefinedSet-hasMember|R-HSA-9700082-kinase domain ALK mutant dimers [plasma membrane]-CandidateSet-hasMember|R-HSA-9700083-kinase domain ALK mutants [plasma membrane]-CandidateSet-hasMember R-HSA-9700656|R-HSA-9700114|R-HSA-9715356|R-HSA-9715355|R-HSA-9715357|R-HSA-9700186|R-HSA-9700184 alectinib resistant ALK mutants don't bind alectinib|NVP-TAE684-resistant ALK mutants don't bind NVP-TAE684|brigatinib-resistant ALK mutants don't bind brigatinib|lorlatinib-resistant ALK mutants don't bind lorlatinib|crizotinib-resistant ALK mutants don't bind crizotinib|Autophosphorylation of point mutants of ALK|Ligand-independent dimerization of point mutants of ALK loss_of_function pubmed:25228534|pubmed:24509625|pubmed:31374369|pubmed:26464158|pubmed:25393798|pubmed:27432227|pubmed:25421750|pubmed:25393796|pubmed:27130468|pubmed:21948233|pubmed:18923525|pubmed:23239810|pubmed:21791641|pubmed:27009859|pubmed:31668326|pubmed:31943796|pubmed:31616196|pubmed:29650534|pubmed:31749991|pubmed:31712133|pubmed:22277784|pubmed:30089600|pubmed:27045755|pubmed:20979473|pubmed:24675041|pubmed:21030459|pubmed:23434628|pubmed:30675302|pubmed:21502504|pubmed:22235099|pubmed:24736079|pubmed:23344087|pubmed:18724359|pubmed:29455642|pubmed:24060861|pubmed:21847362|pubmed:18923524|pubmed:18923523|pubmed:21596819|pubmed:21838707|pubmed:27573755|pubmed:23104988|pubmed:23201355 R-HSA-9717316|R-HSA-9717301|R-HSA-9717319|R-HSA-9717329|R-HSA-9717326|R-HSA-9725370 alectinib-resistant ALK mutants|NVP-TAE684-resistant ALK mutants|brigatinib-resistant ALK mutants|lorlatinib-resistant ALK mutants|crizotinib-resistant ALK mutants|Signaling by ALK fusions and activated point mutants R-HSA-9700662|R-HSA-201521|R-HSA-201519 (PTN/MDK):ALK dimer binds type I ALK-binding TKIs|ALK autophosphorylation downstream of PTN and MDK|ALK:PTN dimerization Signaling by ALK R-HSA-201556 missense NA

PS3 ALK ALK L1152R [plasma membrane] R-HSA-9701836 ALK UniProt:Q9UM73 L-leucine 1152 replaced with L-arginine ReplacedResidue COSMIC:COSM9413102 non-small cell lung carcinoma|lung adenocarcinoma DOID:3908|DOID:3910 R-HSA-9700568-NVP-TAE684-resistant ALK mutants [plasma membrane]-DefinedSet-hasMember|R-HSA-9700564-crizotinib resistant ALK mutants [plasma membrane]-DefinedSet-hasMember R-HSA-9700114|R-HSA-9715357 NVP-TAE684-resistant ALK mutants don't bind NVP-TAE684|crizotinib-resistant ALK mutants don't bind crizotinib loss_of_function pubmed:21948233|pubmed:18923525|pubmed:23239810|pubmed:21791641|pubmed:31712133|pubmed:25393798|pubmed:22277784|pubmed:30089600|pubmed:27045755|pubmed:20979473|pubmed:24675041|pubmed:21030459|pubmed:27432227|pubmed:23434628|pubmed:30675302|pubmed:21502504|pubmed:22235099|pubmed:24736079|pubmed:23344087|pubmed:31749991 R-HSA-9717301|R-HSA-9717326 NVP-TAE684-resistant ALK mutants|crizotinib-resistant ALK mutants R-HSA-9700662 (PTN/MDK):ALK dimer binds type I ALK-binding TKIs Signaling by ALK R-HSA-201556 missense NA

PS3 ALK ALK A1234T [plasma membrane] R-HSA-9699831 ALK UniProt:Q9UM73 L-alanine 1234 replaced with L-threonine ReplacedResidue COSMIC:COSV66574878 neuroblastoma|lung squamous cell carcinoma DOID:769|DOID:3907 pubmed:18923525 R-HSA-9700568-NVP-TAE684-resistant ALK mutants [plasma membrane]-DefinedSet-hasMember R-HSA-9700114 NVP-TAE684-resistant ALK mutants don't bind NVP-TAE684 loss_of_function pubmed:21948233|pubmed:18923525|pubmed:23239810|pubmed:21791641 R-HSA-9717301 NVP-TAE684-resistant ALK mutants R-HSA-9700662 (PTN/MDK):ALK dimer binds type I ALK-binding TKIs Signaling by ALK R-HSA-201556 missense NA

PS3-PS1 ALK ALK D1203N [plasma membrane] R-HSA-9701838 ALK UniProt:Q9UM73 L-aspartic acid 1203 replaced with L-asparagine ReplacedResidue COSMIC:COSM9306550|COSMIC:COSV66565234 non-small cell lung carcinoma|lung adenocarcinoma DOID:3908|DOID:3910 pubmed:21596819 R-HSA-9700568-NVP-TAE684-resistant ALK mutants [plasma membrane]-DefinedSet-hasMember|R-HSA-9714046-brigatinib-resistant ALK mutants [plasma membrane]-DefinedSet-hasMember|R-HSA-9700564-crizotinib resistant ALK mutants [plasma membrane]-DefinedSet-hasMember|R-HSA-9700553-ceritinib-resistant ALK mutants [plasma membrane]-DefinedSet-hasMember R-HSA-9700114|R-HSA-9715356|R-HSA-9715357|R-HSA-9700658 NVP-TAE684-resistant ALK mutants don't bind NVP-TAE684|brigatinib-resistant ALK mutants don't bind brigatinib|crizotinib-resistant ALK mutants don't bind crizotinib|ceritinib-resistant ALK mutants don't bind ceritinib loss_of_function pubmed:21948233|pubmed:18923525|pubmed:23239810|pubmed:21791641|pubmed:25228534|pubmed:27432227|pubmed:27009859|pubmed:31668326|pubmed:25421750|pubmed:31712133|pubmed:25393798|pubmed:22277784|pubmed:30089600|pubmed:27045755|pubmed:20979473|pubmed:24675041|pubmed:21030459|pubmed:23434628|pubmed:30675302|pubmed:21502504|pubmed:22235099|pubmed:24736079|pubmed:23344087|pubmed:31749991|pubmed:30683630 R-HSA-9717301|R-HSA-9717319|R-HSA-9717326|R-HSA-9717323 NVP-TAE684-resistant ALK mutants|brigatinib-resistant ALK mutants|crizotinib-resistant ALK mutants|ceritinib-resistant ALK mutants R-HSA-9700662 (PTN/MDK):ALK dimer binds type I ALK-binding TKIs Signaling by ALK R-HSA-201556 missense NA

PS3 ALK ALK L1122V [plasma membrane] R-HSA-9715269 ALK UniProt:Q9UM73 L-leucine 1122 replaced with L-valine ReplacedResidue COSMIC:COSM9311665 non-small cell lung carcinoma DOID:3908 pubmed:29650534|pubmed:25421750 R-HSA-9714046-brigatinib-resistant ALK mutants [plasma membrane]-DefinedSet-hasMember|R-HSA-9700564-crizotinib resistant ALK mutants [plasma membrane]-DefinedSet-hasMember|R-HSA-9700553-ceritinib-resistant ALK mutants [plasma membrane]-DefinedSet-hasMember|R-HSA-9700082-kinase domain ALK mutant dimers [plasma membrane]-CandidateSet-hasMember|R-HSA-9700083-kinase domain ALK mutants [plasma membrane]-CandidateSet-hasMember R-HSA-9715356|R-HSA-9715357|R-HSA-9700658|R-HSA-9700186|R-HSA-9700184 brigatinib-resistant ALK mutants don't bind brigatinib|crizotinib-resistant ALK mutants don't bind crizotinib|ceritinib-resistant ALK mutants don't bind ceritinib|Autophosphorylation of point mutants of ALK|Ligand-independent dimerization of point mutants of ALK loss_of_function pubmed:25228534|pubmed:27432227|pubmed:27009859|pubmed:23239810|pubmed:31668326|pubmed:25421750|pubmed:31712133|pubmed:25393798|pubmed:21948233|pubmed:22277784|pubmed:30089600|pubmed:27045755|pubmed:20979473|pubmed:24675041|pubmed:21030459|pubmed:23434628|pubmed:21791641|pubmed:30675302|pubmed:21502504|pubmed:22235099|pubmed:24736079|pubmed:23344087|pubmed:31749991|pubmed:30683630|pubmed:18724359|pubmed:29455642|pubmed:18923525|pubmed:24060861|pubmed:21847362|pubmed:18923524|pubmed:18923523|pubmed:21596819|pubmed:21838707|pubmed:27573755|pubmed:23104988|pubmed:23201355 R-HSA-9717319|R-HSA-9717326|R-HSA-9717323|R-HSA-9725370 brigatinib-resistant ALK mutants|crizotinib-resistant ALK mutants|ceritinib-resistant ALK mutants|Signaling by ALK fusions and activated point mutants R-HSA-9700662|R-HSA-201521|R-HSA-201519 (PTN/MDK):ALK dimer binds type I ALK-binding TKIs|ALK autophosphorylation downstream of PTN and MDK|ALK:PTN dimerization Signaling by ALK R-HSA-201556 missense NA

PS3 ALK ALK R1192P [plasma membrane] R-HSA-9699802 ALK UniProt:Q9UM73 L-arginine 1192 replaced with L-proline ReplacedResidue COSMIC:COSM7340824 lung adenocarcinoma DOID:3910 R-HSA-9717270-ASP3026-resistant ALK mutants [plasma membrane]-DefinedSet-hasMember R-HSA-9700646 ASP-3026- resistant ALK mutants don't bind ASP-3026 loss_of_function pubmed:25228534|pubmed:27009859 R-HSA-9717264 ASP-3026-resistant ALK mutants R-HSA-9700662 (PTN/MDK):ALK dimer binds type I ALK-binding TKIs Signaling by ALK R-HSA-201556 missense NA

PS3-PS1 ALK ALK T1151M [plasma membrane] R-HSA-9699838 ALK UniProt:Q9UM73 L-threonine 1151 replaced with L-methionine ReplacedResidue COSMIC:COSM9413100|COSMIC:COSV66556401 non-small cell lung carcinoma|colon adenocarcinoma|neuroblastoma DOID:3908|DOID:234|DOID:769 R-HSA-9717270-ASP3026-resistant ALK mutants [plasma membrane]-DefinedSet-hasMember|R-HSA-9700564-crizotinib resistant ALK mutants [plasma membrane]-DefinedSet-hasMember R-HSA-9700646|R-HSA-9715357 ASP-3026- resistant ALK mutants don't bind ASP-3026|crizotinib-resistant ALK mutants don't bind crizotinib loss_of_function pubmed:25228534|pubmed:27009859|pubmed:31712133|pubmed:25393798|pubmed:21948233|pubmed:22277784|pubmed:30089600|pubmed:27045755|pubmed:20979473|pubmed:24675041|pubmed:21030459|pubmed:27432227|pubmed:23434628|pubmed:21791641|pubmed:23239810|pubmed:30675302|pubmed:21502504|pubmed:22235099|pubmed:24736079|pubmed:23344087|pubmed:31749991 R-HSA-9717264|R-HSA-9717326 ASP-3026-resistant ALK mutants|crizotinib-resistant ALK mutants R-HSA-9700662 (PTN/MDK):ALK dimer binds type I ALK-binding TKIs Signaling by ALK R-HSA-201556 missense NA

PS3 ALK ALK G1128A [plasma membrane] R-HSA-9699836 ALK UniProt:Q9UM73 glycine 1128 replaced with L-alanine ReplacedResidue COSMIC:COSV66582675 lung adenocarcinoma|neuroblastoma DOID:3910|DOID:769 pubmed:18724359|pubmed:20695522 R-HSA-9700564-crizotinib resistant ALK mutants [plasma membrane]-DefinedSet-hasMember|R-HSA-9700082-kinase domain ALK mutant dimers [plasma membrane]-CandidateSet-hasMember|R-HSA-9700083-kinase domain ALK mutants [plasma membrane]-CandidateSet-hasMember R-HSA-9715357|R-HSA-9700186|R-HSA-9700184 crizotinib-resistant ALK mutants don't bind crizotinib|Autophosphorylation of point mutants of ALK|Ligand-independent dimerization of point mutants of ALK loss_of_function pubmed:31712133|pubmed:25393798|pubmed:21948233|pubmed:22277784|pubmed:30089600|pubmed:27045755|pubmed:20979473|pubmed:24675041|pubmed:21030459|pubmed:27432227|pubmed:23434628|pubmed:21791641|pubmed:23239810|pubmed:30675302|pubmed:21502504|pubmed:22235099|pubmed:24736079|pubmed:23344087|pubmed:31749991|pubmed:18724359|pubmed:29455642|pubmed:18923525|pubmed:24060861|pubmed:21847362|pubmed:25421750|pubmed:18923524|pubmed:18923523|pubmed:21596819|pubmed:21838707|pubmed:27573755|pubmed:23104988|pubmed:23201355 R-HSA-9717326|R-HSA-9725370 crizotinib-resistant ALK mutants|Signaling by ALK fusions and activated point mutants R-HSA-9700662|R-HSA-201521|R-HSA-201519 (PTN/MDK):ALK dimer binds type I ALK-binding TKIs|ALK autophosphorylation downstream of PTN and MDK|ALK:PTN dimerization Signaling by ALK R-HSA-201556 missense NA

PS3-PS1 ALK ALK S1206F [plasma membrane] R-HSA-9701418 ALK UniProt:Q9UM73 L-serine 1206 replaced with L-phenylalanine ReplacedResidue COSMIC:COSM5985119|COSMIC:COSV66594791 non-small cell lung carcinoma|salivary gland cancer DOID:3908|DOID:8850 R-HSA-9700564-crizotinib resistant ALK mutants [plasma membrane]-DefinedSet-hasMember R-HSA-9715357 crizotinib-resistant ALK mutants don't bind crizotinib loss_of_function pubmed:31712133|pubmed:25393798|pubmed:21948233|pubmed:22277784|pubmed:30089600|pubmed:27045755|pubmed:20979473|pubmed:24675041|pubmed:21030459|pubmed:27432227|pubmed:23434628|pubmed:21791641|pubmed:23239810|pubmed:30675302|pubmed:21502504|pubmed:22235099|pubmed:24736079|pubmed:23344087|pubmed:31749991 R-HSA-9717326 crizotinib-resistant ALK mutants R-HSA-9700662 (PTN/MDK):ALK dimer binds type I ALK-binding TKIs Signaling by ALK R-HSA-201556 missense NA

PS3 ALK ALK T1151dup [plasma membrane] R-HSA-9701818 ALK UniProt:Q9UM73 Insertion of residues 1151 to 1151 at 1152 from UniProt:Q9UM73 ALK FragmentInsertionModification COSMIC:COSV66558491 non-small cell lung carcinoma DOID:3908 pubmed:22277784 R-HSA-9700564-crizotinib resistant ALK mutants [plasma membrane]-DefinedSet-hasMember R-HSA-9715357 crizotinib-resistant ALK mutants don't bind crizotinib loss_of_function pubmed:31712133|pubmed:25393798|pubmed:21948233|pubmed:22277784|pubmed:30089600|pubmed:27045755|pubmed:20979473|pubmed:24675041|pubmed:21030459|pubmed:27432227|pubmed:23434628|pubmed:21791641|pubmed:23239810|pubmed:30675302|pubmed:21502504|pubmed:22235099|pubmed:24736079|pubmed:23344087|pubmed:31749991 R-HSA-9717326 crizotinib-resistant ALK mutants R-HSA-9700662 (PTN/MDK):ALK dimer binds type I ALK-binding TKIs Signaling by ALK R-HSA-201556 in-frame indel: duplication NA

PS3-PS1 ALK ALK C1156Y [plasma membrane] R-HSA-9701433 ALK UniProt:Q9UM73 L-cysteine 1156 replaced with L-tyrosine ReplacedResidue COSMIC:COSV66562303|COSMIC:COSM9278966 lung adenocarcinoma|lung squamous cell carcinoma DOID:3910|DOID:3907 pubmed:24675041 R-HSA-9700564-crizotinib resistant ALK mutants [plasma membrane]-DefinedSet-hasMember R-HSA-9715357 crizotinib-resistant ALK mutants don't bind crizotinib loss_of_function pubmed:31712133|pubmed:25393798|pubmed:21948233|pubmed:22277784|pubmed:30089600|pubmed:27045755|pubmed:20979473|pubmed:24675041|pubmed:21030459|pubmed:27432227|pubmed:23434628|pubmed:21791641|pubmed:23239810|pubmed:30675302|pubmed:21502504|pubmed:22235099|pubmed:24736079|pubmed:23344087|pubmed:31749991 R-HSA-9717326 crizotinib-resistant ALK mutants R-HSA-9700662 (PTN/MDK):ALK dimer binds type I ALK-binding TKIs Signaling by ALK R-HSA-201556 missense NA

PS3-PS1 ALK p-7Y ALK F1174I [plasma membrane] R-HSA-9699906 ALK UniProt:Q9UM73 L-phenylalanine 1174 replaced with L-isoleucine ReplacedResidue COSMIC:COSM4169861|COSMIC:COSV66568713 neuroblastoma DOID:769 R-HSA-9711929-PI3K-binding ALK mutants [cytosol]-CandidateSet-hasMember|R-HSA-9700077-p-7Y kinase domain ALK mutant dimers [plasma membrane]-CandidateSet-hasCandidate|R-HSA-9711944-STAT3-activating ALK mutants [cytosol]-CandidateSet-hasMember|R-HSA-9713538-SHC-binding ALK mutant dimers [cytosol]-CandidateSet-hasMember R-HSA-9712084|R-HSA-9712083|R-HSA-9712078|R-HSA-9700186|R-HSA-9712085|R-HSA-9712079|R-HSA-9700190|R-HSA-9701810|R-HSA-9700193|R-HSA-9724099 PI3K synthesizes PIP3 downstream of ALK mutants|ALK mutants bind PI3KCA|ALK mutants bind PI3KR1|Autophosphorylation of point mutants of ALK|ALK mutants phosphorylate STAT3|ALK mutants bind STAT3|ALK mutants bind SHC|MECP2 binds the STAT5A gene|ALK mutants phosphorylate SHC1|ALK mutants:p-3Y SHC binds GRB2 pubmed:11110708|pubmed:18097461|pubmed:15374880|pubmed:14962911|pubmed:11280786|pubmed:17353907|pubmed:24060861|pubmed:18923524|pubmed:15588951|pubmed:29455642|pubmed:31366041|pubmed:21656749|pubmed:18923525|pubmed:23201355|pubmed:18594010|pubmed:18923523|pubmed:21494621|pubmed:22249260|pubmed:22570254|pubmed:23104988|pubmed:24445538|pubmed:23139213|pubmed:18724359|pubmed:23239810|pubmed:21847362|pubmed:25421750|pubmed:29873274|pubmed:25342631|pubmed:27573755|pubmed:11850821|pubmed:15895073|pubmed:16170336|pubmed:16939498|pubmed:30692217|pubmed:16153455|pubmed:21415216|pubmed:16825495|pubmed:16871283|pubmed:11751994|pubmed:12934099|pubmed:19459784|pubmed:12424201|pubmed:11943732|pubmed:10706082|pubmed:12185581|pubmed:8633037|pubmed:17922009 R-HSA-9725370|R-HSA-9725371 Signaling by ALK fusions and activated point mutants|Nuclear events stimulated by ALK signaling in cancer R-HSA-201510|R-HSA-201515|R-HSA-201521|R-HSA-9701524 PI3K synthesizes PIP3 downstream of ALK|Active ALK recruits PI3K|ALK autophosphorylation downstream of PTN and MDK|STAT3 is phosphorylated downstream of active ALK Signaling by ALK R-HSA-201556 missense NA

PS3-PS1 ALK p-7Y ALK F1174L [plasma membrane] R-HSA-9699882 ALK UniProt:Q9UM73 L-phenylalanine 1174 replaced with L-leucine ReplacedResidue COSMIC:COSM88796|COSMIC:COSV66555460|COSMIC:COSV66556166|COSMIC:COSV66556325 neuroblastoma DOID:769 pubmed:20632993 R-HSA-9711929-PI3K-binding ALK mutants [cytosol]-CandidateSet-hasMember|R-HSA-9700077-p-7Y kinase domain ALK mutant dimers [plasma membrane]-CandidateSet-hasMember|R-HSA-9711944-STAT3-activating ALK mutants [cytosol]-CandidateSet-hasMember|R-HSA-9713538-SHC-binding ALK mutant dimers [cytosol]-CandidateSet-hasMember R-HSA-9712084|R-HSA-9712083|R-HSA-9712078|R-HSA-9700186|R-HSA-9712085|R-HSA-9712079|R-HSA-9700190|R-HSA-9701810|R-HSA-9700193|R-HSA-9724099 PI3K synthesizes PIP3 downstream of ALK mutants|ALK mutants bind PI3KCA|ALK mutants bind PI3KR1|Autophosphorylation of point mutants of ALK|ALK mutants phosphorylate STAT3|ALK mutants bind STAT3|ALK mutants bind SHC|MECP2 binds the STAT5A gene|ALK mutants phosphorylate SHC1|ALK mutants:p-3Y SHC binds GRB2 pubmed:11110708|pubmed:18097461|pubmed:15374880|pubmed:14962911|pubmed:11280786|pubmed:17353907|pubmed:24060861|pubmed:18923524|pubmed:15588951|pubmed:29455642|pubmed:31366041|pubmed:21656749|pubmed:18923525|pubmed:23201355|pubmed:18594010|pubmed:18923523|pubmed:21494621|pubmed:22249260|pubmed:22570254|pubmed:23104988|pubmed:24445538|pubmed:23139213|pubmed:18724359|pubmed:23239810|pubmed:21847362|pubmed:25421750|pubmed:29873274|pubmed:25342631|pubmed:27573755|pubmed:11850821|pubmed:15895073|pubmed:16170336|pubmed:16939498|pubmed:30692217|pubmed:16153455|pubmed:21415216|pubmed:16825495|pubmed:16871283|pubmed:11751994|pubmed:12934099|pubmed:19459784|pubmed:12424201|pubmed:11943732|pubmed:10706082|pubmed:12185581|pubmed:8633037|pubmed:17922009 R-HSA-9725370|R-HSA-9725371 Signaling by ALK fusions and activated point mutants|Nuclear events stimulated by ALK signaling in cancer R-HSA-201510|R-HSA-201515|R-HSA-201521|R-HSA-9701524 PI3K synthesizes PIP3 downstream of ALK|Active ALK recruits PI3K|ALK autophosphorylation downstream of PTN and MDK|STAT3 is phosphorylated downstream of active ALK Signaling by ALK R-HSA-201556 missense NA

PS3-PS1 ALK p-7Y ALK I1171N [plasma membrane] R-HSA-9700482 ALK UniProt:Q9UM73 L-isoleucine 1171 replaced with L-asparagine ReplacedResidue COSMIC:COSV66556242|COSMIC:COSM9278044 non-small cell lung carcinoma|lung adenocarcinoma|neuroblastoma DOID:3908|DOID:3910|DOID:769 R-HSA-9711929-PI3K-binding ALK mutants [cytosol]-CandidateSet-hasMember|R-HSA-9700077-p-7Y kinase domain ALK mutant dimers [plasma membrane]-CandidateSet-hasMember|R-HSA-9711944-STAT3-activating ALK mutants [cytosol]-CandidateSet-hasMember|R-HSA-9713538-SHC-binding ALK mutant dimers [cytosol]-CandidateSet-hasMember R-HSA-9712084|R-HSA-9712083|R-HSA-9712078|R-HSA-9700186|R-HSA-9712085|R-HSA-9712079|R-HSA-9700190|R-HSA-9701810|R-HSA-9700193|R-HSA-9724099 PI3K synthesizes PIP3 downstream of ALK mutants|ALK mutants bind PI3KCA|ALK mutants bind PI3KR1|Autophosphorylation of point mutants of ALK|ALK mutants phosphorylate STAT3|ALK mutants bind STAT3|ALK mutants bind SHC|MECP2 binds the STAT5A gene|ALK mutants phosphorylate SHC1|ALK mutants:p-3Y SHC binds GRB2 pubmed:11110708|pubmed:18097461|pubmed:15374880|pubmed:14962911|pubmed:11280786|pubmed:17353907|pubmed:24060861|pubmed:18923524|pubmed:15588951|pubmed:29455642|pubmed:31366041|pubmed:21656749|pubmed:18923525|pubmed:23201355|pubmed:18594010|pubmed:18923523|pubmed:21494621|pubmed:22249260|pubmed:22570254|pubmed:23104988|pubmed:24445538|pubmed:23139213|pubmed:18724359|pubmed:23239810|pubmed:21847362|pubmed:25421750|pubmed:29873274|pubmed:25342631|pubmed:27573755|pubmed:11850821|pubmed:15895073|pubmed:16170336|pubmed:16939498|pubmed:30692217|pubmed:16153455|pubmed:21415216|pubmed:16825495|pubmed:16871283|pubmed:11751994|pubmed:12934099|pubmed:19459784|pubmed:12424201|pubmed:11943732|pubmed:10706082|pubmed:12185581|pubmed:8633037|pubmed:17922009 R-HSA-9725370|R-HSA-9725371 Signaling by ALK fusions and activated point mutants|Nuclear events stimulated by ALK signaling in cancer R-HSA-201510|R-HSA-201515|R-HSA-201521|R-HSA-9701524 PI3K synthesizes PIP3 downstream of ALK|Active ALK recruits PI3K|ALK autophosphorylation downstream of PTN and MDK|STAT3 is phosphorylated downstream of active ALK Signaling by ALK R-HSA-201556 missense NA

PS3 ALK p-7Y ALK F1174S [plasma membrane] R-HSA-9699910 ALK UniProt:Q9UM73 L-phenylalanine 1174 replaced with L-serine ReplacedResidue COSMIC:COSV66558767 endometrial cancer|neuroblastoma DOID:1380|DOID:769 R-HSA-9711929-PI3K-binding ALK mutants [cytosol]-CandidateSet-hasMember|R-HSA-9700077-p-7Y kinase domain ALK mutant dimers [plasma membrane]-CandidateSet-hasCandidate R-HSA-9712084|R-HSA-9712083|R-HSA-9712078|R-HSA-9700186 PI3K synthesizes PIP3 downstream of ALK mutants|ALK mutants bind PI3KCA|ALK mutants bind PI3KR1|Autophosphorylation of point mutants of ALK pubmed:11110708|pubmed:18097461|pubmed:15374880|pubmed:14962911|pubmed:11280786|pubmed:17353907|pubmed:24060861|pubmed:18923524|pubmed:15588951|pubmed:29455642|pubmed:31366041|pubmed:21656749|pubmed:18923525|pubmed:23201355|pubmed:18594010|pubmed:18923523|pubmed:21494621|pubmed:22249260|pubmed:22570254|pubmed:23104988|pubmed:24445538|pubmed:23139213|pubmed:18724359|pubmed:23239810|pubmed:21847362|pubmed:25421750 R-HSA-9725370 Signaling by ALK fusions and activated point mutants R-HSA-201510|R-HSA-201515|R-HSA-201521 PI3K synthesizes PIP3 downstream of ALK|Active ALK recruits PI3K|ALK autophosphorylation downstream of PTN and MDK Signaling by ALK R-HSA-201556 missense NA

PS3 ALK p-7Y ALK G1128A [plasma membrane] R-HSA-9700484 ALK UniProt:Q9UM73 glycine 1128 replaced with L-alanine ReplacedResidue COSMIC:COSV66582675 lung adenocarcinoma|neuroblastoma DOID:3910|DOID:769 pubmed:20695522 R-HSA-9711929-PI3K-binding ALK mutants [cytosol]-CandidateSet-hasMember|R-HSA-9700077-p-7Y kinase domain ALK mutant dimers [plasma membrane]-CandidateSet-hasMember|R-HSA-9711944-STAT3-activating ALK mutants [cytosol]-CandidateSet-hasMember|R-HSA-9713538-SHC-binding ALK mutant dimers [cytosol]-CandidateSet-hasMember R-HSA-9712084|R-HSA-9712083|R-HSA-9712078|R-HSA-9700186|R-HSA-9712085|R-HSA-9712079|R-HSA-9700190|R-HSA-9701810|R-HSA-9700193|R-HSA-9724099 PI3K synthesizes PIP3 downstream of ALK mutants|ALK mutants bind PI3KCA|ALK mutants bind PI3KR1|Autophosphorylation of point mutants of ALK|ALK mutants phosphorylate STAT3|ALK mutants bind STAT3|ALK mutants bind SHC|MECP2 binds the STAT5A gene|ALK mutants phosphorylate SHC1|ALK mutants:p-3Y SHC binds GRB2 pubmed:11110708|pubmed:18097461|pubmed:15374880|pubmed:14962911|pubmed:11280786|pubmed:17353907|pubmed:24060861|pubmed:18923524|pubmed:15588951|pubmed:29455642|pubmed:31366041|pubmed:21656749|pubmed:18923525|pubmed:23201355|pubmed:18594010|pubmed:18923523|pubmed:21494621|pubmed:22249260|pubmed:22570254|pubmed:23104988|pubmed:24445538|pubmed:23139213|pubmed:18724359|pubmed:23239810|pubmed:21847362|pubmed:25421750|pubmed:29873274|pubmed:25342631|pubmed:27573755|pubmed:11850821|pubmed:15895073|pubmed:16170336|pubmed:16939498|pubmed:30692217|pubmed:16153455|pubmed:21415216|pubmed:16825495|pubmed:16871283|pubmed:11751994|pubmed:12934099|pubmed:19459784|pubmed:12424201|pubmed:11943732|pubmed:10706082|pubmed:12185581|pubmed:8633037|pubmed:17922009 R-HSA-9725370|R-HSA-9725371 Signaling by ALK fusions and activated point mutants|Nuclear events stimulated by ALK signaling in cancer R-HSA-201510|R-HSA-201515|R-HSA-201521|R-HSA-9701524 PI3K synthesizes PIP3 downstream of ALK|Active ALK recruits PI3K|ALK autophosphorylation downstream of PTN and MDK|STAT3 is phosphorylated downstream of active ALK Signaling by ALK R-HSA-201556 missense NA

PS3 ALK p-7Y ALK F1245C [plasma membrane] R-HSA-9700465 ALK UniProt:Q9UM73 L-phenylalanine 1245 replaced with L-cysteine ReplacedResidue COSMIC:COSV66563458 neuroblastoma DOID:769 R-HSA-9711929-PI3K-binding ALK mutants [cytosol]-CandidateSet-hasMember|R-HSA-9711944-STAT3-activating ALK mutants [cytosol]-CandidateSet-hasMember|R-HSA-9713538-SHC-binding ALK mutant dimers [cytosol]-CandidateSet-hasMember R-HSA-9712084|R-HSA-9712083|R-HSA-9712078|R-HSA-9712085|R-HSA-9712079|R-HSA-9700190|R-HSA-9701810|R-HSA-9700193|R-HSA-9724099 PI3K synthesizes PIP3 downstream of ALK mutants|ALK mutants bind PI3KCA|ALK mutants bind PI3KR1|ALK mutants phosphorylate STAT3|ALK mutants bind STAT3|ALK mutants bind SHC|MECP2 binds the STAT5A gene|ALK mutants phosphorylate SHC1|ALK mutants:p-3Y SHC binds GRB2 pubmed:11110708|pubmed:18097461|pubmed:15374880|pubmed:14962911|pubmed:11280786|pubmed:17353907|pubmed:24060861|pubmed:18923524|pubmed:15588951|pubmed:29455642|pubmed:31366041|pubmed:21656749|pubmed:18923525|pubmed:23201355|pubmed:18594010|pubmed:18923523|pubmed:21494621|pubmed:22249260|pubmed:22570254|pubmed:23104988|pubmed:24445538|pubmed:23139213|pubmed:29873274|pubmed:25342631|pubmed:27573755|pubmed:11850821|pubmed:15895073|pubmed:16170336|pubmed:16939498|pubmed:30692217|pubmed:16153455|pubmed:21415216|pubmed:16825495|pubmed:16871283|pubmed:11751994|pubmed:12934099|pubmed:19459784|pubmed:12424201|pubmed:11943732|pubmed:10706082|pubmed:12185581|pubmed:8633037|pubmed:17922009 R-HSA-9725370|R-HSA-9725371 Signaling by ALK fusions and activated point mutants|Nuclear events stimulated by ALK signaling in cancer R-HSA-201510|R-HSA-201515|R-HSA-9701524 PI3K synthesizes PIP3 downstream of ALK|Active ALK recruits PI3K|STAT3 is phosphorylated downstream of active ALK Signaling by ALK R-HSA-201556 missense NA

PS3 ALK p-7Y ALK R1192P [plasma membrane] R-HSA-9700466 ALK UniProt:Q9UM73 L-arginine 1192 replaced with L-proline ReplacedResidue COSMIC:COSM7340824 lung adenocarcinoma DOID:3910 R-HSA-9711929-PI3K-binding ALK mutants [cytosol]-CandidateSet-hasMember|R-HSA-9711944-STAT3-activating ALK mutants [cytosol]-CandidateSet-hasMember|R-HSA-9713538-SHC-binding ALK mutant dimers [cytosol]-CandidateSet-hasMember R-HSA-9712084|R-HSA-9712083|R-HSA-9712078|R-HSA-9712085|R-HSA-9712079|R-HSA-9700190|R-HSA-9701810|R-HSA-9700193|R-HSA-9724099 PI3K synthesizes PIP3 downstream of ALK mutants|ALK mutants bind PI3KCA|ALK mutants bind PI3KR1|ALK mutants phosphorylate STAT3|ALK mutants bind STAT3|ALK mutants bind SHC|MECP2 binds the STAT5A gene|ALK mutants phosphorylate SHC1|ALK mutants:p-3Y SHC binds GRB2 pubmed:11110708|pubmed:18097461|pubmed:15374880|pubmed:14962911|pubmed:11280786|pubmed:17353907|pubmed:24060861|pubmed:18923524|pubmed:15588951|pubmed:29455642|pubmed:31366041|pubmed:21656749|pubmed:18923525|pubmed:23201355|pubmed:18594010|pubmed:18923523|pubmed:21494621|pubmed:22249260|pubmed:22570254|pubmed:23104988|pubmed:24445538|pubmed:23139213|pubmed:29873274|pubmed:25342631|pubmed:27573755|pubmed:11850821|pubmed:15895073|pubmed:16170336|pubmed:16939498|pubmed:30692217|pubmed:16153455|pubmed:21415216|pubmed:16825495|pubmed:16871283|pubmed:11751994|pubmed:12934099|pubmed:19459784|pubmed:12424201|pubmed:11943732|pubmed:10706082|pubmed:12185581|pubmed:8633037|pubmed:17922009 R-HSA-9725370|R-HSA-9725371 Signaling by ALK fusions and activated point mutants|Nuclear events stimulated by ALK signaling in cancer R-HSA-201510|R-HSA-201515|R-HSA-9701524 PI3K synthesizes PIP3 downstream of ALK|Active ALK recruits PI3K|STAT3 is phosphorylated downstream of active ALK Signaling by ALK R-HSA-201556 missense NA

PS3-PS1 ALK p-7Y ALK L1198F [plasma membrane] R-HSA-9701883 ALK UniProt:Q9UM73 L-leucine 1198 replaced with L-phenylalanine ReplacedResidue COSMIC:COSV66556391|COSMIC:COSM9311666 thyroid carcinoma|lung squamous cell carcinoma DOID:3963|DOID:3907 pubmed:21596819|pubmed:24675041 R-HSA-9711929-PI3K-binding ALK mutants [cytosol]-CandidateSet-hasMember|R-HSA-9700077-p-7Y kinase domain ALK mutant dimers [plasma membrane]-CandidateSet-hasMember|R-HSA-9713538-SHC-binding ALK mutant dimers [cytosol]-CandidateSet-hasMember R-HSA-9712084|R-HSA-9712083|R-HSA-9712078|R-HSA-9700186|R-HSA-9700190|R-HSA-9700193|R-HSA-9724099 PI3K synthesizes PIP3 downstream of ALK mutants|ALK mutants bind PI3KCA|ALK mutants bind PI3KR1|Autophosphorylation of point mutants of ALK|ALK mutants bind SHC|ALK mutants phosphorylate SHC1|ALK mutants:p-3Y SHC binds GRB2 pubmed:11110708|pubmed:18097461|pubmed:15374880|pubmed:14962911|pubmed:11280786|pubmed:17353907|pubmed:24060861|pubmed:18923524|pubmed:15588951|pubmed:29455642|pubmed:31366041|pubmed:21656749|pubmed:18923525|pubmed:23201355|pubmed:18594010|pubmed:18923523|pubmed:21494621|pubmed:22249260|pubmed:22570254|pubmed:23104988|pubmed:24445538|pubmed:23139213|pubmed:18724359|pubmed:23239810|pubmed:21847362|pubmed:25421750|pubmed:27573755|pubmed:11943732|pubmed:10706082|pubmed:12185581|pubmed:8633037 R-HSA-9725370 Signaling by ALK fusions and activated point mutants R-HSA-201510|R-HSA-201515|R-HSA-201521 PI3K synthesizes PIP3 downstream of ALK|Active ALK recruits PI3K|ALK autophosphorylation downstream of PTN and MDK Signaling by ALK R-HSA-201556 missense NA

PS3 ALK p-7Y ALK del318-782 [plasma membrane] R-HSA-9723889 ALK UniProt:Q9UM73 Deletion of residues 318 to 782 FragmentDeletionModification neuroblastoma DOID:769 pubmed:23139213 R-HSA-9711929-PI3K-binding ALK mutants [cytosol]-CandidateSet-hasMember|R-HSA-9700085-p-7Y extracellular domain ALK mutant protein dimers [plasma membrane]-CandidateSet-hasMember|R-HSA-9711944-STAT3-activating ALK mutants [cytosol]-CandidateSet-hasMember|R-HSA-9713538-SHC-binding ALK mutant dimers [cytosol]-CandidateSet-hasMember R-HSA-9712084|R-HSA-9712083|R-HSA-9712078|R-HSA-9700186|R-HSA-9712085|R-HSA-9712079|R-HSA-9700190|R-HSA-9701810|R-HSA-9700193|R-HSA-9724099 PI3K synthesizes PIP3 downstream of ALK mutants|ALK mutants bind PI3KCA|ALK mutants bind PI3KR1|Autophosphorylation of point mutants of ALK|ALK mutants phosphorylate STAT3|ALK mutants bind STAT3|ALK mutants bind SHC|MECP2 binds the STAT5A gene|ALK mutants phosphorylate SHC1|ALK mutants:p-3Y SHC binds GRB2 pubmed:11110708|pubmed:18097461|pubmed:15374880|pubmed:14962911|pubmed:11280786|pubmed:17353907|pubmed:24060861|pubmed:18923524|pubmed:15588951|pubmed:29455642|pubmed:31366041|pubmed:21656749|pubmed:18923525|pubmed:23201355|pubmed:18594010|pubmed:18923523|pubmed:21494621|pubmed:22249260|pubmed:22570254|pubmed:23104988|pubmed:24445538|pubmed:23139213|pubmed:18724359|pubmed:23239810|pubmed:21847362|pubmed:25421750|pubmed:29873274|pubmed:25342631|pubmed:27573755|pubmed:11850821|pubmed:15895073|pubmed:16170336|pubmed:16939498|pubmed:30692217|pubmed:16153455|pubmed:21415216|pubmed:16825495|pubmed:16871283|pubmed:11751994|pubmed:12934099|pubmed:19459784|pubmed:12424201|pubmed:11943732|pubmed:10706082|pubmed:12185581|pubmed:8633037|pubmed:17922009 R-HSA-9725370|R-HSA-9725371 Signaling by ALK fusions and activated point mutants|Nuclear events stimulated by ALK signaling in cancer R-HSA-201510|R-HSA-201515|R-HSA-201521|R-HSA-9701524 PI3K synthesizes PIP3 downstream of ALK|Active ALK recruits PI3K|ALK autophosphorylation downstream of PTN and MDK|STAT3 is phosphorylated downstream of active ALK Signaling by ALK R-HSA-201556 in-frame indel: deletion NA

PS3 ALK p-7Y ALK K1062M [plasma membrane] R-HSA-9700473 ALK UniProt:Q9UM73 L-lysine 1062 replaced with L-methionine ReplacedResidue COSMIC:COSV66591077 neuroblastoma DOID:769 pubmed:18923524 R-HSA-9711929-PI3K-binding ALK mutants [cytosol]-CandidateSet-hasMember|R-HSA-9700558-p-7Y juxtamembrane domain ALK mutant dimers [plasma membrane]-CandidateSet-hasMember|R-HSA-9711944-STAT3-activating ALK mutants [cytosol]-CandidateSet-hasMember|R-HSA-9713538-SHC-binding ALK mutant dimers [cytosol]-CandidateSet-hasMember R-HSA-9712084|R-HSA-9712083|R-HSA-9712078|R-HSA-9700186|R-HSA-9712085|R-HSA-9712079|R-HSA-9700190|R-HSA-9701810|R-HSA-9700193|R-HSA-9724099 PI3K synthesizes PIP3 downstream of ALK mutants|ALK mutants bind PI3KCA|ALK mutants bind PI3KR1|Autophosphorylation of point mutants of ALK|ALK mutants phosphorylate STAT3|ALK mutants bind STAT3|ALK mutants bind SHC|MECP2 binds the STAT5A gene|ALK mutants phosphorylate SHC1|ALK mutants:p-3Y SHC binds GRB2 pubmed:11110708|pubmed:18097461|pubmed:15374880|pubmed:14962911|pubmed:11280786|pubmed:17353907|pubmed:24060861|pubmed:18923524|pubmed:15588951|pubmed:29455642|pubmed:31366041|pubmed:21656749|pubmed:18923525|pubmed:23201355|pubmed:18594010|pubmed:18923523|pubmed:21494621|pubmed:22249260|pubmed:22570254|pubmed:23104988|pubmed:24445538|pubmed:23139213|pubmed:18724359|pubmed:23239810|pubmed:21847362|pubmed:25421750|pubmed:29873274|pubmed:25342631|pubmed:27573755|pubmed:11850821|pubmed:15895073|pubmed:16170336|pubmed:16939498|pubmed:30692217|pubmed:16153455|pubmed:21415216|pubmed:16825495|pubmed:16871283|pubmed:11751994|pubmed:12934099|pubmed:19459784|pubmed:12424201|pubmed:11943732|pubmed:10706082|pubmed:12185581|pubmed:8633037|pubmed:17922009 R-HSA-9725370|R-HSA-9725371 Signaling by ALK fusions and activated point mutants|Nuclear events stimulated by ALK signaling in cancer R-HSA-201510|R-HSA-201515|R-HSA-201521|R-HSA-9701524 PI3K synthesizes PIP3 downstream of ALK|Active ALK recruits PI3K|ALK autophosphorylation downstream of PTN and MDK|STAT3 is phosphorylated downstream of active ALK Signaling by ALK R-HSA-201556 missense NA

PS3 ALK p-7Y ALK G1201E [plasma membrane] R-HSA-9701868 ALK UniProt:Q9UM73 glycine 1201 replaced with L-glutamic acid ReplacedResidue COSMIC:COSV66557297 thyroid carcinoma DOID:3963 pubmed:21596819 R-HSA-9711929-PI3K-binding ALK mutants [cytosol]-CandidateSet-hasMember|R-HSA-9700077-p-7Y kinase domain ALK mutant dimers [plasma membrane]-CandidateSet-hasMember|R-HSA-9713538-SHC-binding ALK mutant dimers [cytosol]-CandidateSet-hasMember R-HSA-9712084|R-HSA-9712083|R-HSA-9712078|R-HSA-9700186|R-HSA-9700190|R-HSA-9700193|R-HSA-9724099 PI3K synthesizes PIP3 downstream of ALK mutants|ALK mutants bind PI3KCA|ALK mutants bind PI3KR1|Autophosphorylation of point mutants of ALK|ALK mutants bind SHC|ALK mutants phosphorylate SHC1|ALK mutants:p-3Y SHC binds GRB2 pubmed:11110708|pubmed:18097461|pubmed:15374880|pubmed:14962911|pubmed:11280786|pubmed:17353907|pubmed:24060861|pubmed:18923524|pubmed:15588951|pubmed:29455642|pubmed:31366041|pubmed:21656749|pubmed:18923525|pubmed:23201355|pubmed:18594010|pubmed:18923523|pubmed:21494621|pubmed:22249260|pubmed:22570254|pubmed:23104988|pubmed:24445538|pubmed:23139213|pubmed:18724359|pubmed:23239810|pubmed:21847362|pubmed:25421750|pubmed:27573755|pubmed:11943732|pubmed:10706082|pubmed:12185581|pubmed:8633037 R-HSA-9725370 Signaling by ALK fusions and activated point mutants R-HSA-201510|R-HSA-201515|R-HSA-201521 PI3K synthesizes PIP3 downstream of ALK|Active ALK recruits PI3K|ALK autophosphorylation downstream of PTN and MDK Signaling by ALK R-HSA-201556 missense NA

PS3-PS1 ALK p-7Y ALK R1275Q [plasma membrane] R-HSA-9699826 ALK UniProt:Q9UM73 L-arginine 1275 replaced with L-glutamine ReplacedResidue COSMIC:COSM88797|COSMIC:COSV66555567 neuroblastoma DOID:769 pubmed:20632993 R-HSA-9711929-PI3K-binding ALK mutants [cytosol]-CandidateSet-hasMember|R-HSA-9700077-p-7Y kinase domain ALK mutant dimers [plasma membrane]-CandidateSet-hasMember|R-HSA-9711944-STAT3-activating ALK mutants [cytosol]-CandidateSet-hasMember|R-HSA-9713538-SHC-binding ALK mutant dimers [cytosol]-CandidateSet-hasMember R-HSA-9712084|R-HSA-9712083|R-HSA-9712078|R-HSA-9700186|R-HSA-9712085|R-HSA-9712079|R-HSA-9700190|R-HSA-9701810|R-HSA-9700193|R-HSA-9724099 PI3K synthesizes PIP3 downstream of ALK mutants|ALK mutants bind PI3KCA|ALK mutants bind PI3KR1|Autophosphorylation of point mutants of ALK|ALK mutants phosphorylate STAT3|ALK mutants bind STAT3|ALK mutants bind SHC|MECP2 binds the STAT5A gene|ALK mutants phosphorylate SHC1|ALK mutants:p-3Y SHC binds GRB2 pubmed:11110708|pubmed:18097461|pubmed:15374880|pubmed:14962911|pubmed:11280786|pubmed:17353907|pubmed:24060861|pubmed:18923524|pubmed:15588951|pubmed:29455642|pubmed:31366041|pubmed:21656749|pubmed:18923525|pubmed:23201355|pubmed:18594010|pubmed:18923523|pubmed:21494621|pubmed:22249260|pubmed:22570254|pubmed:23104988|pubmed:24445538|pubmed:23139213|pubmed:18724359|pubmed:23239810|pubmed:21847362|pubmed:25421750|pubmed:29873274|pubmed:25342631|pubmed:27573755|pubmed:11850821|pubmed:15895073|pubmed:16170336|pubmed:16939498|pubmed:30692217|pubmed:16153455|pubmed:21415216|pubmed:16825495|pubmed:16871283|pubmed:11751994|pubmed:12934099|pubmed:19459784|pubmed:12424201|pubmed:11943732|pubmed:10706082|pubmed:12185581|pubmed:8633037|pubmed:17922009 R-HSA-9725370|R-HSA-9725371 Signaling by ALK fusions and activated point mutants|Nuclear events stimulated by ALK signaling in cancer R-HSA-201510|R-HSA-201515|R-HSA-201521|R-HSA-9701524 PI3K synthesizes PIP3 downstream of ALK|Active ALK recruits PI3K|ALK autophosphorylation downstream of PTN and MDK|STAT3 is phosphorylated downstream of active ALK Signaling by ALK R-HSA-201556 missense NA

PS3 ALK p-7Y ALK del224-318 [plasma membrane] R-HSA-9723882 ALK UniProt:Q9UM73 Deletion of residues 224 to 318 FragmentDeletionModification neuroblastoma DOID:769 pubmed:22249260 R-HSA-9711929-PI3K-binding ALK mutants [cytosol]-CandidateSet-hasMember|R-HSA-9700085-p-7Y extracellular domain ALK mutant protein dimers [plasma membrane]-CandidateSet-hasMember|R-HSA-9711944-STAT3-activating ALK mutants [cytosol]-CandidateSet-hasMember|R-HSA-9713538-SHC-binding ALK mutant dimers [cytosol]-CandidateSet-hasMember R-HSA-9712084|R-HSA-9712083|R-HSA-9712078|R-HSA-9700186|R-HSA-9712085|R-HSA-9712079|R-HSA-9700190|R-HSA-9701810|R-HSA-9700193|R-HSA-9724099 PI3K synthesizes PIP3 downstream of ALK mutants|ALK mutants bind PI3KCA|ALK mutants bind PI3KR1|Autophosphorylation of point mutants of ALK|ALK mutants phosphorylate STAT3|ALK mutants bind STAT3|ALK mutants bind SHC|MECP2 binds the STAT5A gene|ALK mutants phosphorylate SHC1|ALK mutants:p-3Y SHC binds GRB2 pubmed:11110708|pubmed:18097461|pubmed:15374880|pubmed:14962911|pubmed:11280786|pubmed:17353907|pubmed:24060861|pubmed:18923524|pubmed:15588951|pubmed:29455642|pubmed:31366041|pubmed:21656749|pubmed:18923525|pubmed:23201355|pubmed:18594010|pubmed:18923523|pubmed:21494621|pubmed:22249260|pubmed:22570254|pubmed:23104988|pubmed:24445538|pubmed:23139213|pubmed:18724359|pubmed:23239810|pubmed:21847362|pubmed:25421750|pubmed:29873274|pubmed:25342631|pubmed:27573755|pubmed:11850821|pubmed:15895073|pubmed:16170336|pubmed:16939498|pubmed:30692217|pubmed:16153455|pubmed:21415216|pubmed:16825495|pubmed:16871283|pubmed:11751994|pubmed:12934099|pubmed:19459784|pubmed:12424201|pubmed:11943732|pubmed:10706082|pubmed:12185581|pubmed:8633037|pubmed:17922009 R-HSA-9725370|R-HSA-9725371 Signaling by ALK fusions and activated point mutants|Nuclear events stimulated by ALK signaling in cancer R-HSA-201510|R-HSA-201515|R-HSA-201521|R-HSA-9701524 PI3K synthesizes PIP3 downstream of ALK|Active ALK recruits PI3K|ALK autophosphorylation downstream of PTN and MDK|STAT3 is phosphorylated downstream of active ALK Signaling by ALK R-HSA-201556 in-frame indel: deletion NA

PS3 ALK p-7Y ALK T1087I [plasma membrane] R-HSA-9723885 ALK UniProt:Q9UM73 L-threonine 1087 replaced with L-isoleucine ReplacedResidue neuroblastoma DOID:769 pubmed:23104988|pubmed:18923524 R-HSA-9700558-p-7Y juxtamembrane domain ALK mutant dimers [plasma membrane]-CandidateSet-hasMember R-HSA-9700186 Autophosphorylation of point mutants of ALK pubmed:18724359|pubmed:29455642|pubmed:18923525|pubmed:23239810|pubmed:24060861|pubmed:21847362|pubmed:25421750|pubmed:18923524 R-HSA-9725370 Signaling by ALK fusions and activated point mutants R-HSA-201521 ALK autophosphorylation downstream of PTN and MDK Signaling by ALK R-HSA-201556 missense NA

PM5 ALK p-7Y ALK R1061Q [plasma membrane] R-HSA-9723884 ALK UniProt:Q9UM73 L-arginine 1062 replaced with L-glutamine ReplacedResidue COSMIC:COSV66572475 neuroblastoma DOID:769 pubmed:20719933 R-HSA-9700558-p-7Y juxtamembrane domain ALK mutant dimers [plasma membrane]-CandidateSet-hasCandidate R-HSA-9700186 Autophosphorylation of point mutants of ALK pubmed:18724359|pubmed:29455642|pubmed:18923525|pubmed:23239810|pubmed:24060861|pubmed:21847362|pubmed:25421750|pubmed:18923524 R-HSA-9725370 Signaling by ALK fusions and activated point mutants R-HSA-201521 ALK autophosphorylation downstream of PTN and MDK Signaling by ALK R-HSA-201556 missense NA

PS3 ALK p-7Y ALK L1196M [plasma membrane] R-HSA-9700464 ALK UniProt:Q9UM73 L-leucine 1196 replaced with L-methionine ReplacedResidue COSMIC:COSV66556435 lung adenocarcinoma|neuroblastoma DOID:3910|DOID:769 R-HSA-9700077-p-7Y kinase domain ALK mutant dimers [plasma membrane]-CandidateSet-hasMember R-HSA-9700186 Autophosphorylation of point mutants of ALK pubmed:18724359|pubmed:29455642|pubmed:18923525|pubmed:23239810|pubmed:24060861|pubmed:21847362|pubmed:25421750|pubmed:18923524 R-HSA-9725370 Signaling by ALK fusions and activated point mutants R-HSA-201521 ALK autophosphorylation downstream of PTN and MDK Signaling by ALK R-HSA-201556 missense NA

PS3 ALK p-7Y ALK M1166R [plasma membrane] R-HSA-9700477 ALK UniProt:Q9UM73 L-methionine 1166 replaced with L-arginine ReplacedResidue COSMIC:COSV66565852 neuroblastoma DOID:769 R-HSA-9700077-p-7Y kinase domain ALK mutant dimers [plasma membrane]-CandidateSet-hasMember R-HSA-9700186 Autophosphorylation of point mutants of ALK pubmed:18724359|pubmed:29455642|pubmed:18923525|pubmed:23239810|pubmed:24060861|pubmed:21847362|pubmed:25421750|pubmed:18923524 R-HSA-9725370 Signaling by ALK fusions and activated point mutants R-HSA-201521 ALK autophosphorylation downstream of PTN and MDK Signaling by ALK R-HSA-201556 missense NA

PS3 ALK p-Y ALK S1206C [plasma membrane] R-HSA-9715292 ALK UniProt:Q9UM73 L-serine 1206 replaced with L-cysteine ReplacedResidue COSMIC:COSM9311656 non-small cell lung carcinoma DOID:3908 pubmed:27432227|pubmed:25421750 R-HSA-9700077-p-7Y kinase domain ALK mutant dimers [plasma membrane]-CandidateSet-hasMember R-HSA-9700186 Autophosphorylation of point mutants of ALK pubmed:18724359|pubmed:29455642|pubmed:18923525|pubmed:23239810|pubmed:24060861|pubmed:21847362|pubmed:25421750|pubmed:18923524 R-HSA-9725370 Signaling by ALK fusions and activated point mutants R-HSA-201521 ALK autophosphorylation downstream of PTN and MDK Signaling by ALK R-HSA-201556 missense NA

PS3 ALK p- 7Y ALK L1122V [plasma membrane] R-HSA-9715294 ALK UniProt:Q9UM73 L-leucine 1122 replaced with L-valine ReplacedResidue COSMIC:COSM9311665 non-small cell lung carcinoma DOID:3908 pubmed:29650534|pubmed:25421750 R-HSA-9700077-p-7Y kinase domain ALK mutant dimers [plasma membrane]-CandidateSet-hasMember R-HSA-9700186 Autophosphorylation of point mutants of ALK pubmed:18724359|pubmed:29455642|pubmed:18923525|pubmed:23239810|pubmed:24060861|pubmed:21847362|pubmed:25421750|pubmed:18923524 R-HSA-9725370 Signaling by ALK fusions and activated point mutants R-HSA-201521 ALK autophosphorylation downstream of PTN and MDK Signaling by ALK R-HSA-201556 missense NA

PS3 ALK p-7Y ALK E1384K [plasma membrane] R-HSA-9715285 ALK UniProt:Q9UM73 L-glutamic acid 1384 replaced with L-lysine ReplacedResidue COSMIC:COSV66589093 anaplastic large cell lymphoma|cervical squamous cell carcinoma DOID:0050744|DOID:3744 pubmed:21847362 R-HSA-9700077-p-7Y kinase domain ALK mutant dimers [plasma membrane]-CandidateSet-hasMember R-HSA-9700186 Autophosphorylation of point mutants of ALK pubmed:18724359|pubmed:29455642|pubmed:18923525|pubmed:23239810|pubmed:24060861|pubmed:21847362|pubmed:25421750|pubmed:18923524 R-HSA-9725370 Signaling by ALK fusions and activated point mutants R-HSA-201521 ALK autophosphorylation downstream of PTN and MDK Signaling by ALK R-HSA-201556 missense NA

PS3 ALK p-7Y ALK L1196Q [plasma membrane] R-HSA-9700476 ALK UniProt:Q9UM73 L-leucine 1196 replaced with L-asparagine ReplacedResidue COSMIC:COSV66565358 anaplastic large cell lymphoma DOID:0050744 R-HSA-9700077-p-7Y kinase domain ALK mutant dimers [plasma membrane]-CandidateSet-hasMember R-HSA-9700186 Autophosphorylation of point mutants of ALK pubmed:18724359|pubmed:29455642|pubmed:18923525|pubmed:23239810|pubmed:24060861|pubmed:21847362|pubmed:25421750|pubmed:18923524 R-HSA-9725370 Signaling by ALK fusions and activated point mutants R-HSA-201521 ALK autophosphorylation downstream of PTN and MDK Signaling by ALK R-HSA-201556 missense NA

PS3 ALK p-7Y ALK V1180L [plasma membrane] R-HSA-9715289 ALK UniProt:Q9UM73 L-valine 1180 replaced with L-leucine ReplacedResidue COSMIC:COSV66587894 lung squamous cell carcinoma DOID:3907 pubmed:25228534 R-HSA-9700077-p-7Y kinase domain ALK mutant dimers [plasma membrane]-CandidateSet-hasMember R-HSA-9700186 Autophosphorylation of point mutants of ALK pubmed:18724359|pubmed:29455642|pubmed:18923525|pubmed:23239810|pubmed:24060861|pubmed:21847362|pubmed:25421750|pubmed:18923524 R-HSA-9725370 Signaling by ALK fusions and activated point mutants R-HSA-201521 ALK autophosphorylation downstream of PTN and MDK Signaling by ALK R-HSA-201556 missense NA

PM5-PS1 ALK p-7Y ALK G1201R [plasma membrane] R-HSA-9701860 ALK UniProt:Q9UM73 glycine 1201 replaced with L-arginine ReplacedResidue COSMIC:COSM7002298|COSMIC:COSV66565301 melanoma DOID:1909 R-HSA-9700077-p-7Y kinase domain ALK mutant dimers [plasma membrane]-CandidateSet-hasCandidate R-HSA-9700186 Autophosphorylation of point mutants of ALK pubmed:18724359|pubmed:29455642|pubmed:18923525|pubmed:23239810|pubmed:24060861|pubmed:21847362|pubmed:25421750|pubmed:18923524 R-HSA-9725370 Signaling by ALK fusions and activated point mutants R-HSA-201521 ALK autophosphorylation downstream of PTN and MDK Signaling by ALK R-HSA-201556 missense NA

PM5-PS1 ALK p-7Y ALK I1171T [plasma membrane] R-HSA-9700489 ALK UniProt:Q9UM73 L-isoleucine 1171 replaced with L-threonine ReplacedResidue COSMIC:COSV66564577|COSMIC:COSM9306547 non-small cell lung carcinoma|lung adenocarcinoma DOID:3908|DOID:3910 R-HSA-9700077-p-7Y kinase domain ALK mutant dimers [plasma membrane]-CandidateSet-hasCandidate R-HSA-9700186 Autophosphorylation of point mutants of ALK pubmed:18724359|pubmed:29455642|pubmed:18923525|pubmed:23239810|pubmed:24060861|pubmed:21847362|pubmed:25421750|pubmed:18923524 R-HSA-9725370 Signaling by ALK fusions and activated point mutants R-HSA-201521 ALK autophosphorylation downstream of PTN and MDK Signaling by ALK R-HSA-201556 missense NA

PM5 ALK p-7Y ALK R1275L [plasma membrane] R-HSA-9699800 ALK UniProt:Q9UM73 L-arginine 1275 replaced with L-leucine ReplacedResidue COSMIC:COSV66558183 neuroblastoma DOID:769 R-HSA-9700077-p-7Y kinase domain ALK mutant dimers [plasma membrane]-CandidateSet-hasCandidate R-HSA-9700186 Autophosphorylation of point mutants of ALK pubmed:18724359|pubmed:29455642|pubmed:18923525|pubmed:23239810|pubmed:24060861|pubmed:21847362|pubmed:25421750|pubmed:18923524 R-HSA-9725370 Signaling by ALK fusions and activated point mutants R-HSA-201521 ALK autophosphorylation downstream of PTN and MDK Signaling by ALK R-HSA-201556 missense NA

PM5-PS1 ALK p-7Y ALK F1174C [plasma membrane] R-HSA-9699821 ALK UniProt:Q9UM73 L-phenylalanine 1174 replaced with L-cysteine ReplacedResidue COSMIC:COSV66560921|COSMIC:COSM4422790 non-small cell lung carcinoma|neuroblastoma DOID:3908|DOID:769 R-HSA-9700077-p-7Y kinase domain ALK mutant dimers [plasma membrane]-CandidateSet-hasCandidate R-HSA-9700186 Autophosphorylation of point mutants of ALK pubmed:18724359|pubmed:29455642|pubmed:18923525|pubmed:23239810|pubmed:24060861|pubmed:21847362|pubmed:25421750|pubmed:18923524 R-HSA-9725370 Signaling by ALK fusions and activated point mutants R-HSA-201521 ALK autophosphorylation downstream of PTN and MDK Signaling by ALK R-HSA-201556 missense NA

PM5-PS1 ALK p-7Y ALK F1174V [plasma membrane] R-HSA-9699921 ALK UniProt:Q9UM73 L-phenylalanine 1174 replaced with L-valine ReplacedResidue COSMIC:COSV66559314|COSMIC:COSM4422791 non-small cell lung carcinoma|neuroblastoma DOID:3908|DOID:769 R-HSA-9700077-p-7Y kinase domain ALK mutant dimers [plasma membrane]-CandidateSet-hasCandidate R-HSA-9700186 Autophosphorylation of point mutants of ALK pubmed:18724359|pubmed:29455642|pubmed:18923525|pubmed:23239810|pubmed:24060861|pubmed:21847362|pubmed:25421750|pubmed:18923524 R-HSA-9725370 Signaling by ALK fusions and activated point mutants R-HSA-201521 ALK autophosphorylation downstream of PTN and MDK Signaling by ALK R-HSA-201556 missense NA

PM5 ALK p-7Y ALK M1166N [plasma membrane] R-HSA-9723299 ALK UniProt:Q9UM73 L-methionine 1166 replaced with L-asparagine ReplacedResidue COSMIC:COSV66574940 neuroblastoma DOID:769 pubmed:23104988 R-HSA-9700077-p-7Y kinase domain ALK mutant dimers [plasma membrane]-CandidateSet-hasCandidate R-HSA-9700186 Autophosphorylation of point mutants of ALK pubmed:18724359|pubmed:29455642|pubmed:18923525|pubmed:23239810|pubmed:24060861|pubmed:21847362|pubmed:25421750|pubmed:18923524 R-HSA-9725370 Signaling by ALK fusions and activated point mutants R-HSA-201521 ALK autophosphorylation downstream of PTN and MDK Signaling by ALK R-HSA-201556 missense NA

PM5 ALK p-7Y ALK M1166T [plasma membrane] R-HSA-9700486 ALK UniProt:Q9UM73 L-methionine 1166 replaced with L-threonine ReplacedResidue COSMIC:COSV66588108 non-small cell lung carcinoma DOID:3908 R-HSA-9700077-p-7Y kinase domain ALK mutant dimers [plasma membrane]-CandidateSet-hasCandidate R-HSA-9700186 Autophosphorylation of point mutants of ALK pubmed:18724359|pubmed:29455642|pubmed:18923525|pubmed:23239810|pubmed:24060861|pubmed:21847362|pubmed:25421750|pubmed:18923524 R-HSA-9725370 Signaling by ALK fusions and activated point mutants R-HSA-201521 ALK autophosphorylation downstream of PTN and MDK Signaling by ALK R-HSA-201556 missense NA

PM5 ALK p-7Y ALK Y1239H [plasma membrane] R-HSA-9715290 ALK UniProt:Q9UM73 L-tyrosine 1239 replaced with L-histidine ReplacedResidue anaplastic large cell lymphoma DOID:0050744 pubmed:21847362 R-HSA-9700077-p-7Y kinase domain ALK mutant dimers [plasma membrane]-CandidateSet-hasCandidate R-HSA-9700186 Autophosphorylation of point mutants of ALK pubmed:18724359|pubmed:29455642|pubmed:18923525|pubmed:23239810|pubmed:24060861|pubmed:21847362|pubmed:25421750|pubmed:18923524 R-HSA-9725370 Signaling by ALK fusions and activated point mutants R-HSA-201521 ALK autophosphorylation downstream of PTN and MDK Signaling by ALK R-HSA-201556 missense NA

PM1-PM4 ALK p-7Y ALK R1275* [plasma membrane] R-HSA-9699915 ALK UniProt:Q9UM73 Nonsense mutation at L-arginine 1275 NonsenseMutation COSMIC:COSV66557562 astrocytoma DOID:3069 R-HSA-9700077-p-7Y kinase domain ALK mutant dimers [plasma membrane]-CandidateSet-hasCandidate R-HSA-9700186 Autophosphorylation of point mutants of ALK pubmed:18724359|pubmed:29455642|pubmed:18923525|pubmed:23239810|pubmed:24060861|pubmed:21847362|pubmed:25421750|pubmed:18923524 R-HSA-9725370 Signaling by ALK fusions and activated point mutants R-HSA-201521 ALK autophosphorylation downstream of PTN and MDK Signaling by ALK R-HSA-201556 nonsense NA

PM5 ALK p-7Y ALK I1171S [plasma membrane] R-HSA-9714063 ALK UniProt:Q9UM73 L-isoleucine 1171 replaced with L-serine ReplacedResidue COSMIC:COSV66567934 non-small cell lung carcinoma DOID:3908 pubmed:31943796 R-HSA-9700077-p-7Y kinase domain ALK mutant dimers [plasma membrane]-CandidateSet-hasCandidate R-HSA-9700186 Autophosphorylation of point mutants of ALK pubmed:18724359|pubmed:29455642|pubmed:18923525|pubmed:23239810|pubmed:24060861|pubmed:21847362|pubmed:25421750|pubmed:18923524 R-HSA-9725370 Signaling by ALK fusions and activated point mutants R-HSA-201521 ALK autophosphorylation downstream of PTN and MDK Signaling by ALK R-HSA-201556 missense NA

PM5-PS1 ALK p-7Y ALK G1128S [plasma membrane] R-HSA-9700462 ALK UniProt:Q9UM73 glycine 1128 replaced with L-serine ReplacedResidue COSMIC:COSV66567934|COSMIC:COSM5352215 non-small cell lung carcinoma DOID:3908 R-HSA-9700077-p-7Y kinase domain ALK mutant dimers [plasma membrane]-CandidateSet-hasCandidate R-HSA-9700186 Autophosphorylation of point mutants of ALK pubmed:18724359|pubmed:29455642|pubmed:18923525|pubmed:23239810|pubmed:24060861|pubmed:21847362|pubmed:25421750|pubmed:18923524 R-HSA-9725370 Signaling by ALK fusions and activated point mutants R-HSA-201521 ALK autophosphorylation downstream of PTN and MDK Signaling by ALK R-HSA-201556 missense NA

PM5 ALK p-7Y ALK G1128V [plasma membrane] R-HSA-9700480 ALK UniProt:Q9UM73 glycine 1128 replaced with L-valine ReplacedResidue COSMIC:COSV101202380 melanoma DOID:1909 R-HSA-9700077-p-7Y kinase domain ALK mutant dimers [plasma membrane]-CandidateSet-hasCandidate R-HSA-9700186 Autophosphorylation of point mutants of ALK pubmed:18724359|pubmed:29455642|pubmed:18923525|pubmed:23239810|pubmed:24060861|pubmed:21847362|pubmed:25421750|pubmed:18923524 R-HSA-9725370 Signaling by ALK fusions and activated point mutants R-HSA-201521 ALK autophosphorylation downstream of PTN and MDK Signaling by ALK R-HSA-201556 missense NA

PS3 ALK p-7Y ALK H694R [plasma membrane] R-HSA-9715293 ALK UniProt:Q9UM73 L-histidine 694 replaced with L-arginine ReplacedResidue anaplastic large cell lymphoma DOID:0050744 pubmed:21847362 R-HSA-9700085-p-7Y extracellular domain ALK mutant protein dimers [plasma membrane]-CandidateSet-hasMember R-HSA-9700186 Autophosphorylation of point mutants of ALK pubmed:18724359|pubmed:29455642|pubmed:18923525|pubmed:23239810|pubmed:24060861|pubmed:21847362|pubmed:25421750|pubmed:18923524 R-HSA-9725370 Signaling by ALK fusions and activated point mutants R-HSA-201521 ALK autophosphorylation downstream of PTN and MDK Signaling by ALK R-HSA-201556 missense NA

PM5 ALK p-7Y ALK G881D [plasma membrane] R-HSA-9715286 ALK UniProt:Q9UM73 glycine 881 replaced with L-aspartic acid ReplacedResidue anaplastic large cell lymphoma DOID:0050744 pubmed:21847362 R-HSA-9700085-p-7Y extracellular domain ALK mutant protein dimers [plasma membrane]-CandidateSet-hasCandidate R-HSA-9700186 Autophosphorylation of point mutants of ALK pubmed:18724359|pubmed:29455642|pubmed:18923525|pubmed:23239810|pubmed:24060861|pubmed:21847362|pubmed:25421750|pubmed:18923524 R-HSA-9725370 Signaling by ALK fusions and activated point mutants R-HSA-201521 ALK autophosphorylation downstream of PTN and MDK Signaling by ALK R-HSA-201556 missense NA

PM5 ALK p-7Y ALK V597A [plasma membrane] R-HSA-9715295 ALK UniProt:Q9UM73 L-valine 597 replaced with L-alanine ReplacedResidue anaplastic large cell lymphoma DOID:0050744 pubmed:21847362 R-HSA-9700085-p-7Y extracellular domain ALK mutant protein dimers [plasma membrane]-CandidateSet-hasCandidate R-HSA-9700186 Autophosphorylation of point mutants of ALK pubmed:18724359|pubmed:29455642|pubmed:18923525|pubmed:23239810|pubmed:24060861|pubmed:21847362|pubmed:25421750|pubmed:18923524 R-HSA-9725370 Signaling by ALK fusions and activated point mutants R-HSA-201521 ALK autophosphorylation downstream of PTN and MDK Signaling by ALK R-HSA-201556 missense NA

PM5 ALK p-7Y ALK S413N [plasma membrane] R-HSA-9715287 ALK UniProt:Q9UM73 L-serine 413 replaced with L-asparagine ReplacedResidue anaplastic large cell lymphoma DOID:0050744 pubmed:21847362 R-HSA-9700085-p-7Y extracellular domain ALK mutant protein dimers [plasma membrane]-CandidateSet-hasCandidate R-HSA-9700186 Autophosphorylation of point mutants of ALK pubmed:18724359|pubmed:29455642|pubmed:18923525|pubmed:23239810|pubmed:24060861|pubmed:21847362|pubmed:25421750|pubmed:18923524 R-HSA-9725370 Signaling by ALK fusions and activated point mutants R-HSA-201521 ALK autophosphorylation downstream of PTN and MDK Signaling by ALK R-HSA-201556 missense NA

PS3 ALK ALK H694R [plasma membrane] R-HSA-9715263 ALK UniProt:Q9UM73 L-histidine 694 replaced with L-arginine ReplacedResidue anaplastic large cell lymphoma DOID:0050744 pubmed:21847362 R-HSA-9700078-extracellular domain ALK mutant dimers [plasma membrane]-CandidateSet-hasMember|R-HSA-9700087-extracellular domain ALK mutants [plasma membrane]-CandidateSet-hasMember R-HSA-9700186|R-HSA-9700184 Autophosphorylation of point mutants of ALK|Ligand-independent dimerization of point mutants of ALK pubmed:18724359|pubmed:29455642|pubmed:18923525|pubmed:23239810|pubmed:24060861|pubmed:21847362|pubmed:25421750|pubmed:18923524|pubmed:18923523|pubmed:21596819|pubmed:21838707|pubmed:27573755|pubmed:23104988|pubmed:23201355 R-HSA-9725370 Signaling by ALK fusions and activated point mutants R-HSA-201521|R-HSA-201519 ALK autophosphorylation downstream of PTN and MDK|ALK:PTN dimerization Signaling by ALK R-HSA-201556 missense NA

PS3 ALK ALK del318-782 [plasma membrane] R-HSA-9723869 ALK UniProt:Q9UM73 Deletion of residues 318 to 782 FragmentDeletionModification neuroblastoma DOID:769 pubmed:23139213 R-HSA-9700078-extracellular domain ALK mutant dimers [plasma membrane]-CandidateSet-hasMember|R-HSA-9700087-extracellular domain ALK mutants [plasma membrane]-CandidateSet-hasMember R-HSA-9700186|R-HSA-9700184 Autophosphorylation of point mutants of ALK|Ligand-independent dimerization of point mutants of ALK pubmed:18724359|pubmed:29455642|pubmed:18923525|pubmed:23239810|pubmed:24060861|pubmed:21847362|pubmed:25421750|pubmed:18923524|pubmed:18923523|pubmed:21596819|pubmed:21838707|pubmed:27573755|pubmed:23104988|pubmed:23201355 R-HSA-9725370 Signaling by ALK fusions and activated point mutants R-HSA-201521|R-HSA-201519 ALK autophosphorylation downstream of PTN and MDK|ALK:PTN dimerization Signaling by ALK R-HSA-201556 in-frame indel: deletion NA

PS3 ALK ALK H224_G318del [plasma membrane] R-HSA-9723870 ALK UniProt:Q9UM73 Deletion of residues 224 to 318 FragmentDeletionModification neuroblastoma DOID:769 pubmed:22249260 R-HSA-9700078-extracellular domain ALK mutant dimers [plasma membrane]-CandidateSet-hasMember|R-HSA-9700087-extracellular domain ALK mutants [plasma membrane]-CandidateSet-hasMember R-HSA-9700186|R-HSA-9700184 Autophosphorylation of point mutants of ALK|Ligand-independent dimerization of point mutants of ALK pubmed:18724359|pubmed:29455642|pubmed:18923525|pubmed:23239810|pubmed:24060861|pubmed:21847362|pubmed:25421750|pubmed:18923524|pubmed:18923523|pubmed:21596819|pubmed:21838707|pubmed:27573755|pubmed:23104988|pubmed:23201355 R-HSA-9725370 Signaling by ALK fusions and activated point mutants R-HSA-201521|R-HSA-201519 ALK autophosphorylation downstream of PTN and MDK|ALK:PTN dimerization Signaling by ALK R-HSA-201556 in-frame indel: deletion NA

PM5 ALK ALK V597A [plasma membrane] R-HSA-9715265 ALK UniProt:Q9UM73 L-valine 597 replaced with L-alanine ReplacedResidue anaplastic large cell lymphoma DOID:0050744 pubmed:21847362 R-HSA-9700078-extracellular domain ALK mutant dimers [plasma membrane]-CandidateSet-hasCandidate|R-HSA-9700087-extracellular domain ALK mutants [plasma membrane]-CandidateSet-hasCandidate R-HSA-9700186|R-HSA-9700184 Autophosphorylation of point mutants of ALK|Ligand-independent dimerization of point mutants of ALK pubmed:18724359|pubmed:29455642|pubmed:18923525|pubmed:23239810|pubmed:24060861|pubmed:21847362|pubmed:25421750|pubmed:18923524|pubmed:18923523|pubmed:21596819|pubmed:21838707|pubmed:27573755|pubmed:23104988|pubmed:23201355 R-HSA-9725370 Signaling by ALK fusions and activated point mutants R-HSA-201521|R-HSA-201519 ALK autophosphorylation downstream of PTN and MDK|ALK:PTN dimerization Signaling by ALK R-HSA-201556 missense NA

PM5 ALK ALK S413N [plasma membrane] R-HSA-9715267 ALK UniProt:Q9UM73 L-serine 413 replaced with L-asparagine ReplacedResidue anaplastic large cell lymphoma DOID:0050744 pubmed:21847362 R-HSA-9700078-extracellular domain ALK mutant dimers [plasma membrane]-CandidateSet-hasCandidate|R-HSA-9700087-extracellular domain ALK mutants [plasma membrane]-CandidateSet-hasCandidate R-HSA-9700186|R-HSA-9700184 Autophosphorylation of point mutants of ALK|Ligand-independent dimerization of point mutants of ALK pubmed:18724359|pubmed:29455642|pubmed:18923525|pubmed:23239810|pubmed:24060861|pubmed:21847362|pubmed:25421750|pubmed:18923524|pubmed:18923523|pubmed:21596819|pubmed:21838707|pubmed:27573755|pubmed:23104988|pubmed:23201355 R-HSA-9725370 Signaling by ALK fusions and activated point mutants R-HSA-201521|R-HSA-201519 ALK autophosphorylation downstream of PTN and MDK|ALK:PTN dimerization Signaling by ALK R-HSA-201556 missense NA

PM5 ALK ALK G881D [plasma membrane] R-HSA-9715260 ALK UniProt:Q9UM73 glycine 881 replaced with L-aspartic acid ReplacedResidue anaplastic large cell lymphoma DOID:0050744 pubmed:21847362 R-HSA-9700078-extracellular domain ALK mutant dimers [plasma membrane]-CandidateSet-hasCandidate|R-HSA-9700087-extracellular domain ALK mutants [plasma membrane]-CandidateSet-hasCandidate R-HSA-9700186|R-HSA-9700184 Autophosphorylation of point mutants of ALK|Ligand-independent dimerization of point mutants of ALK pubmed:18724359|pubmed:29455642|pubmed:18923525|pubmed:23239810|pubmed:24060861|pubmed:21847362|pubmed:25421750|pubmed:18923524|pubmed:18923523|pubmed:21596819|pubmed:21838707|pubmed:27573755|pubmed:23104988|pubmed:23201355 R-HSA-9725370 Signaling by ALK fusions and activated point mutants R-HSA-201521|R-HSA-201519 ALK autophosphorylation downstream of PTN and MDK|ALK:PTN dimerization Signaling by ALK R-HSA-201556 missense NA

PS3 ALK ALK T1087I [plasma membrane] R-HSA-9723874 ALK UniProt:Q9UM73 L-threonine 1087 replaced with L-isoleucine ReplacedResidue neuroblastoma DOID:769 pubmed:23104988|pubmed:18923524 R-HSA-9700430-juxtamembrane domain ALK mutant dimers [plasma membrane]-CandidateSet-hasMember|R-HSA-9700559-juxtamembrane domain ALK mutants [plasma membrane]-CandidateSet-hasMember R-HSA-9700186|R-HSA-9700184 Autophosphorylation of point mutants of ALK|Ligand-independent dimerization of point mutants of ALK pubmed:18724359|pubmed:29455642|pubmed:18923525|pubmed:23239810|pubmed:24060861|pubmed:21847362|pubmed:25421750|pubmed:18923524|pubmed:18923523|pubmed:21596819|pubmed:21838707|pubmed:27573755|pubmed:23104988|pubmed:23201355 R-HSA-9725370 Signaling by ALK fusions and activated point mutants R-HSA-201521|R-HSA-201519 ALK autophosphorylation downstream of PTN and MDK|ALK:PTN dimerization Signaling by ALK R-HSA-201556 missense NA

PS3 ALK ALK K1062M [plasma membrane] R-HSA-9700435 ALK UniProt:Q9UM73 L-lysine 1062 replaced with L-methionine ReplacedResidue COSMIC:COSV66591077 neuroblastoma DOID:769 pubmed:21596819|pubmed:18923524 R-HSA-9700430-juxtamembrane domain ALK mutant dimers [plasma membrane]-CandidateSet-hasMember|R-HSA-9700559-juxtamembrane domain ALK mutants [plasma membrane]-CandidateSet-hasMember R-HSA-9700186|R-HSA-9700184 Autophosphorylation of point mutants of ALK|Ligand-independent dimerization of point mutants of ALK pubmed:18724359|pubmed:29455642|pubmed:18923525|pubmed:23239810|pubmed:24060861|pubmed:21847362|pubmed:25421750|pubmed:18923524|pubmed:18923523|pubmed:21596819|pubmed:21838707|pubmed:27573755|pubmed:23104988|pubmed:23201355 R-HSA-9725370 Signaling by ALK fusions and activated point mutants R-HSA-201521|R-HSA-201519 ALK autophosphorylation downstream of PTN and MDK|ALK:PTN dimerization Signaling by ALK R-HSA-201556 missense NA

PM5 ALK ALK R1061Q [plasma membrane] R-HSA-9723864 ALK UniProt:Q9UM73 L-arginine 1062 replaced with L-glutamine ReplacedResidue COSMIC:COSV66572475 neuroblastoma DOID:769 pubmed:20719933 R-HSA-9700430-juxtamembrane domain ALK mutant dimers [plasma membrane]-CandidateSet-hasCandidate|R-HSA-9700559-juxtamembrane domain ALK mutants [plasma membrane]-CandidateSet-hasCandidate R-HSA-9700186|R-HSA-9700184 Autophosphorylation of point mutants of ALK|Ligand-independent dimerization of point mutants of ALK pubmed:18724359|pubmed:29455642|pubmed:18923525|pubmed:23239810|pubmed:24060861|pubmed:21847362|pubmed:25421750|pubmed:18923524|pubmed:18923523|pubmed:21596819|pubmed:21838707|pubmed:27573755|pubmed:23104988|pubmed:23201355 R-HSA-9725370 Signaling by ALK fusions and activated point mutants R-HSA-201521|R-HSA-201519 ALK autophosphorylation downstream of PTN and MDK|ALK:PTN dimerization Signaling by ALK R-HSA-201556 missense NA

PS3 ALK ALK G1201E [plasma membrane] R-HSA-9701842 ALK UniProt:Q9UM73 glycine 1201 replaced with L-glutamic acid ReplacedResidue COSMIC:COSV66557297 thyroid carcinoma DOID:3963 pubmed:21596819 R-HSA-9700082-kinase domain ALK mutant dimers [plasma membrane]-CandidateSet-hasMember|R-HSA-9700083-kinase domain ALK mutants [plasma membrane]-CandidateSet-hasMember R-HSA-9700186|R-HSA-9700184 Autophosphorylation of point mutants of ALK|Ligand-independent dimerization of point mutants of ALK pubmed:18724359|pubmed:29455642|pubmed:18923525|pubmed:23239810|pubmed:24060861|pubmed:21847362|pubmed:25421750|pubmed:18923524|pubmed:18923523|pubmed:21596819|pubmed:21838707|pubmed:27573755|pubmed:23104988|pubmed:23201355 R-HSA-9725370 Signaling by ALK fusions and activated point mutants R-HSA-201521|R-HSA-201519 ALK autophosphorylation downstream of PTN and MDK|ALK:PTN dimerization Signaling by ALK R-HSA-201556 missense NA

PS3 ALK ALK M1166R [plasma membrane] R-HSA-9699902 ALK UniProt:Q9UM73 L-methionine 1166 replaced with L-arginine ReplacedResidue COSMIC:COSV66565852 neuroblastoma DOID:769 pubmed:23104988 R-HSA-9700082-kinase domain ALK mutant dimers [plasma membrane]-CandidateSet-hasMember|R-HSA-9700083-kinase domain ALK mutants [plasma membrane]-CandidateSet-hasMember R-HSA-9700186|R-HSA-9700184 Autophosphorylation of point mutants of ALK|Ligand-independent dimerization of point mutants of ALK pubmed:18724359|pubmed:29455642|pubmed:18923525|pubmed:23239810|pubmed:24060861|pubmed:21847362|pubmed:25421750|pubmed:18923524|pubmed:18923523|pubmed:21596819|pubmed:21838707|pubmed:27573755|pubmed:23104988|pubmed:23201355 R-HSA-9725370 Signaling by ALK fusions and activated point mutants R-HSA-201521|R-HSA-201519 ALK autophosphorylation downstream of PTN and MDK|ALK:PTN dimerization Signaling by ALK R-HSA-201556 missense NA

PS3 ALK ALK E1384K [plasma membrane] R-HSA-9715262 ALK UniProt:Q9UM73 L-glutamic acid 1384 replaced with L-lysine ReplacedResidue COSMIC:COSV66589093 anaplastic large cell lymphoma|cervical squamous cell carcinoma DOID:0050744|DOID:3744 pubmed:21847362 R-HSA-9700082-kinase domain ALK mutant dimers [plasma membrane]-CandidateSet-hasMember|R-HSA-9700083-kinase domain ALK mutants [plasma membrane]-CandidateSet-hasMember R-HSA-9700186|R-HSA-9700184 Autophosphorylation of point mutants of ALK|Ligand-independent dimerization of point mutants of ALK pubmed:18724359|pubmed:29455642|pubmed:18923525|pubmed:23239810|pubmed:24060861|pubmed:21847362|pubmed:25421750|pubmed:18923524|pubmed:18923523|pubmed:21596819|pubmed:21838707|pubmed:27573755|pubmed:23104988|pubmed:23201355 R-HSA-9725370 Signaling by ALK fusions and activated point mutants R-HSA-201521|R-HSA-201519 ALK autophosphorylation downstream of PTN and MDK|ALK:PTN dimerization Signaling by ALK R-HSA-201556 missense NA

PM1-PM4 ALK ALK R1275* [plasma membrane] R-HSA-9699913 ALK UniProt:Q9UM73 Nonsense mutation at L-arginine 1275 NonsenseMutation COSMIC:COSV66557562 astrocytoma DOID:3069 R-HSA-9700082-kinase domain ALK mutant dimers [plasma membrane]-CandidateSet-hasCandidate|R-HSA-9700083-kinase domain ALK mutants [plasma membrane]-CandidateSet-hasCandidate R-HSA-9700186|R-HSA-9700184 Autophosphorylation of point mutants of ALK|Ligand-independent dimerization of point mutants of ALK pubmed:18724359|pubmed:29455642|pubmed:18923525|pubmed:23239810|pubmed:24060861|pubmed:21847362|pubmed:25421750|pubmed:18923524|pubmed:18923523|pubmed:21596819|pubmed:21838707|pubmed:27573755|pubmed:23104988|pubmed:23201355 R-HSA-9725370 Signaling by ALK fusions and activated point mutants R-HSA-201521|R-HSA-201519 ALK autophosphorylation downstream of PTN and MDK|ALK:PTN dimerization Signaling by ALK R-HSA-201556 nonsense NA

PM5-PS1 ALK ALK G1201R [plasma membrane] R-HSA-9701841 ALK UniProt:Q9UM73 glycine 1201 replaced with L-arginine ReplacedResidue COSMIC:COSM7002298|COSMIC:COSV66565301 melanoma DOID:1909 R-HSA-9700082-kinase domain ALK mutant dimers [plasma membrane]-CandidateSet-hasCandidate|R-HSA-9700083-kinase domain ALK mutants [plasma membrane]-CandidateSet-hasCandidate R-HSA-9700186|R-HSA-9700184 Autophosphorylation of point mutants of ALK|Ligand-independent dimerization of point mutants of ALK pubmed:18724359|pubmed:29455642|pubmed:18923525|pubmed:23239810|pubmed:24060861|pubmed:21847362|pubmed:25421750|pubmed:18923524|pubmed:18923523|pubmed:21596819|pubmed:21838707|pubmed:27573755|pubmed:23104988|pubmed:23201355 R-HSA-9725370 Signaling by ALK fusions and activated point mutants R-HSA-201521|R-HSA-201519 ALK autophosphorylation downstream of PTN and MDK|ALK:PTN dimerization Signaling by ALK R-HSA-201556 missense NA

PM5 ALK ALK R1275L [plasma membrane] R-HSA-9699841 ALK UniProt:Q9UM73 L-arginine 1275 replaced with L-leucine ReplacedResidue COSMIC:COSV66558183 neuroblastoma DOID:769 R-HSA-9700082-kinase domain ALK mutant dimers [plasma membrane]-CandidateSet-hasCandidate|R-HSA-9700083-kinase domain ALK mutants [plasma membrane]-CandidateSet-hasCandidate R-HSA-9700186|R-HSA-9700184 Autophosphorylation of point mutants of ALK|Ligand-independent dimerization of point mutants of ALK pubmed:18724359|pubmed:29455642|pubmed:18923525|pubmed:23239810|pubmed:24060861|pubmed:21847362|pubmed:25421750|pubmed:18923524|pubmed:18923523|pubmed:21596819|pubmed:21838707|pubmed:27573755|pubmed:23104988|pubmed:23201355 R-HSA-9725370 Signaling by ALK fusions and activated point mutants R-HSA-201521|R-HSA-201519 ALK autophosphorylation downstream of PTN and MDK|ALK:PTN dimerization Signaling by ALK R-HSA-201556 missense NA

PM5 ALK ALK M1166N [plasma membrane] R-HSA-9723293 ALK UniProt:Q9UM73 L-methionine 1166 replaced with L-asparagine ReplacedResidue COSMIC:COSV66574940 neuroblastoma DOID:769 pubmed:23104988 R-HSA-9700082-kinase domain ALK mutant dimers [plasma membrane]-CandidateSet-hasCandidate|R-HSA-9700083-kinase domain ALK mutants [plasma membrane]-CandidateSet-hasCandidate R-HSA-9700186|R-HSA-9700184 Autophosphorylation of point mutants of ALK|Ligand-independent dimerization of point mutants of ALK pubmed:18724359|pubmed:29455642|pubmed:18923525|pubmed:23239810|pubmed:24060861|pubmed:21847362|pubmed:25421750|pubmed:18923524|pubmed:18923523|pubmed:21596819|pubmed:21838707|pubmed:27573755|pubmed:23104988|pubmed:23201355 R-HSA-9725370 Signaling by ALK fusions and activated point mutants R-HSA-201521|R-HSA-201519 ALK autophosphorylation downstream of PTN and MDK|ALK:PTN dimerization Signaling by ALK R-HSA-201556 missense NA

PM5 ALK ALK M1166T [plasma membrane] R-HSA-9699916 ALK UniProt:Q9UM73 L-methionine 1166 replaced with L-threonine ReplacedResidue COSMIC:COSV66588108 non-small cell lung carcinoma DOID:3908 R-HSA-9700082-kinase domain ALK mutant dimers [plasma membrane]-CandidateSet-hasCandidate|R-HSA-9700083-kinase domain ALK mutants [plasma membrane]-CandidateSet-hasCandidate R-HSA-9700186|R-HSA-9700184 Autophosphorylation of point mutants of ALK|Ligand-independent dimerization of point mutants of ALK pubmed:18724359|pubmed:29455642|pubmed:18923525|pubmed:23239810|pubmed:24060861|pubmed:21847362|pubmed:25421750|pubmed:18923524|pubmed:18923523|pubmed:21596819|pubmed:21838707|pubmed:27573755|pubmed:23104988|pubmed:23201355 R-HSA-9725370 Signaling by ALK fusions and activated point mutants R-HSA-201521|R-HSA-201519 ALK autophosphorylation downstream of PTN and MDK|ALK:PTN dimerization Signaling by ALK R-HSA-201556 missense NA

PM5 ALK ALK G1128V [plasma membrane] R-HSA-9699797 ALK UniProt:Q9UM73 glycine 1128 replaced with L-valine ReplacedResidue COSMIC:COSV101202380 melanoma DOID:1909 R-HSA-9700082-kinase domain ALK mutant dimers [plasma membrane]-CandidateSet-hasCandidate|R-HSA-9700083-kinase domain ALK mutants [plasma membrane]-CandidateSet-hasCandidate R-HSA-9700186|R-HSA-9700184 Autophosphorylation of point mutants of ALK|Ligand-independent dimerization of point mutants of ALK pubmed:18724359|pubmed:29455642|pubmed:18923525|pubmed:23239810|pubmed:24060861|pubmed:21847362|pubmed:25421750|pubmed:18923524|pubmed:18923523|pubmed:21596819|pubmed:21838707|pubmed:27573755|pubmed:23104988|pubmed:23201355 R-HSA-9725370 Signaling by ALK fusions and activated point mutants R-HSA-201521|R-HSA-201519 ALK autophosphorylation downstream of PTN and MDK|ALK:PTN dimerization Signaling by ALK R-HSA-201556 missense NA

PM5-PS1 ALK ALK G1128S [plasma membrane] R-HSA-9699840 ALK UniProt:Q9UM73 glycine 1128 replaced with L-serine ReplacedResidue COSMIC:COSV66567934|COSMIC:COSM5352215 non-small cell lung carcinoma DOID:3908 R-HSA-9700082-kinase domain ALK mutant dimers [plasma membrane]-CandidateSet-hasCandidate|R-HSA-9700083-kinase domain ALK mutants [plasma membrane]-CandidateSet-hasCandidate R-HSA-9700186|R-HSA-9700184 Autophosphorylation of point mutants of ALK|Ligand-independent dimerization of point mutants of ALK pubmed:18724359|pubmed:29455642|pubmed:18923525|pubmed:23239810|pubmed:24060861|pubmed:21847362|pubmed:25421750|pubmed:18923524|pubmed:18923523|pubmed:21596819|pubmed:21838707|pubmed:27573755|pubmed:23104988|pubmed:23201355 R-HSA-9725370 Signaling by ALK fusions and activated point mutants R-HSA-201521|R-HSA-201519 ALK autophosphorylation downstream of PTN and MDK|ALK:PTN dimerization Signaling by ALK R-HSA-201556 missense NA

PM5 ALK ALK Y1239H [plasma membrane] R-HSA-9715266 ALK UniProt:Q9UM73 L-tyrosine 1239 replaced with L-histidine ReplacedResidue anaplastic large cell lymphoma DOID:0050744 pubmed:21847362 R-HSA-9700082-kinase domain ALK mutant dimers [plasma membrane]-CandidateSet-hasCandidate|R-HSA-9700083-kinase domain ALK mutants [plasma membrane]-CandidateSet-hasCandidate R-HSA-9700186|R-HSA-9700184 Autophosphorylation of point mutants of ALK|Ligand-independent dimerization of point mutants of ALK pubmed:18724359|pubmed:29455642|pubmed:18923525|pubmed:23239810|pubmed:24060861|pubmed:21847362|pubmed:25421750|pubmed:18923524|pubmed:18923523|pubmed:21596819|pubmed:21838707|pubmed:27573755|pubmed:23104988|pubmed:23201355 R-HSA-9725370 Signaling by ALK fusions and activated point mutants R-HSA-201521|R-HSA-201519 ALK autophosphorylation downstream of PTN and MDK|ALK:PTN dimerization Signaling by ALK R-HSA-201556 missense NA

PM5 ALK ALK F1174S [plasma membrane] R-HSA-9699880 ALK UniProt:Q9UM73 L-phenylalanine 1174 replaced with L-serine ReplacedResidue COSMIC:COSV66558767 endometrial cancer|neuroblastoma DOID:1380|DOID:769 R-HSA-9700082-kinase domain ALK mutant dimers [plasma membrane]-CandidateSet-hasCandidate|R-HSA-9700083-kinase domain ALK mutants [plasma membrane]-CandidateSet-hasCandidate R-HSA-9700186|R-HSA-9700184 Autophosphorylation of point mutants of ALK|Ligand-independent dimerization of point mutants of ALK pubmed:18724359|pubmed:29455642|pubmed:18923525|pubmed:23239810|pubmed:24060861|pubmed:21847362|pubmed:25421750|pubmed:18923524|pubmed:18923523|pubmed:21596819|pubmed:21838707|pubmed:27573755|pubmed:23104988|pubmed:23201355 R-HSA-9725370 Signaling by ALK fusions and activated point mutants R-HSA-201521|R-HSA-201519 ALK autophosphorylation downstream of PTN and MDK|ALK:PTN dimerization Signaling by ALK R-HSA-201556 missense NA

PM1-PM4 AMER1 AMER1 R497* [cytosol] R-HSA-9663943 AMER1 UniProt:Q5JTC6 Nonsense mutation at L-arginine 497 NonsenseMutation COSMIC:COSV57655167 colon adenocarcinoma|cancer DOID:234|DOID:162 pubmed:26071483 R-HSA-4839741-truncated AMER1 mutants [cytosol]-CandidateSet-hasCandidate R-HSA-4839746 Truncated AMER1 mutants destabilize the destruction complex loss_of_function pubmed:19760609|pubmed:21248786|pubmed:17510365|pubmed:18311776 R-HSA-5467348 Truncations of AMER1 destabilize the destruction complex R-HSA-195251 Assembly of the destruction complex Degradation of beta-catenin by the destruction complex R-HSA-195253 1904885 beta-catenin destruction complex assembly nonsense NA

PM1-PM4 AMER1 AMER1 S210* [cytosol] R-HSA-5251492 AMER1 UniProt:Q5JTC6 Nonsense mutation at L-serine 210 NonsenseMutation COSMIC:COSV57655668 kidney cancer|nephroblastoma DOID:263|DOID:2154 pubmed:18311776 R-HSA-4839741-truncated AMER1 mutants [cytosol]-CandidateSet-hasCandidate R-HSA-4839746 Truncated AMER1 mutants destabilize the destruction complex loss_of_function pubmed:19760609|pubmed:21248786|pubmed:17510365|pubmed:18311776 R-HSA-5467348 Truncations of AMER1 destabilize the destruction complex R-HSA-195251 Assembly of the destruction complex Degradation of beta-catenin by the destruction complex R-HSA-195253 1904885 beta-catenin destruction complex assembly nonsense NA

PM1-PM4-PS1 AMER1 AMER1 R358* [cytosol] R-HSA-4839640 AMER1 UniProt:Q5JTC6 Nonsense mutation at L-arginine 358 NonsenseMutation COSMIC:COSM30811|COSMIC:COSV57654223 colorectal cancer|cancer|nephroblastoma DOID:9256|DOID:162|DOID:2154 R-HSA-4839741-truncated AMER1 mutants [cytosol]-CandidateSet-hasCandidate R-HSA-4839746 Truncated AMER1 mutants destabilize the destruction complex loss_of_function pubmed:19760609|pubmed:21248786|pubmed:17510365|pubmed:18311776 R-HSA-5467348 Truncations of AMER1 destabilize the destruction complex R-HSA-195251 Assembly of the destruction complex Degradation of beta-catenin by the destruction complex R-HSA-195253 1904885 beta-catenin destruction complex assembly nonsense NA

PM1-PM4 AMER1 AMER1 R353* [cytosol] R-HSA-9663939 AMER1 UniProt:Q5JTC6 Nonsense mutation at L-arginine 353 NonsenseMutation COSMIC:COSV57654256 colorectal cancer|colon adenocarcinoma|cancer DOID:9256|DOID:234|DOID:162 pubmed:19760609 R-HSA-4839741-truncated AMER1 mutants [cytosol]-CandidateSet-hasCandidate R-HSA-4839746 Truncated AMER1 mutants destabilize the destruction complex loss_of_function pubmed:19760609|pubmed:21248786|pubmed:17510365|pubmed:18311776 R-HSA-5467348 Truncations of AMER1 destabilize the destruction complex R-HSA-195251 Assembly of the destruction complex Degradation of beta-catenin by the destruction complex R-HSA-195253 1904885 beta-catenin destruction complex assembly nonsense NA

PS3 AMN AMN T41I [plasma membrane] R-HSA-3299658 AMN UniProt:Q9BXJ7 L-threonine 41 replaced with L-isoleucine ReplacedResidue megaloblastic anemia DOID:13382 R-HSA-3299662-AMN mutants [plasma membrane]-DefinedSet-hasMember R-HSA-3296477 Defective AMN does not transport GIF:Cbl loss_of_function pubmed:13852753|pubmed:12590260|pubmed:13828999 R-HSA-3359462 Defective AMN causes MGA1 R-HSA-3000103 CUBN:AMN binds CBLIF:RCbl Metabolism of water-soluble vitamins and cofactors R-HSA-196849 6767 water-soluble vitamin metabolic process missense loss_of_function of water-soluble vitamin metabolic process

PS3 AMN AMN G5Afs*12 [plasma membrane] R-HSA-3296484 AMN UniProt:Q9BXJ7 Replacement of residues 5 to 15 by AGSCCGCSSAH FragmentReplacedModification megaloblastic anemia DOID:13382 R-HSA-3299662-AMN mutants [plasma membrane]-DefinedSet-hasMember R-HSA-3296477 Defective AMN does not transport GIF:Cbl loss_of_function pubmed:13852753|pubmed:12590260|pubmed:13828999 R-HSA-3359462 Defective AMN causes MGA1 R-HSA-3000103 CUBN:AMN binds CBLIF:RCbl Metabolism of water-soluble vitamins and cofactors R-HSA-196849 6767 water-soluble vitamin metabolic process frameshift loss_of_function of water-soluble vitamin metabolic process

PM5 AP3B1 AP3B1(1-859)-p-BRAF(381-766) fusion [cytosol] R-HSA-6802516 AP3B1 UniProt:O00203 Insertion of residues 381 to 766 at 860 from UniProt:P15056 BRAF FragmentInsertionModification thyroid carcinoma|cancer DOID:3963|DOID:162 pubmed:25204415 R-HSA-6802698-p-BRAF/RAF fusion dimers [cytosol]-CandidateSet-hasCandidate R-HSA-6802935|R-HSA-6802933|R-HSA-6802934|R-HSA-6802927|R-HSA-6802932 MAPKs are phosphorylated downstream of BRAF and RAF fusion dimers|p-BRAF and RAF fusion dimers phosphorylate MAP2Ks|p-BRAF and RAF fusion dimers bind MAP2Ks and MAPKs|BRAF and RAF fusion mutant dimers are phosphorylated|Dissociation of BRAF/RAF fusion complex gain_of_function pubmed:24345920|pubmed:15630448|pubmed:25907612|pubmed:21424530|pubmed:20526349|pubmed:18974108|pubmed:24135138|pubmed:25204415|pubmed:22745804|pubmed:23583981 R-HSA-6802952 Signaling by BRAF and RAF1 fusions R-HSA-5672973|R-HSA-5672978|R-HSA-5672972|R-HSA-5672969|R-HSA-5672980 MAP2Ks phosphorylate MAPKs|RAF phosphorylates MAP2K dimer|MAP2Ks and MAPKs bind to the activated RAF complex|Phosphorylation of RAF|Dissociation of RAS:RAF complex RAF/MAP kinase cascade R-HSA-5673001 165 MAPK cascade fusion gain_of_function of MAPK cascade

PM5 AP3B1 AP3B1(1-859)-BRAF(381-766) fusion [cytosol] R-HSA-6802269 AP3B1 UniProt:O00203 Insertion of residues 381 to 766 at 860 from UniProt:P15056 BRAF FragmentInsertionModification thyroid carcinoma|cancer DOID:3963|DOID:162 pubmed:25204415 R-HSA-6802720-BRAF/RAF fusion dimers [cytosol]-CandidateSet-hasCandidate R-HSA-6802927 BRAF and RAF fusion mutant dimers are phosphorylated gain_of_function pubmed:24345920|pubmed:15630448|pubmed:25907612|pubmed:21424530|pubmed:20526349|pubmed:18974108|pubmed:24135138|pubmed:25204415|pubmed:22745804|pubmed:23583981 R-HSA-6802952 Signaling by BRAF and RAF1 fusions R-HSA-5672969 Phosphorylation of RAF RAF/MAP kinase cascade R-HSA-5673001 165 MAPK cascade fusion gain_of_function of MAPK cascade

PS3 APC APC F1197* [cytosol] R-HSA-5246655 APC UniProt:P25054 Nonsense mutation at L-phenylalanine 1197 NonsenseMutation colorectal cancer DOID:9256 R-HSA-5246650-APC polyubiquitination mutants [cytosol]-DefinedSet-hasMember R-HSA-5246696 APC truncation mutants are not K63 polyubiquitinated loss_of_function pubmed:22761442 R-HSA-5467333 APC truncation mutants are not K63 polyubiquitinated R-HSA-5246693 APC is K63-polyubiquitinated Degradation of beta-catenin by the destruction complex R-HSA-195253 nonsense NA

PS3 APC APC Q1338* [cytosol] R-HSA-5246654 APC UniProt:P25054 Nonsense mutation at L-glutamine 1338 NonsenseMutation COSMIC:COSV57325971 colorectal cancer DOID:9256 R-HSA-5246650-APC polyubiquitination mutants [cytosol]-DefinedSet-hasMember|R-HSA-4791256-APC truncation mutants [cytosol]-CandidateSet-hasMember R-HSA-5246696|R-HSA-4791278 APC truncation mutants are not K63 polyubiquitinated|APC truncation mutants have impaired AXIN binding loss_of_function pubmed:22761442|pubmed:10749138|pubmed:8757136|pubmed:11522655|pubmed:9065402|pubmed:22438566|pubmed:10921899|pubmed:23256519|pubmed:9601641|pubmed:8638126|pubmed:9556553|pubmed:8259519|pubmed:8259518 R-HSA-5467333|R-HSA-5467337 APC truncation mutants are not K63 polyubiquitinated|APC truncation mutants have impaired AXIN binding R-HSA-5246693|R-HSA-195251 APC is K63-polyubiquitinated|Assembly of the destruction complex Degradation of beta-catenin by the destruction complex R-HSA-195253 1904885 beta-catenin destruction complex assembly nonsense NA

PS3 APC APC S811* [cytosol] R-HSA-5246652 APC UniProt:P25054 Nonsense mutation at L-serine 811 NonsenseMutation COSMIC:COSV57338557 colorectal cancer DOID:9256 R-HSA-5246650-APC polyubiquitination mutants [cytosol]-DefinedSet-hasMember R-HSA-5246696 APC truncation mutants are not K63 polyubiquitinated loss_of_function pubmed:22761442 R-HSA-5467333 APC truncation mutants are not K63 polyubiquitinated R-HSA-5246693 APC is K63-polyubiquitinated Degradation of beta-catenin by the destruction complex R-HSA-195253 nonsense NA

PS3 APC APC I1417 fs*2 [cytosol] R-HSA-5246651 APC UniProt:P25054 Replacement of residues 1417 to 1417 by L FragmentReplacedModification colorectal cancer DOID:9256 R-HSA-5246650-APC polyubiquitination mutants [cytosol]-DefinedSet-hasMember|R-HSA-4791256-APC truncation mutants [cytosol]-CandidateSet-hasMember R-HSA-5246696|R-HSA-4791278 APC truncation mutants are not K63 polyubiquitinated|APC truncation mutants have impaired AXIN binding loss_of_function pubmed:22761442|pubmed:10749138|pubmed:8757136|pubmed:11522655|pubmed:9065402|pubmed:22438566|pubmed:10921899|pubmed:23256519|pubmed:9601641|pubmed:8638126|pubmed:9556553|pubmed:8259519|pubmed:8259518 R-HSA-5467333|R-HSA-5467337 APC truncation mutants are not K63 polyubiquitinated|APC truncation mutants have impaired AXIN binding R-HSA-5246693|R-HSA-195251 APC is K63-polyubiquitinated|Assembly of the destruction complex Degradation of beta-catenin by the destruction complex R-HSA-195253 1904885 beta-catenin destruction complex assembly frameshift NA

PS3 APC APC E1554* [cytosol] R-HSA-5246648 APC UniProt:P25054 Nonsense mutation at L-glutamic acid 1554 NonsenseMutation COSMIC:COSV57332231 colorectal cancer DOID:9256 R-HSA-5246650-APC polyubiquitination mutants [cytosol]-DefinedSet-hasMember R-HSA-5246696 APC truncation mutants are not K63 polyubiquitinated loss_of_function pubmed:22761442 R-HSA-5467333 APC truncation mutants are not K63 polyubiquitinated R-HSA-5246693 APC is K63-polyubiquitinated Degradation of beta-catenin by the destruction complex R-HSA-195253 nonsense NA

PM1-PM4 APC APC R213* [cytosol] R-HSA-9666924 APC UniProt:P25054 Nonsense mutation at L-arginine 213 NonsenseMutation COSMIC:COSV57321978 colorectal cancer DOID:9256 R-HSA-4791256-APC truncation mutants [cytosol]-CandidateSet-hasCandidate R-HSA-4791278 APC truncation mutants have impaired AXIN binding loss_of_function pubmed:10749138|pubmed:8757136|pubmed:11522655|pubmed:9065402|pubmed:22438566|pubmed:10921899|pubmed:23256519|pubmed:9601641|pubmed:8638126|pubmed:9556553|pubmed:8259519|pubmed:8259518 R-HSA-5467337 APC truncation mutants have impaired AXIN binding R-HSA-195251 Assembly of the destruction complex Degradation of beta-catenin by the destruction complex R-HSA-195253 1904885 beta-catenin destruction complex assembly nonsense NA

PM1-PM4 APC APC C1410* [cytosol] R-HSA-9666925 APC UniProt:P25054 Nonsense mutation at L-cysteine 1410 NonsenseMutation COSMIC:COSV57322030 colorectal cancer DOID:9256 R-HSA-4791256-APC truncation mutants [cytosol]-CandidateSet-hasCandidate R-HSA-4791278 APC truncation mutants have impaired AXIN binding loss_of_function pubmed:10749138|pubmed:8757136|pubmed:11522655|pubmed:9065402|pubmed:22438566|pubmed:10921899|pubmed:23256519|pubmed:9601641|pubmed:8638126|pubmed:9556553|pubmed:8259519|pubmed:8259518 R-HSA-5467337 APC truncation mutants have impaired AXIN binding R-HSA-195251 Assembly of the destruction complex Degradation of beta-catenin by the destruction complex R-HSA-195253 1904885 beta-catenin destruction complex assembly nonsense NA

PM1-PM4 APC APC K1310* [cytosol] R-HSA-9666926 APC UniProt:P25054 Nonsense mutation at L-lysine 1310 NonsenseMutation COSMIC:COSV57357132 colorectal cancer DOID:9256 R-HSA-4791256-APC truncation mutants [cytosol]-CandidateSet-hasCandidate R-HSA-4791278 APC truncation mutants have impaired AXIN binding loss_of_function pubmed:10749138|pubmed:8757136|pubmed:11522655|pubmed:9065402|pubmed:22438566|pubmed:10921899|pubmed:23256519|pubmed:9601641|pubmed:8638126|pubmed:9556553|pubmed:8259519|pubmed:8259518 R-HSA-5467337 APC truncation mutants have impaired AXIN binding R-HSA-195251 Assembly of the destruction complex Degradation of beta-catenin by the destruction complex R-HSA-195253 1904885 beta-catenin destruction complex assembly nonsense NA

PM1-PM4 APC APC R499* [cytosol] R-HSA-9666927 APC UniProt:P25054 Nonsense mutation at L-arginine 499 NonsenseMutation COSMIC:COSV57322064 colorectal cancer DOID:9256 R-HSA-4791256-APC truncation mutants [cytosol]-CandidateSet-hasCandidate R-HSA-4791278 APC truncation mutants have impaired AXIN binding loss_of_function pubmed:10749138|pubmed:8757136|pubmed:11522655|pubmed:9065402|pubmed:22438566|pubmed:10921899|pubmed:23256519|pubmed:9601641|pubmed:8638126|pubmed:9556553|pubmed:8259519|pubmed:8259518 R-HSA-5467337 APC truncation mutants have impaired AXIN binding R-HSA-195251 Assembly of the destruction complex Degradation of beta-catenin by the destruction complex R-HSA-195253 1904885 beta-catenin destruction complex assembly nonsense NA

PM1-PM4 APC APC R1450* [cytosol] R-HSA-5251499 APC UniProt:P25054 Nonsense mutation at L-arginine 1450 NonsenseMutation COSMIC:COSV57321313 colorectal cancer DOID:9256 pubmed:11746989|pubmed:1338904 R-HSA-4791256-APC truncation mutants [cytosol]-CandidateSet-hasCandidate R-HSA-4791278 APC truncation mutants have impaired AXIN binding loss_of_function pubmed:10749138|pubmed:8757136|pubmed:11522655|pubmed:9065402|pubmed:22438566|pubmed:10921899|pubmed:23256519|pubmed:9601641|pubmed:8638126|pubmed:9556553|pubmed:8259519|pubmed:8259518 R-HSA-5467337 APC truncation mutants have impaired AXIN binding R-HSA-195251 Assembly of the destruction complex Degradation of beta-catenin by the destruction complex R-HSA-195253 1904885 beta-catenin destruction complex assembly nonsense NA

PM1-PM4 APC APC G1357* [cytosol] R-HSA-9666940 APC UniProt:P25054 Nonsense mutation at glycine 1357 NonsenseMutation COSMIC:COSV57321275 colorectal cancer DOID:9256 R-HSA-4791256-APC truncation mutants [cytosol]-CandidateSet-hasCandidate R-HSA-4791278 APC truncation mutants have impaired AXIN binding loss_of_function pubmed:10749138|pubmed:8757136|pubmed:11522655|pubmed:9065402|pubmed:22438566|pubmed:10921899|pubmed:23256519|pubmed:9601641|pubmed:8638126|pubmed:9556553|pubmed:8259519|pubmed:8259518 R-HSA-5467337 APC truncation mutants have impaired AXIN binding R-HSA-195251 Assembly of the destruction complex Degradation of beta-catenin by the destruction complex R-HSA-195253 1904885 beta-catenin destruction complex assembly nonsense NA

PM1-PM4-PS1 APC APC L1488* [cytosol] R-HSA-9666941 APC UniProt:P25054 Nonsense mutation at L-leucine 1488 NonsenseMutation COSMIC:COSV57360736|COSMIC:COSV57323073 colorectal cancer DOID:9256 R-HSA-4791256-APC truncation mutants [cytosol]-CandidateSet-hasCandidate R-HSA-4791278 APC truncation mutants have impaired AXIN binding loss_of_function pubmed:10749138|pubmed:8757136|pubmed:11522655|pubmed:9065402|pubmed:22438566|pubmed:10921899|pubmed:23256519|pubmed:9601641|pubmed:8638126|pubmed:9556553|pubmed:8259519|pubmed:8259518 R-HSA-5467337 APC truncation mutants have impaired AXIN binding R-HSA-195251 Assembly of the destruction complex Degradation of beta-catenin by the destruction complex R-HSA-195253 1904885 beta-catenin destruction complex assembly nonsense NA

PM1-PM4 APC APC S1400* [cytosol] R-HSA-9666942 APC UniProt:P25054 Nonsense mutation at L-serine 1400 NonsenseMutation COSMIC:COSV57326621 colorectal cancer DOID:9256 R-HSA-4791256-APC truncation mutants [cytosol]-CandidateSet-hasCandidate R-HSA-4791278 APC truncation mutants have impaired AXIN binding loss_of_function pubmed:10749138|pubmed:8757136|pubmed:11522655|pubmed:9065402|pubmed:22438566|pubmed:10921899|pubmed:23256519|pubmed:9601641|pubmed:8638126|pubmed:9556553|pubmed:8259519|pubmed:8259518 R-HSA-5467337 APC truncation mutants have impaired AXIN binding R-HSA-195251 Assembly of the destruction complex Degradation of beta-catenin by the destruction complex R-HSA-195253 1904885 beta-catenin destruction complex assembly nonsense NA

PM1-PM4-PS1 APC APC S1346* [cytosol] R-HSA-9666943 APC UniProt:P25054 Nonsense mutation at L-serine 1346 NonsenseMutation COSMIC:COSV57328373|COSMIC:COSV57325995 colorectal cancer DOID:9256 R-HSA-4791256-APC truncation mutants [cytosol]-CandidateSet-hasCandidate R-HSA-4791278 APC truncation mutants have impaired AXIN binding loss_of_function pubmed:10749138|pubmed:8757136|pubmed:11522655|pubmed:9065402|pubmed:22438566|pubmed:10921899|pubmed:23256519|pubmed:9601641|pubmed:8638126|pubmed:9556553|pubmed:8259519|pubmed:8259518 R-HSA-5467337 APC truncation mutants have impaired AXIN binding R-HSA-195251 Assembly of the destruction complex Degradation of beta-catenin by the destruction complex R-HSA-195253 1904885 beta-catenin destruction complex assembly nonsense NA

PM1-PM4 APC APC R405* [cytosol] R-HSA-9666936 APC UniProt:P25054 Nonsense mutation at L-arginine 405 NonsenseMutation COSMIC:COSV57322404 colorectal cancer DOID:9256 R-HSA-4791256-APC truncation mutants [cytosol]-CandidateSet-hasCandidate R-HSA-4791278 APC truncation mutants have impaired AXIN binding loss_of_function pubmed:10749138|pubmed:8757136|pubmed:11522655|pubmed:9065402|pubmed:22438566|pubmed:10921899|pubmed:23256519|pubmed:9601641|pubmed:8638126|pubmed:9556553|pubmed:8259519|pubmed:8259518 R-HSA-5467337 APC truncation mutants have impaired AXIN binding R-HSA-195251 Assembly of the destruction complex Degradation of beta-catenin by the destruction complex R-HSA-195253 1904885 beta-catenin destruction complex assembly nonsense NA

PM1-PM4-PS1 APC APC S1281* [cytosol] R-HSA-9666937 APC UniProt:P25054 Nonsense mutation at L-serine 1281 NonsenseMutation COSMIC:COSV57330499|COSMIC:COSV57326196 colorectal cancer DOID:9256 R-HSA-4791256-APC truncation mutants [cytosol]-CandidateSet-hasCandidate R-HSA-4791278 APC truncation mutants have impaired AXIN binding loss_of_function pubmed:10749138|pubmed:8757136|pubmed:11522655|pubmed:9065402|pubmed:22438566|pubmed:10921899|pubmed:23256519|pubmed:9601641|pubmed:8638126|pubmed:9556553|pubmed:8259519|pubmed:8259518 R-HSA-5467337 APC truncation mutants have impaired AXIN binding R-HSA-195251 Assembly of the destruction complex Degradation of beta-catenin by the destruction complex R-HSA-195253 1904885 beta-catenin destruction complex assembly nonsense NA

PM1-PM4 APC APC E1573* [cytosol] R-HSA-9666938 APC UniProt:P25054 Nonsense mutation at L-glutamic acid 1573 NonsenseMutation COSMIC:COSV57324336 colorectal cancer DOID:9256 R-HSA-4791256-APC truncation mutants [cytosol]-CandidateSet-hasCandidate R-HSA-4791278 APC truncation mutants have impaired AXIN binding loss_of_function pubmed:10749138|pubmed:8757136|pubmed:11522655|pubmed:9065402|pubmed:22438566|pubmed:10921899|pubmed:23256519|pubmed:9601641|pubmed:8638126|pubmed:9556553|pubmed:8259519|pubmed:8259518 R-HSA-5467337 APC truncation mutants have impaired AXIN binding R-HSA-195251 Assembly of the destruction complex Degradation of beta-catenin by the destruction complex R-HSA-195253 1904885 beta-catenin destruction complex assembly nonsense NA

PM1-PM4 APC APC R805* [cytosol] R-HSA-9666939 APC UniProt:P25054 Nonsense mutation at L-arginine 805 NonsenseMutation COSMIC:COSV57322578 colorectal cancer DOID:9256 R-HSA-4791256-APC truncation mutants [cytosol]-CandidateSet-hasCandidate R-HSA-4791278 APC truncation mutants have impaired AXIN binding loss_of_function pubmed:10749138|pubmed:8757136|pubmed:11522655|pubmed:9065402|pubmed:22438566|pubmed:10921899|pubmed:23256519|pubmed:9601641|pubmed:8638126|pubmed:9556553|pubmed:8259519|pubmed:8259518 R-HSA-5467337 APC truncation mutants have impaired AXIN binding R-HSA-195251 Assembly of the destruction complex Degradation of beta-catenin by the destruction complex R-HSA-195253 1904885 beta-catenin destruction complex assembly nonsense NA

PM1-PM4 APC APC R1386* [cytosol] R-HSA-9666932 APC UniProt:P25054 Nonsense mutation at L-arginine 1386 NonsenseMutation COSMIC:COSV57330977 colorectal cancer DOID:9256 R-HSA-4791256-APC truncation mutants [cytosol]-CandidateSet-hasCandidate R-HSA-4791278 APC truncation mutants have impaired AXIN binding loss_of_function pubmed:10749138|pubmed:8757136|pubmed:11522655|pubmed:9065402|pubmed:22438566|pubmed:10921899|pubmed:23256519|pubmed:9601641|pubmed:8638126|pubmed:9556553|pubmed:8259519|pubmed:8259518 R-HSA-5467337 APC truncation mutants have impaired AXIN binding R-HSA-195251 Assembly of the destruction complex Degradation of beta-catenin by the destruction complex R-HSA-195253 1904885 beta-catenin destruction complex assembly nonsense NA

PM1-PM4-PS1 APC APC S1327* [cytosol] R-HSA-9666933 APC UniProt:P25054 Nonsense mutation at L-serine 1327 NonsenseMutation COSMIC:COSV57335489|COSMIC:COSV57323640 colorectal cancer DOID:9256 R-HSA-4791256-APC truncation mutants [cytosol]-CandidateSet-hasCandidate R-HSA-4791278 APC truncation mutants have impaired AXIN binding loss_of_function pubmed:10749138|pubmed:8757136|pubmed:11522655|pubmed:9065402|pubmed:22438566|pubmed:10921899|pubmed:23256519|pubmed:9601641|pubmed:8638126|pubmed:9556553|pubmed:8259519|pubmed:8259518 R-HSA-5467337 APC truncation mutants have impaired AXIN binding R-HSA-195251 Assembly of the destruction complex Degradation of beta-catenin by the destruction complex R-HSA-195253 1904885 beta-catenin destruction complex assembly nonsense NA

PM1-PM4 APC APC E1209* [cytosol] R-HSA-9666934 APC UniProt:P25054 Nonsense mutation at L-glutamic acid 1209 NonsenseMutation COSMIC:COSV57327136 colorectal cancer DOID:9256 R-HSA-4791256-APC truncation mutants [cytosol]-CandidateSet-hasCandidate R-HSA-4791278 APC truncation mutants have impaired AXIN binding loss_of_function pubmed:10749138|pubmed:8757136|pubmed:11522655|pubmed:9065402|pubmed:22438566|pubmed:10921899|pubmed:23256519|pubmed:9601641|pubmed:8638126|pubmed:9556553|pubmed:8259519|pubmed:8259518 R-HSA-5467337 APC truncation mutants have impaired AXIN binding R-HSA-195251 Assembly of the destruction complex Degradation of beta-catenin by the destruction complex R-HSA-195253 1904885 beta-catenin destruction complex assembly nonsense NA

PM1-PM4 APC APC Q1469* [cytosol] R-HSA-9666935 APC UniProt:P25054 Nonsense mutation at L-asparagine 1469 NonsenseMutation COSMIC:COSV57332774 colorectal cancer DOID:9256 R-HSA-4791256-APC truncation mutants [cytosol]-CandidateSet-hasCandidate R-HSA-4791278 APC truncation mutants have impaired AXIN binding loss_of_function pubmed:10749138|pubmed:8757136|pubmed:11522655|pubmed:9065402|pubmed:22438566|pubmed:10921899|pubmed:23256519|pubmed:9601641|pubmed:8638126|pubmed:9556553|pubmed:8259519|pubmed:8259518 R-HSA-5467337 APC truncation mutants have impaired AXIN binding R-HSA-195251 Assembly of the destruction complex Degradation of beta-catenin by the destruction complex R-HSA-195253 1904885 beta-catenin destruction complex assembly nonsense NA

PM1-PM4 APC APC R564* [cytosol] R-HSA-9666928 APC UniProt:P25054 Nonsense mutation at L-arginine 564 NonsenseMutation COSMIC:COSV57321509 colorectal cancer DOID:9256 R-HSA-4791256-APC truncation mutants [cytosol]-CandidateSet-hasCandidate R-HSA-4791278 APC truncation mutants have impaired AXIN binding loss_of_function pubmed:10749138|pubmed:8757136|pubmed:11522655|pubmed:9065402|pubmed:22438566|pubmed:10921899|pubmed:23256519|pubmed:9601641|pubmed:8638126|pubmed:9556553|pubmed:8259519|pubmed:8259518 R-HSA-5467337 APC truncation mutants have impaired AXIN binding R-HSA-195251 Assembly of the destruction complex Degradation of beta-catenin by the destruction complex R-HSA-195253 1904885 beta-catenin destruction complex assembly nonsense NA

PM1-PM4 APC APC E1536* [cytosol] R-HSA-9666929 APC UniProt:P25054 Nonsense mutation at L-glutamic acid 1536 NonsenseMutation COSMIC:COSV57330094 colorectal cancer DOID:9256 R-HSA-4791256-APC truncation mutants [cytosol]-CandidateSet-hasCandidate R-HSA-4791278 APC truncation mutants have impaired AXIN binding loss_of_function pubmed:10749138|pubmed:8757136|pubmed:11522655|pubmed:9065402|pubmed:22438566|pubmed:10921899|pubmed:23256519|pubmed:9601641|pubmed:8638126|pubmed:9556553|pubmed:8259519|pubmed:8259518 R-HSA-5467337 APC truncation mutants have impaired AXIN binding R-HSA-195251 Assembly of the destruction complex Degradation of beta-catenin by the destruction complex R-HSA-195253 1904885 beta-catenin destruction complex assembly nonsense NA

PM1-PM4 APC APC G1288* [cytosol] R-HSA-9666930 APC UniProt:P25054 Nonsense mutation at glycine 1288 NonsenseMutation COSMIC:COSV57327258 colorectal cancer DOID:9256 R-HSA-4791256-APC truncation mutants [cytosol]-CandidateSet-hasCandidate R-HSA-4791278 APC truncation mutants have impaired AXIN binding loss_of_function pubmed:10749138|pubmed:8757136|pubmed:11522655|pubmed:9065402|pubmed:22438566|pubmed:10921899|pubmed:23256519|pubmed:9601641|pubmed:8638126|pubmed:9556553|pubmed:8259519|pubmed:8259518 R-HSA-5467337 APC truncation mutants have impaired AXIN binding R-HSA-195251 Assembly of the destruction complex Degradation of beta-catenin by the destruction complex R-HSA-195253 1904885 beta-catenin destruction complex assembly nonsense NA

PM1-PM4 APC APC G1312* [cytosol] R-HSA-9666931 APC UniProt:P25054 Nonsense mutation at glycine 1312 NonsenseMutation COSMIC:COSV57321523 colorectal cancer DOID:9256 R-HSA-4791256-APC truncation mutants [cytosol]-CandidateSet-hasCandidate R-HSA-4791278 APC truncation mutants have impaired AXIN binding loss_of_function pubmed:10749138|pubmed:8757136|pubmed:11522655|pubmed:9065402|pubmed:22438566|pubmed:10921899|pubmed:23256519|pubmed:9601641|pubmed:8638126|pubmed:9556553|pubmed:8259519|pubmed:8259518 R-HSA-5467337 APC truncation mutants have impaired AXIN binding R-HSA-195251 Assembly of the destruction complex Degradation of beta-catenin by the destruction complex R-HSA-195253 1904885 beta-catenin destruction complex assembly nonsense NA

PM1-PM4 APC APC R283* [cytosol] R-HSA-9666988 APC UniProt:P25054 Nonsense mutation at L-arginine 283 NonsenseMutation COSMIC:COSV57325157 colorectal cancer DOID:9256 R-HSA-4791256-APC truncation mutants [cytosol]-CandidateSet-hasCandidate R-HSA-4791278 APC truncation mutants have impaired AXIN binding loss_of_function pubmed:10749138|pubmed:8757136|pubmed:11522655|pubmed:9065402|pubmed:22438566|pubmed:10921899|pubmed:23256519|pubmed:9601641|pubmed:8638126|pubmed:9556553|pubmed:8259519|pubmed:8259518 R-HSA-5467337 APC truncation mutants have impaired AXIN binding R-HSA-195251 Assembly of the destruction complex Degradation of beta-catenin by the destruction complex R-HSA-195253 1904885 beta-catenin destruction complex assembly nonsense NA

PM1-PM4 APC APC Q1367* [cytosol] R-HSA-9666989 APC UniProt:P25054 Nonsense mutation at L-glutamine 1367 NonsenseMutation COSMIC:COSV57323776 colorectal cancer DOID:9256 R-HSA-4791256-APC truncation mutants [cytosol]-CandidateSet-hasCandidate R-HSA-4791278 APC truncation mutants have impaired AXIN binding loss_of_function pubmed:10749138|pubmed:8757136|pubmed:11522655|pubmed:9065402|pubmed:22438566|pubmed:10921899|pubmed:23256519|pubmed:9601641|pubmed:8638126|pubmed:9556553|pubmed:8259519|pubmed:8259518 R-HSA-5467337 APC truncation mutants have impaired AXIN binding R-HSA-195251 Assembly of the destruction complex Degradation of beta-catenin by the destruction complex R-HSA-195253 1904885 beta-catenin destruction complex assembly nonsense NA

PM1-PM4 APC APC E1353* [cytosol] R-HSA-9666990 APC UniProt:P25054 Nonsense mutation at L-glutamic acid 1353 NonsenseMutation COSMIC:COSV57321750 colorectal cancer DOID:9256 R-HSA-4791256-APC truncation mutants [cytosol]-CandidateSet-hasCandidate R-HSA-4791278 APC truncation mutants have impaired AXIN binding loss_of_function pubmed:10749138|pubmed:8757136|pubmed:11522655|pubmed:9065402|pubmed:22438566|pubmed:10921899|pubmed:23256519|pubmed:9601641|pubmed:8638126|pubmed:9556553|pubmed:8259519|pubmed:8259518 R-HSA-5467337 APC truncation mutants have impaired AXIN binding R-HSA-195251 Assembly of the destruction complex Degradation of beta-catenin by the destruction complex R-HSA-195253 1904885 beta-catenin destruction complex assembly nonsense NA

PM1-PM4 APC APC R332* [cytosol] R-HSA-9666991 APC UniProt:P25054 Nonsense mutation at L-arginine 332 NonsenseMutation COSMIC:COSV57320525 colorectal cancer DOID:9256 R-HSA-4791256-APC truncation mutants [cytosol]-CandidateSet-hasCandidate R-HSA-4791278 APC truncation mutants have impaired AXIN binding loss_of_function pubmed:10749138|pubmed:8757136|pubmed:11522655|pubmed:9065402|pubmed:22438566|pubmed:10921899|pubmed:23256519|pubmed:9601641|pubmed:8638126|pubmed:9556553|pubmed:8259519|pubmed:8259518 R-HSA-5467337 APC truncation mutants have impaired AXIN binding R-HSA-195251 Assembly of the destruction complex Degradation of beta-catenin by the destruction complex R-HSA-195253 1904885 beta-catenin destruction complex assembly nonsense NA

PM1-PM4 APC APC E1544* [cytosol] R-HSA-9666963 APC UniProt:P25054 Nonsense mutation at L-glutamic acid 1544 NonsenseMutation COSMIC:COSV57327947 colorectal cancer DOID:9256 R-HSA-4791256-APC truncation mutants [cytosol]-CandidateSet-hasCandidate R-HSA-4791278 APC truncation mutants have impaired AXIN binding loss_of_function pubmed:10749138|pubmed:8757136|pubmed:11522655|pubmed:9065402|pubmed:22438566|pubmed:10921899|pubmed:23256519|pubmed:9601641|pubmed:8638126|pubmed:9556553|pubmed:8259519|pubmed:8259518 R-HSA-5467337 APC truncation mutants have impaired AXIN binding R-HSA-195251 Assembly of the destruction complex Degradation of beta-catenin by the destruction complex R-HSA-195253 1904885 beta-catenin destruction complex assembly nonsense NA

PM1-PM4 APC APC Q1406* [cytosol] R-HSA-9666962 APC UniProt:P25054 Nonsense mutation at L-asparagine 1406 NonsenseMutation COSMIC:COSV57321535 colorectal cancer DOID:9256 R-HSA-4791256-APC truncation mutants [cytosol]-CandidateSet-hasCandidate R-HSA-4791278 APC truncation mutants have impaired AXIN binding loss_of_function pubmed:10749138|pubmed:8757136|pubmed:11522655|pubmed:9065402|pubmed:22438566|pubmed:10921899|pubmed:23256519|pubmed:9601641|pubmed:8638126|pubmed:9556553|pubmed:8259519|pubmed:8259518 R-HSA-5467337 APC truncation mutants have impaired AXIN binding R-HSA-195251 Assembly of the destruction complex Degradation of beta-catenin by the destruction complex R-HSA-195253 1904885 beta-catenin destruction complex assembly nonsense NA

PM1-PM4 APC APC E1397* [cytosol] R-HSA-9666961 APC UniProt:P25054 Nonsense mutation at L-glutamic acid 1397 NonsenseMutation COSMIC:COSV57321785 colorectal cancer DOID:9256 R-HSA-4791256-APC truncation mutants [cytosol]-CandidateSet-hasCandidate R-HSA-4791278 APC truncation mutants have impaired AXIN binding loss_of_function pubmed:10749138|pubmed:8757136|pubmed:11522655|pubmed:9065402|pubmed:22438566|pubmed:10921899|pubmed:23256519|pubmed:9601641|pubmed:8638126|pubmed:9556553|pubmed:8259519|pubmed:8259518 R-HSA-5467337 APC truncation mutants have impaired AXIN binding R-HSA-195251 Assembly of the destruction complex Degradation of beta-catenin by the destruction complex R-HSA-195253 1904885 beta-catenin destruction complex assembly nonsense NA

PM1-PM4 APC APC E1451* [cytosol] R-HSA-9666960 APC UniProt:P25054 Nonsense mutation at L-glutamic acid 1451 NonsenseMutation COSMIC:COSV57351757 colorectal cancer DOID:9256 R-HSA-4791256-APC truncation mutants [cytosol]-CandidateSet-hasCandidate R-HSA-4791278 APC truncation mutants have impaired AXIN binding loss_of_function pubmed:10749138|pubmed:8757136|pubmed:11522655|pubmed:9065402|pubmed:22438566|pubmed:10921899|pubmed:23256519|pubmed:9601641|pubmed:8638126|pubmed:9556553|pubmed:8259519|pubmed:8259518 R-HSA-5467337 APC truncation mutants have impaired AXIN binding R-HSA-195251 Assembly of the destruction complex Degradation of beta-catenin by the destruction complex R-HSA-195253 1904885 beta-catenin destruction complex assembly nonsense NA

PM1-PM4-PS1 APC APC S1539* [cytosol] R-HSA-9666967 APC UniProt:P25054 Nonsense mutation at L-serine 1539 NonsenseMutation COSMIC:COSV57338819|COSMIC:COSV57338685 colorectal cancer DOID:9256 R-HSA-4791256-APC truncation mutants [cytosol]-CandidateSet-hasCandidate R-HSA-4791278 APC truncation mutants have impaired AXIN binding loss_of_function pubmed:10749138|pubmed:8757136|pubmed:11522655|pubmed:9065402|pubmed:22438566|pubmed:10921899|pubmed:23256519|pubmed:9601641|pubmed:8638126|pubmed:9556553|pubmed:8259519|pubmed:8259518 R-HSA-5467337 APC truncation mutants have impaired AXIN binding R-HSA-195251 Assembly of the destruction complex Degradation of beta-catenin by the destruction complex R-HSA-195253 1904885 beta-catenin destruction complex assembly nonsense NA

PM1-PM4 APC APC S1392* [cytosol] R-HSA-9666966 APC UniProt:P25054 Nonsense mutation at L-serine 1392 NonsenseMutation COSMIC:COSV57326586 colorectal cancer DOID:9256 R-HSA-4791256-APC truncation mutants [cytosol]-CandidateSet-hasCandidate R-HSA-4791278 APC truncation mutants have impaired AXIN binding loss_of_function pubmed:10749138|pubmed:8757136|pubmed:11522655|pubmed:9065402|pubmed:22438566|pubmed:10921899|pubmed:23256519|pubmed:9601641|pubmed:8638126|pubmed:9556553|pubmed:8259519|pubmed:8259518 R-HSA-5467337 APC truncation mutants have impaired AXIN binding R-HSA-195251 Assembly of the destruction complex Degradation of beta-catenin by the destruction complex R-HSA-195253 1904885 beta-catenin destruction complex assembly nonsense NA

PM1-PM4 APC APC Q886* [cytosol] R-HSA-9666965 APC UniProt:P25054 Nonsense mutation at L-asparagine 886 NonsenseMutation COSMIC:COSV57332678 colorectal cancer DOID:9256 R-HSA-4791256-APC truncation mutants [cytosol]-CandidateSet-hasCandidate R-HSA-4791278 APC truncation mutants have impaired AXIN binding loss_of_function pubmed:10749138|pubmed:8757136|pubmed:11522655|pubmed:9065402|pubmed:22438566|pubmed:10921899|pubmed:23256519|pubmed:9601641|pubmed:8638126|pubmed:9556553|pubmed:8259519|pubmed:8259518 R-HSA-5467337 APC truncation mutants have impaired AXIN binding R-HSA-195251 Assembly of the destruction complex Degradation of beta-catenin by the destruction complex R-HSA-195253 1904885 beta-catenin destruction complex assembly nonsense NA

PM1-PM4-PS1 APC APC Y1376* [cytosol] R-HSA-9666964 APC UniProt:P25054 Nonsense mutation at L-tyrosine 1376 NonsenseMutation COSMIC:COSV57326134|COSMIC:COSV57378080 colorectal cancer DOID:9256 R-HSA-4791256-APC truncation mutants [cytosol]-CandidateSet-hasCandidate R-HSA-4791278 APC truncation mutants have impaired AXIN binding loss_of_function pubmed:10749138|pubmed:8757136|pubmed:11522655|pubmed:9065402|pubmed:22438566|pubmed:10921899|pubmed:23256519|pubmed:9601641|pubmed:8638126|pubmed:9556553|pubmed:8259519|pubmed:8259518 R-HSA-5467337 APC truncation mutants have impaired AXIN binding R-HSA-195251 Assembly of the destruction complex Degradation of beta-catenin by the destruction complex R-HSA-195253 1904885 beta-catenin destruction complex assembly nonsense NA

PM1-PM4 APC APC G1412* [cytosol] R-HSA-9666971 APC UniProt:P25054 Nonsense mutation at glycine 1412 NonsenseMutation COSMIC:COSV57341688 colorectal cancer DOID:9256 R-HSA-4791256-APC truncation mutants [cytosol]-CandidateSet-hasCandidate R-HSA-4791278 APC truncation mutants have impaired AXIN binding loss_of_function pubmed:10749138|pubmed:8757136|pubmed:11522655|pubmed:9065402|pubmed:22438566|pubmed:10921899|pubmed:23256519|pubmed:9601641|pubmed:8638126|pubmed:9556553|pubmed:8259519|pubmed:8259518 R-HSA-5467337 APC truncation mutants have impaired AXIN binding R-HSA-195251 Assembly of the destruction complex Degradation of beta-catenin by the destruction complex R-HSA-195253 1904885 beta-catenin destruction complex assembly nonsense NA

PM1-PM4-PS1 APC APC S1315* [cytosol] R-HSA-9666970 APC UniProt:P25054 Nonsense mutation at L-serine 1315 NonsenseMutation COSMIC:COSV57326747|COSMIC:COSV57328428 colorectal cancer DOID:9256 R-HSA-4791256-APC truncation mutants [cytosol]-CandidateSet-hasCandidate R-HSA-4791278 APC truncation mutants have impaired AXIN binding loss_of_function pubmed:10749138|pubmed:8757136|pubmed:11522655|pubmed:9065402|pubmed:22438566|pubmed:10921899|pubmed:23256519|pubmed:9601641|pubmed:8638126|pubmed:9556553|pubmed:8259519|pubmed:8259518 R-HSA-5467337 APC truncation mutants have impaired AXIN binding R-HSA-195251 Assembly of the destruction complex Degradation of beta-catenin by the destruction complex R-HSA-195253 1904885 beta-catenin destruction complex assembly nonsense NA

PM1-PM4-PS1 APC APC S895* [cytosol] R-HSA-9666969 APC UniProt:P25054 Nonsense mutation at L-serine 895 NonsenseMutation COSMIC:COSV57340513|COSMIC:COSV57327400 colorectal cancer DOID:9256 R-HSA-4791256-APC truncation mutants [cytosol]-CandidateSet-hasCandidate R-HSA-4791278 APC truncation mutants have impaired AXIN binding loss_of_function pubmed:10749138|pubmed:8757136|pubmed:11522655|pubmed:9065402|pubmed:22438566|pubmed:10921899|pubmed:23256519|pubmed:9601641|pubmed:8638126|pubmed:9556553|pubmed:8259519|pubmed:8259518 R-HSA-5467337 APC truncation mutants have impaired AXIN binding R-HSA-195251 Assembly of the destruction complex Degradation of beta-catenin by the destruction complex R-HSA-195253 1904885 beta-catenin destruction complex assembly nonsense NA

PM1-PM4 APC APC E1306* [cytosol] R-HSA-9666975 APC UniProt:P25054 Nonsense mutation at L-glutamic acid 1306 NonsenseMutation COSMIC:COSV57322511 colorectal cancer DOID:9256 R-HSA-4791256-APC truncation mutants [cytosol]-CandidateSet-hasCandidate R-HSA-4791278 APC truncation mutants have impaired AXIN binding loss_of_function pubmed:10749138|pubmed:8757136|pubmed:11522655|pubmed:9065402|pubmed:22438566|pubmed:10921899|pubmed:23256519|pubmed:9601641|pubmed:8638126|pubmed:9556553|pubmed:8259519|pubmed:8259518 R-HSA-5467337 APC truncation mutants have impaired AXIN binding R-HSA-195251 Assembly of the destruction complex Degradation of beta-catenin by the destruction complex R-HSA-195253 1904885 beta-catenin destruction complex assembly nonsense NA

PM1-PM4-PS1 APC APC S943* [cytosol] R-HSA-9666973 APC UniProt:P25054 Nonsense mutation at L-serine 943 NonsenseMutation COSMIC:COSV57334177|COSMIC:COSV57340756 colorectal cancer DOID:9256 R-HSA-4791256-APC truncation mutants [cytosol]-CandidateSet-hasCandidate R-HSA-4791278 APC truncation mutants have impaired AXIN binding loss_of_function pubmed:10749138|pubmed:8757136|pubmed:11522655|pubmed:9065402|pubmed:22438566|pubmed:10921899|pubmed:23256519|pubmed:9601641|pubmed:8638126|pubmed:9556553|pubmed:8259519|pubmed:8259518 R-HSA-5467337 APC truncation mutants have impaired AXIN binding R-HSA-195251 Assembly of the destruction complex Degradation of beta-catenin by the destruction complex R-HSA-195253 1904885 beta-catenin destruction complex assembly nonsense NA

PM1-PM4 APC APC Q1303* [cytosol] R-HSA-9666972 APC UniProt:P25054 Nonsense mutation at L-asparagine 1303 NonsenseMutation COSMIC:COSV57323819 colorectal cancer DOID:9256 R-HSA-4791256-APC truncation mutants [cytosol]-CandidateSet-hasCandidate R-HSA-4791278 APC truncation mutants have impaired AXIN binding loss_of_function pubmed:10749138|pubmed:8757136|pubmed:11522655|pubmed:9065402|pubmed:22438566|pubmed:10921899|pubmed:23256519|pubmed:9601641|pubmed:8638126|pubmed:9556553|pubmed:8259519|pubmed:8259518 R-HSA-5467337 APC truncation mutants have impaired AXIN binding R-HSA-195251 Assembly of the destruction complex Degradation of beta-catenin by the destruction complex R-HSA-195253 1904885 beta-catenin destruction complex assembly nonsense NA

PM1-PM4 APC APC E941* [cytosol] R-HSA-9666947 APC UniProt:P25054 Nonsense mutation at L-glutamic acid 941 NonsenseMutation COSMIC:COSV57327519 colorectal cancer DOID:9256 R-HSA-4791256-APC truncation mutants [cytosol]-CandidateSet-hasCandidate R-HSA-4791278 APC truncation mutants have impaired AXIN binding loss_of_function pubmed:10749138|pubmed:8757136|pubmed:11522655|pubmed:9065402|pubmed:22438566|pubmed:10921899|pubmed:23256519|pubmed:9601641|pubmed:8638126|pubmed:9556553|pubmed:8259519|pubmed:8259518 R-HSA-5467337 APC truncation mutants have impaired AXIN binding R-HSA-195251 Assembly of the destruction complex Degradation of beta-catenin by the destruction complex R-HSA-195253 1904885 beta-catenin destruction complex assembly nonsense NA

PM1-PM4 APC APC Q767* [cytosol] R-HSA-9666946 APC UniProt:P25054 Nonsense mutation at L-asparagine 767 NonsenseMutation COSMIC:COSV57328302 colorectal cancer DOID:9256 R-HSA-4791256-APC truncation mutants [cytosol]-CandidateSet-hasCandidate R-HSA-4791278 APC truncation mutants have impaired AXIN binding loss_of_function pubmed:10749138|pubmed:8757136|pubmed:11522655|pubmed:9065402|pubmed:22438566|pubmed:10921899|pubmed:23256519|pubmed:9601641|pubmed:8638126|pubmed:9556553|pubmed:8259519|pubmed:8259518 R-HSA-5467337 APC truncation mutants have impaired AXIN binding R-HSA-195251 Assembly of the destruction complex Degradation of beta-catenin by the destruction complex R-HSA-195253 1904885 beta-catenin destruction complex assembly nonsense NA

PM1-PM4 APC APC E1374* [cytosol] R-HSA-9666945 APC UniProt:P25054 Nonsense mutation at L-glutamic acid 1374 NonsenseMutation COSMIC:COSV57322620 colorectal cancer DOID:9256 R-HSA-4791256-APC truncation mutants [cytosol]-CandidateSet-hasCandidate R-HSA-4791278 APC truncation mutants have impaired AXIN binding loss_of_function pubmed:10749138|pubmed:8757136|pubmed:11522655|pubmed:9065402|pubmed:22438566|pubmed:10921899|pubmed:23256519|pubmed:9601641|pubmed:8638126|pubmed:9556553|pubmed:8259519|pubmed:8259518 R-HSA-5467337 APC truncation mutants have impaired AXIN binding R-HSA-195251 Assembly of the destruction complex Degradation of beta-catenin by the destruction complex R-HSA-195253 1904885 beta-catenin destruction complex assembly nonsense NA

PM1-PM4 APC APC E1284* [cytosol] R-HSA-9666944 APC UniProt:P25054 Nonsense mutation at L-glutamic acid 1284 NonsenseMutation COSMIC:COSV57324438 colorectal cancer DOID:9256 R-HSA-4791256-APC truncation mutants [cytosol]-CandidateSet-hasCandidate R-HSA-4791278 APC truncation mutants have impaired AXIN binding loss_of_function pubmed:10749138|pubmed:8757136|pubmed:11522655|pubmed:9065402|pubmed:22438566|pubmed:10921899|pubmed:23256519|pubmed:9601641|pubmed:8638126|pubmed:9556553|pubmed:8259519|pubmed:8259518 R-HSA-5467337 APC truncation mutants have impaired AXIN binding R-HSA-195251 Assembly of the destruction complex Degradation of beta-catenin by the destruction complex R-HSA-195253 1904885 beta-catenin destruction complex assembly nonsense NA

PM1-PM4 APC APC E1408* [cytosol] R-HSA-9666951 APC UniProt:P25054 Nonsense mutation at L-glutamic acid 1408 NonsenseMutation COSMIC:COSV57326063 colorectal cancer DOID:9256 R-HSA-4791256-APC truncation mutants [cytosol]-CandidateSet-hasCandidate R-HSA-4791278 APC truncation mutants have impaired AXIN binding loss_of_function pubmed:10749138|pubmed:8757136|pubmed:11522655|pubmed:9065402|pubmed:22438566|pubmed:10921899|pubmed:23256519|pubmed:9601641|pubmed:8638126|pubmed:9556553|pubmed:8259519|pubmed:8259518 R-HSA-5467337 APC truncation mutants have impaired AXIN binding R-HSA-195251 Assembly of the destruction complex Degradation of beta-catenin by the destruction complex R-HSA-195253 1904885 beta-catenin destruction complex assembly nonsense NA

PM1-PM4 APC APC K1363* [cytosol] R-HSA-9666950 APC UniProt:P25054 Nonsense mutation at L-lysine 1363 NonsenseMutation COSMIC:COSV57337694 colorectal cancer DOID:9256 R-HSA-4791256-APC truncation mutants [cytosol]-CandidateSet-hasCandidate R-HSA-4791278 APC truncation mutants have impaired AXIN binding loss_of_function pubmed:10749138|pubmed:8757136|pubmed:11522655|pubmed:9065402|pubmed:22438566|pubmed:10921899|pubmed:23256519|pubmed:9601641|pubmed:8638126|pubmed:9556553|pubmed:8259519|pubmed:8259518 R-HSA-5467337 APC truncation mutants have impaired AXIN binding R-HSA-195251 Assembly of the destruction complex Degradation of beta-catenin by the destruction complex R-HSA-195253 1904885 beta-catenin destruction complex assembly nonsense NA

PM1-PM4 APC APC Q1294* [cytosol] R-HSA-9666949 APC UniProt:P25054 Nonsense mutation at L-asparagine 1294 NonsenseMutation COSMIC:COSV57323524 colorectal cancer DOID:9256 R-HSA-4791256-APC truncation mutants [cytosol]-CandidateSet-hasCandidate R-HSA-4791278 APC truncation mutants have impaired AXIN binding loss_of_function pubmed:10749138|pubmed:8757136|pubmed:11522655|pubmed:9065402|pubmed:22438566|pubmed:10921899|pubmed:23256519|pubmed:9601641|pubmed:8638126|pubmed:9556553|pubmed:8259519|pubmed:8259518 R-HSA-5467337 APC truncation mutants have impaired AXIN binding R-HSA-195251 Assembly of the destruction complex Degradation of beta-catenin by the destruction complex R-HSA-195253 1904885 beta-catenin destruction complex assembly nonsense NA

PM1-PM4-PS1 APC APC L1564* [cytosol] R-HSA-9666948 APC UniProt:P25054 Nonsense mutation at L-leucine 1564 NonsenseMutation COSMIC:COSV57390166|COSMIC:COSV57346018 colorectal cancer DOID:9256 R-HSA-4791256-APC truncation mutants [cytosol]-CandidateSet-hasCandidate R-HSA-4791278 APC truncation mutants have impaired AXIN binding loss_of_function pubmed:10749138|pubmed:8757136|pubmed:11522655|pubmed:9065402|pubmed:22438566|pubmed:10921899|pubmed:23256519|pubmed:9601641|pubmed:8638126|pubmed:9556553|pubmed:8259519|pubmed:8259518 R-HSA-5467337 APC truncation mutants have impaired AXIN binding R-HSA-195251 Assembly of the destruction complex Degradation of beta-catenin by the destruction complex R-HSA-195253 1904885 beta-catenin destruction complex assembly nonsense NA

PM1-PM4 APC APC Q1429* [cytosol] R-HSA-9666955 APC UniProt:P25054 Nonsense mutation at L-glutamine 1429 NonsenseMutation COSMIC:COSV57322632 colorectal cancer DOID:9256 R-HSA-4791256-APC truncation mutants [cytosol]-CandidateSet-hasCandidate R-HSA-4791278 APC truncation mutants have impaired AXIN binding loss_of_function pubmed:10749138|pubmed:8757136|pubmed:11522655|pubmed:9065402|pubmed:22438566|pubmed:10921899|pubmed:23256519|pubmed:9601641|pubmed:8638126|pubmed:9556553|pubmed:8259519|pubmed:8259518 R-HSA-5467337 APC truncation mutants have impaired AXIN binding R-HSA-195251 Assembly of the destruction complex Degradation of beta-catenin by the destruction complex R-HSA-195253 1904885 beta-catenin destruction complex assembly nonsense NA

PM1-PM4-PS1 APC APC W1049* [cytosol] R-HSA-9666954 APC UniProt:P25054 Nonsense mutation at L-tryptophan 1049 NonsenseMutation COSMIC:COSV57350496|COSMIC:COSV57322894 colorectal cancer DOID:9256 R-HSA-4791256-APC truncation mutants [cytosol]-CandidateSet-hasCandidate R-HSA-4791278 APC truncation mutants have impaired AXIN binding loss_of_function pubmed:10749138|pubmed:8757136|pubmed:11522655|pubmed:9065402|pubmed:22438566|pubmed:10921899|pubmed:23256519|pubmed:9601641|pubmed:8638126|pubmed:9556553|pubmed:8259519|pubmed:8259518 R-HSA-5467337 APC truncation mutants have impaired AXIN binding R-HSA-195251 Assembly of the destruction complex Degradation of beta-catenin by the destruction complex R-HSA-195253 1904885 beta-catenin destruction complex assembly nonsense NA

PM1-PM4 APC APC G1309Dfs*4 [cytosol] R-HSA-4791251 APC UniProt:P25054 Replacement of residues 1309 to 1311 by DWN FragmentReplacedModification COSMIC:COSV57321812 colorectal cancer DOID:9256 pubmed:8111410 R-HSA-4791256-APC truncation mutants [cytosol]-CandidateSet-hasCandidate R-HSA-4791278 APC truncation mutants have impaired AXIN binding loss_of_function pubmed:10749138|pubmed:8757136|pubmed:11522655|pubmed:9065402|pubmed:22438566|pubmed:10921899|pubmed:23256519|pubmed:9601641|pubmed:8638126|pubmed:9556553|pubmed:8259519|pubmed:8259518 R-HSA-5467337 APC truncation mutants have impaired AXIN binding R-HSA-195251 Assembly of the destruction complex Degradation of beta-catenin by the destruction complex R-HSA-195253 1904885 beta-catenin destruction complex assembly frameshift NA

PM1-PM4 APC APC E1317* [cytosol] R-HSA-9666953 APC UniProt:P25054 Nonsense mutation at L-glutamic acid 1317 NonsenseMutation COSMIC:COSV57325114 colorectal cancer DOID:9256 R-HSA-4791256-APC truncation mutants [cytosol]-CandidateSet-hasCandidate R-HSA-4791278 APC truncation mutants have impaired AXIN binding loss_of_function pubmed:10749138|pubmed:8757136|pubmed:11522655|pubmed:9065402|pubmed:22438566|pubmed:10921899|pubmed:23256519|pubmed:9601641|pubmed:8638126|pubmed:9556553|pubmed:8259519|pubmed:8259518 R-HSA-5467337 APC truncation mutants have impaired AXIN binding R-HSA-195251 Assembly of the destruction complex Degradation of beta-catenin by the destruction complex R-HSA-195253 1904885 beta-catenin destruction complex assembly nonsense NA

PM1-PM4 APC APC E1464* [cytosol] R-HSA-9666952 APC UniProt:P25054 Nonsense mutation at L-glutamic acid 1464 NonsenseMutation COSMIC:COSV57338936 colorectal cancer DOID:9256 R-HSA-4791256-APC truncation mutants [cytosol]-CandidateSet-hasCandidate R-HSA-4791278 APC truncation mutants have impaired AXIN binding loss_of_function pubmed:10749138|pubmed:8757136|pubmed:11522655|pubmed:9065402|pubmed:22438566|pubmed:10921899|pubmed:23256519|pubmed:9601641|pubmed:8638126|pubmed:9556553|pubmed:8259519|pubmed:8259518 R-HSA-5467337 APC truncation mutants have impaired AXIN binding R-HSA-195251 Assembly of the destruction complex Degradation of beta-catenin by the destruction complex R-HSA-195253 1904885 beta-catenin destruction complex assembly nonsense NA

PM1-PM4 APC APC Q1127* [cytosol] R-HSA-9666959 APC UniProt:P25054 Nonsense mutation at L-asparagine 1127 NonsenseMutation COSMIC:COSV57332099 colorectal cancer DOID:9256 R-HSA-4791256-APC truncation mutants [cytosol]-CandidateSet-hasCandidate R-HSA-4791278 APC truncation mutants have impaired AXIN binding loss_of_function pubmed:10749138|pubmed:8757136|pubmed:11522655|pubmed:9065402|pubmed:22438566|pubmed:10921899|pubmed:23256519|pubmed:9601641|pubmed:8638126|pubmed:9556553|pubmed:8259519|pubmed:8259518 R-HSA-5467337 APC truncation mutants have impaired AXIN binding R-HSA-195251 Assembly of the destruction complex Degradation of beta-catenin by the destruction complex R-HSA-195253 1904885 beta-catenin destruction complex assembly nonsense NA

PM1-PM4 APC APC E1577* [cytosol] R-HSA-9666958 APC UniProt:P25054 Nonsense mutation at L-glutamic acid 1577 NonsenseMutation COSMIC:COSV57322139 colorectal cancer DOID:9256 R-HSA-4791256-APC truncation mutants [cytosol]-CandidateSet-hasCandidate R-HSA-4791278 APC truncation mutants have impaired AXIN binding loss_of_function pubmed:10749138|pubmed:8757136|pubmed:11522655|pubmed:9065402|pubmed:22438566|pubmed:10921899|pubmed:23256519|pubmed:9601641|pubmed:8638126|pubmed:9556553|pubmed:8259519|pubmed:8259518 R-HSA-5467337 APC truncation mutants have impaired AXIN binding R-HSA-195251 Assembly of the destruction complex Degradation of beta-catenin by the destruction complex R-HSA-195253 1904885 beta-catenin destruction complex assembly nonsense NA

PM1-PM4-PS1 APC APC S1344* [cytosol] R-HSA-9666957 APC UniProt:P25054 Nonsense mutation at L-serine 1344 NonsenseMutation COSMIC:COSV57323895|COSMIC:COSV57323283 colorectal cancer DOID:9256 R-HSA-4791256-APC truncation mutants [cytosol]-CandidateSet-hasCandidate R-HSA-4791278 APC truncation mutants have impaired AXIN binding loss_of_function pubmed:10749138|pubmed:8757136|pubmed:11522655|pubmed:9065402|pubmed:22438566|pubmed:10921899|pubmed:23256519|pubmed:9601641|pubmed:8638126|pubmed:9556553|pubmed:8259519|pubmed:8259518 R-HSA-5467337 APC truncation mutants have impaired AXIN binding R-HSA-195251 Assembly of the destruction complex Degradation of beta-catenin by the destruction complex R-HSA-195253 1904885 beta-catenin destruction complex assembly nonsense NA

PM1-PM4 APC APC K1308* [cytosol] R-HSA-9666956 APC UniProt:P25054 Nonsense mutation at L-lysine 1308 NonsenseMutation COSMIC:COSV57320906 colorectal cancer DOID:9256 R-HSA-4791256-APC truncation mutants [cytosol]-CandidateSet-hasCandidate R-HSA-4791278 APC truncation mutants have impaired AXIN binding loss_of_function pubmed:10749138|pubmed:8757136|pubmed:11522655|pubmed:9065402|pubmed:22438566|pubmed:10921899|pubmed:23256519|pubmed:9601641|pubmed:8638126|pubmed:9556553|pubmed:8259519|pubmed:8259518 R-HSA-5467337 APC truncation mutants have impaired AXIN binding R-HSA-195251 Assembly of the destruction complex Degradation of beta-catenin by the destruction complex R-HSA-195253 1904885 beta-catenin destruction complex assembly nonsense NA

PM1-PM4 APC APC E1345* [cytosol] R-HSA-9666995 APC UniProt:P25054 Nonsense mutation at L-glutamic acid 1345 NonsenseMutation COSMIC:COSV57322919 colorectal cancer DOID:9256 R-HSA-4791256-APC truncation mutants [cytosol]-CandidateSet-hasCandidate R-HSA-4791278 APC truncation mutants have impaired AXIN binding loss_of_function pubmed:10749138|pubmed:8757136|pubmed:11522655|pubmed:9065402|pubmed:22438566|pubmed:10921899|pubmed:23256519|pubmed:9601641|pubmed:8638126|pubmed:9556553|pubmed:8259519|pubmed:8259518 R-HSA-5467337 APC truncation mutants have impaired AXIN binding R-HSA-195251 Assembly of the destruction complex Degradation of beta-catenin by the destruction complex R-HSA-195253 1904885 beta-catenin destruction complex assembly nonsense NA

PM1-PM4 APC APC E1322* [cytosol] R-HSA-9666994 APC UniProt:P25054 Nonsense mutation at L-glutamic acid 1322 NonsenseMutation COSMIC:COSV57325414 colorectal cancer DOID:9256 R-HSA-4791256-APC truncation mutants [cytosol]-CandidateSet-hasCandidate R-HSA-4791278 APC truncation mutants have impaired AXIN binding loss_of_function pubmed:10749138|pubmed:8757136|pubmed:11522655|pubmed:9065402|pubmed:22438566|pubmed:10921899|pubmed:23256519|pubmed:9601641|pubmed:8638126|pubmed:9556553|pubmed:8259519|pubmed:8259518 R-HSA-5467337 APC truncation mutants have impaired AXIN binding R-HSA-195251 Assembly of the destruction complex Degradation of beta-catenin by the destruction complex R-HSA-195253 1904885 beta-catenin destruction complex assembly nonsense NA

PM1-PM4 APC APC E1151* [cytosol] R-HSA-9666993 APC UniProt:P25054 Nonsense mutation at L-glutamic acid 1151 NonsenseMutation COSMIC:COSV57324251 colorectal cancer DOID:9256 R-HSA-4791256-APC truncation mutants [cytosol]-CandidateSet-hasCandidate R-HSA-4791278 APC truncation mutants have impaired AXIN binding loss_of_function pubmed:10749138|pubmed:8757136|pubmed:11522655|pubmed:9065402|pubmed:22438566|pubmed:10921899|pubmed:23256519|pubmed:9601641|pubmed:8638126|pubmed:9556553|pubmed:8259519|pubmed:8259518 R-HSA-5467337 APC truncation mutants have impaired AXIN binding R-HSA-195251 Assembly of the destruction complex Degradation of beta-catenin by the destruction complex R-HSA-195253 1904885 beta-catenin destruction complex assembly nonsense NA

PM1-PM4 APC APC E1295* [cytosol] R-HSA-9666992 APC UniProt:P25054 Nonsense mutation at L-glutamic acid 1295 NonsenseMutation COSMIC:COSV57323670 colorectal cancer DOID:9256 R-HSA-4791256-APC truncation mutants [cytosol]-CandidateSet-hasCandidate R-HSA-4791278 APC truncation mutants have impaired AXIN binding loss_of_function pubmed:10749138|pubmed:8757136|pubmed:11522655|pubmed:9065402|pubmed:22438566|pubmed:10921899|pubmed:23256519|pubmed:9601641|pubmed:8638126|pubmed:9556553|pubmed:8259519|pubmed:8259518 R-HSA-5467337 APC truncation mutants have impaired AXIN binding R-HSA-195251 Assembly of the destruction complex Degradation of beta-catenin by the destruction complex R-HSA-195253 1904885 beta-catenin destruction complex assembly nonsense NA

PM1-PM4 APC APC Q1328* [cytosol] R-HSA-9666998 APC UniProt:P25054 Nonsense mutation at L-asparagine 1328 NonsenseMutation COSMIC:COSV57324976 colorectal cancer DOID:9256 R-HSA-4791256-APC truncation mutants [cytosol]-CandidateSet-hasCandidate R-HSA-4791278 APC truncation mutants have impaired AXIN binding loss_of_function pubmed:10749138|pubmed:8757136|pubmed:11522655|pubmed:9065402|pubmed:22438566|pubmed:10921899|pubmed:23256519|pubmed:9601641|pubmed:8638126|pubmed:9556553|pubmed:8259519|pubmed:8259518 R-HSA-5467337 APC truncation mutants have impaired AXIN binding R-HSA-195251 Assembly of the destruction complex Degradation of beta-catenin by the destruction complex R-HSA-195253 1904885 beta-catenin destruction complex assembly nonsense NA

PM1-PM4-PS1 APC APC S1282* [cytosol] R-HSA-9666997 APC UniProt:P25054 Nonsense mutation at L-serine 1282 NonsenseMutation COSMIC:COSV57344541|COSMIC:COSV57326418 colorectal cancer DOID:9256 R-HSA-4791256-APC truncation mutants [cytosol]-CandidateSet-hasCandidate R-HSA-4791278 APC truncation mutants have impaired AXIN binding loss_of_function pubmed:10749138|pubmed:8757136|pubmed:11522655|pubmed:9065402|pubmed:22438566|pubmed:10921899|pubmed:23256519|pubmed:9601641|pubmed:8638126|pubmed:9556553|pubmed:8259519|pubmed:8259518 R-HSA-5467337 APC truncation mutants have impaired AXIN binding R-HSA-195251 Assembly of the destruction complex Degradation of beta-catenin by the destruction complex R-HSA-195253 1904885 beta-catenin destruction complex assembly nonsense NA

PM1-PM4 APC APC R1114* [cytosol] R-HSA-9666996 APC UniProt:P25054 Nonsense mutation at L-arginine 1114 NonsenseMutation COSMIC:COSV57321465 colorectal cancer DOID:9256 R-HSA-4791256-APC truncation mutants [cytosol]-CandidateSet-hasCandidate R-HSA-4791278 APC truncation mutants have impaired AXIN binding loss_of_function pubmed:10749138|pubmed:8757136|pubmed:11522655|pubmed:9065402|pubmed:22438566|pubmed:10921899|pubmed:23256519|pubmed:9601641|pubmed:8638126|pubmed:9556553|pubmed:8259519|pubmed:8259518 R-HSA-5467337 APC truncation mutants have impaired AXIN binding R-HSA-195251 Assembly of the destruction complex Degradation of beta-catenin by the destruction complex R-HSA-195253 1904885 beta-catenin destruction complex assembly nonsense NA

PM1-PM4 APC APC E984* [cytosol] R-HSA-9666979 APC UniProt:P25054 Nonsense mutation at L-glutamic acid 984 NonsenseMutation COSMIC:COSV57331724 colorectal cancer DOID:9256 R-HSA-4791256-APC truncation mutants [cytosol]-CandidateSet-hasCandidate R-HSA-4791278 APC truncation mutants have impaired AXIN binding loss_of_function pubmed:10749138|pubmed:8757136|pubmed:11522655|pubmed:9065402|pubmed:22438566|pubmed:10921899|pubmed:23256519|pubmed:9601641|pubmed:8638126|pubmed:9556553|pubmed:8259519|pubmed:8259518 R-HSA-5467337 APC truncation mutants have impaired AXIN binding R-HSA-195251 Assembly of the destruction complex Degradation of beta-catenin by the destruction complex R-HSA-195253 1904885 beta-catenin destruction complex assembly nonsense NA

PM1-PM4 APC APC E1379* [cytosol] R-HSA-9666978 APC UniProt:P25054 Nonsense mutation at L-glutamic acid 1379 NonsenseMutation COSMIC:COSV57320699 colorectal cancer DOID:9256 R-HSA-4791256-APC truncation mutants [cytosol]-CandidateSet-hasCandidate R-HSA-4791278 APC truncation mutants have impaired AXIN binding loss_of_function pubmed:10749138|pubmed:8757136|pubmed:11522655|pubmed:9065402|pubmed:22438566|pubmed:10921899|pubmed:23256519|pubmed:9601641|pubmed:8638126|pubmed:9556553|pubmed:8259519|pubmed:8259518 R-HSA-5467337 APC truncation mutants have impaired AXIN binding R-HSA-195251 Assembly of the destruction complex Degradation of beta-catenin by the destruction complex R-HSA-195253 1904885 beta-catenin destruction complex assembly nonsense NA

PM1-PM4 APC APC R232* [cytosol] R-HSA-9666977 APC UniProt:P25054 Nonsense mutation at L-arginine 232 NonsenseMutation COSMIC:COSV57320753 colorectal cancer DOID:9256 R-HSA-4791256-APC truncation mutants [cytosol]-CandidateSet-hasCandidate R-HSA-4791278 APC truncation mutants have impaired AXIN binding loss_of_function pubmed:10749138|pubmed:8757136|pubmed:11522655|pubmed:9065402|pubmed:22438566|pubmed:10921899|pubmed:23256519|pubmed:9601641|pubmed:8638126|pubmed:9556553|pubmed:8259519|pubmed:8259518 R-HSA-5467337 APC truncation mutants have impaired AXIN binding R-HSA-195251 Assembly of the destruction complex Degradation of beta-catenin by the destruction complex R-HSA-195253 1904885 beta-catenin destruction complex assembly nonsense NA

PM1-PM4-PS1 APC APC S1356* [cytosol] R-HSA-9666976 APC UniProt:P25054 Nonsense mutation at L-serine 1356 NonsenseMutation COSMIC:COSV57330189|COSMIC:COSV57325773 colorectal cancer DOID:9256 R-HSA-4791256-APC truncation mutants [cytosol]-CandidateSet-hasCandidate R-HSA-4791278 APC truncation mutants have impaired AXIN binding loss_of_function pubmed:10749138|pubmed:8757136|pubmed:11522655|pubmed:9065402|pubmed:22438566|pubmed:10921899|pubmed:23256519|pubmed:9601641|pubmed:8638126|pubmed:9556553|pubmed:8259519|pubmed:8259518 R-HSA-5467337 APC truncation mutants have impaired AXIN binding R-HSA-195251 Assembly of the destruction complex Degradation of beta-catenin by the destruction complex R-HSA-195253 1904885 beta-catenin destruction complex assembly nonsense NA

PM1-PM4 APC APC R554* [cytosol] R-HSA-9666983 APC UniProt:P25054 Nonsense mutation at L-arginine 554 NonsenseMutation COSMIC:COSV57321615 colorectal cancer DOID:9256 R-HSA-4791256-APC truncation mutants [cytosol]-CandidateSet-hasCandidate R-HSA-4791278 APC truncation mutants have impaired AXIN binding loss_of_function pubmed:10749138|pubmed:8757136|pubmed:11522655|pubmed:9065402|pubmed:22438566|pubmed:10921899|pubmed:23256519|pubmed:9601641|pubmed:8638126|pubmed:9556553|pubmed:8259519|pubmed:8259518 R-HSA-5467337 APC truncation mutants have impaired AXIN binding R-HSA-195251 Assembly of the destruction complex Degradation of beta-catenin by the destruction complex R-HSA-195253 1904885 beta-catenin destruction complex assembly nonsense NA

PM1-PM4 APC APC Q1378* [cytosol] R-HSA-9666982 APC UniProt:P25054 Nonsense mutation at L-asparagine 1378 NonsenseMutation COSMIC:COSV57322261 colorectal cancer DOID:9256 R-HSA-4791256-APC truncation mutants [cytosol]-CandidateSet-hasCandidate R-HSA-4791278 APC truncation mutants have impaired AXIN binding loss_of_function pubmed:10749138|pubmed:8757136|pubmed:11522655|pubmed:9065402|pubmed:22438566|pubmed:10921899|pubmed:23256519|pubmed:9601641|pubmed:8638126|pubmed:9556553|pubmed:8259519|pubmed:8259518 R-HSA-5467337 APC truncation mutants have impaired AXIN binding R-HSA-195251 Assembly of the destruction complex Degradation of beta-catenin by the destruction complex R-HSA-195253 1904885 beta-catenin destruction complex assembly nonsense NA

PM1-PM4 APC APC E1286* [cytosol] R-HSA-9666981 APC UniProt:P25054 Nonsense mutation at L-glutamic acid 1286 NonsenseMutation COSMIC:COSV57325166 colorectal cancer DOID:9256 R-HSA-4791256-APC truncation mutants [cytosol]-CandidateSet-hasCandidate R-HSA-4791278 APC truncation mutants have impaired AXIN binding loss_of_function pubmed:10749138|pubmed:8757136|pubmed:11522655|pubmed:9065402|pubmed:22438566|pubmed:10921899|pubmed:23256519|pubmed:9601641|pubmed:8638126|pubmed:9556553|pubmed:8259519|pubmed:8259518 R-HSA-5467337 APC truncation mutants have impaired AXIN binding R-HSA-195251 Assembly of the destruction complex Degradation of beta-catenin by the destruction complex R-HSA-195253 1904885 beta-catenin destruction complex assembly nonsense NA

PM1-PM4-PS1 APC APC Y935* [cytosol] R-HSA-9666980 APC UniProt:P25054 Nonsense mutation at L-tyrosine 935 NonsenseMutation COSMIC:COSV57328770|COSMIC:COSV57324059 colorectal cancer DOID:9256 R-HSA-4791256-APC truncation mutants [cytosol]-CandidateSet-hasCandidate R-HSA-4791278 APC truncation mutants have impaired AXIN binding loss_of_function pubmed:10749138|pubmed:8757136|pubmed:11522655|pubmed:9065402|pubmed:22438566|pubmed:10921899|pubmed:23256519|pubmed:9601641|pubmed:8638126|pubmed:9556553|pubmed:8259519|pubmed:8259518 R-HSA-5467337 APC truncation mutants have impaired AXIN binding R-HSA-195251 Assembly of the destruction complex Degradation of beta-catenin by the destruction complex R-HSA-195253 1904885 beta-catenin destruction complex assembly nonsense NA

PM1-PM4 APC APC E1309* [cytosol] R-HSA-9666987 APC UniProt:P25054 Nonsense mutation at L-glutamic acid 1309 NonsenseMutation COSMIC:COSV57321386 colorectal cancer DOID:9256 R-HSA-4791256-APC truncation mutants [cytosol]-CandidateSet-hasCandidate R-HSA-4791278 APC truncation mutants have impaired AXIN binding loss_of_function pubmed:10749138|pubmed:8757136|pubmed:11522655|pubmed:9065402|pubmed:22438566|pubmed:10921899|pubmed:23256519|pubmed:9601641|pubmed:8638126|pubmed:9556553|pubmed:8259519|pubmed:8259518 R-HSA-5467337 APC truncation mutants have impaired AXIN binding R-HSA-195251 Assembly of the destruction complex Degradation of beta-catenin by the destruction complex R-HSA-195253 1904885 beta-catenin destruction complex assembly nonsense NA

PM1-PM4 APC APC R302* [cytosol] R-HSA-9666986 APC UniProt:P25054 Nonsense mutation at L-arginine 302 NonsenseMutation COSMIC:COSV57324169 colorectal cancer DOID:9256 R-HSA-4791256-APC truncation mutants [cytosol]-CandidateSet-hasCandidate R-HSA-4791278 APC truncation mutants have impaired AXIN binding loss_of_function pubmed:10749138|pubmed:8757136|pubmed:11522655|pubmed:9065402|pubmed:22438566|pubmed:10921899|pubmed:23256519|pubmed:9601641|pubmed:8638126|pubmed:9556553|pubmed:8259519|pubmed:8259518 R-HSA-5467337 APC truncation mutants have impaired AXIN binding R-HSA-195251 Assembly of the destruction complex Degradation of beta-catenin by the destruction complex R-HSA-195253 1904885 beta-catenin destruction complex assembly nonsense NA

PM1-PM4 APC APC R876* [cytosol] R-HSA-9666985 APC UniProt:P25054 Nonsense mutation at L-arginine 876 NonsenseMutation COSMIC:COSV57320538 colorectal cancer DOID:9256 R-HSA-4791256-APC truncation mutants [cytosol]-CandidateSet-hasCandidate R-HSA-4791278 APC truncation mutants have impaired AXIN binding loss_of_function pubmed:10749138|pubmed:8757136|pubmed:11522655|pubmed:9065402|pubmed:22438566|pubmed:10921899|pubmed:23256519|pubmed:9601641|pubmed:8638126|pubmed:9556553|pubmed:8259519|pubmed:8259518 R-HSA-5467337 APC truncation mutants have impaired AXIN binding R-HSA-195251 Assembly of the destruction complex Degradation of beta-catenin by the destruction complex R-HSA-195253 1904885 beta-catenin destruction complex assembly nonsense NA

PM1-PM4 APC APC K993* [cytosol] R-HSA-9666984 APC UniProt:P25054 Nonsense mutation at L-lysine 993 NonsenseMutation COSMIC:COSV57326945 colorectal cancer DOID:9256 R-HSA-4791256-APC truncation mutants [cytosol]-CandidateSet-hasCandidate R-HSA-4791278 APC truncation mutants have impaired AXIN binding loss_of_function pubmed:10749138|pubmed:8757136|pubmed:11522655|pubmed:9065402|pubmed:22438566|pubmed:10921899|pubmed:23256519|pubmed:9601641|pubmed:8638126|pubmed:9556553|pubmed:8259519|pubmed:8259518 R-HSA-5467337 APC truncation mutants have impaired AXIN binding R-HSA-195251 Assembly of the destruction complex Degradation of beta-catenin by the destruction complex R-HSA-195253 1904885 beta-catenin destruction complex assembly nonsense NA

PS3 APRT APRT D65V [cytosol] R-HSA-9734183 APRT UniProt:P07741 L-aspartic acid 65 replaced with L-valine ReplacedResidue adenine phosphoribosyltransferase deficiency DOID:0060350 pubmed:1746557 R-HSA-9734185-APRT dimer mutants [cytosol]-DefinedSet-hasMember R-HSA-9734193 Defective APRT does not convert adenine to AMP loss_of_function pubmed:7915931|pubmed:3343350|pubmed:1746557|pubmed:865583|pubmed:22700886 R-HSA-9734195 Defective APRT disrupts adenine salvage R-HSA-74213 APRT catalyzes the conversion of adenine to AMP Nucleotide salvage R-HSA-8956321 43173 nucleotide salvage missense loss_of_function of nucleotide salvage

PS3 APRT APRT L110P [cytosol] R-HSA-9734191 APRT UniProt:P07741 L-leucine 110 replaced with L-proline ReplacedResidue adenine phosphoribosyltransferase deficiency DOID:0060350 pubmed:7915931 R-HSA-9734185-APRT dimer mutants [cytosol]-DefinedSet-hasMember R-HSA-9734193 Defective APRT does not convert adenine to AMP loss_of_function pubmed:7915931|pubmed:3343350|pubmed:1746557|pubmed:865583|pubmed:22700886 R-HSA-9734195 Defective APRT disrupts adenine salvage R-HSA-74213 APRT catalyzes the conversion of adenine to AMP Nucleotide salvage R-HSA-8956321 43173 nucleotide salvage missense loss_of_function of nucleotide salvage

PS3 APRT APRT M136T [cytosol] R-HSA-9734184 APRT UniProt:P07741 L-methionine 136 replaced with L-threonine ReplacedResidue adenine phosphoribosyltransferase deficiency DOID:0060350 pubmed:3343350 R-HSA-9734185-APRT dimer mutants [cytosol]-DefinedSet-hasMember R-HSA-9734193 Defective APRT does not convert adenine to AMP loss_of_function pubmed:7915931|pubmed:3343350|pubmed:1746557|pubmed:865583|pubmed:22700886 R-HSA-9734195 Defective APRT disrupts adenine salvage R-HSA-74213 APRT catalyzes the conversion of adenine to AMP Nucleotide salvage R-HSA-8956321 43173 nucleotide salvage missense loss_of_function of nucleotide salvage

PS3 ARSB ARSB Y86del [lysosomal lumen] R-HSA-2314606 ARSB UniProt:P15848 Deletion of residues 86 to 86 FragmentDeletionModification mucopolysaccharidosis VI DOID:12800 R-HSA-2314611-ARSB mutants [lysosomal lumen]-DefinedSet-hasMember R-HSA-9036065|R-HSA-2282889 Defective ARSB does not hydrolyse DS|Defective ARSB does not hydrolyse C4S/C6S chains loss_of_function pubmed:8651289|pubmed:8116615|pubmed:1550123|pubmed:10036316|pubmed:14974081 R-HSA-2206285 MPS VI - Maroteaux-Lamy syndrome R-HSA-1606789|R-HSA-1793207 ARSB hydrolyses DS|ARSB hydrolyses C4S/C6S chains Glycosaminoglycan metabolism R-HSA-1630316 30203 glycosaminoglycan metabolic process in-frame indel: deletion loss_of_function of glycosaminoglycan metabolic process

PS3 ARSB ARSB C117R [lysosomal lumen] R-HSA-2314618 ARSB UniProt:P15848 L-cysteine 117 replaced with L-arginine ReplacedResidue mucopolysaccharidosis VI DOID:12800 R-HSA-2314611-ARSB mutants [lysosomal lumen]-DefinedSet-hasMember R-HSA-9036065|R-HSA-2282889 Defective ARSB does not hydrolyse DS|Defective ARSB does not hydrolyse C4S/C6S chains loss_of_function pubmed:8651289|pubmed:8116615|pubmed:1550123|pubmed:10036316|pubmed:14974081 R-HSA-2206285 MPS VI - Maroteaux-Lamy syndrome R-HSA-1606789|R-HSA-1793207 ARSB hydrolyses DS|ARSB hydrolyses C4S/C6S chains Glycosaminoglycan metabolism R-HSA-1630316 30203 glycosaminoglycan metabolic process missense loss_of_function of glycosaminoglycan metabolic process

PS3 ARSB ARSB P116H [lysosomal lumen] R-HSA-2314619 ARSB UniProt:P15848 L-proline 116 replaced with L-histidine ReplacedResidue mucopolysaccharidosis VI DOID:12800 R-HSA-2314611-ARSB mutants [lysosomal lumen]-DefinedSet-hasMember R-HSA-9036065|R-HSA-2282889 Defective ARSB does not hydrolyse DS|Defective ARSB does not hydrolyse C4S/C6S chains loss_of_function pubmed:8651289|pubmed:8116615|pubmed:1550123|pubmed:10036316|pubmed:14974081 R-HSA-2206285 MPS VI - Maroteaux-Lamy syndrome R-HSA-1606789|R-HSA-1793207 ARSB hydrolyses DS|ARSB hydrolyses C4S/C6S chains Glycosaminoglycan metabolism R-HSA-1630316 30203 glycosaminoglycan metabolic process missense loss_of_function of glycosaminoglycan metabolic process

PS3 ARSB ARSB G144R [lysosomal lumen] R-HSA-2314612 ARSB UniProt:P15848 glycine 144 replaced with L-arginine ReplacedResidue mucopolysaccharidosis VI DOID:12800 R-HSA-2314611-ARSB mutants [lysosomal lumen]-DefinedSet-hasMember R-HSA-9036065|R-HSA-2282889 Defective ARSB does not hydrolyse DS|Defective ARSB does not hydrolyse C4S/C6S chains loss_of_function pubmed:8651289|pubmed:8116615|pubmed:1550123|pubmed:10036316|pubmed:14974081 R-HSA-2206285 MPS VI - Maroteaux-Lamy syndrome R-HSA-1606789|R-HSA-1793207 ARSB hydrolyses DS|ARSB hydrolyses C4S/C6S chains Glycosaminoglycan metabolism R-HSA-1630316 30203 glycosaminoglycan metabolic process missense loss_of_function of glycosaminoglycan metabolic process

PS3 ARSB ARSB R95Q [lysosomal lumen] R-HSA-2314614 ARSB UniProt:P15848 L-arginine 95 replaced with L-glutamine ReplacedResidue mucopolysaccharidosis VI DOID:12800 R-HSA-2314611-ARSB mutants [lysosomal lumen]-DefinedSet-hasMember R-HSA-9036065|R-HSA-2282889 Defective ARSB does not hydrolyse DS|Defective ARSB does not hydrolyse C4S/C6S chains loss_of_function pubmed:8651289|pubmed:8116615|pubmed:1550123|pubmed:10036316|pubmed:14974081 R-HSA-2206285 MPS VI - Maroteaux-Lamy syndrome R-HSA-1606789|R-HSA-1793207 ARSB hydrolyses DS|ARSB hydrolyses C4S/C6S chains Glycosaminoglycan metabolism R-HSA-1630316 30203 glycosaminoglycan metabolic process missense loss_of_function of glycosaminoglycan metabolic process

PS3 ARSB ARSB H393P [lysosomal lumen] R-HSA-5649492 ARSB UniProt:P15848 L-histidine 393 replaced with L-proline ReplacedResidue mucopolysaccharidosis VI DOID:12800 R-HSA-2314611-ARSB mutants [lysosomal lumen]-DefinedSet-hasMember R-HSA-9036065|R-HSA-2282889 Defective ARSB does not hydrolyse DS|Defective ARSB does not hydrolyse C4S/C6S chains loss_of_function pubmed:8651289|pubmed:8116615|pubmed:1550123|pubmed:10036316|pubmed:14974081 R-HSA-2206285 MPS VI - Maroteaux-Lamy syndrome R-HSA-1606789|R-HSA-1793207 ARSB hydrolyses DS|ARSB hydrolyses C4S/C6S chains Glycosaminoglycan metabolism R-HSA-1630316 30203 glycosaminoglycan metabolic process missense loss_of_function of glycosaminoglycan metabolic process

PM5 ATG7 ATG7(1-693)-p-BRAF(381-766) fusion [cytosol] R-HSA-6802517 ATG7 UniProt:O95352 Insertion of residues 381 to 766 at 694 from UniProt:P15056 BRAF FragmentInsertionModification cancer|skin melanoma DOID:162|DOID:8923 pubmed:25204415 R-HSA-6802698-p-BRAF/RAF fusion dimers [cytosol]-CandidateSet-hasCandidate R-HSA-6802935|R-HSA-6802933|R-HSA-6802934|R-HSA-6802927|R-HSA-6802932 MAPKs are phosphorylated downstream of BRAF and RAF fusion dimers|p-BRAF and RAF fusion dimers phosphorylate MAP2Ks|p-BRAF and RAF fusion dimers bind MAP2Ks and MAPKs|BRAF and RAF fusion mutant dimers are phosphorylated|Dissociation of BRAF/RAF fusion complex gain_of_function pubmed:24345920|pubmed:15630448|pubmed:25907612|pubmed:21424530|pubmed:20526349|pubmed:18974108|pubmed:24135138|pubmed:25204415|pubmed:22745804|pubmed:23583981 R-HSA-6802952 Signaling by BRAF and RAF1 fusions R-HSA-5672973|R-HSA-5672978|R-HSA-5672972|R-HSA-5672969|R-HSA-5672980 MAP2Ks phosphorylate MAPKs|RAF phosphorylates MAP2K dimer|MAP2Ks and MAPKs bind to the activated RAF complex|Phosphorylation of RAF|Dissociation of RAS:RAF complex RAF/MAP kinase cascade R-HSA-5673001 165 MAPK cascade fusion gain_of_function of MAPK cascade

PM5 ATG7 ATG7(1-693)-BRAF(381-766) fusion [cytosol] R-HSA-6802272 ATG7 UniProt:O95352 Insertion of residues 381 to 766 at 694 from UniProt:P15056 BRAF FragmentInsertionModification cancer|skin melanoma DOID:162|DOID:8923 pubmed:25204415 R-HSA-6802720-BRAF/RAF fusion dimers [cytosol]-CandidateSet-hasCandidate R-HSA-6802927 BRAF and RAF fusion mutant dimers are phosphorylated gain_of_function pubmed:24345920|pubmed:15630448|pubmed:25907612|pubmed:21424530|pubmed:20526349|pubmed:18974108|pubmed:24135138|pubmed:25204415|pubmed:22745804|pubmed:23583981 R-HSA-6802952 Signaling by BRAF and RAF1 fusions R-HSA-5672969 Phosphorylation of RAF RAF/MAP kinase cascade R-HSA-5673001 165 MAPK cascade fusion gain_of_function of MAPK cascade

PS3 ATIC ATIC(1-230)-p-7Y-ALK(1059-1620) fusion [cytosol] R-HSA-9710833 ATIC UniProt:P31939 Insertion of residues 1059 to 1620 at 231 from UniProt:Q9UM73 ALK FragmentInsertionModification anaplastic large cell lymphoma DOID:0050744 pubmed:10706887|pubmed:10706082 R-HSA-9699980-p-7Y ALK fusion protein dimers [cytosol]-CandidateSet-hasMember|R-HSA-9710874-GRB2- and PLC gamma-binding ALK fusion protein dimers [cytosol]-CandidateSet-hasMember|R-HSA-9713538-SHC-binding ALK mutant dimers [cytosol]-CandidateSet-hasMember R-HSA-9700181|R-HSA-9712086|R-HSA-9710917|R-HSA-9700190|R-HSA-9700193|R-HSA-9710914|R-HSA-9724099 Autophosphorylation of ALK fusions|ALK fusions phosphorylate PLCG1|ALK fusion proteins bind PLCG1|ALK mutants bind SHC|ALK mutants phosphorylate SHC1|ALK fusions bind GRB2|ALK mutants:p-3Y SHC binds GRB2 pubmed:27573755|pubmed:29455642|pubmed:23201355|pubmed:19459784|pubmed:18097461|pubmed:9819383|pubmed:11943732|pubmed:31366041|pubmed:24060861|pubmed:10706082|pubmed:12185581|pubmed:8633037|pubmed:22249260|pubmed:22570254|pubmed:23139213|pubmed:26301689|pubmed:17483340 R-HSA-9725370 Signaling by ALK fusions and activated point mutants R-HSA-201521|R-HSA-9700171 ALK autophosphorylation downstream of PTN and MDK|Active ALK phosphorylates PLCG1 Signaling by ALK R-HSA-201556 fusion NA

PM5 ATIC ATIC(1-229)-p-7Y-ALK(1058-1620) fusion [cytosol] R-HSA-9710847 ATIC UniProt:P31939 Insertion of residues 1058 to 1620 at 230 from UniProt:Q9UM73 ALK FragmentInsertionModification anaplastic large cell lymphoma DOID:0050744 pubmed:10702393 R-HSA-9699980-p-7Y ALK fusion protein dimers [cytosol]-CandidateSet-hasCandidate|R-HSA-9710874-GRB2- and PLC gamma-binding ALK fusion protein dimers [cytosol]-CandidateSet-hasCandidate|R-HSA-9713538-SHC-binding ALK mutant dimers [cytosol]-CandidateSet-hasCandidate R-HSA-9700181|R-HSA-9712086|R-HSA-9710917|R-HSA-9700190|R-HSA-9700193|R-HSA-9710914|R-HSA-9724099 Autophosphorylation of ALK fusions|ALK fusions phosphorylate PLCG1|ALK fusion proteins bind PLCG1|ALK mutants bind SHC|ALK mutants phosphorylate SHC1|ALK fusions bind GRB2|ALK mutants:p-3Y SHC binds GRB2 pubmed:27573755|pubmed:29455642|pubmed:23201355|pubmed:19459784|pubmed:18097461|pubmed:9819383|pubmed:11943732|pubmed:31366041|pubmed:24060861|pubmed:10706082|pubmed:12185581|pubmed:8633037|pubmed:22249260|pubmed:22570254|pubmed:23139213|pubmed:26301689|pubmed:17483340 R-HSA-9725370 Signaling by ALK fusions and activated point mutants R-HSA-201521|R-HSA-9700171 ALK autophosphorylation downstream of PTN and MDK|Active ALK phosphorylates PLCG1 Signaling by ALK R-HSA-201556 fusion NA

PS3-PS1 ATIC ATIC(1-230)-ALK(1059-1620) fusion [cytosol] R-HSA-9710832 ATIC UniProt:P31939 Insertion of residues 1059 to 1620 at 231 from UniProt:Q9UM73 ALK FragmentInsertionModification COSMIC:COSF446|COSMIC:COSF444 anaplastic large cell lymphoma DOID:0050744 pubmed:10706887|pubmed:10706082 R-HSA-9700079-ALK fusion protein dimers [cytosol]-CandidateSet-hasMember|R-HSA-9700080-ALK fusion proteins [cytosol]-CandidateSet-hasMember R-HSA-9700181|R-HSA-9700179 Autophosphorylation of ALK fusions|Ligand-independent dimerization of ALK fusions pubmed:27573755|pubmed:29455642|pubmed:23201355|pubmed:22347464|pubmed:28659337|pubmed:24613930|pubmed:28741662|pubmed:12920229|pubmed:10702393|pubmed:21164297|pubmed:26062823|pubmed:11943732|pubmed:24445538|pubmed:10934142|pubmed:25813404|pubmed:12750159|pubmed:21134980|pubmed:27874193|pubmed:27245569|pubmed:21415216|pubmed:8633037|pubmed:17625570|pubmed:22327622|pubmed:28404650|pubmed:24475247|pubmed:10556217|pubmed:18701132|pubmed:23661334|pubmed:24736082|pubmed:21213368|pubmed:15208656|pubmed:24518094|pubmed:23957430|pubmed:26001147|pubmed:21656749|pubmed:18593892|pubmed:10216106|pubmed:10706082|pubmed:10706887|pubmed:25393796|pubmed:12112524|pubmed:24060861|pubmed:22658521|pubmed:24496003|pubmed:7824924|pubmed:16161041|pubmed:21494621|pubmed:11310834 R-HSA-9725370 Signaling by ALK fusions and activated point mutants R-HSA-201521|R-HSA-201519 ALK autophosphorylation downstream of PTN and MDK|ALK:PTN dimerization Signaling by ALK R-HSA-201556 fusion NA

PM5 ATIC ATIC(1-229)-ALK(1058-1620) fusion [cytosol] R-HSA-9710829 ATIC UniProt:P31939 Insertion of residues 1058 to 1620 at 230 from UniProt:Q9UM73 ALK FragmentInsertionModification COSMIC:COSF444 anaplastic large cell lymphoma DOID:0050744 pubmed:10702393 R-HSA-9700079-ALK fusion protein dimers [cytosol]-CandidateSet-hasCandidate|R-HSA-9700080-ALK fusion proteins [cytosol]-CandidateSet-hasCandidate R-HSA-9700181|R-HSA-9700179 Autophosphorylation of ALK fusions|Ligand-independent dimerization of ALK fusions pubmed:27573755|pubmed:29455642|pubmed:23201355|pubmed:22347464|pubmed:28659337|pubmed:24613930|pubmed:28741662|pubmed:12920229|pubmed:10702393|pubmed:21164297|pubmed:26062823|pubmed:11943732|pubmed:24445538|pubmed:10934142|pubmed:25813404|pubmed:12750159|pubmed:21134980|pubmed:27874193|pubmed:27245569|pubmed:21415216|pubmed:8633037|pubmed:17625570|pubmed:22327622|pubmed:28404650|pubmed:24475247|pubmed:10556217|pubmed:18701132|pubmed:23661334|pubmed:24736082|pubmed:21213368|pubmed:15208656|pubmed:24518094|pubmed:23957430|pubmed:26001147|pubmed:21656749|pubmed:18593892|pubmed:10216106|pubmed:10706082|pubmed:10706887|pubmed:25393796|pubmed:12112524|pubmed:24060861|pubmed:22658521|pubmed:24496003|pubmed:7824924|pubmed:16161041|pubmed:21494621|pubmed:11310834 R-HSA-9725370 Signaling by ALK fusions and activated point mutants R-HSA-201521|R-HSA-201519 ALK autophosphorylation downstream of PTN and MDK|ALK:PTN dimerization Signaling by ALK R-HSA-201556 fusion NA

PS3 ATRX ATRX E288* [nucleoplasm] R-HSA-9670958 ATRX UniProt:P46100 Nonsense mutation at L-glutamic acid 288 NonsenseMutation COSMIC:COSV64881511 paraganglioma DOID:0050773 pubmed:14990586 R-HSA-9670618-ATRX truncation mutants (DAXX binding region) [nucleoplasm]-CandidateSet-hasMember R-HSA-9670619 Defective ATRX does not bind DAXX loss_of_function pubmed:28875424|pubmed:14990586|pubmed:21252315 R-HSA-9670615 Defective Inhibition of DNA Recombination at Telomere Due to ATRX Mutations R-HSA-9007926 ATRX binds DAXX Telomere Maintenance R-HSA-157579 723 telomere maintenance nonsense loss_of_function of telomere maintenance

PS3 ATRX ATRX K329Ifs*3 [nucleoplasm] R-HSA-9672232 ATRX UniProt:P46100 Replacement of residues 329 to 330 by IR FragmentReplacedModification COSMIC:COSV64871958 cancer|astrocytoma DOID:162|DOID:3069 pubmed:14990586 R-HSA-9670618-ATRX truncation mutants (DAXX binding region) [nucleoplasm]-CandidateSet-hasMember R-HSA-9670619 Defective ATRX does not bind DAXX loss_of_function pubmed:28875424|pubmed:14990586|pubmed:21252315 R-HSA-9670615 Defective Inhibition of DNA Recombination at Telomere Due to ATRX Mutations R-HSA-9007926 ATRX binds DAXX Telomere Maintenance R-HSA-157579 723 telomere maintenance frameshift loss_of_function of telomere maintenance

PS3 ATRX ATRX Q176* [nucleoplasm] R-HSA-9671592 ATRX UniProt:P46100 Nonsense mutation at L-glutamine 176 NonsenseMutation COSMIC:COSV64870354 cancer|astrocytoma DOID:162|DOID:3069 pubmed:14990586 R-HSA-9670618-ATRX truncation mutants (DAXX binding region) [nucleoplasm]-CandidateSet-hasMember R-HSA-9670619 Defective ATRX does not bind DAXX loss_of_function pubmed:28875424|pubmed:14990586|pubmed:21252315 R-HSA-9670615 Defective Inhibition of DNA Recombination at Telomere Due to ATRX Mutations R-HSA-9007926 ATRX binds DAXX Telomere Maintenance R-HSA-157579 723 telomere maintenance nonsense loss_of_function of telomere maintenance

PS3 ATRX ATRX C268Qfs*18 [nucleoplasm] R-HSA-9672233 ATRX UniProt:P46100 Replacement of residues 268 to 284 by QSLCWTWSLHVTAYLRI FragmentReplacedModification COSMIC:COSV64874030 brain oligodendroglioma|cancer DOID:3187|DOID:162 pubmed:14990586 R-HSA-9670618-ATRX truncation mutants (DAXX binding region) [nucleoplasm]-CandidateSet-hasMember R-HSA-9670619 Defective ATRX does not bind DAXX loss_of_function pubmed:28875424|pubmed:14990586|pubmed:21252315 R-HSA-9670615 Defective Inhibition of DNA Recombination at Telomere Due to ATRX Mutations R-HSA-9007926 ATRX binds DAXX Telomere Maintenance R-HSA-157579 723 telomere maintenance frameshift loss_of_function of telomere maintenance

PS3 ATRX ATRX Q177* [nucleoplasm] R-HSA-9671589 ATRX UniProt:P46100 Nonsense mutation at L-glutamine 177 NonsenseMutation COSMIC:COSV64869924 cancer|astrocytoma DOID:162|DOID:3069 pubmed:14990586 R-HSA-9670618-ATRX truncation mutants (DAXX binding region) [nucleoplasm]-CandidateSet-hasMember R-HSA-9670619 Defective ATRX does not bind DAXX loss_of_function pubmed:28875424|pubmed:14990586|pubmed:21252315 R-HSA-9670615 Defective Inhibition of DNA Recombination at Telomere Due to ATRX Mutations R-HSA-9007926 ATRX binds DAXX Telomere Maintenance R-HSA-157579 723 telomere maintenance nonsense loss_of_function of telomere maintenance

PS3 ATRX ATRX K330Nfs*2 [nucleoplasm] R-HSA-9672231 ATRX UniProt:P46100 Replacement of residues 330 to 330 by N FragmentReplacedModification COSMIC:COSV64882726 cancer|astrocytoma DOID:162|DOID:3069 pubmed:14990586 R-HSA-9670618-ATRX truncation mutants (DAXX binding region) [nucleoplasm]-CandidateSet-hasMember R-HSA-9670619 Defective ATRX does not bind DAXX loss_of_function pubmed:28875424|pubmed:14990586|pubmed:21252315 R-HSA-9670615 Defective Inhibition of DNA Recombination at Telomere Due to ATRX Mutations R-HSA-9007926 ATRX binds DAXX Telomere Maintenance R-HSA-157579 723 telomere maintenance frameshift loss_of_function of telomere maintenance

PS3 ATRX ATRX G161* [nucleoplasm] R-HSA-9671266 ATRX UniProt:P46100 Nonsense mutation at glycine 161 NonsenseMutation COSMIC:COSV64869716 lung adenocarcinoma|cancer|lung small cell carcinoma DOID:3910|DOID:162|DOID:5409 pubmed:14990586 R-HSA-9670618-ATRX truncation mutants (DAXX binding region) [nucleoplasm]-CandidateSet-hasMember R-HSA-9670619 Defective ATRX does not bind DAXX loss_of_function pubmed:28875424|pubmed:14990586|pubmed:21252315 R-HSA-9670615 Defective Inhibition of DNA Recombination at Telomere Due to ATRX Mutations R-HSA-9007926 ATRX binds DAXX Telomere Maintenance R-HSA-157579 723 telomere maintenance nonsense loss_of_function of telomere maintenance

PS3 ATRX ATRX Q176Hfs*13 [nucleoplasm] R-HSA-9671806 ATRX UniProt:P46100 Replacement of residues 176 to 187 by HTGQSFSKRFHL FragmentReplacedModification COSMIC:COSV64869293 stomach cancer|cancer DOID:10534|DOID:162 pubmed:14990586 R-HSA-9670618-ATRX truncation mutants (DAXX binding region) [nucleoplasm]-CandidateSet-hasMember R-HSA-9670619 Defective ATRX does not bind DAXX loss_of_function pubmed:28875424|pubmed:14990586|pubmed:21252315 R-HSA-9670615 Defective Inhibition of DNA Recombination at Telomere Due to ATRX Mutations R-HSA-9007926 ATRX binds DAXX Telomere Maintenance R-HSA-157579 723 telomere maintenance frameshift loss_of_function of telomere maintenance

PS3 ATRX ATRX R160Pfs*29 [nucleoplasm] R-HSA-9671801 ATRX UniProt:P46100 Replacement of residues 160 to 187 by PRRRWASWDCELHCLWTTGQSFSKRFHL FragmentReplacedModification COSMIC:COSV64870011 cancer|large intestine cancer DOID:162|DOID:5672 pubmed:14990586 R-HSA-9670618-ATRX truncation mutants (DAXX binding region) [nucleoplasm]-CandidateSet-hasMember R-HSA-9670619 Defective ATRX does not bind DAXX loss_of_function pubmed:28875424|pubmed:14990586|pubmed:21252315 R-HSA-9670615 Defective Inhibition of DNA Recombination at Telomere Due to ATRX Mutations R-HSA-9007926 ATRX binds DAXX Telomere Maintenance R-HSA-157579 723 telomere maintenance frameshift loss_of_function of telomere maintenance

PS3 ATRX ATRX K319* [nucleoplasm] R-HSA-9671669 ATRX UniProt:P46100 Nonsense mutation at L-lysine 319 NonsenseMutation COSMIC:COSV64880901 cancer|astrocytoma DOID:162|DOID:3069 pubmed:14990586 R-HSA-9670618-ATRX truncation mutants (DAXX binding region) [nucleoplasm]-CandidateSet-hasMember R-HSA-9670619 Defective ATRX does not bind DAXX loss_of_function pubmed:28875424|pubmed:14990586|pubmed:21252315 R-HSA-9670615 Defective Inhibition of DNA Recombination at Telomere Due to ATRX Mutations R-HSA-9007926 ATRX binds DAXX Telomere Maintenance R-HSA-157579 723 telomere maintenance nonsense loss_of_function of telomere maintenance

PS3 ATRX ATRX W222* [nucleoplasm] R-HSA-9671670 ATRX UniProt:P46100 Nonsense mutation at L-tryptophan 222 NonsenseMutation COSMIC:COSV64880098 cancer|astrocytoma DOID:162|DOID:3069 pubmed:14990586 R-HSA-9670618-ATRX truncation mutants (DAXX binding region) [nucleoplasm]-CandidateSet-hasMember R-HSA-9670619 Defective ATRX does not bind DAXX loss_of_function pubmed:28875424|pubmed:14990586|pubmed:21252315 R-HSA-9670615 Defective Inhibition of DNA Recombination at Telomere Due to ATRX Mutations R-HSA-9007926 ATRX binds DAXX Telomere Maintenance R-HSA-157579 723 telomere maintenance nonsense loss_of_function of telomere maintenance

PS3 ATRX ATRX E63* [nucleoplasm] R-HSA-9670961 ATRX UniProt:P46100 Nonsense mutation at L-glutamic acid 63 NonsenseMutation COSMIC:COSV64869650 thyroid cancer|cancer|breast cancer DOID:1781|DOID:162|DOID:1612 pubmed:14990586 R-HSA-9670618-ATRX truncation mutants (DAXX binding region) [nucleoplasm]-CandidateSet-hasMember R-HSA-9670619 Defective ATRX does not bind DAXX loss_of_function pubmed:28875424|pubmed:14990586|pubmed:21252315 R-HSA-9670615 Defective Inhibition of DNA Recombination at Telomere Due to ATRX Mutations R-HSA-9007926 ATRX binds DAXX Telomere Maintenance R-HSA-157579 723 telomere maintenance nonsense loss_of_function of telomere maintenance

PS3 ATRX ATRX S79* [nucleoplasm] R-HSA-9671244 ATRX UniProt:P46100 Nonsense mutation at L-serine 79 NonsenseMutation COSMIC:COSV64870029 esophagus squamous cell carcinoma|myelodysplastic syndrome|cancer DOID:3748|DOID:0050908|DOID:162 pubmed:14990586 R-HSA-9670618-ATRX truncation mutants (DAXX binding region) [nucleoplasm]-CandidateSet-hasMember R-HSA-9670619 Defective ATRX does not bind DAXX loss_of_function pubmed:28875424|pubmed:14990586|pubmed:21252315 R-HSA-9670615 Defective Inhibition of DNA Recombination at Telomere Due to ATRX Mutations R-HSA-9007926 ATRX binds DAXX Telomere Maintenance R-HSA-157579 723 telomere maintenance nonsense loss_of_function of telomere maintenance

PS3 ATRX ATRX H166Mfs*4 [nucleoplasm] R-HSA-9672205 ATRX UniProt:P46100 Replacement of residues 166 to 168 by MGL FragmentReplacedModification COSMIC:COSV64869199 cancer|astrocytoma DOID:162|DOID:3069 pubmed:14990586 R-HSA-9670618-ATRX truncation mutants (DAXX binding region) [nucleoplasm]-CandidateSet-hasMember R-HSA-9670619 Defective ATRX does not bind DAXX loss_of_function pubmed:28875424|pubmed:14990586|pubmed:21252315 R-HSA-9670615 Defective Inhibition of DNA Recombination at Telomere Due to ATRX Mutations R-HSA-9007926 ATRX binds DAXX Telomere Maintenance R-HSA-157579 723 telomere maintenance frameshift loss_of_function of telomere maintenance

PS3 ATRX ATRX Y266* [nucleoplasm] R-HSA-9671624 ATRX UniProt:P46100 Nonsense mutation at L-tyrosine 266 NonsenseMutation COSMIC:COSV64871796 cancer|osteosarcoma DOID:162|DOID:3347 pubmed:14990586 R-HSA-9670618-ATRX truncation mutants (DAXX binding region) [nucleoplasm]-CandidateSet-hasMember R-HSA-9670619 Defective ATRX does not bind DAXX loss_of_function pubmed:28875424|pubmed:14990586|pubmed:21252315 R-HSA-9670615 Defective Inhibition of DNA Recombination at Telomere Due to ATRX Mutations R-HSA-9007926 ATRX binds DAXX Telomere Maintenance R-HSA-157579 723 telomere maintenance nonsense loss_of_function of telomere maintenance

PS3 ATRX ATRX L253* [nucleoplasm] R-HSA-9671688 ATRX UniProt:P46100 Nonsense mutation at L-leucine 253 NonsenseMutation COSMIC:COSV64871392 cancer|astrocytoma DOID:162|DOID:3069 pubmed:14990586 R-HSA-9670618-ATRX truncation mutants (DAXX binding region) [nucleoplasm]-CandidateSet-hasMember R-HSA-9670619 Defective ATRX does not bind DAXX loss_of_function pubmed:28875424|pubmed:14990586|pubmed:21252315 R-HSA-9670615 Defective Inhibition of DNA Recombination at Telomere Due to ATRX Mutations R-HSA-9007926 ATRX binds DAXX Telomere Maintenance R-HSA-157579 723 telomere maintenance nonsense loss_of_function of telomere maintenance

PS3 ATRX ATRX W263* [nucleoplasm] R-HSA-9671685 ATRX UniProt:P46100 Nonsense mutation at L-tryptophan 263 NonsenseMutation COSMIC:COSV64878305 cancer|astrocytoma DOID:162|DOID:3069 pubmed:14990586 R-HSA-9670618-ATRX truncation mutants (DAXX binding region) [nucleoplasm]-CandidateSet-hasMember R-HSA-9670619 Defective ATRX does not bind DAXX loss_of_function pubmed:28875424|pubmed:14990586|pubmed:21252315 R-HSA-9670615 Defective Inhibition of DNA Recombination at Telomere Due to ATRX Mutations R-HSA-9007926 ATRX binds DAXX Telomere Maintenance R-HSA-157579 723 telomere maintenance nonsense loss_of_function of telomere maintenance

PS3 ATRX ATRX Y187* [nucleoplasm] R-HSA-9671622 ATRX UniProt:P46100 Nonsense mutation at L-tyrosine 187 NonsenseMutation COSMIC:COSV64869221 cancer|astrocytoma DOID:162|DOID:3069 pubmed:14990586 R-HSA-9670618-ATRX truncation mutants (DAXX binding region) [nucleoplasm]-CandidateSet-hasMember R-HSA-9670619 Defective ATRX does not bind DAXX loss_of_function pubmed:28875424|pubmed:14990586|pubmed:21252315 R-HSA-9670615 Defective Inhibition of DNA Recombination at Telomere Due to ATRX Mutations R-HSA-9007926 ATRX binds DAXX Telomere Maintenance R-HSA-157579 723 telomere maintenance nonsense loss_of_function of telomere maintenance

PS3 ATRX ATRX Y204* [nucleoplasm] R-HSA-9671619 ATRX UniProt:P46100 Nonsense mutation at L-tyrosine 204 NonsenseMutation COSMIC:COSV64873595 cancer|brain glioma DOID:162|DOID:0060108 pubmed:14990586 R-HSA-9670618-ATRX truncation mutants (DAXX binding region) [nucleoplasm]-CandidateSet-hasMember R-HSA-9670619 Defective ATRX does not bind DAXX loss_of_function pubmed:28875424|pubmed:14990586|pubmed:21252315 R-HSA-9670615 Defective Inhibition of DNA Recombination at Telomere Due to ATRX Mutations R-HSA-9007926 ATRX binds DAXX Telomere Maintenance R-HSA-157579 723 telomere maintenance nonsense loss_of_function of telomere maintenance

PS3 ATRX ATRX Q292* [nucleoplasm] R-HSA-9671581 ATRX UniProt:P46100 Nonsense mutation at L-glutamine 292 NonsenseMutation COSMIC:COSV64875650 cancer|brain glioma|astrocytoma DOID:162|DOID:0060108|DOID:3069 pubmed:14990586 R-HSA-9670618-ATRX truncation mutants (DAXX binding region) [nucleoplasm]-CandidateSet-hasMember R-HSA-9670619 Defective ATRX does not bind DAXX loss_of_function pubmed:28875424|pubmed:14990586|pubmed:21252315 R-HSA-9670615 Defective Inhibition of DNA Recombination at Telomere Due to ATRX Mutations R-HSA-9007926 ATRX binds DAXX Telomere Maintenance R-HSA-157579 723 telomere maintenance nonsense loss_of_function of telomere maintenance

PS3 ATRX ATRX P190Hfs*15 [nucleoplasm] R-HSA-9672216 ATRX UniProt:P46100 Replacement of residues 190 to 203 by HCKFLFVRIALSIT FragmentReplacedModification COSMIC:COSV64869753 cancer|gastric neuroendocrine neoplasm DOID:162|DOID:4715 pubmed:14990586 R-HSA-9670618-ATRX truncation mutants (DAXX binding region) [nucleoplasm]-CandidateSet-hasMember R-HSA-9670619 Defective ATRX does not bind DAXX loss_of_function pubmed:28875424|pubmed:14990586|pubmed:21252315 R-HSA-9670615 Defective Inhibition of DNA Recombination at Telomere Due to ATRX Mutations R-HSA-9007926 ATRX binds DAXX Telomere Maintenance R-HSA-157579 723 telomere maintenance frameshift loss_of_function of telomere maintenance

PS3 ATRX ATRX L274Ffs*8 [nucleoplasm] R-HSA-9671833 ATRX UniProt:P46100 Replacement of residues 274 to 280 by FGLGHCM FragmentReplacedModification COSMIC:COSV64870879 cancer|astrocytoma DOID:162|DOID:3069 pubmed:14990586 R-HSA-9670618-ATRX truncation mutants (DAXX binding region) [nucleoplasm]-CandidateSet-hasMember R-HSA-9670619 Defective ATRX does not bind DAXX loss_of_function pubmed:28875424|pubmed:14990586|pubmed:21252315 R-HSA-9670615 Defective Inhibition of DNA Recombination at Telomere Due to ATRX Mutations R-HSA-9007926 ATRX binds DAXX Telomere Maintenance R-HSA-157579 723 telomere maintenance frameshift loss_of_function of telomere maintenance

PS3 ATRX ATRX Q193* [nucleoplasm] R-HSA-9671579 ATRX UniProt:P46100 Nonsense mutation at L-glutamine 193 NonsenseMutation COSMIC:COSV64869800 cancer|breast cancer DOID:162|DOID:1612 pubmed:14990586 R-HSA-9670618-ATRX truncation mutants (DAXX binding region) [nucleoplasm]-CandidateSet-hasMember R-HSA-9670619 Defective ATRX does not bind DAXX loss_of_function pubmed:28875424|pubmed:14990586|pubmed:21252315 R-HSA-9670615 Defective Inhibition of DNA Recombination at Telomere Due to ATRX Mutations R-HSA-9007926 ATRX binds DAXX Telomere Maintenance R-HSA-157579 723 telomere maintenance nonsense loss_of_function of telomere maintenance

PS3 ATRX ATRX R188* [nucleoplasm] R-HSA-9671637 ATRX UniProt:P46100 Nonsense mutation at L-arginine 188 NonsenseMutation COSMIC:COSV64869867 cancer|astrocytoma DOID:162|DOID:3069 pubmed:14990586 R-HSA-9670618-ATRX truncation mutants (DAXX binding region) [nucleoplasm]-CandidateSet-hasMember R-HSA-9670619 Defective ATRX does not bind DAXX loss_of_function pubmed:28875424|pubmed:14990586|pubmed:21252315 R-HSA-9670615 Defective Inhibition of DNA Recombination at Telomere Due to ATRX Mutations R-HSA-9007926 ATRX binds DAXX Telomere Maintenance R-HSA-157579 723 telomere maintenance nonsense loss_of_function of telomere maintenance

PS3 ATRX ATRX S213* [nucleoplasm] R-HSA-9671255 ATRX UniProt:P46100 Nonsense mutation at L-serine 213 NonsenseMutation COSMIC:COSV64875010 cancer|osteosarcoma DOID:162|DOID:3347 pubmed:14990586 R-HSA-9670618-ATRX truncation mutants (DAXX binding region) [nucleoplasm]-CandidateSet-hasMember R-HSA-9670619 Defective ATRX does not bind DAXX loss_of_function pubmed:28875424|pubmed:14990586|pubmed:21252315 R-HSA-9670615 Defective Inhibition of DNA Recombination at Telomere Due to ATRX Mutations R-HSA-9007926 ATRX binds DAXX Telomere Maintenance R-HSA-157579 723 telomere maintenance nonsense loss_of_function of telomere maintenance

PS3 ATRX ATRX M205* [nucleoplasm] R-HSA-9672215 ATRX UniProt:P46100 Nonsense mutation at L-methionine 205 NonsenseMutation COSMIC:COSV64884053 cancer|brain glioma DOID:162|DOID:0060108 pubmed:14990586 R-HSA-9670618-ATRX truncation mutants (DAXX binding region) [nucleoplasm]-CandidateSet-hasMember R-HSA-9670619 Defective ATRX does not bind DAXX loss_of_function pubmed:28875424|pubmed:14990586|pubmed:21252315 R-HSA-9670615 Defective Inhibition of DNA Recombination at Telomere Due to ATRX Mutations R-HSA-9007926 ATRX binds DAXX Telomere Maintenance R-HSA-157579 723 telomere maintenance nonsense loss_of_function of telomere maintenance

PS3 ATRX ATRX L273Ffs*9 [nucleoplasm] R-HSA-9671825 ATRX UniProt:P46100 Replacement of residues 273 to 280 by FVGLGHCM FragmentReplacedModification COSMIC:COSV64879786 cancer|astrocytoma DOID:162|DOID:3069 pubmed:14990586 R-HSA-9670618-ATRX truncation mutants (DAXX binding region) [nucleoplasm]-CandidateSet-hasMember R-HSA-9670619 Defective ATRX does not bind DAXX loss_of_function pubmed:28875424|pubmed:14990586|pubmed:21252315 R-HSA-9670615 Defective Inhibition of DNA Recombination at Telomere Due to ATRX Mutations R-HSA-9007926 ATRX binds DAXX Telomere Maintenance R-HSA-157579 723 telomere maintenance frameshift loss_of_function of telomere maintenance

PS3 ATRX ATRX R250* [nucleoplasm] R-HSA-9671634 ATRX UniProt:P46100 Nonsense mutation at L-arginine 250 NonsenseMutation COSMIC:COSV64873935 histiocytoma|cancer|skin melanoma|cervix small cell carcinoma|islet cell tumor|skin squamous cell carcinoma|retroperitoneal neuroblastoma DOID:4231|DOID:162|DOID:8923|DOID:6740|DOID:1799|DOID:3151|DOID:0050937 pubmed:14990586 R-HSA-9670618-ATRX truncation mutants (DAXX binding region) [nucleoplasm]-CandidateSet-hasMember R-HSA-9670619 Defective ATRX does not bind DAXX loss_of_function pubmed:28875424|pubmed:14990586|pubmed:21252315 R-HSA-9670615 Defective Inhibition of DNA Recombination at Telomere Due to ATRX Mutations R-HSA-9007926 ATRX binds DAXX Telomere Maintenance R-HSA-157579 723 telomere maintenance nonsense loss_of_function of telomere maintenance

PS3 ATRX ATRX D184Ifs*22 [nucleoplasm] R-HSA-9672211 ATRX UniProt:P46100 Replacement of residues 184 to 204 by IPFIDTLHCKFLFVRIALSIT FragmentReplacedModification COSMIC:COSV64869827 cancer|brain glioma|astrocytoma DOID:162|DOID:0060108|DOID:3069 pubmed:14990586 R-HSA-9670618-ATRX truncation mutants (DAXX binding region) [nucleoplasm]-CandidateSet-hasMember R-HSA-9670619 Defective ATRX does not bind DAXX loss_of_function pubmed:28875424|pubmed:14990586|pubmed:21252315 R-HSA-9670615 Defective Inhibition of DNA Recombination at Telomere Due to ATRX Mutations R-HSA-9007926 ATRX binds DAXX Telomere Maintenance R-HSA-157579 723 telomere maintenance frameshift loss_of_function of telomere maintenance

PM1-PM4 ATRX ATRX V478Ffs*36 [nucleoplasm] R-HSA-9672558 ATRX UniProt:P46100 Replacement of residues 478 to 512 by FQQRNKEQIKVPVVNIRNLIEKKNLNMNLPTLLKI FragmentReplacedModification COSMIC:COSV64873848 cancer|brain glioma DOID:162|DOID:0060108 R-HSA-9670618-ATRX truncation mutants (DAXX binding region) [nucleoplasm]-CandidateSet-hasCandidate R-HSA-9670619 Defective ATRX does not bind DAXX loss_of_function pubmed:28875424|pubmed:14990586|pubmed:21252315 R-HSA-9670615 Defective Inhibition of DNA Recombination at Telomere Due to ATRX Mutations R-HSA-9007926 ATRX binds DAXX Telomere Maintenance R-HSA-157579 723 telomere maintenance frameshift loss_of_function of telomere maintenance

PM1-PM4 ATRX ATRX T534Wfs*3 [nucleoplasm] R-HSA-9672553 ATRX UniProt:P46100 Replacement of residues 534 to 535 by WN FragmentReplacedModification COSMIC:COSV64883111 cancer|astrocytoma DOID:162|DOID:3069 R-HSA-9670618-ATRX truncation mutants (DAXX binding region) [nucleoplasm]-CandidateSet-hasCandidate R-HSA-9670619 Defective ATRX does not bind DAXX loss_of_function pubmed:28875424|pubmed:14990586|pubmed:21252315 R-HSA-9670615 Defective Inhibition of DNA Recombination at Telomere Due to ATRX Mutations R-HSA-9007926 ATRX binds DAXX Telomere Maintenance R-HSA-157579 723 telomere maintenance frameshift loss_of_function of telomere maintenance

PM1-PM4 ATRX ATRX G859Rfs*4 [nucleoplasm] R-HSA-9672298 ATRX UniProt:P46100 Replacement of residues 859 to 861 by RNG FragmentReplacedModification COSMIC:COSV64878899 cancer|breast cancer|large intestine cancer DOID:162|DOID:1612|DOID:5672 R-HSA-9670618-ATRX truncation mutants (DAXX binding region) [nucleoplasm]-CandidateSet-hasCandidate R-HSA-9670619 Defective ATRX does not bind DAXX loss_of_function pubmed:28875424|pubmed:14990586|pubmed:21252315 R-HSA-9670615 Defective Inhibition of DNA Recombination at Telomere Due to ATRX Mutations R-HSA-9007926 ATRX binds DAXX Telomere Maintenance R-HSA-157579 723 telomere maintenance frameshift loss_of_function of telomere maintenance

PM1-PM4 ATRX ATRX T792Ifs*12 [nucleoplasm] R-HSA-9672299 ATRX UniProt:P46100 Replacement of residues 792 to 802 by ILKRANQLRAL FragmentReplacedModification COSMIC:COSV64871353 leiomyosarcoma|cancer DOID:1967|DOID:162 R-HSA-9670618-ATRX truncation mutants (DAXX binding region) [nucleoplasm]-CandidateSet-hasCandidate R-HSA-9670619 Defective ATRX does not bind DAXX loss_of_function pubmed:28875424|pubmed:14990586|pubmed:21252315 R-HSA-9670615 Defective Inhibition of DNA Recombination at Telomere Due to ATRX Mutations R-HSA-9007926 ATRX binds DAXX Telomere Maintenance R-HSA-157579 723 telomere maintenance frameshift loss_of_function of telomere maintenance

PM1-PM4 ATRX ATRX P995Tfs*5 [nucleoplasm] R-HSA-9672292 ATRX UniProt:P46100 Replacement of residues 995 to 998 by TFRL FragmentReplacedModification COSMIC:COSV64882907 cancer|astrocytoma DOID:162|DOID:3069 R-HSA-9670618-ATRX truncation mutants (DAXX binding region) [nucleoplasm]-CandidateSet-hasCandidate R-HSA-9670619 Defective ATRX does not bind DAXX loss_of_function pubmed:28875424|pubmed:14990586|pubmed:21252315 R-HSA-9670615 Defective Inhibition of DNA Recombination at Telomere Due to ATRX Mutations R-HSA-9007926 ATRX binds DAXX Telomere Maintenance R-HSA-157579 723 telomere maintenance frameshift loss_of_function of telomere maintenance

PM1-PM4 ATRX ATRX K994Efs*6 [nucleoplasm] R-HSA-9672294 ATRX UniProt:P46100 Replacement of residues 994 to 998 by ETFRL FragmentReplacedModification COSMIC:COSV64883994 cancer|large intestine cancer DOID:162|DOID:5672 R-HSA-9670618-ATRX truncation mutants (DAXX binding region) [nucleoplasm]-CandidateSet-hasCandidate R-HSA-9670619 Defective ATRX does not bind DAXX loss_of_function pubmed:28875424|pubmed:14990586|pubmed:21252315 R-HSA-9670615 Defective Inhibition of DNA Recombination at Telomere Due to ATRX Mutations R-HSA-9007926 ATRX binds DAXX Telomere Maintenance R-HSA-157579 723 telomere maintenance frameshift loss_of_function of telomere maintenance

PM1-PM4 ATRX ATRX I1049Nfs*4 [nucleoplasm] R-HSA-9672291 ATRX UniProt:P46100 Replacement of residues 1049 to 1051 by NKR FragmentReplacedModification COSMIC:COSV64873241 prostate adenocarcinoma|leiomyosarcoma|cancer|large intestine cancer|astrocytoma DOID:2526|DOID:1967|DOID:162|DOID:5672|DOID:3069 R-HSA-9670618-ATRX truncation mutants (DAXX binding region) [nucleoplasm]-CandidateSet-hasCandidate R-HSA-9670619 Defective ATRX does not bind DAXX loss_of_function pubmed:28875424|pubmed:14990586|pubmed:21252315 R-HSA-9670615 Defective Inhibition of DNA Recombination at Telomere Due to ATRX Mutations R-HSA-9007926 ATRX binds DAXX Telomere Maintenance R-HSA-157579 723 telomere maintenance frameshift loss_of_function of telomere maintenance

PM1-PM4 ATRX ATRX T387Qfs*27 [nucleoplasm] R-HSA-9672573 ATRX UniProt:P46100 Replacement of residues 387 to 412 by QNYVSLRLLSLCWLILRRLILHWKKT FragmentReplacedModification COSMIC:COSV64884811 cancer|astrocytoma DOID:162|DOID:3069 R-HSA-9670618-ATRX truncation mutants (DAXX binding region) [nucleoplasm]-CandidateSet-hasCandidate R-HSA-9670619 Defective ATRX does not bind DAXX loss_of_function pubmed:28875424|pubmed:14990586|pubmed:21252315 R-HSA-9670615 Defective Inhibition of DNA Recombination at Telomere Due to ATRX Mutations R-HSA-9007926 ATRX binds DAXX Telomere Maintenance R-HSA-157579 723 telomere maintenance frameshift loss_of_function of telomere maintenance

PM1-PM4 ATRX ATRX S471Vfs*43 [nucleoplasm] R-HSA-9672574 ATRX UniProt:P46100 Replacement of residues 471 to 512 by VSTCIRMFQQRNKEQIKVPVVNIRNLIEKKNLNMNLPTLLKI FragmentReplacedModification COSMIC:COSV64886023 cancer|astrocytoma DOID:162|DOID:3069 R-HSA-9670618-ATRX truncation mutants (DAXX binding region) [nucleoplasm]-CandidateSet-hasCandidate R-HSA-9670619 Defective ATRX does not bind DAXX loss_of_function pubmed:28875424|pubmed:14990586|pubmed:21252315 R-HSA-9670615 Defective Inhibition of DNA Recombination at Telomere Due to ATRX Mutations R-HSA-9007926 ATRX binds DAXX Telomere Maintenance R-HSA-157579 723 telomere maintenance frameshift loss_of_function of telomere maintenance

PM1-PM4 ATRX ATRX S558Ifs*4 [nucleoplasm] R-HSA-9672575 ATRX UniProt:P46100 Replacement of residues 558 to 560 by IHL FragmentReplacedModification COSMIC:COSV64870734 cancer|skin melanoma DOID:162|DOID:8923 R-HSA-9670618-ATRX truncation mutants (DAXX binding region) [nucleoplasm]-CandidateSet-hasCandidate R-HSA-9670619 Defective ATRX does not bind DAXX loss_of_function pubmed:28875424|pubmed:14990586|pubmed:21252315 R-HSA-9670615 Defective Inhibition of DNA Recombination at Telomere Due to ATRX Mutations R-HSA-9007926 ATRX binds DAXX Telomere Maintenance R-HSA-157579 723 telomere maintenance frameshift loss_of_function of telomere maintenance

PM1-PM4 ATRX ATRX K358Nfs*2 [nucleoplasm] R-HSA-9672569 ATRX UniProt:P46100 Replacement of residues 358 to 358 by N FragmentReplacedModification COSMIC:COSV64872512 cancer|brain glioma|large intestine cancer DOID:162|DOID:0060108|DOID:5672 R-HSA-9670618-ATRX truncation mutants (DAXX binding region) [nucleoplasm]-CandidateSet-hasCandidate R-HSA-9670619 Defective ATRX does not bind DAXX loss_of_function pubmed:28875424|pubmed:14990586|pubmed:21252315 R-HSA-9670615 Defective Inhibition of DNA Recombination at Telomere Due to ATRX Mutations R-HSA-9007926 ATRX binds DAXX Telomere Maintenance R-HSA-157579 723 telomere maintenance frameshift loss_of_function of telomere maintenance

PM1-PM4 ATRX ATRX L639Wfs*10 [nucleoplasm] R-HSA-9672570 ATRX UniProt:P46100 Replacement of residues 639 to 647 by WLKMKFHYF FragmentReplacedModification COSMIC:COSV64883801 cancer|astrocytoma DOID:162|DOID:3069 R-HSA-9670618-ATRX truncation mutants (DAXX binding region) [nucleoplasm]-CandidateSet-hasCandidate R-HSA-9670619 Defective ATRX does not bind DAXX loss_of_function pubmed:28875424|pubmed:14990586|pubmed:21252315 R-HSA-9670615 Defective Inhibition of DNA Recombination at Telomere Due to ATRX Mutations R-HSA-9007926 ATRX binds DAXX Telomere Maintenance R-HSA-157579 723 telomere maintenance frameshift loss_of_function of telomere maintenance

PM1-PM4 ATRX ATRX G551* [nucleoplasm] R-HSA-9671290 ATRX UniProt:P46100 Nonsense mutation at glycine 551 NonsenseMutation COSMIC:COSV64877662 cancer|brain glioma DOID:162|DOID:0060108 R-HSA-9670618-ATRX truncation mutants (DAXX binding region) [nucleoplasm]-CandidateSet-hasCandidate R-HSA-9670619 Defective ATRX does not bind DAXX loss_of_function pubmed:28875424|pubmed:14990586|pubmed:21252315 R-HSA-9670615 Defective Inhibition of DNA Recombination at Telomere Due to ATRX Mutations R-HSA-9007926 ATRX binds DAXX Telomere Maintenance R-HSA-157579 723 telomere maintenance nonsense loss_of_function of telomere maintenance

PM1-PM4 ATRX ATRX G1071* [nucleoplasm] R-HSA-9671291 ATRX UniProt:P46100 Nonsense mutation at glycine 1071 NonsenseMutation COSMIC:COSV64877662 cancer|brain glioma DOID:162|DOID:0060108 R-HSA-9670618-ATRX truncation mutants (DAXX binding region) [nucleoplasm]-CandidateSet-hasCandidate R-HSA-9670619 Defective ATRX does not bind DAXX loss_of_function pubmed:28875424|pubmed:14990586|pubmed:21252315 R-HSA-9670615 Defective Inhibition of DNA Recombination at Telomere Due to ATRX Mutations R-HSA-9007926 ATRX binds DAXX Telomere Maintenance R-HSA-157579 723 telomere maintenance nonsense loss_of_function of telomere maintenance

PM1-PM4 ATRX ATRX K425Rfs*8 [nucleoplasm] R-HSA-9672564 ATRX UniProt:P46100 Replacement of residues 425 to 431 by REKYQRA FragmentReplacedModification COSMIC:COSV64875864 cancer|astrocytoma DOID:162|DOID:3069 R-HSA-9670618-ATRX truncation mutants (DAXX binding region) [nucleoplasm]-CandidateSet-hasCandidate R-HSA-9670619 Defective ATRX does not bind DAXX loss_of_function pubmed:28875424|pubmed:14990586|pubmed:21252315 R-HSA-9670615 Defective Inhibition of DNA Recombination at Telomere Due to ATRX Mutations R-HSA-9007926 ATRX binds DAXX Telomere Maintenance R-HSA-157579 723 telomere maintenance frameshift loss_of_function of telomere maintenance

PM1-PM4 ATRX ATRX E1010Mfs*24 [nucleoplasm] R-HSA-9672305 ATRX UniProt:P46100 Replacement of residues 1010 to 1032 by MNLHLMALKSYLSEKKFVIFLRA FragmentReplacedModification COSMIC:COSV64885160 cancer|astrocytoma DOID:162|DOID:3069 R-HSA-9670618-ATRX truncation mutants (DAXX binding region) [nucleoplasm]-CandidateSet-hasCandidate R-HSA-9670619 Defective ATRX does not bind DAXX loss_of_function pubmed:28875424|pubmed:14990586|pubmed:21252315 R-HSA-9670615 Defective Inhibition of DNA Recombination at Telomere Due to ATRX Mutations R-HSA-9007926 ATRX binds DAXX Telomere Maintenance R-HSA-157579 723 telomere maintenance frameshift loss_of_function of telomere maintenance

PM1-PM4 ATRX ATRX E991Gfs*9 [nucleoplasm] R-HSA-9672306 ATRX UniProt:P46100 Replacement of residues 991 to 998 by GKKETFRL FragmentReplacedModification COSMIC:COSV64876543 cancer|astrocytoma DOID:162|DOID:3069 R-HSA-9670618-ATRX truncation mutants (DAXX binding region) [nucleoplasm]-CandidateSet-hasCandidate R-HSA-9670619 Defective ATRX does not bind DAXX loss_of_function pubmed:28875424|pubmed:14990586|pubmed:21252315 R-HSA-9670615 Defective Inhibition of DNA Recombination at Telomere Due to ATRX Mutations R-HSA-9007926 ATRX binds DAXX Telomere Maintenance R-HSA-157579 723 telomere maintenance frameshift loss_of_function of telomere maintenance

PM1-PM4 ATRX ATRX N428Yfs*5 [nucleoplasm] R-HSA-9672562 ATRX UniProt:P46100 Replacement of residues 428 to 431 by YQRA FragmentReplacedModification COSMIC:COSV64873269 cancer|astrocytoma DOID:162|DOID:3069 R-HSA-9670618-ATRX truncation mutants (DAXX binding region) [nucleoplasm]-CandidateSet-hasCandidate R-HSA-9670619 Defective ATRX does not bind DAXX loss_of_function pubmed:28875424|pubmed:14990586|pubmed:21252315 R-HSA-9670615 Defective Inhibition of DNA Recombination at Telomere Due to ATRX Mutations R-HSA-9007926 ATRX binds DAXX Telomere Maintenance R-HSA-157579 723 telomere maintenance frameshift loss_of_function of telomere maintenance

PM1-PM4 ATRX ATRX I360Rfs*6 [nucleoplasm] R-HSA-9672563 ATRX UniProt:P46100 Replacement of residues 360 to 364 by RPQPT FragmentReplacedModification COSMIC:COSV64874552 cancer|astrocytoma DOID:162|DOID:3069 R-HSA-9670618-ATRX truncation mutants (DAXX binding region) [nucleoplasm]-CandidateSet-hasCandidate R-HSA-9670619 Defective ATRX does not bind DAXX loss_of_function pubmed:28875424|pubmed:14990586|pubmed:21252315 R-HSA-9670615 Defective Inhibition of DNA Recombination at Telomere Due to ATRX Mutations R-HSA-9007926 ATRX binds DAXX Telomere Maintenance R-HSA-157579 723 telomere maintenance frameshift loss_of_function of telomere maintenance

PM1-PM4 ATRX ATRX S1101* [nucleoplasm] R-HSA-9671245 ATRX UniProt:P46100 Nonsense mutation at L-serine 1101 NonsenseMutation COSMIC:COSV64886124 cancer|astrocytoma DOID:162|DOID:3069 R-HSA-9670618-ATRX truncation mutants (DAXX binding region) [nucleoplasm]-CandidateSet-hasCandidate R-HSA-9670619 Defective ATRX does not bind DAXX loss_of_function pubmed:28875424|pubmed:14990586|pubmed:21252315 R-HSA-9670615 Defective Inhibition of DNA Recombination at Telomere Due to ATRX Mutations R-HSA-9007926 ATRX binds DAXX Telomere Maintenance R-HSA-157579 723 telomere maintenance nonsense loss_of_function of telomere maintenance

PM1-PM4 ATRX ATRX K1018Rfs*5 [nucleoplasm] R-HSA-9672270 ATRX UniProt:P46100 Replacement of residues 1018 to 1021 by RKVT FragmentReplacedModification COSMIC:COSV64875644 cancer|astrocytoma DOID:162|DOID:3069 R-HSA-9670618-ATRX truncation mutants (DAXX binding region) [nucleoplasm]-CandidateSet-hasCandidate R-HSA-9670619 Defective ATRX does not bind DAXX loss_of_function pubmed:28875424|pubmed:14990586|pubmed:21252315 R-HSA-9670615 Defective Inhibition of DNA Recombination at Telomere Due to ATRX Mutations R-HSA-9007926 ATRX binds DAXX Telomere Maintenance R-HSA-157579 723 telomere maintenance frameshift loss_of_function of telomere maintenance

PM1-PM4 ATRX ATRX S729* [nucleoplasm] R-HSA-9671246 ATRX UniProt:P46100 Nonsense mutation at L-serine 729 NonsenseMutation COSMIC:COSV64877344 cancer|large intestine cancer DOID:162|DOID:5672 R-HSA-9670618-ATRX truncation mutants (DAXX binding region) [nucleoplasm]-CandidateSet-hasCandidate R-HSA-9670619 Defective ATRX does not bind DAXX loss_of_function pubmed:28875424|pubmed:14990586|pubmed:21252315 R-HSA-9670615 Defective Inhibition of DNA Recombination at Telomere Due to ATRX Mutations R-HSA-9007926 ATRX binds DAXX Telomere Maintenance R-HSA-157579 723 telomere maintenance nonsense loss_of_function of telomere maintenance

PM1-PM4 ATRX ATRX V957Sfs*7 [nucleoplasm] R-HSA-9672271 ATRX UniProt:P46100 Replacement of residues 957 to 962 by STGWLI FragmentReplacedModification COSMIC:COSV64875290 cancer|large intestine cancer DOID:162|DOID:5672 R-HSA-9670618-ATRX truncation mutants (DAXX binding region) [nucleoplasm]-CandidateSet-hasCandidate R-HSA-9670619 Defective ATRX does not bind DAXX loss_of_function pubmed:28875424|pubmed:14990586|pubmed:21252315 R-HSA-9670615 Defective Inhibition of DNA Recombination at Telomere Due to ATRX Mutations R-HSA-9007926 ATRX binds DAXX Telomere Maintenance R-HSA-157579 723 telomere maintenance frameshift loss_of_function of telomere maintenance

PM1-PM4 ATRX ATRX S567* [nucleoplasm] R-HSA-9671243 ATRX UniProt:P46100 Nonsense mutation at L-serine 567 NonsenseMutation COSMIC:COSV64882307 cancer|lung small cell carcinoma DOID:162|DOID:5409 R-HSA-9670618-ATRX truncation mutants (DAXX binding region) [nucleoplasm]-CandidateSet-hasCandidate R-HSA-9670619 Defective ATRX does not bind DAXX loss_of_function pubmed:28875424|pubmed:14990586|pubmed:21252315 R-HSA-9670615 Defective Inhibition of DNA Recombination at Telomere Due to ATRX Mutations R-HSA-9007926 ATRX binds DAXX Telomere Maintenance R-HSA-157579 723 telomere maintenance nonsense loss_of_function of telomere maintenance

PM1-PM4 ATRX ATRX R885Sfs*21 [nucleoplasm] R-HSA-9672284 ATRX UniProt:P46100 Replacement of residues 885 to 904 by SKRLSLQQKAQLIKTRPSWN FragmentReplacedModification COSMIC:COSV64880784 cancer|astrocytoma DOID:162|DOID:3069 R-HSA-9670618-ATRX truncation mutants (DAXX binding region) [nucleoplasm]-CandidateSet-hasCandidate R-HSA-9670619 Defective ATRX does not bind DAXX loss_of_function pubmed:28875424|pubmed:14990586|pubmed:21252315 R-HSA-9670615 Defective Inhibition of DNA Recombination at Telomere Due to ATRX Mutations R-HSA-9007926 ATRX binds DAXX Telomere Maintenance R-HSA-157579 723 telomere maintenance frameshift loss_of_function of telomere maintenance

PM1-PM4 ATRX ATRX S797* [nucleoplasm] R-HSA-9671260 ATRX UniProt:P46100 Nonsense mutation at L-serine 797 NonsenseMutation COSMIC:COSV64870525 cancer|astrocytoma DOID:162|DOID:3069 R-HSA-9670618-ATRX truncation mutants (DAXX binding region) [nucleoplasm]-CandidateSet-hasCandidate R-HSA-9670619 Defective ATRX does not bind DAXX loss_of_function pubmed:28875424|pubmed:14990586|pubmed:21252315 R-HSA-9670615 Defective Inhibition of DNA Recombination at Telomere Due to ATRX Mutations R-HSA-9007926 ATRX binds DAXX Telomere Maintenance R-HSA-157579 723 telomere maintenance nonsense loss_of_function of telomere maintenance

PM1-PM4 ATRX ATRX T844Yfs*5 [nucleoplasm] R-HSA-9672286 ATRX UniProt:P46100 Replacement of residues 844 to 847 by YKRF FragmentReplacedModification COSMIC:COSV64880897 cancer|islet cell tumor DOID:162|DOID:1799 R-HSA-9670618-ATRX truncation mutants (DAXX binding region) [nucleoplasm]-CandidateSet-hasCandidate R-HSA-9670619 Defective ATRX does not bind DAXX loss_of_function pubmed:28875424|pubmed:14990586|pubmed:21252315 R-HSA-9670615 Defective Inhibition of DNA Recombination at Telomere Due to ATRX Mutations R-HSA-9007926 ATRX binds DAXX Telomere Maintenance R-HSA-157579 723 telomere maintenance frameshift loss_of_function of telomere maintenance

PM1-PM4 ATRX ATRX S788* [nucleoplasm] R-HSA-9671257 ATRX UniProt:P46100 Nonsense mutation at L-serine 788 NonsenseMutation COSMIC:COSV64874366 cancer|astrocytoma DOID:162|DOID:3069 R-HSA-9670618-ATRX truncation mutants (DAXX binding region) [nucleoplasm]-CandidateSet-hasCandidate R-HSA-9670619 Defective ATRX does not bind DAXX loss_of_function pubmed:28875424|pubmed:14990586|pubmed:21252315 R-HSA-9670615 Defective Inhibition of DNA Recombination at Telomere Due to ATRX Mutations R-HSA-9007926 ATRX binds DAXX Telomere Maintenance R-HSA-157579 723 telomere maintenance nonsense loss_of_function of telomere maintenance

PM1-PM4 ATRX ATRX R840Kfs*9 [nucleoplasm] R-HSA-9672276 ATRX UniProt:P46100 Replacement of residues 840 to 847 by KNSKYKRF FragmentReplacedModification COSMIC:COSV64870834 leiomyosarcoma|stomach cancer|cancer|medulloblastoma|large intestine cancer|astrocytoma DOID:1967|DOID:10534|DOID:162|DOID:0050902|DOID:5672|DOID:3069 R-HSA-9670618-ATRX truncation mutants (DAXX binding region) [nucleoplasm]-CandidateSet-hasCandidate R-HSA-9670619 Defective ATRX does not bind DAXX loss_of_function pubmed:28875424|pubmed:14990586|pubmed:21252315 R-HSA-9670615 Defective Inhibition of DNA Recombination at Telomere Due to ATRX Mutations R-HSA-9007926 ATRX binds DAXX Telomere Maintenance R-HSA-157579 723 telomere maintenance frameshift loss_of_function of telomere maintenance

PM1-PM4 ATRX ATRX S750* [nucleoplasm] R-HSA-9671254 ATRX UniProt:P46100 Nonsense mutation at L-serine 750 NonsenseMutation COSMIC:COSV64879736 cancer|breast cancer DOID:162|DOID:1612 R-HSA-9670618-ATRX truncation mutants (DAXX binding region) [nucleoplasm]-CandidateSet-hasCandidate R-HSA-9670619 Defective ATRX does not bind DAXX loss_of_function pubmed:28875424|pubmed:14990586|pubmed:21252315 R-HSA-9670615 Defective Inhibition of DNA Recombination at Telomere Due to ATRX Mutations R-HSA-9007926 ATRX binds DAXX Telomere Maintenance R-HSA-157579 723 telomere maintenance nonsense loss_of_function of telomere maintenance

PM1-PM4 ATRX ATRX S342* [nucleoplasm] R-HSA-9671250 ATRX UniProt:P46100 Nonsense mutation at L-serine 342 NonsenseMutation COSMIC:COSV64876814 cancer DOID:162 R-HSA-9670618-ATRX truncation mutants (DAXX binding region) [nucleoplasm]-CandidateSet-hasCandidate R-HSA-9670619 Defective ATRX does not bind DAXX loss_of_function pubmed:28875424|pubmed:14990586|pubmed:21252315 R-HSA-9670615 Defective Inhibition of DNA Recombination at Telomere Due to ATRX Mutations R-HSA-9007926 ATRX binds DAXX Telomere Maintenance R-HSA-157579 723 telomere maintenance nonsense loss_of_function of telomere maintenance

PM1-PM4 ATRX ATRX C1122Lfs*8 [nucleoplasm] R-HSA-9672748 ATRX UniProt:P46100 Replacement of residues 1122 to 1128 by LTLLIRD FragmentReplacedModification COSMIC:COSV64871832 cancer|skin melanoma DOID:162|DOID:8923 R-HSA-9670618-ATRX truncation mutants (DAXX binding region) [nucleoplasm]-CandidateSet-hasCandidate R-HSA-9670619 Defective ATRX does not bind DAXX loss_of_function pubmed:28875424|pubmed:14990586|pubmed:21252315 R-HSA-9670615 Defective Inhibition of DNA Recombination at Telomere Due to ATRX Mutations R-HSA-9007926 ATRX binds DAXX Telomere Maintenance R-HSA-157579 723 telomere maintenance frameshift loss_of_function of telomere maintenance

PM1-PM4 ATRX ATRX E431* [nucleoplasm] R-HSA-9670957 ATRX UniProt:P46100 Nonsense mutation at L-glutamic acid 431 NonsenseMutation COSMIC:COSV64879070 lung adenocarcinoma|cancer DOID:3910|DOID:162 R-HSA-9670618-ATRX truncation mutants (DAXX binding region) [nucleoplasm]-CandidateSet-hasCandidate R-HSA-9670619 Defective ATRX does not bind DAXX loss_of_function pubmed:28875424|pubmed:14990586|pubmed:21252315 R-HSA-9670615 Defective Inhibition of DNA Recombination at Telomere Due to ATRX Mutations R-HSA-9007926 ATRX binds DAXX Telomere Maintenance R-HSA-157579 723 telomere maintenance nonsense loss_of_function of telomere maintenance

PM1-PM4 ATRX ATRX E482* [nucleoplasm] R-HSA-9670959 ATRX UniProt:P46100 Nonsense mutation at L-glutamic acid 482 NonsenseMutation COSMIC:COSV64887171 cancer|adrenal cortical adenoma DOID:162|DOID:0050891 R-HSA-9670618-ATRX truncation mutants (DAXX binding region) [nucleoplasm]-CandidateSet-hasCandidate R-HSA-9670619 Defective ATRX does not bind DAXX loss_of_function pubmed:28875424|pubmed:14990586|pubmed:21252315 R-HSA-9670615 Defective Inhibition of DNA Recombination at Telomere Due to ATRX Mutations R-HSA-9007926 ATRX binds DAXX Telomere Maintenance R-HSA-157579 723 telomere maintenance nonsense loss_of_function of telomere maintenance

PM1-PM4 ATRX ATRX E643* [nucleoplasm] R-HSA-9670952 ATRX UniProt:P46100 Nonsense mutation at L-glutamic acid 643 NonsenseMutation COSMIC:COSV64877074 cancer DOID:162 R-HSA-9670618-ATRX truncation mutants (DAXX binding region) [nucleoplasm]-CandidateSet-hasCandidate R-HSA-9670619 Defective ATRX does not bind DAXX loss_of_function pubmed:28875424|pubmed:14990586|pubmed:21252315 R-HSA-9670615 Defective Inhibition of DNA Recombination at Telomere Due to ATRX Mutations R-HSA-9007926 ATRX binds DAXX Telomere Maintenance R-HSA-157579 723 telomere maintenance nonsense loss_of_function of telomere maintenance

PM1-PM4 ATRX ATRX K1081Rfs*37 [nucleoplasm] R-HSA-9672745 ATRX UniProt:P46100 Replacement of residues 1081 to 1116 by RVRMEHMVERRKGASCLERVQGRDKIVHHLILRNIP FragmentReplacedModification COSMIC:COSV64872241 cancer|astrocytoma DOID:162|DOID:3069 R-HSA-9670618-ATRX truncation mutants (DAXX binding region) [nucleoplasm]-CandidateSet-hasCandidate R-HSA-9670619 Defective ATRX does not bind DAXX loss_of_function pubmed:28875424|pubmed:14990586|pubmed:21252315 R-HSA-9670615 Defective Inhibition of DNA Recombination at Telomere Due to ATRX Mutations R-HSA-9007926 ATRX binds DAXX Telomere Maintenance R-HSA-157579 723 telomere maintenance frameshift loss_of_function of telomere maintenance

PM1-PM4 ATRX ATRX E1065* [nucleoplasm] R-HSA-9670953 ATRX UniProt:P46100 Nonsense mutation at L-glutamic acid 1065 NonsenseMutation COSMIC:COSV64875064 vulva squamous cell carcinoma|cancer|astrocytoma DOID:2101|DOID:162|DOID:3069 R-HSA-9670618-ATRX truncation mutants (DAXX binding region) [nucleoplasm]-CandidateSet-hasCandidate R-HSA-9670619 Defective ATRX does not bind DAXX loss_of_function pubmed:28875424|pubmed:14990586|pubmed:21252315 R-HSA-9670615 Defective Inhibition of DNA Recombination at Telomere Due to ATRX Mutations R-HSA-9007926 ATRX binds DAXX Telomere Maintenance R-HSA-157579 723 telomere maintenance nonsense loss_of_function of telomere maintenance

PM1-PM4 ATRX ATRX E1159* [nucleoplasm] R-HSA-9670954 ATRX UniProt:P46100 Nonsense mutation at L-glutamic acid 1159 NonsenseMutation COSMIC:COSV64884222 lung adenocarcinoma|cancer DOID:3910|DOID:162 R-HSA-9670618-ATRX truncation mutants (DAXX binding region) [nucleoplasm]-CandidateSet-hasCandidate R-HSA-9670619 Defective ATRX does not bind DAXX loss_of_function pubmed:28875424|pubmed:14990586|pubmed:21252315 R-HSA-9670615 Defective Inhibition of DNA Recombination at Telomere Due to ATRX Mutations R-HSA-9007926 ATRX binds DAXX Telomere Maintenance R-HSA-157579 723 telomere maintenance nonsense loss_of_function of telomere maintenance

PM1-PM4 ATRX ATRX D1106Ifs*12 [nucleoplasm] R-HSA-9672747 ATRX UniProt:P46100 Replacement of residues 1106 to 1116 by IVHHLILRNIP FragmentReplacedModification COSMIC:COSV64883066 brain oligodendroglioma|cancer DOID:3187|DOID:162 R-HSA-9670618-ATRX truncation mutants (DAXX binding region) [nucleoplasm]-CandidateSet-hasCandidate R-HSA-9670619 Defective ATRX does not bind DAXX loss_of_function pubmed:28875424|pubmed:14990586|pubmed:21252315 R-HSA-9670615 Defective Inhibition of DNA Recombination at Telomere Due to ATRX Mutations R-HSA-9007926 ATRX binds DAXX Telomere Maintenance R-HSA-157579 723 telomere maintenance frameshift loss_of_function of telomere maintenance

PM1-PM4 ATRX ATRX E585* [nucleoplasm] R-HSA-9670948 ATRX UniProt:P46100 Nonsense mutation at L-glutamic acid 585 NonsenseMutation COSMIC:COSV64883151 endometrial cancer|cancer DOID:1380|DOID:162 R-HSA-9670618-ATRX truncation mutants (DAXX binding region) [nucleoplasm]-CandidateSet-hasCandidate R-HSA-9670619 Defective ATRX does not bind DAXX loss_of_function pubmed:28875424|pubmed:14990586|pubmed:21252315 R-HSA-9670615 Defective Inhibition of DNA Recombination at Telomere Due to ATRX Mutations R-HSA-9007926 ATRX binds DAXX Telomere Maintenance R-HSA-157579 723 telomere maintenance nonsense loss_of_function of telomere maintenance

PM1-PM4 ATRX ATRX K1057Rfs*61 [nucleoplasm] R-HSA-9672741 ATRX UniProt:P46100 Replacement of residues 1057 to 1116 by RMNYLIMLRSQQGKEIVVTLQRIKRVRMEHMVERRKGASCLERVQGRDKIVHHLILRNIP FragmentReplacedModification COSMIC:COSV64873532 cancer|astrocytoma DOID:162|DOID:3069 R-HSA-9670618-ATRX truncation mutants (DAXX binding region) [nucleoplasm]-CandidateSet-hasCandidate R-HSA-9670619 Defective ATRX does not bind DAXX loss_of_function pubmed:28875424|pubmed:14990586|pubmed:21252315 R-HSA-9670615 Defective Inhibition of DNA Recombination at Telomere Due to ATRX Mutations R-HSA-9007926 ATRX binds DAXX Telomere Maintenance R-HSA-157579 723 telomere maintenance frameshift loss_of_function of telomere maintenance

PM1-PM4 ATRX ATRX E533* [nucleoplasm] R-HSA-9670949 ATRX UniProt:P46100 Nonsense mutation at L-glutamic acid 533 NonsenseMutation COSMIC:COSV64877534 cancer|astrocytoma DOID:162|DOID:3069 R-HSA-9670618-ATRX truncation mutants (DAXX binding region) [nucleoplasm]-CandidateSet-hasCandidate R-HSA-9670619 Defective ATRX does not bind DAXX loss_of_function pubmed:28875424|pubmed:14990586|pubmed:21252315 R-HSA-9670615 Defective Inhibition of DNA Recombination at Telomere Due to ATRX Mutations R-HSA-9007926 ATRX binds DAXX Telomere Maintenance R-HSA-157579 723 telomere maintenance nonsense loss_of_function of telomere maintenance

PM1-PM4 ATRX ATRX E680* [nucleoplasm] R-HSA-9670950 ATRX UniProt:P46100 Nonsense mutation at L-glutamic acid 680 NonsenseMutation COSMIC:COSV64885777 endometrial cancer|cancer DOID:1380|DOID:162 R-HSA-9670618-ATRX truncation mutants (DAXX binding region) [nucleoplasm]-CandidateSet-hasCandidate R-HSA-9670619 Defective ATRX does not bind DAXX loss_of_function pubmed:28875424|pubmed:14990586|pubmed:21252315 R-HSA-9670615 Defective Inhibition of DNA Recombination at Telomere Due to ATRX Mutations R-HSA-9007926 ATRX binds DAXX Telomere Maintenance R-HSA-157579 723 telomere maintenance nonsense loss_of_function of telomere maintenance

PM1-PM4 ATRX ATRX D1126Rfs*3 [nucleoplasm] R-HSA-9672738 ATRX UniProt:P46100 Replacement of residues 1126 to 1127 by RD FragmentReplacedModification COSMIC:COSV64873638 endometrial cancer|cancer DOID:1380|DOID:162 R-HSA-9670618-ATRX truncation mutants (DAXX binding region) [nucleoplasm]-CandidateSet-hasCandidate R-HSA-9670619 Defective ATRX does not bind DAXX loss_of_function pubmed:28875424|pubmed:14990586|pubmed:21252315 R-HSA-9670615 Defective Inhibition of DNA Recombination at Telomere Due to ATRX Mutations R-HSA-9007926 ATRX binds DAXX Telomere Maintenance R-HSA-157579 723 telomere maintenance frameshift loss_of_function of telomere maintenance

PM1-PM4 ATRX ATRX K1143Rfs*47 [nucleoplasm] R-HSA-9672739 ATRX UniProt:P46100 Replacement of residues 1143 to 1188 by REILRKYKVAHHHLMLRKVLKIIKRRSKELHLKRRQSLSRRKRETP FragmentReplacedModification COSMIC:COSV64879887 cancer|astrocytoma DOID:162|DOID:3069 R-HSA-9670618-ATRX truncation mutants (DAXX binding region) [nucleoplasm]-CandidateSet-hasCandidate R-HSA-9670619 Defective ATRX does not bind DAXX loss_of_function pubmed:28875424|pubmed:14990586|pubmed:21252315 R-HSA-9670615 Defective Inhibition of DNA Recombination at Telomere Due to ATRX Mutations R-HSA-9007926 ATRX binds DAXX Telomere Maintenance R-HSA-157579 723 telomere maintenance frameshift loss_of_function of telomere maintenance

PM1-PM4 ATRX ATRX D791* [nucleoplasm] R-HSA-9672254 ATRX UniProt:P46100 Nonsense mutation at L-aspartic acid 791 NonsenseMutation COSMIC:COSV64884269 cancer|fibrosarcoma DOID:162|DOID:3355 R-HSA-9670618-ATRX truncation mutants (DAXX binding region) [nucleoplasm]-CandidateSet-hasCandidate R-HSA-9670619 Defective ATRX does not bind DAXX loss_of_function pubmed:28875424|pubmed:14990586|pubmed:21252315 R-HSA-9670615 Defective Inhibition of DNA Recombination at Telomere Due to ATRX Mutations R-HSA-9007926 ATRX binds DAXX Telomere Maintenance R-HSA-157579 723 telomere maintenance nonsense loss_of_function of telomere maintenance

PM1-PM4 ATRX ATRX E625* [nucleoplasm] R-HSA-9670964 ATRX UniProt:P46100 Nonsense mutation at L-glutamic acid 625 NonsenseMutation COSMIC:COSV64870633 adrenocortical carcinoma|cancer DOID:3948|DOID:162 R-HSA-9670618-ATRX truncation mutants (DAXX binding region) [nucleoplasm]-CandidateSet-hasCandidate R-HSA-9670619 Defective ATRX does not bind DAXX loss_of_function pubmed:28875424|pubmed:14990586|pubmed:21252315 R-HSA-9670615 Defective Inhibition of DNA Recombination at Telomere Due to ATRX Mutations R-HSA-9007926 ATRX binds DAXX Telomere Maintenance R-HSA-157579 723 telomere maintenance nonsense loss_of_function of telomere maintenance

PM1-PM4 ATRX ATRX L359Tfs*3 [nucleoplasm] R-HSA-9672244 ATRX UniProt:P46100 Replacement of residues 359 to 360 by TD FragmentReplacedModification COSMIC:COSV64871091 cancer|breast cancer|large intestine cancer|astrocytoma DOID:162|DOID:1612|DOID:5672|DOID:3069 R-HSA-9670618-ATRX truncation mutants (DAXX binding region) [nucleoplasm]-CandidateSet-hasCandidate R-HSA-9670619 Defective ATRX does not bind DAXX loss_of_function pubmed:28875424|pubmed:14990586|pubmed:21252315 R-HSA-9670615 Defective Inhibition of DNA Recombination at Telomere Due to ATRX Mutations R-HSA-9007926 ATRX binds DAXX Telomere Maintenance R-HSA-157579 723 telomere maintenance frameshift loss_of_function of telomere maintenance

PM1-PM4 ATRX ATRX P663Yfs*10 [nucleoplasm] R-HSA-9672247 ATRX UniProt:P46100 Replacement of residues 663 to 671 by YTLEATDRN FragmentReplacedModification COSMIC:COSV64870763 neuroblastoma|cancer DOID:769|DOID:162 R-HSA-9670618-ATRX truncation mutants (DAXX binding region) [nucleoplasm]-CandidateSet-hasCandidate R-HSA-9670619 Defective ATRX does not bind DAXX loss_of_function pubmed:28875424|pubmed:14990586|pubmed:21252315 R-HSA-9670615 Defective Inhibition of DNA Recombination at Telomere Due to ATRX Mutations R-HSA-9007926 ATRX binds DAXX Telomere Maintenance R-HSA-157579 723 telomere maintenance frameshift loss_of_function of telomere maintenance

PM1-PM4 ATRX ATRX E1119* [nucleoplasm] R-HSA-9670960 ATRX UniProt:P46100 Nonsense mutation at L-glutamic acid 1119 NonsenseMutation COSMIC:COSV64883029 endometrial cancer|cancer DOID:1380|DOID:162 R-HSA-9670618-ATRX truncation mutants (DAXX binding region) [nucleoplasm]-CandidateSet-hasCandidate R-HSA-9670619 Defective ATRX does not bind DAXX loss_of_function pubmed:28875424|pubmed:14990586|pubmed:21252315 R-HSA-9670615 Defective Inhibition of DNA Recombination at Telomere Due to ATRX Mutations R-HSA-9007926 ATRX binds DAXX Telomere Maintenance R-HSA-157579 723 telomere maintenance nonsense loss_of_function of telomere maintenance

PM1-PM4 ATRX ATRX R1128Sfs*2 [nucleoplasm] R-HSA-9672753 ATRX UniProt:P46100 Replacement of residues 1128 to 1128 by S FragmentReplacedModification cancer|brain stem glioma DOID:162|DOID:4202 R-HSA-9670618-ATRX truncation mutants (DAXX binding region) [nucleoplasm]-CandidateSet-hasCandidate R-HSA-9670619 Defective ATRX does not bind DAXX loss_of_function pubmed:28875424|pubmed:14990586|pubmed:21252315 R-HSA-9670615 Defective Inhibition of DNA Recombination at Telomere Due to ATRX Mutations R-HSA-9007926 ATRX binds DAXX Telomere Maintenance R-HSA-157579 723 telomere maintenance frameshift loss_of_function of telomere maintenance

PM1-PM4 ATRX ATRX L359Hfs*3 [nucleoplasm] R-HSA-9672241 ATRX UniProt:P46100 Replacement of residues 359 to 360 by HD FragmentReplacedModification COSMIC:COSV64873017 cancer|brain glioma|astrocytoma DOID:162|DOID:0060108|DOID:3069 R-HSA-9670618-ATRX truncation mutants (DAXX binding region) [nucleoplasm]-CandidateSet-hasCandidate R-HSA-9670619 Defective ATRX does not bind DAXX loss_of_function pubmed:28875424|pubmed:14990586|pubmed:21252315 R-HSA-9670615 Defective Inhibition of DNA Recombination at Telomere Due to ATRX Mutations R-HSA-9007926 ATRX binds DAXX Telomere Maintenance R-HSA-157579 723 telomere maintenance frameshift loss_of_function of telomere maintenance

PM1-PM4 ATRX ATRX L452Ffs*12 [nucleoplasm] R-HSA-9672242 ATRX UniProt:P46100 Replacement of residues 452 to 462 by FGKEGYFKVRS FragmentReplacedModification COSMIC:COSV64873147 cancer|brain glioma DOID:162|DOID:0060108 R-HSA-9670618-ATRX truncation mutants (DAXX binding region) [nucleoplasm]-CandidateSet-hasCandidate R-HSA-9670619 Defective ATRX does not bind DAXX loss_of_function pubmed:28875424|pubmed:14990586|pubmed:21252315 R-HSA-9670615 Defective Inhibition of DNA Recombination at Telomere Due to ATRX Mutations R-HSA-9007926 ATRX binds DAXX Telomere Maintenance R-HSA-157579 723 telomere maintenance frameshift loss_of_function of telomere maintenance

PM1-PM4 ATRX ATRX I383Nfs*11 [nucleoplasm] R-HSA-9672243 ATRX UniProt:P46100 Replacement of residues 383 to 392 by NQFCYKITSA FragmentReplacedModification COSMIC:COSV64876182 cancer|astrocytoma DOID:162|DOID:3069 R-HSA-9670618-ATRX truncation mutants (DAXX binding region) [nucleoplasm]-CandidateSet-hasCandidate R-HSA-9670619 Defective ATRX does not bind DAXX loss_of_function pubmed:28875424|pubmed:14990586|pubmed:21252315 R-HSA-9670615 Defective Inhibition of DNA Recombination at Telomere Due to ATRX Mutations R-HSA-9007926 ATRX binds DAXX Telomere Maintenance R-HSA-157579 723 telomere maintenance frameshift loss_of_function of telomere maintenance

PM1-PM4 ATRX ATRX L664* [nucleoplasm] R-HSA-9671689 ATRX UniProt:P46100 Nonsense mutation at L-leucine 664 NonsenseMutation COSMIC:COSV64879362 cancer|astrocytoma DOID:162|DOID:3069 R-HSA-9670618-ATRX truncation mutants (DAXX binding region) [nucleoplasm]-CandidateSet-hasCandidate R-HSA-9670619 Defective ATRX does not bind DAXX loss_of_function pubmed:28875424|pubmed:14990586|pubmed:21252315 R-HSA-9670615 Defective Inhibition of DNA Recombination at Telomere Due to ATRX Mutations R-HSA-9007926 ATRX binds DAXX Telomere Maintenance R-HSA-157579 723 telomere maintenance nonsense loss_of_function of telomere maintenance

PM1-PM4 ATRX ATRX L513* [nucleoplasm] R-HSA-9671690 ATRX UniProt:P46100 Nonsense mutation at L-leucine 513 NonsenseMutation COSMIC:COSV64887073 cancer|astrocytoma DOID:162|DOID:3069 R-HSA-9670618-ATRX truncation mutants (DAXX binding region) [nucleoplasm]-CandidateSet-hasCandidate R-HSA-9670619 Defective ATRX does not bind DAXX loss_of_function pubmed:28875424|pubmed:14990586|pubmed:21252315 R-HSA-9670615 Defective Inhibition of DNA Recombination at Telomere Due to ATRX Mutations R-HSA-9007926 ATRX binds DAXX Telomere Maintenance R-HSA-157579 723 telomere maintenance nonsense loss_of_function of telomere maintenance

PM1-PM4 ATRX ATRX I1049* [nucleoplasm] R-HSA-9672715 ATRX UniProt:P46100 Nonsense mutation at L-isoleucine 1049 NonsenseMutation COSMIC:COSV64879254 cancer|osteosarcoma DOID:162|DOID:3347 R-HSA-9670618-ATRX truncation mutants (DAXX binding region) [nucleoplasm]-CandidateSet-hasCandidate R-HSA-9670619 Defective ATRX does not bind DAXX loss_of_function pubmed:28875424|pubmed:14990586|pubmed:21252315 R-HSA-9670615 Defective Inhibition of DNA Recombination at Telomere Due to ATRX Mutations R-HSA-9007926 ATRX binds DAXX Telomere Maintenance R-HSA-157579 723 telomere maintenance nonsense loss_of_function of telomere maintenance

PM1-PM4 ATRX ATRX E976Dfs*2 [nucleoplasm] R-HSA-9672708 ATRX UniProt:P46100 Replacement of residues 976 to 976 by D FragmentReplacedModification COSMIC:COSV64879877 bladder adenocarcinoma|cancer DOID:3711|DOID:162 R-HSA-9670618-ATRX truncation mutants (DAXX binding region) [nucleoplasm]-CandidateSet-hasCandidate R-HSA-9670619 Defective ATRX does not bind DAXX loss_of_function pubmed:28875424|pubmed:14990586|pubmed:21252315 R-HSA-9670615 Defective Inhibition of DNA Recombination at Telomere Due to ATRX Mutations R-HSA-9007926 ATRX binds DAXX Telomere Maintenance R-HSA-157579 723 telomere maintenance frameshift loss_of_function of telomere maintenance

PM1-PM4 ATRX ATRX T684Sfs*2 [nucleoplasm] R-HSA-9672605 ATRX UniProt:P46100 Replacement of residues 684 to 684 by S FragmentReplacedModification COSMIC:COSV64873251 cancer|astrocytoma DOID:162|DOID:3069 R-HSA-9670618-ATRX truncation mutants (DAXX binding region) [nucleoplasm]-CandidateSet-hasCandidate R-HSA-9670619 Defective ATRX does not bind DAXX loss_of_function pubmed:28875424|pubmed:14990586|pubmed:21252315 R-HSA-9670615 Defective Inhibition of DNA Recombination at Telomere Due to ATRX Mutations R-HSA-9007926 ATRX binds DAXX Telomere Maintenance R-HSA-157579 723 telomere maintenance frameshift loss_of_function of telomere maintenance

PM1-PM4 ATRX ATRX L738Gfs*13 [nucleoplasm] R-HSA-9672604 ATRX UniProt:P46100 Replacement of residues 738 to 749 by GEQDESQFFFRY FragmentReplacedModification COSMIC:COSV64878768 leiomyosarcoma|cancer DOID:1967|DOID:162 R-HSA-9670618-ATRX truncation mutants (DAXX binding region) [nucleoplasm]-CandidateSet-hasCandidate R-HSA-9670619 Defective ATRX does not bind DAXX loss_of_function pubmed:28875424|pubmed:14990586|pubmed:21252315 R-HSA-9670615 Defective Inhibition of DNA Recombination at Telomere Due to ATRX Mutations R-HSA-9007926 ATRX binds DAXX Telomere Maintenance R-HSA-157579 723 telomere maintenance frameshift loss_of_function of telomere maintenance

PM1-PM4 ATRX ATRX Q554Rfs*21 [nucleoplasm] R-HSA-9672582 ATRX UniProt:P46100 Replacement of residues 554 to 573 by RSGEFICKIKYFFKRQQRRY FragmentReplacedModification COSMIC:COSV64870513 cancer|astrocytoma DOID:162|DOID:3069 R-HSA-9670618-ATRX truncation mutants (DAXX binding region) [nucleoplasm]-CandidateSet-hasCandidate R-HSA-9670619 Defective ATRX does not bind DAXX loss_of_function pubmed:28875424|pubmed:14990586|pubmed:21252315 R-HSA-9670615 Defective Inhibition of DNA Recombination at Telomere Due to ATRX Mutations R-HSA-9007926 ATRX binds DAXX Telomere Maintenance R-HSA-157579 723 telomere maintenance frameshift loss_of_function of telomere maintenance

PM1-PM4 ATRX ATRX K778Nfs*23 [nucleoplasm] R-HSA-9672625 ATRX UniProt:P46100 Replacement of residues 778 to 799 by NEKVLHLAQILILKRANQLRAL FragmentReplacedModification COSMIC:COSV64871399 cancer|astrocytoma DOID:162|DOID:3069 R-HSA-9670618-ATRX truncation mutants (DAXX binding region) [nucleoplasm]-CandidateSet-hasCandidate R-HSA-9670619 Defective ATRX does not bind DAXX loss_of_function pubmed:28875424|pubmed:14990586|pubmed:21252315 R-HSA-9670615 Defective Inhibition of DNA Recombination at Telomere Due to ATRX Mutations R-HSA-9007926 ATRX binds DAXX Telomere Maintenance R-HSA-157579 723 telomere maintenance frameshift loss_of_function of telomere maintenance

PM1-PM4 ATRX ATRX S747Ffs*6 [nucleoplasm] R-HSA-9672628 ATRX UniProt:P46100 Replacement of residues 747 to 751 by FFFRY FragmentReplacedModification COSMIC:COSV64880470 cancer|astrocytoma DOID:162|DOID:3069 R-HSA-9670618-ATRX truncation mutants (DAXX binding region) [nucleoplasm]-CandidateSet-hasCandidate R-HSA-9670619 Defective ATRX does not bind DAXX loss_of_function pubmed:28875424|pubmed:14990586|pubmed:21252315 R-HSA-9670615 Defective Inhibition of DNA Recombination at Telomere Due to ATRX Mutations R-HSA-9007926 ATRX binds DAXX Telomere Maintenance R-HSA-157579 723 telomere maintenance frameshift loss_of_function of telomere maintenance

PM1-PM4 ATRX ATRX Y341* [nucleoplasm] R-HSA-9671612 ATRX UniProt:P46100 Nonsense mutation at L-tyrosine 341 NonsenseMutation COSMIC:COSV64872234 cancer|astrocytoma DOID:162|DOID:3069 R-HSA-9670618-ATRX truncation mutants (DAXX binding region) [nucleoplasm]-CandidateSet-hasCandidate R-HSA-9670619 Defective ATRX does not bind DAXX loss_of_function pubmed:28875424|pubmed:14990586|pubmed:21252315 R-HSA-9670615 Defective Inhibition of DNA Recombination at Telomere Due to ATRX Mutations R-HSA-9007926 ATRX binds DAXX Telomere Maintenance R-HSA-157579 723 telomere maintenance nonsense loss_of_function of telomere maintenance

PM1-PM4 ATRX ATRX Q727* [nucleoplasm] R-HSA-9671586 ATRX UniProt:P46100 Nonsense mutation at L-glutamine 727 NonsenseMutation COSMIC:COSV64876208 leiomyosarcoma|cancer DOID:1967|DOID:162 R-HSA-9670618-ATRX truncation mutants (DAXX binding region) [nucleoplasm]-CandidateSet-hasCandidate R-HSA-9670619 Defective ATRX does not bind DAXX loss_of_function pubmed:28875424|pubmed:14990586|pubmed:21252315 R-HSA-9670615 Defective Inhibition of DNA Recombination at Telomere Due to ATRX Mutations R-HSA-9007926 ATRX binds DAXX Telomere Maintenance R-HSA-157579 723 telomere maintenance nonsense loss_of_function of telomere maintenance

PM1-PM4 ATRX ATRX D774Mfs*29 [nucleoplasm] R-HSA-9672608 ATRX UniProt:P46100 Replacement of residues 774 to 801 by MIKEKGNEKVLHLAQILILKRANQLRAL FragmentReplacedModification COSMIC:COSV64878434 lung adenocarcinoma|cancer DOID:3910|DOID:162 R-HSA-9670618-ATRX truncation mutants (DAXX binding region) [nucleoplasm]-CandidateSet-hasCandidate R-HSA-9670619 Defective ATRX does not bind DAXX loss_of_function pubmed:28875424|pubmed:14990586|pubmed:21252315 R-HSA-9670615 Defective Inhibition of DNA Recombination at Telomere Due to ATRX Mutations R-HSA-9007926 ATRX binds DAXX Telomere Maintenance R-HSA-157579 723 telomere maintenance frameshift loss_of_function of telomere maintenance

PM1-PM4 ATRX ATRX N676Kfs*17 [nucleoplasm] R-HSA-9672619 ATRX UniProt:P46100 Replacement of residues 676 to 691 by KNVMKQLRRNKNYQFQ FragmentReplacedModification COSMIC:COSV64882612 cancer|skin melanoma DOID:162|DOID:8923 R-HSA-9670618-ATRX truncation mutants (DAXX binding region) [nucleoplasm]-CandidateSet-hasCandidate R-HSA-9670619 Defective ATRX does not bind DAXX loss_of_function pubmed:28875424|pubmed:14990586|pubmed:21252315 R-HSA-9670615 Defective Inhibition of DNA Recombination at Telomere Due to ATRX Mutations R-HSA-9007926 ATRX binds DAXX Telomere Maintenance R-HSA-157579 723 telomere maintenance frameshift loss_of_function of telomere maintenance

PM1-PM4 ATRX ATRX V725Gfs*7 [nucleoplasm] R-HSA-9672618 ATRX UniProt:P46100 Replacement of residues 725 to 730 by GSKFRF FragmentReplacedModification COSMIC:COSV64876399 cancer|astrocytoma DOID:162|DOID:3069 R-HSA-9670618-ATRX truncation mutants (DAXX binding region) [nucleoplasm]-CandidateSet-hasCandidate R-HSA-9670619 Defective ATRX does not bind DAXX loss_of_function pubmed:28875424|pubmed:14990586|pubmed:21252315 R-HSA-9670615 Defective Inhibition of DNA Recombination at Telomere Due to ATRX Mutations R-HSA-9007926 ATRX binds DAXX Telomere Maintenance R-HSA-157579 723 telomere maintenance frameshift loss_of_function of telomere maintenance

PM1-PM4 ATRX ATRX I737Kfs*3 [nucleoplasm] R-HSA-9672617 ATRX UniProt:P46100 Replacement of residues 737 to 738 by KR FragmentReplacedModification COSMIC:COSV64870518 cancer|astrocytoma DOID:162|DOID:3069 R-HSA-9670618-ATRX truncation mutants (DAXX binding region) [nucleoplasm]-CandidateSet-hasCandidate R-HSA-9670619 Defective ATRX does not bind DAXX loss_of_function pubmed:28875424|pubmed:14990586|pubmed:21252315 R-HSA-9670615 Defective Inhibition of DNA Recombination at Telomere Due to ATRX Mutations R-HSA-9007926 ATRX binds DAXX Telomere Maintenance R-HSA-157579 723 telomere maintenance frameshift loss_of_function of telomere maintenance

PM1-PM4 ATRX ATRX E723Dfs*9 [nucleoplasm] R-HSA-9672616 ATRX UniProt:P46100 Replacement of residues 723 to 730 by DCGSKFRF FragmentReplacedModification COSMIC:COSV64885165 cancer|brain glioma|papillary thyroid carcinoma DOID:162|DOID:0060108|DOID:3969 R-HSA-9670618-ATRX truncation mutants (DAXX binding region) [nucleoplasm]-CandidateSet-hasCandidate R-HSA-9670619 Defective ATRX does not bind DAXX loss_of_function pubmed:28875424|pubmed:14990586|pubmed:21252315 R-HSA-9670615 Defective Inhibition of DNA Recombination at Telomere Due to ATRX Mutations R-HSA-9007926 ATRX binds DAXX Telomere Maintenance R-HSA-157579 723 telomere maintenance frameshift loss_of_function of telomere maintenance

PM1-PM4 ATRX ATRX Q984* [nucleoplasm] R-HSA-9671598 ATRX UniProt:P46100 Nonsense mutation at L-glutamine 984 NonsenseMutation COSMIC:COSV64877067 leiomyosarcoma|cancer DOID:1967|DOID:162 R-HSA-9670618-ATRX truncation mutants (DAXX binding region) [nucleoplasm]-CandidateSet-hasCandidate R-HSA-9670619 Defective ATRX does not bind DAXX loss_of_function pubmed:28875424|pubmed:14990586|pubmed:21252315 R-HSA-9670615 Defective Inhibition of DNA Recombination at Telomere Due to ATRX Mutations R-HSA-9007926 ATRX binds DAXX Telomere Maintenance R-HSA-157579 723 telomere maintenance nonsense loss_of_function of telomere maintenance

PM1-PM4 ATRX ATRX Q391* [nucleoplasm] R-HSA-9671597 ATRX UniProt:P46100 Nonsense mutation at L-glutamine 391 NonsenseMutation COSMIC:COSV64874680 cancer|astrocytoma DOID:162|DOID:3069 R-HSA-9670618-ATRX truncation mutants (DAXX binding region) [nucleoplasm]-CandidateSet-hasCandidate R-HSA-9670619 Defective ATRX does not bind DAXX loss_of_function pubmed:28875424|pubmed:14990586|pubmed:21252315 R-HSA-9670615 Defective Inhibition of DNA Recombination at Telomere Due to ATRX Mutations R-HSA-9007926 ATRX binds DAXX Telomere Maintenance R-HSA-157579 723 telomere maintenance nonsense loss_of_function of telomere maintenance

PM1-PM4 ATRX ATRX Q545* [nucleoplasm] R-HSA-9671596 ATRX UniProt:P46100 Nonsense mutation at L-glutamine 545 NonsenseMutation COSMIC:COSV64878792 cancer|islet cell tumor DOID:162|DOID:1799 R-HSA-9670618-ATRX truncation mutants (DAXX binding region) [nucleoplasm]-CandidateSet-hasCandidate R-HSA-9670619 Defective ATRX does not bind DAXX loss_of_function pubmed:28875424|pubmed:14990586|pubmed:21252315 R-HSA-9670615 Defective Inhibition of DNA Recombination at Telomere Due to ATRX Mutations R-HSA-9007926 ATRX binds DAXX Telomere Maintenance R-HSA-157579 723 telomere maintenance nonsense loss_of_function of telomere maintenance

PM1-PM4 ATRX ATRX S747Rfs*6 [nucleoplasm] R-HSA-9672620 ATRX UniProt:P46100 Replacement of residues 747 to 751 by RLCMI FragmentReplacedModification COSMIC:COSV64873140 cancer|brain glioma DOID:162|DOID:0060108 R-HSA-9670618-ATRX truncation mutants (DAXX binding region) [nucleoplasm]-CandidateSet-hasCandidate R-HSA-9670619 Defective ATRX does not bind DAXX loss_of_function pubmed:28875424|pubmed:14990586|pubmed:21252315 R-HSA-9670615 Defective Inhibition of DNA Recombination at Telomere Due to ATRX Mutations R-HSA-9007926 ATRX binds DAXX Telomere Maintenance R-HSA-157579 723 telomere maintenance frameshift loss_of_function of telomere maintenance

PM1-PM4 ATRX ATRX E886Lfs*18 [nucleoplasm] R-HSA-9672659 ATRX UniProt:P46100 Replacement of residues 886 to 902 by LSLQQKAQLIKTRPSWN FragmentReplacedModification COSMIC:COSV64874673 cancer|large intestine cancer DOID:162|DOID:5672 R-HSA-9670618-ATRX truncation mutants (DAXX binding region) [nucleoplasm]-CandidateSet-hasCandidate R-HSA-9670619 Defective ATRX does not bind DAXX loss_of_function pubmed:28875424|pubmed:14990586|pubmed:21252315 R-HSA-9670615 Defective Inhibition of DNA Recombination at Telomere Due to ATRX Mutations R-HSA-9007926 ATRX binds DAXX Telomere Maintenance R-HSA-157579 723 telomere maintenance frameshift loss_of_function of telomere maintenance

PM1-PM4 ATRX ATRX S876Lfs*29 [nucleoplasm] R-HSA-9672657 ATRX UniProt:P46100 Replacement of residues 876 to 903 by LMMLKENKRERLSLQQKAQLIKTRPSWN FragmentReplacedModification COSMIC:COSV64879018 cancer|brain glioma DOID:162|DOID:0060108 R-HSA-9670618-ATRX truncation mutants (DAXX binding region) [nucleoplasm]-CandidateSet-hasCandidate R-HSA-9670619 Defective ATRX does not bind DAXX loss_of_function pubmed:28875424|pubmed:14990586|pubmed:21252315 R-HSA-9670615 Defective Inhibition of DNA Recombination at Telomere Due to ATRX Mutations R-HSA-9007926 ATRX binds DAXX Telomere Maintenance R-HSA-157579 723 telomere maintenance frameshift loss_of_function of telomere maintenance

PM1-PM4 ATRX ATRX R808* [nucleoplasm] R-HSA-9671638 ATRX UniProt:P46100 Nonsense mutation at L-arginine 808 NonsenseMutation COSMIC:COSV64874953 cancer|astrocytoma DOID:162|DOID:3069 R-HSA-9670618-ATRX truncation mutants (DAXX binding region) [nucleoplasm]-CandidateSet-hasCandidate R-HSA-9670619 Defective ATRX does not bind DAXX loss_of_function pubmed:28875424|pubmed:14990586|pubmed:21252315 R-HSA-9670615 Defective Inhibition of DNA Recombination at Telomere Due to ATRX Mutations R-HSA-9007926 ATRX binds DAXX Telomere Maintenance R-HSA-157579 723 telomere maintenance nonsense loss_of_function of telomere maintenance

PM1-PM4 ATRX ATRX A834Pfs*35 [nucleoplasm] R-HSA-9672662 ATRX UniProt:P46100 Replacement of residues 834 to 867 by PEPPKKEFQIQKILTLLKMRNTAKKEWIIKGTKI FragmentReplacedModification COSMIC:COSV64875734 cancer|astrocytoma DOID:162|DOID:3069 R-HSA-9670618-ATRX truncation mutants (DAXX binding region) [nucleoplasm]-CandidateSet-hasCandidate R-HSA-9670619 Defective ATRX does not bind DAXX loss_of_function pubmed:28875424|pubmed:14990586|pubmed:21252315 R-HSA-9670615 Defective Inhibition of DNA Recombination at Telomere Due to ATRX Mutations R-HSA-9007926 ATRX binds DAXX Telomere Maintenance R-HSA-157579 723 telomere maintenance frameshift loss_of_function of telomere maintenance

PM1-PM4 ATRX ATRX R666* [nucleoplasm] R-HSA-9671636 ATRX UniProt:P46100 Nonsense mutation at L-arginine 666 NonsenseMutation COSMIC:COSV64871414 cancer|liver carcinoma|astrocytoma DOID:162|DOID:686|DOID:3069 R-HSA-9670618-ATRX truncation mutants (DAXX binding region) [nucleoplasm]-CandidateSet-hasCandidate R-HSA-9670619 Defective ATRX does not bind DAXX loss_of_function pubmed:28875424|pubmed:14990586|pubmed:21252315 R-HSA-9670615 Defective Inhibition of DNA Recombination at Telomere Due to ATRX Mutations R-HSA-9007926 ATRX binds DAXX Telomere Maintenance R-HSA-157579 723 telomere maintenance nonsense loss_of_function of telomere maintenance

PM1-PM4 ATRX ATRX Q883Rfs*13 [nucleoplasm] R-HSA-9672665 ATRX UniProt:P46100 Replacement of residues 883 to 894 by RERDFLFSRRHS FragmentReplacedModification COSMIC:COSV64873219 leiomyosarcoma|cancer DOID:1967|DOID:162 R-HSA-9670618-ATRX truncation mutants (DAXX binding region) [nucleoplasm]-CandidateSet-hasCandidate R-HSA-9670619 Defective ATRX does not bind DAXX loss_of_function pubmed:28875424|pubmed:14990586|pubmed:21252315 R-HSA-9670615 Defective Inhibition of DNA Recombination at Telomere Due to ATRX Mutations R-HSA-9007926 ATRX binds DAXX Telomere Maintenance R-HSA-157579 723 telomere maintenance frameshift loss_of_function of telomere maintenance

PM1-PM4 ATRX ATRX R781* [nucleoplasm] R-HSA-9671640 ATRX UniProt:P46100 Nonsense mutation at L-arginine 781 NonsenseMutation COSMIC:COSV64873866 cancer|skin melanoma|brain glioma|astrocytoma DOID:162|DOID:8923|DOID:0060108|DOID:3069 R-HSA-9670618-ATRX truncation mutants (DAXX binding region) [nucleoplasm]-CandidateSet-hasCandidate R-HSA-9670619 Defective ATRX does not bind DAXX loss_of_function pubmed:28875424|pubmed:14990586|pubmed:21252315 R-HSA-9670615 Defective Inhibition of DNA Recombination at Telomere Due to ATRX Mutations R-HSA-9007926 ATRX binds DAXX Telomere Maintenance R-HSA-157579 723 telomere maintenance nonsense loss_of_function of telomere maintenance

PM1-PM4 ATRX ATRX K823Rfs*7 [nucleoplasm] R-HSA-9672671 ATRX UniProt:P46100 Replacement of residues 823 to 828 by RDKEHE FragmentReplacedModification COSMIC:COSV64872179 cancer|astrocytoma DOID:162|DOID:3069 R-HSA-9670618-ATRX truncation mutants (DAXX binding region) [nucleoplasm]-CandidateSet-hasCandidate R-HSA-9670619 Defective ATRX does not bind DAXX loss_of_function pubmed:28875424|pubmed:14990586|pubmed:21252315 R-HSA-9670615 Defective Inhibition of DNA Recombination at Telomere Due to ATRX Mutations R-HSA-9007926 ATRX binds DAXX Telomere Maintenance R-HSA-157579 723 telomere maintenance frameshift loss_of_function of telomere maintenance

PM1-PM4 ATRX ATRX R840Efs*29 [nucleoplasm] R-HSA-9672670 ATRX UniProt:P46100 Replacement of residues 840 to 867 by EFQIQKILTLLKMRNTAKKEWIIKGTKI FragmentReplacedModification COSMIC:COSV64872356 rhabdomyosarcoma|cancer|large intestine cancer|astrocytoma DOID:3247|DOID:162|DOID:5672|DOID:3069 R-HSA-9670618-ATRX truncation mutants (DAXX binding region) [nucleoplasm]-CandidateSet-hasCandidate R-HSA-9670619 Defective ATRX does not bind DAXX loss_of_function pubmed:28875424|pubmed:14990586|pubmed:21252315 R-HSA-9670615 Defective Inhibition of DNA Recombination at Telomere Due to ATRX Mutations R-HSA-9007926 ATRX binds DAXX Telomere Maintenance R-HSA-157579 723 telomere maintenance frameshift loss_of_function of telomere maintenance

PM1-PM4 ATRX ATRX G859Efs*10 [nucleoplasm] R-HSA-9672643 ATRX UniProt:P46100 Replacement of residues 859 to 867 by EWIIKGTKI FragmentReplacedModification COSMIC:COSV64879300 cancer|large intestine cancer DOID:162|DOID:5672 R-HSA-9670618-ATRX truncation mutants (DAXX binding region) [nucleoplasm]-CandidateSet-hasCandidate R-HSA-9670619 Defective ATRX does not bind DAXX loss_of_function pubmed:28875424|pubmed:14990586|pubmed:21252315 R-HSA-9670615 Defective Inhibition of DNA Recombination at Telomere Due to ATRX Mutations R-HSA-9007926 ATRX binds DAXX Telomere Maintenance R-HSA-157579 723 telomere maintenance frameshift loss_of_function of telomere maintenance

PM1-PM4 ATRX ATRX E886Dfs*10 [nucleoplasm] R-HSA-9672647 ATRX UniProt:P46100 Replacement of residues 886 to 894 by DFLFSRRHS FragmentReplacedModification COSMIC:COSV64885043 cancer|large intestine cancer DOID:162|DOID:5672 R-HSA-9670618-ATRX truncation mutants (DAXX binding region) [nucleoplasm]-CandidateSet-hasCandidate R-HSA-9670619 Defective ATRX does not bind DAXX loss_of_function pubmed:28875424|pubmed:14990586|pubmed:21252315 R-HSA-9670615 Defective Inhibition of DNA Recombination at Telomere Due to ATRX Mutations R-HSA-9007926 ATRX binds DAXX Telomere Maintenance R-HSA-157579 723 telomere maintenance frameshift loss_of_function of telomere maintenance

PM1-PM4 ATRX ATRX F888Sfs*17 [nucleoplasm] R-HSA-9672651 ATRX UniProt:P46100 Replacement of residues 888 to 903 by SLQQKAQLIKTRPSWN FragmentReplacedModification COSMIC:COSV64877038 cancer|brain glioma|astrocytoma DOID:162|DOID:0060108|DOID:3069 R-HSA-9670618-ATRX truncation mutants (DAXX binding region) [nucleoplasm]-CandidateSet-hasCandidate R-HSA-9670619 Defective ATRX does not bind DAXX loss_of_function pubmed:28875424|pubmed:14990586|pubmed:21252315 R-HSA-9670615 Defective Inhibition of DNA Recombination at Telomere Due to ATRX Mutations R-HSA-9007926 ATRX binds DAXX Telomere Maintenance R-HSA-157579 723 telomere maintenance frameshift loss_of_function of telomere maintenance

PM1-PM4 ATRX ATRX S784Lfs*13 [nucleoplasm] R-HSA-9672654 ATRX UniProt:P46100 Replacement of residues 784 to 795 by LILKRANQLRAL FragmentReplacedModification COSMIC:COSV64878925 cancer|astrocytoma DOID:162|DOID:3069 R-HSA-9670618-ATRX truncation mutants (DAXX binding region) [nucleoplasm]-CandidateSet-hasCandidate R-HSA-9670619 Defective ATRX does not bind DAXX loss_of_function pubmed:28875424|pubmed:14990586|pubmed:21252315 R-HSA-9670615 Defective Inhibition of DNA Recombination at Telomere Due to ATRX Mutations R-HSA-9007926 ATRX binds DAXX Telomere Maintenance R-HSA-157579 723 telomere maintenance frameshift loss_of_function of telomere maintenance

PM1-PM4 ATRX ATRX S871Hfs*34 [nucleoplasm] R-HSA-9672653 ATRX UniProt:P46100 Replacement of residues 871 to 903 by HKKDHLMMLKENKRERLSLQQKAQLIKTRPSWN FragmentReplacedModification COSMIC:COSV64879434 cancer|brain glioma DOID:162|DOID:0060108 R-HSA-9670618-ATRX truncation mutants (DAXX binding region) [nucleoplasm]-CandidateSet-hasCandidate R-HSA-9670619 Defective ATRX does not bind DAXX loss_of_function pubmed:28875424|pubmed:14990586|pubmed:21252315 R-HSA-9670615 Defective Inhibition of DNA Recombination at Telomere Due to ATRX Mutations R-HSA-9007926 ATRX binds DAXX Telomere Maintenance R-HSA-157579 723 telomere maintenance frameshift loss_of_function of telomere maintenance

PM1-PM4 ATRX ATRX I1049Mfs*3 [nucleoplasm] R-HSA-9672691 ATRX UniProt:P46100 Replacement of residues 1049 to 1050 by MR FragmentReplacedModification COSMIC:COSV64879667 cancer|astrocytoma DOID:162|DOID:3069 R-HSA-9670618-ATRX truncation mutants (DAXX binding region) [nucleoplasm]-CandidateSet-hasCandidate R-HSA-9670619 Defective ATRX does not bind DAXX loss_of_function pubmed:28875424|pubmed:14990586|pubmed:21252315 R-HSA-9670615 Defective Inhibition of DNA Recombination at Telomere Due to ATRX Mutations R-HSA-9007926 ATRX binds DAXX Telomere Maintenance R-HSA-157579 723 telomere maintenance frameshift loss_of_function of telomere maintenance

PM1-PM4 ATRX ATRX K562* [nucleoplasm] R-HSA-9671665 ATRX UniProt:P46100 Nonsense mutation at L-lysine 562 NonsenseMutation COSMIC:COSV64873800 leiomyosarcoma|cancer DOID:1967|DOID:162 R-HSA-9670618-ATRX truncation mutants (DAXX binding region) [nucleoplasm]-CandidateSet-hasCandidate R-HSA-9670619 Defective ATRX does not bind DAXX loss_of_function pubmed:28875424|pubmed:14990586|pubmed:21252315 R-HSA-9670615 Defective Inhibition of DNA Recombination at Telomere Due to ATRX Mutations R-HSA-9007926 ATRX binds DAXX Telomere Maintenance R-HSA-157579 723 telomere maintenance nonsense loss_of_function of telomere maintenance

PM1-PM4 ATRX ATRX K1045* [nucleoplasm] R-HSA-9671664 ATRX UniProt:P46100 Nonsense mutation at L-lysine 1045 NonsenseMutation COSMIC:COSV64873817 cancer|brain glioma|medulloblastoma|astrocytoma DOID:162|DOID:0060108|DOID:0050902|DOID:3069 R-HSA-9670618-ATRX truncation mutants (DAXX binding region) [nucleoplasm]-CandidateSet-hasCandidate R-HSA-9670619 Defective ATRX does not bind DAXX loss_of_function pubmed:28875424|pubmed:14990586|pubmed:21252315 R-HSA-9670615 Defective Inhibition of DNA Recombination at Telomere Due to ATRX Mutations R-HSA-9007926 ATRX binds DAXX Telomere Maintenance R-HSA-157579 723 telomere maintenance nonsense loss_of_function of telomere maintenance

PM1-PM4 ATRX ATRX K910* [nucleoplasm] R-HSA-9671671 ATRX UniProt:P46100 Nonsense mutation at L-lysine 910 NonsenseMutation COSMIC:COSV64872211 cancer|angiomyolipoma DOID:162|DOID:3314 R-HSA-9670618-ATRX truncation mutants (DAXX binding region) [nucleoplasm]-CandidateSet-hasCandidate R-HSA-9670619 Defective ATRX does not bind DAXX loss_of_function pubmed:28875424|pubmed:14990586|pubmed:21252315 R-HSA-9670615 Defective Inhibition of DNA Recombination at Telomere Due to ATRX Mutations R-HSA-9007926 ATRX binds DAXX Telomere Maintenance R-HSA-157579 723 telomere maintenance nonsense loss_of_function of telomere maintenance

PM1-PM4 ATRX ATRX K993Rfs*10 [nucleoplasm] R-HSA-9672695 ATRX UniProt:P46100 Replacement of residues 993 to 1001 by RNLQTLRKK FragmentReplacedModification COSMIC:COSV64872428 cancer|large intestine cancer DOID:162|DOID:5672 R-HSA-9670618-ATRX truncation mutants (DAXX binding region) [nucleoplasm]-CandidateSet-hasCandidate R-HSA-9670619 Defective ATRX does not bind DAXX loss_of_function pubmed:28875424|pubmed:14990586|pubmed:21252315 R-HSA-9670615 Defective Inhibition of DNA Recombination at Telomere Due to ATRX Mutations R-HSA-9007926 ATRX binds DAXX Telomere Maintenance R-HSA-157579 723 telomere maintenance frameshift loss_of_function of telomere maintenance

PM1-PM4 ATRX ATRX K967* [nucleoplasm] R-HSA-9671668 ATRX UniProt:P46100 Nonsense mutation at L-lysine 967 NonsenseMutation COSMIC:COSV64877893 cancer|large intestine cancer DOID:162|DOID:5672 R-HSA-9670618-ATRX truncation mutants (DAXX binding region) [nucleoplasm]-CandidateSet-hasCandidate R-HSA-9670619 Defective ATRX does not bind DAXX loss_of_function pubmed:28875424|pubmed:14990586|pubmed:21252315 R-HSA-9670615 Defective Inhibition of DNA Recombination at Telomere Due to ATRX Mutations R-HSA-9007926 ATRX binds DAXX Telomere Maintenance R-HSA-157579 723 telomere maintenance nonsense loss_of_function of telomere maintenance

PM1-PM4 ATRX ATRX K823* [nucleoplasm] R-HSA-9671675 ATRX UniProt:P46100 Nonsense mutation at L-lysine 823 NonsenseMutation COSMIC:COSV64883624 myelodysplastic syndrome|cancer DOID:0050908|DOID:162 R-HSA-9670618-ATRX truncation mutants (DAXX binding region) [nucleoplasm]-CandidateSet-hasCandidate R-HSA-9670619 Defective ATRX does not bind DAXX loss_of_function pubmed:28875424|pubmed:14990586|pubmed:21252315 R-HSA-9670615 Defective Inhibition of DNA Recombination at Telomere Due to ATRX Mutations R-HSA-9007926 ATRX binds DAXX Telomere Maintenance R-HSA-157579 723 telomere maintenance nonsense loss_of_function of telomere maintenance

PM1-PM4 ATRX ATRX K782* [nucleoplasm] R-HSA-9671674 ATRX UniProt:P46100 Nonsense mutation at L-lysine 782 NonsenseMutation COSMIC:COSV64882713 lung adenocarcinoma|cancer DOID:3910|DOID:162 R-HSA-9670618-ATRX truncation mutants (DAXX binding region) [nucleoplasm]-CandidateSet-hasCandidate R-HSA-9670619 Defective ATRX does not bind DAXX loss_of_function pubmed:28875424|pubmed:14990586|pubmed:21252315 R-HSA-9670615 Defective Inhibition of DNA Recombination at Telomere Due to ATRX Mutations R-HSA-9007926 ATRX binds DAXX Telomere Maintenance R-HSA-157579 723 telomere maintenance nonsense loss_of_function of telomere maintenance

PM1-PM4 ATRX ATRX V1002* [nucleoplasm] R-HSA-9672698 ATRX UniProt:P46100 Nonsense mutation at L-valine 1002 NonsenseMutation COSMIC:COSV64877476 cancer|large intestine cancer|astrocytoma DOID:162|DOID:5672|DOID:3069 R-HSA-9670618-ATRX truncation mutants (DAXX binding region) [nucleoplasm]-CandidateSet-hasCandidate R-HSA-9670619 Defective ATRX does not bind DAXX loss_of_function pubmed:28875424|pubmed:14990586|pubmed:21252315 R-HSA-9670615 Defective Inhibition of DNA Recombination at Telomere Due to ATRX Mutations R-HSA-9007926 ATRX binds DAXX Telomere Maintenance R-HSA-157579 723 telomere maintenance nonsense loss_of_function of telomere maintenance

PM1-PM4 ATRX ATRX E1017Dfs*5 [nucleoplasm] R-HSA-9672702 ATRX UniProt:P46100 Replacement of residues 1017 to 1020 by DLRA FragmentReplacedModification COSMIC:COSV64880087 cancer|astrocytoma DOID:162|DOID:3069 R-HSA-9670618-ATRX truncation mutants (DAXX binding region) [nucleoplasm]-CandidateSet-hasCandidate R-HSA-9670619 Defective ATRX does not bind DAXX loss_of_function pubmed:28875424|pubmed:14990586|pubmed:21252315 R-HSA-9670615 Defective Inhibition of DNA Recombination at Telomere Due to ATRX Mutations R-HSA-9007926 ATRX binds DAXX Telomere Maintenance R-HSA-157579 723 telomere maintenance frameshift loss_of_function of telomere maintenance

PM1-PM4 ATRX ATRX K983* [nucleoplasm] R-HSA-9671659 ATRX UniProt:P46100 Nonsense mutation at L-lysine 983 NonsenseMutation COSMIC:COSV64883878 ovary serous adenocarcinoma|cancer DOID:5744|DOID:162 R-HSA-9670618-ATRX truncation mutants (DAXX binding region) [nucleoplasm]-CandidateSet-hasCandidate R-HSA-9670619 Defective ATRX does not bind DAXX loss_of_function pubmed:28875424|pubmed:14990586|pubmed:21252315 R-HSA-9670615 Defective Inhibition of DNA Recombination at Telomere Due to ATRX Mutations R-HSA-9007926 ATRX binds DAXX Telomere Maintenance R-HSA-157579 723 telomere maintenance nonsense loss_of_function of telomere maintenance

PM1-PM4 ATRX ATRX K455* [nucleoplasm] R-HSA-9671657 ATRX UniProt:P46100 Nonsense mutation at L-lysine 455 NonsenseMutation COSMIC:COSV64877606 cancer|islet cell tumor DOID:162|DOID:1799 R-HSA-9670618-ATRX truncation mutants (DAXX binding region) [nucleoplasm]-CandidateSet-hasCandidate R-HSA-9670619 Defective ATRX does not bind DAXX loss_of_function pubmed:28875424|pubmed:14990586|pubmed:21252315 R-HSA-9670615 Defective Inhibition of DNA Recombination at Telomere Due to ATRX Mutations R-HSA-9007926 ATRX binds DAXX Telomere Maintenance R-HSA-157579 723 telomere maintenance nonsense loss_of_function of telomere maintenance

PM1-PM4 ATRX ATRX K1052* [nucleoplasm] R-HSA-9671656 ATRX UniProt:P46100 Nonsense mutation at L-lysine 1052 NonsenseMutation COSMIC:COSV64873492 angiosarcoma|cancer DOID:0001816|DOID:162 R-HSA-9670618-ATRX truncation mutants (DAXX binding region) [nucleoplasm]-CandidateSet-hasCandidate R-HSA-9670619 Defective ATRX does not bind DAXX loss_of_function pubmed:28875424|pubmed:14990586|pubmed:21252315 R-HSA-9670615 Defective Inhibition of DNA Recombination at Telomere Due to ATRX Mutations R-HSA-9007926 ATRX binds DAXX Telomere Maintenance R-HSA-157579 723 telomere maintenance nonsense loss_of_function of telomere maintenance

PM1-PM4 ATRX ATRX K971Tfs*31 [nucleoplasm] R-HSA-9672686 ATRX UniProt:P46100 Replacement of residues 971 to 1000 by TRAMKLLKMIKSRAKRELKKKRNLQTLRKK FragmentReplacedModification COSMIC:COSV64886436 cancer|astrocytoma DOID:162|DOID:3069 R-HSA-9670618-ATRX truncation mutants (DAXX binding region) [nucleoplasm]-CandidateSet-hasCandidate R-HSA-9670619 Defective ATRX does not bind DAXX loss_of_function pubmed:28875424|pubmed:14990586|pubmed:21252315 R-HSA-9670615 Defective Inhibition of DNA Recombination at Telomere Due to ATRX Mutations R-HSA-9007926 ATRX binds DAXX Telomere Maintenance R-HSA-157579 723 telomere maintenance frameshift loss_of_function of telomere maintenance

PM1-PM4 ATRX ATRX K459* [nucleoplasm] R-HSA-9671660 ATRX UniProt:P46100 Nonsense mutation at L-lysine 459 NonsenseMutation COSMIC:COSV64886139 ocular melanoma|cancer DOID:1752|DOID:162 R-HSA-9670618-ATRX truncation mutants (DAXX binding region) [nucleoplasm]-CandidateSet-hasCandidate R-HSA-9670619 Defective ATRX does not bind DAXX loss_of_function pubmed:28875424|pubmed:14990586|pubmed:21252315 R-HSA-9670615 Defective Inhibition of DNA Recombination at Telomere Due to ATRX Mutations R-HSA-9007926 ATRX binds DAXX Telomere Maintenance R-HSA-157579 723 telomere maintenance nonsense loss_of_function of telomere maintenance

PM1-PM4 ATRX ATRX T1172Lfs*18 [nucleoplasm] R-HSA-9672734 ATRX UniProt:P46100 Replacement of residues 1172 to 1188 by LHLKRRQSLSRRKRETP FragmentReplacedModification COSMIC:COSV64880080 cancer|skin melanoma DOID:162|DOID:8923 R-HSA-9670618-ATRX truncation mutants (DAXX binding region) [nucleoplasm]-CandidateSet-hasCandidate R-HSA-9670619 Defective ATRX does not bind DAXX loss_of_function pubmed:28875424|pubmed:14990586|pubmed:21252315 R-HSA-9670615 Defective Inhibition of DNA Recombination at Telomere Due to ATRX Mutations R-HSA-9007926 ATRX binds DAXX Telomere Maintenance R-HSA-157579 723 telomere maintenance frameshift loss_of_function of telomere maintenance

PM1-PM4 ATRX ATRX K826* [nucleoplasm] R-HSA-9671681 ATRX UniProt:P46100 Nonsense mutation at L-lysine 826 NonsenseMutation COSMIC:COSV64886132 lung adenocarcinoma|cancer DOID:3910|DOID:162 R-HSA-9670618-ATRX truncation mutants (DAXX binding region) [nucleoplasm]-CandidateSet-hasCandidate R-HSA-9670619 Defective ATRX does not bind DAXX loss_of_function pubmed:28875424|pubmed:14990586|pubmed:21252315 R-HSA-9670615 Defective Inhibition of DNA Recombination at Telomere Due to ATRX Mutations R-HSA-9007926 ATRX binds DAXX Telomere Maintenance R-HSA-157579 723 telomere maintenance nonsense loss_of_function of telomere maintenance

PM1-PM4 ATRX ATRX I1049Kfs*69 [nucleoplasm] R-HSA-9672705 ATRX UniProt:P46100 Replacement of residues 1049 to 1116 by KEIKLLKRRMNYLIMLRSQQGKEIVVTLQRIKRVRMEHMVERRKGASCLERVQGRDKIVHHLILRNIP FragmentReplacedModification COSMIC:COSV64880556 cancer|brain glioma DOID:162|DOID:0060108 R-HSA-9670618-ATRX truncation mutants (DAXX binding region) [nucleoplasm]-CandidateSet-hasCandidate R-HSA-9670619 Defective ATRX does not bind DAXX loss_of_function pubmed:28875424|pubmed:14990586|pubmed:21252315 R-HSA-9670615 Defective Inhibition of DNA Recombination at Telomere Due to ATRX Mutations R-HSA-9007926 ATRX binds DAXX Telomere Maintenance R-HSA-157579 723 telomere maintenance frameshift loss_of_function of telomere maintenance

PM1-PM4 ATRX ATRX K945Rfs*25 [nucleoplasm] R-HSA-9672711 ATRX UniProt:P46100 Replacement of residues 945 to 968 by RASISKPKHVKKYRMAYLILQRNS FragmentReplacedModification COSMIC:COSV64874687 cancer|brain glioma DOID:162|DOID:0060108 R-HSA-9670618-ATRX truncation mutants (DAXX binding region) [nucleoplasm]-CandidateSet-hasCandidate R-HSA-9670619 Defective ATRX does not bind DAXX loss_of_function pubmed:28875424|pubmed:14990586|pubmed:21252315 R-HSA-9670615 Defective Inhibition of DNA Recombination at Telomere Due to ATRX Mutations R-HSA-9007926 ATRX binds DAXX Telomere Maintenance R-HSA-157579 723 telomere maintenance frameshift loss_of_function of telomere maintenance

PM1-PM4 ATRX ATRX K1001Nfs*3 [nucleoplasm] R-HSA-9672709 ATRX UniProt:P46100 Replacement of residues 1001 to 1002 by NN FragmentReplacedModification COSMIC:COSV64879718 leiomyosarcoma|cancer DOID:1967|DOID:162 R-HSA-9670618-ATRX truncation mutants (DAXX binding region) [nucleoplasm]-CandidateSet-hasCandidate R-HSA-9670619 Defective ATRX does not bind DAXX loss_of_function pubmed:28875424|pubmed:14990586|pubmed:21252315 R-HSA-9670615 Defective Inhibition of DNA Recombination at Telomere Due to ATRX Mutations R-HSA-9007926 ATRX binds DAXX Telomere Maintenance R-HSA-157579 723 telomere maintenance frameshift loss_of_function of telomere maintenance

PS3 AVP AVP G23R [extracellular region] R-HSA-5621464 AVP UniProt:P01185 glycine 23 replaced with L-arginine ReplacedResidue neurohypophyseal diabetes insipidus DOID:12388 R-HSA-5621383-AVP mutants [extracellular region]-DefinedSet-hasMember R-HSA-9035517|R-HSA-5621425 "Defective AVP mutants do not bind AVPR2|Defective AVP mutants do not bind AVPR1A,B" loss_of_function pubmed:23360744|pubmed:10369876|pubmed:8514868|pubmed:9467595|pubmed:8103767|pubmed:14673472|pubmed:7714110|pubmed:12107248 R-HSA-9036092|R-HSA-5619099 "Defective AVP does not bind AVPR2 and causes neurohypophyseal diabetes insipidus (NDI)|Defective AVP does not bind AVPR1A,B and causes neurohypophyseal diabetes insipidus (NDI)" R-HSA-392263|R-HSA-388468 "AVP(20-28) binds AVPR2|AVP(20-28) binds AVPR1A,B" Vasopressin regulates renal water homeostasis via Aquaporins|Class A/1 (Rhodopsin-like receptors) R-HSA-432040|R-HSA-373076 0003091|0007186 renal water homeostasis|G protein-coupled receptor signaling pathway missense loss_of_function of renal water homeostasis|G protein-coupled receptor signaling pathway

PS3 AVP AVP Y2H [extracellular region] R-HSA-5621449 AVP UniProt:P01185 L-tyrosine 2 replaced with L-histidine ReplacedResidue neurohypophyseal diabetes insipidus DOID:12388 R-HSA-5621383-AVP mutants [extracellular region]-DefinedSet-hasMember R-HSA-9035517|R-HSA-5621425 "Defective AVP mutants do not bind AVPR2|Defective AVP mutants do not bind AVPR1A,B" loss_of_function pubmed:23360744|pubmed:10369876|pubmed:8514868|pubmed:9467595|pubmed:8103767|pubmed:14673472|pubmed:7714110|pubmed:12107248 R-HSA-9036092|R-HSA-5619099 "Defective AVP does not bind AVPR2 and causes neurohypophyseal diabetes insipidus (NDI)|Defective AVP does not bind AVPR1A,B and causes neurohypophyseal diabetes insipidus (NDI)" R-HSA-392263|R-HSA-388468 "AVP(20-28) binds AVPR2|AVP(20-28) binds AVPR1A,B" Vasopressin regulates renal water homeostasis via Aquaporins|Class A/1 (Rhodopsin-like receptors) R-HSA-432040|R-HSA-373076 0003091|0007186 renal water homeostasis|G protein-coupled receptor signaling pathway missense loss_of_function of renal water homeostasis|G protein-coupled receptor signaling pathway

PS3 AVP AVP A1T [extracellular region] R-HSA-5621473 AVP UniProt:P01185 L-alanine 1 replaced with L-threonine ReplacedResidue neurohypophyseal diabetes insipidus DOID:12388 R-HSA-5621383-AVP mutants [extracellular region]-DefinedSet-hasMember R-HSA-9035517|R-HSA-5621425 "Defective AVP mutants do not bind AVPR2|Defective AVP mutants do not bind AVPR1A,B" loss_of_function pubmed:23360744|pubmed:10369876|pubmed:8514868|pubmed:9467595|pubmed:8103767|pubmed:14673472|pubmed:7714110|pubmed:12107248 R-HSA-9036092|R-HSA-5619099 "Defective AVP does not bind AVPR2 and causes neurohypophyseal diabetes insipidus (NDI)|Defective AVP does not bind AVPR1A,B and causes neurohypophyseal diabetes insipidus (NDI)" R-HSA-392263|R-HSA-388468 "AVP(20-28) binds AVPR2|AVP(20-28) binds AVPR1A,B" Vasopressin regulates renal water homeostasis via Aquaporins|Class A/1 (Rhodopsin-like receptors) R-HSA-432040|R-HSA-373076 0003091|0007186 renal water homeostasis|G protein-coupled receptor signaling pathway missense loss_of_function of renal water homeostasis|G protein-coupled receptor signaling pathway

PS3 AVP AVP V67A [extracellular region] R-HSA-5621446 AVP UniProt:P01185 L-valine 67 replaced with L-alanine ReplacedResidue neurohypophyseal diabetes insipidus DOID:12388 R-HSA-5621383-AVP mutants [extracellular region]-DefinedSet-hasMember R-HSA-9035517|R-HSA-5621425 "Defective AVP mutants do not bind AVPR2|Defective AVP mutants do not bind AVPR1A,B" loss_of_function pubmed:23360744|pubmed:10369876|pubmed:8514868|pubmed:9467595|pubmed:8103767|pubmed:14673472|pubmed:7714110|pubmed:12107248 R-HSA-9036092|R-HSA-5619099 "Defective AVP does not bind AVPR2 and causes neurohypophyseal diabetes insipidus (NDI)|Defective AVP does not bind AVPR1A,B and causes neurohypophyseal diabetes insipidus (NDI)" R-HSA-392263|R-HSA-388468 "AVP(20-28) binds AVPR2|AVP(20-28) binds AVPR1A,B" Vasopressin regulates renal water homeostasis via Aquaporins|Class A/1 (Rhodopsin-like receptors) R-HSA-432040|R-HSA-373076 0003091|0007186 renal water homeostasis|G protein-coupled receptor signaling pathway missense loss_of_function of renal water homeostasis|G protein-coupled receptor signaling pathway

PS3 AVP AVP C67* [extracellular region] R-HSA-5621431 AVP UniProt:P01185 Nonsense mutation at L-cysteine 67 NonsenseMutation neurohypophyseal diabetes insipidus DOID:12388 R-HSA-5621383-AVP mutants [extracellular region]-DefinedSet-hasMember R-HSA-9035517|R-HSA-5621425 "Defective AVP mutants do not bind AVPR2|Defective AVP mutants do not bind AVPR1A,B" loss_of_function pubmed:23360744|pubmed:10369876|pubmed:8514868|pubmed:9467595|pubmed:8103767|pubmed:14673472|pubmed:7714110|pubmed:12107248 R-HSA-9036092|R-HSA-5619099 "Defective AVP does not bind AVPR2 and causes neurohypophyseal diabetes insipidus (NDI)|Defective AVP does not bind AVPR1A,B and causes neurohypophyseal diabetes insipidus (NDI)" R-HSA-392263|R-HSA-388468 "AVP(20-28) binds AVPR2|AVP(20-28) binds AVPR1A,B" Vasopressin regulates renal water homeostasis via Aquaporins|Class A/1 (Rhodopsin-like receptors) R-HSA-432040|R-HSA-373076 0003091|0007186 renal water homeostasis|G protein-coupled receptor signaling pathway nonsense loss_of_function of renal water homeostasis|G protein-coupled receptor signaling pathway

PS3 AVP AVP P7L [extracellular region] R-HSA-5621460 AVP UniProt:P01185 L-proline 7 replaced with L-leucine ReplacedResidue neurohypophyseal diabetes insipidus DOID:12388 R-HSA-5621383-AVP mutants [extracellular region]-DefinedSet-hasMember R-HSA-9035517|R-HSA-5621425 "Defective AVP mutants do not bind AVPR2|Defective AVP mutants do not bind AVPR1A,B" loss_of_function pubmed:23360744|pubmed:10369876|pubmed:8514868|pubmed:9467595|pubmed:8103767|pubmed:14673472|pubmed:7714110|pubmed:12107248 R-HSA-9036092|R-HSA-5619099 "Defective AVP does not bind AVPR2 and causes neurohypophyseal diabetes insipidus (NDI)|Defective AVP does not bind AVPR1A,B and causes neurohypophyseal diabetes insipidus (NDI)" R-HSA-392263|R-HSA-388468 "AVP(20-28) binds AVPR2|AVP(20-28) binds AVPR1A,B" Vasopressin regulates renal water homeostasis via Aquaporins|Class A/1 (Rhodopsin-like receptors) R-HSA-432040|R-HSA-373076 0003091|0007186 renal water homeostasis|G protein-coupled receptor signaling pathway missense loss_of_function of renal water homeostasis|G protein-coupled receptor signaling pathway

PS3 AVP AVP E47del [extracellular region] R-HSA-5621396 AVP UniProt:P01185 Deletion of residues 47 to 47 FragmentDeletionModification neurohypophyseal diabetes insipidus DOID:12388 R-HSA-5621383-AVP mutants [extracellular region]-DefinedSet-hasMember R-HSA-9035517|R-HSA-5621425 "Defective AVP mutants do not bind AVPR2|Defective AVP mutants do not bind AVPR1A,B" loss_of_function pubmed:23360744|pubmed:10369876|pubmed:8514868|pubmed:9467595|pubmed:8103767|pubmed:14673472|pubmed:7714110|pubmed:12107248 R-HSA-9036092|R-HSA-5619099 "Defective AVP does not bind AVPR2 and causes neurohypophyseal diabetes insipidus (NDI)|Defective AVP does not bind AVPR1A,B and causes neurohypophyseal diabetes insipidus (NDI)" R-HSA-392263|R-HSA-388468 "AVP(20-28) binds AVPR2|AVP(20-28) binds AVPR1A,B" Vasopressin regulates renal water homeostasis via Aquaporins|Class A/1 (Rhodopsin-like receptors) R-HSA-432040|R-HSA-373076 0003091|0007186 renal water homeostasis|G protein-coupled receptor signaling pathway in-frame indel: deletion loss_of_function of renal water homeostasis|G protein-coupled receptor signaling pathway

PS3 AXIN1 AXIN1 L396M [cytosol] R-HSA-5251503 AXIN1 UniProt:O15169 L-leucine 396 replaced with L-methionine ReplacedResidue COSMIC:COSV51990449 colorectal cancer|cancer DOID:9256|DOID:162 pubmed:10862053 R-HSA-4839738-truncation and missense AXIN mutants [cytosol]-CandidateSet-hasMember R-HSA-4839734 AXIN mutants destabilize the destruction complex loss_of_function|decreased_transcript_level pubmed:10862053|pubmed:15735151|pubmed:10700176|pubmed:11746989|pubmed:12101426 R-HSA-5467340 AXIN missense mutants destabilize the destruction complex R-HSA-195251 Assembly of the destruction complex Degradation of beta-catenin by the destruction complex R-HSA-195253 1904885 beta-catenin destruction complex assembly missense NA

PS3 AXIN1 AXIN1 Y346* [cytosol] R-HSA-4839753 AXIN1 UniProt:O15169 Nonsense mutation at L-tyrosine 346 NonsenseMutation COSMIC:COSM142836 hepatocellular carcinoma|cancer DOID:684|DOID:162 pubmed:10700176 R-HSA-4839738-truncation and missense AXIN mutants [cytosol]-CandidateSet-hasMember R-HSA-4839734 AXIN mutants destabilize the destruction complex loss_of_function|decreased_transcript_level pubmed:10862053|pubmed:15735151|pubmed:10700176|pubmed:11746989|pubmed:12101426 R-HSA-5467340 AXIN missense mutants destabilize the destruction complex R-HSA-195251 Assembly of the destruction complex Degradation of beta-catenin by the destruction complex R-HSA-195253 1904885 beta-catenin destruction complex assembly nonsense NA

PM1-PM4 AXIN1 AXIN1 E406* [cytosol] R-HSA-5251500 AXIN1 UniProt:O15169 Nonsense mutation at L-glutamic acid 406 NonsenseMutation COSMIC:COSV51986188 hepatocellular carcinoma|cancer DOID:684|DOID:162 pubmed:10700176 R-HSA-4839738-truncation and missense AXIN mutants [cytosol]-CandidateSet-hasCandidate R-HSA-4839734 AXIN mutants destabilize the destruction complex loss_of_function|decreased_transcript_level pubmed:10862053|pubmed:15735151|pubmed:10700176|pubmed:11746989|pubmed:12101426 R-HSA-5467340 AXIN missense mutants destabilize the destruction complex R-HSA-195251 Assembly of the destruction complex Degradation of beta-catenin by the destruction complex R-HSA-195253 1904885 beta-catenin destruction complex assembly nonsense NA

PM1-PM4 AXIN1 AXIN1 W247* [cytosol] R-HSA-5251512 AXIN1 UniProt:O15169 Nonsense mutation at L-tryptophan 247 NonsenseMutation COSMIC:COSV51989213 hepatocellular carcinoma|cancer DOID:684|DOID:162 pubmed:10700176 R-HSA-4839738-truncation and missense AXIN mutants [cytosol]-CandidateSet-hasCandidate R-HSA-4839734 AXIN mutants destabilize the destruction complex loss_of_function|decreased_transcript_level pubmed:10862053|pubmed:15735151|pubmed:10700176|pubmed:11746989|pubmed:12101426 R-HSA-5467340 AXIN missense mutants destabilize the destruction complex R-HSA-195251 Assembly of the destruction complex Degradation of beta-catenin by the destruction complex R-HSA-195253 1904885 beta-catenin destruction complex assembly nonsense NA

PM5 AXIN1 AXIN1 P354L [cytosol] R-HSA-5251511 AXIN1 UniProt:O15169 L-proline 354 replaced with L-leucine ReplacedResidue COSMIC:COSV51983922 hepatocellular carcinoma|cancer DOID:684|DOID:162 pubmed:12101426 R-HSA-4839738-truncation and missense AXIN mutants [cytosol]-CandidateSet-hasCandidate R-HSA-4839734 AXIN mutants destabilize the destruction complex loss_of_function|decreased_transcript_level pubmed:10862053|pubmed:15735151|pubmed:10700176|pubmed:11746989|pubmed:12101426 R-HSA-5467340 AXIN missense mutants destabilize the destruction complex R-HSA-195251 Assembly of the destruction complex Degradation of beta-catenin by the destruction complex R-HSA-195253 1904885 beta-catenin destruction complex assembly missense NA

PM5 AXIN1 AXIN1 P255S [cytosol] R-HSA-5251505 AXIN1 UniProt:O15169 L-proline 255 replaced with L-serine ReplacedResidue cancer|medulloblastoma DOID:162|DOID:0050902 pubmed:12555076|pubmed:11585731 R-HSA-4839738-truncation and missense AXIN mutants [cytosol]-CandidateSet-hasCandidate R-HSA-4839734 AXIN mutants destabilize the destruction complex loss_of_function|decreased_transcript_level pubmed:10862053|pubmed:15735151|pubmed:10700176|pubmed:11746989|pubmed:12101426 R-HSA-5467340 AXIN missense mutants destabilize the destruction complex R-HSA-195251 Assembly of the destruction complex Degradation of beta-catenin by the destruction complex R-HSA-195253 1904885 beta-catenin destruction complex assembly missense NA

PS3 B3GALT6 B3GALT6 P67L [Golgi membrane] R-HSA-4420348 B3GALT6 UniProt:Q96L58 L-proline 67 replaced with L-leucine ReplacedResidue spondyloepimetaphyseal dysplasia DOID:0080027 R-HSA-4420346-B3GALT6 mutants [Golgi membrane]-DefinedSet-hasMember R-HSA-4420365 Defective B3GALT6 does not transfer Gal to the tetrasaccharide linker loss_of_function pubmed:10506123|pubmed:23664118|pubmed:23664117 R-HSA-4420332 Defective B3GALT6 causes EDSP2 and SEMDJL1 R-HSA-1889978 B3GALT6 transfers Gal to the tetrasaccharide linker Glycosaminoglycan metabolism R-HSA-1630316 30203 glycosaminoglycan metabolic process missense loss_of_function of glycosaminoglycan metabolic process

PS3 B3GALT6 B3GALT6 D207H [Golgi membrane] R-HSA-4420350 B3GALT6 UniProt:Q96L58 L-aspartic acid 207 replaced with L-histidine ReplacedResidue spondyloepimetaphyseal dysplasia DOID:0080027 R-HSA-4420346-B3GALT6 mutants [Golgi membrane]-DefinedSet-hasMember R-HSA-4420365 Defective B3GALT6 does not transfer Gal to the tetrasaccharide linker loss_of_function pubmed:10506123|pubmed:23664118|pubmed:23664117 R-HSA-4420332 Defective B3GALT6 causes EDSP2 and SEMDJL1 R-HSA-1889978 B3GALT6 transfers Gal to the tetrasaccharide linker Glycosaminoglycan metabolism R-HSA-1630316 30203 glycosaminoglycan metabolic process missense loss_of_function of glycosaminoglycan metabolic process

PS3 B3GALT6 B3GALT6 D118Afs*160 [Golgi membrane] R-HSA-4420334 B3GALT6 UniProt:Q96L58 Replacement of residues 118 to 276 by ACCCCPRCATPTKTSRPRCWPCWPGWTSTWPSSSCSRRTTTPSRGWTRCWPSCAPASPRAAAASTGASSRAAAASSRGGAGARPPGNSATTTCPTRWAAATCSRPTWCTTCASAATTCAPGTARTCLWAPGWRRWTSSGSTTRASTPNTGPAAAATSTW FragmentReplacedModification Ehlers-Danlos syndrome DOID:13359 R-HSA-4420346-B3GALT6 mutants [Golgi membrane]-DefinedSet-hasMember R-HSA-4420365 Defective B3GALT6 does not transfer Gal to the tetrasaccharide linker loss_of_function pubmed:10506123|pubmed:23664118|pubmed:23664117 R-HSA-4420332 Defective B3GALT6 causes EDSP2 and SEMDJL1 R-HSA-1889978 B3GALT6 transfers Gal to the tetrasaccharide linker Glycosaminoglycan metabolism R-HSA-1630316 30203 glycosaminoglycan metabolic process frameshift loss_of_function of glycosaminoglycan metabolic process

PS3 B3GALT6 B3GALT6 C300S [Golgi membrane] R-HSA-4420366 B3GALT6 UniProt:Q96L58 L-cysteine 300 replaced with L-serine ReplacedResidue spondyloepimetaphyseal dysplasia DOID:0080027 R-HSA-4420346-B3GALT6 mutants [Golgi membrane]-DefinedSet-hasMember R-HSA-4420365 Defective B3GALT6 does not transfer Gal to the tetrasaccharide linker loss_of_function pubmed:10506123|pubmed:23664118|pubmed:23664117 R-HSA-4420332 Defective B3GALT6 causes EDSP2 and SEMDJL1 R-HSA-1889978 B3GALT6 transfers Gal to the tetrasaccharide linker Glycosaminoglycan metabolism R-HSA-1630316 30203 glycosaminoglycan metabolic process missense loss_of_function of glycosaminoglycan metabolic process

PS3 B3GALT6 B3GALT6 D156N [Golgi membrane] R-HSA-4420367 B3GALT6 UniProt:Q96L58 L-aspartic acid 156 replaced with L-asparagine ReplacedResidue spondyloepimetaphyseal dysplasia DOID:0080027 R-HSA-4420346-B3GALT6 mutants [Golgi membrane]-DefinedSet-hasMember R-HSA-4420365 Defective B3GALT6 does not transfer Gal to the tetrasaccharide linker loss_of_function pubmed:10506123|pubmed:23664118|pubmed:23664117 R-HSA-4420332 Defective B3GALT6 causes EDSP2 and SEMDJL1 R-HSA-1889978 B3GALT6 transfers Gal to the tetrasaccharide linker Glycosaminoglycan metabolism R-HSA-1630316 30203 glycosaminoglycan metabolic process missense loss_of_function of glycosaminoglycan metabolic process

PS3 B3GALT6 B3GALT6 R232C [Golgi membrane] R-HSA-4420361 B3GALT6 UniProt:Q96L58 L-arginine 232 replaced with L-cysteine ReplacedResidue spondyloepimetaphyseal dysplasia DOID:0080027 R-HSA-4420346-B3GALT6 mutants [Golgi membrane]-DefinedSet-hasMember R-HSA-4420365 Defective B3GALT6 does not transfer Gal to the tetrasaccharide linker loss_of_function pubmed:10506123|pubmed:23664118|pubmed:23664117 R-HSA-4420332 Defective B3GALT6 causes EDSP2 and SEMDJL1 R-HSA-1889978 B3GALT6 transfers Gal to the tetrasaccharide linker Glycosaminoglycan metabolism R-HSA-1630316 30203 glycosaminoglycan metabolic process missense loss_of_function of glycosaminoglycan metabolic process

PS3 B3GALT6 B3GALT6 R197Afs*81 [Golgi membrane] R-HSA-4420356 B3GALT6 UniProt:Q96L58 Replacement of residues 197 to 276 by AGARPPGNSATTTCPTRWAAATCSRPTWCTTCASAATTCAPGTARTCLWAPGWRRWTSSGSTTRASTPNTGPAAAATSTW FragmentReplacedModification Ehlers-Danlos syndrome DOID:13359 R-HSA-4420346-B3GALT6 mutants [Golgi membrane]-DefinedSet-hasMember R-HSA-4420365 Defective B3GALT6 does not transfer Gal to the tetrasaccharide linker loss_of_function pubmed:10506123|pubmed:23664118|pubmed:23664117 R-HSA-4420332 Defective B3GALT6 causes EDSP2 and SEMDJL1 R-HSA-1889978 B3GALT6 transfers Gal to the tetrasaccharide linker Glycosaminoglycan metabolism R-HSA-1630316 30203 glycosaminoglycan metabolic process frameshift loss_of_function of glycosaminoglycan metabolic process

PS3 B3GALT6 B3GALT6 S65G [Golgi membrane] R-HSA-4420368 B3GALT6 UniProt:Q96L58 L-serine 65 replaced with glycine ReplacedResidue spondyloepimetaphyseal dysplasia DOID:0080027 R-HSA-4420346-B3GALT6 mutants [Golgi membrane]-DefinedSet-hasMember R-HSA-4420365 Defective B3GALT6 does not transfer Gal to the tetrasaccharide linker loss_of_function pubmed:10506123|pubmed:23664118|pubmed:23664117 R-HSA-4420332 Defective B3GALT6 causes EDSP2 and SEMDJL1 R-HSA-1889978 B3GALT6 transfers Gal to the tetrasaccharide linker Glycosaminoglycan metabolism R-HSA-1630316 30203 glycosaminoglycan metabolic process missense loss_of_function of glycosaminoglycan metabolic process
[truncated: 4,725,893 more chars]
