# Supplementary material for: Pathway-based, reaction-specific annotation of disease variants for elucidation of molecular phenotypes
Source: Database (Oxford). 2024 May 7;2024:baae031. doi: 10.1093/database/baae031 (PMC11184451; doi:10.1093/database/baae031)
Supplement: baae031_Supp [file baae031_supp.zip › suppl_data/Database_Orlic_Milacic_Reactome_of_Disease_Variants_SupplementaryTable1_Legend.docx]

Legend of Supplementary Table 1. To see the Supplementary Table 1 in the table format, save it as a plain text file and then open the file in Excel as a tab delimited file.

| **Column** | **Supplementary Table 1 Header** | **Header Description** |
| --- | --- | --- |
| A | ACMG_AMP_Criterion | Curator-appended pathogenicity classification in accordance with ACMG/AMP guidelines - not directly available in the database |
| B | Genename | Reference Gene HGNC Symbol |
| C | displayName | Disease variant EWAS name |
| D | stable_id | Reactome stable identifier for disease variant EWAS |
| E | referenceEntity_name | UniProt-derived gene symbol |
| F | referenceEntity_id | UniProt identifier |
| G | hasModifiedResidue_displayName | genetically modified residue(s) in disease variant EWAS relative to reference protein sequence |
| H | modifiedResidue_class | Reactome class of genetically modified residue |
| I | cross_reference | cross reference to external disease variant database |
| J | disease | disease term from Disease Ontology |
| K | disease_identifier | Disease Ontology identifier |
| L | entityWithAccessionedSequence_literatureReference_pubMedIdentifier | PubMed identifier(s) for papers describing the disease variant EWAS |
| M | first_entitySet | Reactome stable identifier, display name, type, and EWAS status for disease sets containing the disease variant EWAS. Only the nearest neighbor sets are included. |
| N | entityWithAccessionedSequence_reactionLikeEvent_stable_id | Reactome stable identifier for disease reaction(s) in which disease variant EWAS participates. |
| O | entityWithAccessionedSequence_reactionLikeEvent_displayName | Display name for disease reaction(s) in which disease variant EWAS participates. |
| P | entityWithAccessionedSequence_reactionLikeEvent_entityFunctionalStatus_functionalStatus_functionalStatusType_displayName | Functional status of disease variant EWAS in disease reaction(s) |
| Q | reactionLikeEvent_literatureReference_pubMedIdentifier | PubMed identifier(s) for papers describing disease reaction(s) in which disease variant EWAS participates |
| R | entityWithAccessionedSequence_pathway_stable_id | Reactome stable identifier for disease pathways(s) in which disease variant EWAS participates. Only diagram-level pathways are included. |
| S | entityWithAccessionedSequence_pathway_displayName | Display name for disease pathway(s) in which disease variant EWAS participates. Only diagram-level pathways are included. |
| T | entityWithAccessionedSequence_reactionLikeEvent_normalReaction_stable_id | Reactome stable identifier for normal reaction counterpart of disease reaction in which disease variant EWAS participates. |
| U | entityWithAccessionedSequence_reactionLikeEvent_normalReaction_displayName | Display name for normal reaction counterpart of disease reaction in which disease variant EWAS participates. |
| V | entityWithAccessionedSequence_pathway_normalPathway_displayName | Reactome stable identifier for normal pathway counterpart of disease pathway in which disease variant EWAS participates. |
| W | entityWithAccessionedSequence_pathway_normalPathway_stable_id | Display name for normal pathway counterpart of disease pathway in which disease variant EWAS participates. |
| X | normal_reaction_like_event_go_biological_process_accession | Acession number for Gene Ontology Biological Process associated with normal reaction counterpart of disease reaction in which disease variant EWAS participates |
| Y | normal_reaction_like_event_go_biological_process_displayName | Gene Ontology Biological Process term associated with normal reaction counterpart of disease reaction in which disease variant EWAS participates |
| Z | entityWithAccessionedSequence_pathway_normalPathway_goBiologicalProcess_accession | Acession number for Gene Ontology Biological Process associated with normal pathway counterpart of disease pathway in which disease variant EWAS participates |
| AA | entityWithAccessionedSequence_pathway_normalPathway_goBiologicalProcess_displayName | Gene Ontology Biological Process term associated with normal pathway counterpart of disease pathway in which disease variant EWAS participates |
| AB | Disease Variant Type | Curator-appended disease variant type - not directly available in the database |
| AC | Molecular Phenotype | Curator-appended molecular disease phenotype derived from the functional status and the Gene Ontology Biological Process for the normal pathway - not directly available in the database |
