# Supplementary material for: Pathway-based, reaction-specific annotation of disease variants for elucidation of molecular phenotypes
Source: Database (Oxford). 2024 May 7;2024:baae031. doi: 10.1093/database/baae031 (PMC11184451; doi:10.1093/database/baae031)
Supplement: baae031_Supp [file baae031_supp.zip › suppl_data/Database_Orlic_Milacic_Reactome_of_Disease_Variants_SupplementaryTable2.docx]

|  | A | B | C | D | E | F | G | H | I | J | K | L | M | N | O | P | Q | R |
| --- | --- | --- | --- | --- | --- | --- | --- | --- | --- | --- | --- | --- | --- | --- | --- | --- | --- | --- |
| 1 | **Pathway identifier** | **Pathway name** | **Front page item** | **Wild‐type pathway** | **#Entities found** | **#Entities total** | **Entities ratio** | **Entities**  **pValue** | **Entities FDR** | **#Reactions**  **found** | **#Reactions**  **total** | **Reactions ratio** | **Species**  **identifier** | **Species name** | **Submitted entities found** | **Found reaction identifiers** | **Number of Reactome disease genes found** | **Percent of Reactome disease genes** |
| 2 | R‐HSA‐1643685 | Disease | YES | NO | 397 | 2528 | 0.166612 | 1.11E‐16 | 1.33E‐14 | 908 | 1787 | 0.125881 | 9606 | Homo sapiens | RIP;TFG;FGFR1OP2;KDR;TRIM24;CYP1B1;AKT1;LRAT;MMUT;SLC12A6;SL C34A3;SLC34A2;CUBN;SLC6A19;ALG8;DAXX;ALG9;SLC6A18;ALG6;SLC34 A1;SLC35C1;SLC11A2;ALG2;ALG3;TALDO1;MOGS;ALG1;SND1;EML4;CYP 27A1;BCR;SLC5A7;NEIL3;RBP4;BTD;CYP2U1;BIN2;PRKAR1A;AKAP9;SERPI NG1;NHLRC1;HPRT1;NEIL1;TP53;CFTR;MMADHC;GNE;SLC22A5;SLC35D1  ;ABCB6;MAOA;ABCB4;ZC3HAV1;PIK3R1;SLC5A1;SLC5A2;GNS;SLC5A5;FX R1;CYP27B1;RNF213;RDH12;HLCS;NTHL1;SI;NEU1;CHST14;ATG7;ARSB;S PTBN1;SEC31A;ST3GAL3;BARD1;ABCA1;BCL11A;CBLIF;ABCA3;PMM2;SL C33A1;ABCA4;EIF2AK3;FN1;BRAF;ERLIN2;CP;EXT1;SLC6A5;EXT2;PPFIBP1  ;TCN2;PAH;GOLGB1;STRN;GSDME;CD320;HIP1;WDR48;ACY1;OGG1;HEX B;GBE1;CLTC;HEXA;BRCA1;BRCA2;SLC6A2;SLC6A3;KHK;IKBKB;MECP2;GY S2;SLC22A12;GYS1;SHH;ZMYM2;ATIC;HYAL1;OPN1LW;SLC22A18;CNTRL; IDS;IKBKG;HRAS;SFTPB;CHST6;SLC2A10;GAA;SFTPC;AGTRAP;AP3B1;CYP 7B1;APRT;F8;F9;MRAS;SLC7A7;OPN1MW;CYP21A2;SLC7A9;KIT;RDH5;AL DOB;AVP;TLR3;SLC29A3;CHST3;AMER1;KANK1;AHCY;SLC20A2;IDUA;TR AK1;ABCB11;PAPSS2;EGFR;PAPSS1;CYP17A1;NRAS;CUX1;LMNA;SLC17A5  ;PMS2;SHOC2;SLC17A8;MGAT2;RANBP2;NPM1;SLC16A1;CDKN2A;AGK;G SS;F12;FMO3;MLH1;GCK;TRIP11;CTNNB1;AGGF1;RHAG;CEP43;GALNT12  ;DPAGT1;TPMT;MPI;PPP1CB;HHAT;NAGLU;KIF5B;LARGE1;GGT1;GYG1;S LC39A4;EPM2A;ABCG8;RNF43;PDGFRA;ABCG5;MAP2K1;MAP2K2;TPM4; TPM3;FBXW7;ALG14;LMO7;ALG12;ALG11;MAT1A;TICAM1;DHDDS;DPM 1;MSH6;DPM2;DPM3;MSH2;SLC9A6;MSH3;SLCO2A1;SLC9A9;RAF1;CARS  1;SQSTM1;VCL;SLC27A4;SLC26A2;NOTCH1;FAM114A2;SLC1A1;SLC1A3;C SF2RB;CYP4F22;KLC1;CYP19A1;CSF2RA;SLCO1B1;BAG4;BCL2L11;CYP11A 1;TPR;SLCO1B3;RIPK1;STRA6;MPDU1;SMAD2;SMAD4;SMAD3;CPSF6;ID H1;SRD5A3;GFPT1;MSN;B3GALT6;DOLK;PALB2;G6PC1;QKI;SLC2A9;NFKB IA;GCLC;G6PC3;CYP11B2;CYP11B1;TBXAS1;NF1;FGFR4;SLC26A4;FGFR3;S LC26A3;FGFR2;FGFR1;GALK1;POMT2;ALK;ABCD4;GSK3B;B4GALT1;FLT3;  POMT1;SLC2A1;SLC40A1;PTEN;SLC2A2;MTR;TENT4A;OPLAH;HK1;SGSH; | HSA‐4755545;R‐HSA‐1220611;R‐HSA‐1839112;R‐HSA‐1220614;R‐HSA‐9664646;R‐HSA‐1839114;R‐ HSA‐1220612;R‐HSA‐9734274;R‐HSA‐1220613;R‐HSA‐5638209;R‐HSA‐5638222;R‐HSA‐1839110;R‐ HSA‐1169421;R‐HSA‐1839107;R‐HSA‐9035949;R‐HSA‐9035950;R‐HSA‐5683325;R‐HSA‐9724090;R‐ HSA‐9035960;R‐HSA‐9035966;R‐HSA‐2023462;R‐HSA‐9685162;R‐HSA‐2023460;R‐HSA‐9035954;R‐ HSA‐4755572;R‐HSA‐9035956;R‐HSA‐2023456;R‐HSA‐9035976;R‐HSA‐2769000;R‐HSA‐9035978;R‐ HSA‐9754839;R‐HSA‐9646295;R‐HSA‐2769007;R‐HSA‐5654544;R‐HSA‐9035983;R‐HSA‐5654545;R‐ HSA‐9035982;R‐HSA‐9670874;R‐HSA‐2768993;R‐HSA‐2768999;R‐HSA‐9629917;R‐HSA‐8876246;R‐ HSA‐9605313;R‐HSA‐9660615;R‐HSA‐9724099;R‐HSA‐8876240;R‐HSA‐8876255;R‐HSA‐9035987;R‐ HSA‐2769008;R‐HSA‐9035988;R‐HSA‐2769015;R‐HSA‐5683209;R‐HSA‐9035990;R‐HSA‐8876262;R‐ HSA‐9036008;R‐HSA‐9036011;R‐HSA‐8876258;R‐HSA‐9036012;R‐HSA‐5603379;R‐HSA‐3858506;R‐ HSA‐9036025;R‐HSA‐192627;R‐HSA‐5660706;R‐HSA‐8851710;R‐HSA‐9670888;R‐HSA‐9036021;R‐ HSA‐9036020;R‐HSA‐4225086;R‐HSA‐5679145;R‐HSA‐9703440;R‐HSA‐9703441;R‐HSA‐9703442;R‐ HSA‐5654748;R‐HSA‐5660890;R‐HSA‐9703430;R‐HSA‐9723906;R‐HSA‐9709571;R‐HSA‐9703437;R‐ HSA‐4839635;R‐HSA‐9703438;R‐HSA‐4839634;R‐HSA‐9703439;R‐HSA‐9703432;R‐HSA‐9703433;R‐ HSA‐9703434;R‐HSA‐4839638;R‐HSA‐9703435;R‐HSA‐2691211;R‐HSA‐9664567;R‐HSA‐9734193;R‐ HSA‐9652277;R‐HSA‐2691214;R‐HSA‐2691226;R‐HSA‐4827388;R‐HSA‐9709601;R‐HSA‐5660910;R‐ HSA‐6785245;R‐HSA‐2691219;R‐HSA‐6785244;R‐HSA‐9652264;R‐HSA‐5621918;R‐HSA‐5683355;R‐ HSA‐3797226;R‐HSA‐5662851;R‐HSA‐5621888;R‐HSA‐4755600;R‐HSA‐2318585;R‐HSA‐9664588;R‐ HSA‐9660538;R‐HSA‐9660536;R‐HSA‐168184;R‐HSA‐5660840;R‐HSA‐1218833;R‐HSA‐9664918;R‐ HSA‐4839746;R‐HSA‐9763200;R‐HSA‐5579084;R‐HSA‐5579081;R‐HSA‐2453818;R‐HSA‐9664950;R‐ HSA‐3322135;R‐HSA‐9701810;R‐HSA‐3322140;R‐HSA‐9658813;R‐HSA‐9664933;R‐HSA‐5661039;R‐ HSA‐9687465;R‐HSA‐9664940;R‐HSA‐3322125;R‐HSA‐9712084;R‐HSA‐9712085;R‐HSA‐9712086;R‐ HSA‐9664976;R‐HSA‐9712087;R‐HSA‐9664983;R‐HSA‐5228811;R‐HSA‐9712081;R‐HSA‐9712082;R‐ HSA‐9712083;R‐HSA‐9609689;R‐HSA‐9664991;R‐HSA‐9712088;R‐HSA‐8876497;R‐HSA‐9712078;R‐ HSA‐9712079;R‐HSA‐9636296;R‐HSA‐168285;R‐HSA‐9665009;R‐HSA‐3645780;R‐HSA‐3828061;R‐ HSA‐5228840;R‐HSA‐9005561;R‐HSA‐9036283;R‐HSA‐9036285;R‐HSA‐9681382;R‐HSA‐5687585;R‐ HSA‐9665000;R‐HSA‐3299657;R‐HSA‐4839734;R‐HSA‐8868344;R‐HSA‐9036041;R‐HSA‐2029983;R‐ HSA‐2046363;R‐HSA‐5609939;R‐HSA‐9036046;R‐HSA‐9036037;R‐HSA‐180622;R‐HSA‐5683672;R‐ HSA‐9036056;R‐HSA‐6785524;R‐HSA‐168337;R‐HSA‐9036061;R‐HSA‐5624256;R‐HSA‐9036050;R‐ | 372 | 100.00% |
| 3 | R‐HSA‐1430728 | Metabolism | YES | YES | 137 | 3644 | 0.240163 | 0.00412 | 0.021291 | 303 | 2268 | 0.159763 | 9606 | Homo sapiens | MTRR;TPMT;PPP1CB;PNP;NAGLU;AKT1;CYP1B1;LRAT;MMUT;GGT1;GYG 1;EPM2A;CUBN;TALDO1;MAT1A;CYP27A1;RBP4;BTD;CYP2U1;PRKAR1A; NHLRC1;HPRT1;MMADHC;SLC26A2;SLC22A5;SLC35D1;MAOA;ABCB4;PIK 3R1;CYP4F22;GNS;CYP19A1;SLC5A5;CYP27B1;SLCO1B1;CYP11A1;HLCS;T PR;NEU1;SLCO1B3;CHST14;ARSB;ST3GAL3;ABCA1;CBLIF;IDH1;SRD5A3;B 3GALT6;G6PC1;EXT1;EXT2;GCLC;G6PC3;TCN2;CYP11B2;PAH;CYP11B1;TB XAS1;GALK1;CD320;ABCD4;B4GALT1;ACY1;HEXB;GBE1;HEXA;SLC2A1;PT EN;SLC2A2;MTR;OPLAH;KHK;HK1;SGSH;GYS2;GYS1;CYP26B1;ATIC;HYAL 1;CYP2R1;IDS;GUSB;SLC37A4;ABCD1;PGM1;HGSNAT;CHST6;AMN;ABCC  2;UGT1A1;ABCC8;DCXR;GAA;CYP7B1;APRT;CYP26C1;PIK3CA;CYP21A2;R FT1;ALDOB;B4GALT7;CHST3;AHCY;GALT;IDUA;ABCB11;PAPSS2;PAPSS1; CYP17A1;CHSY1;UGT1A4;RANBP2;SLC16A1;MMAA;MMAB;B3GAT3;AGK  ;GSS;FMO3;GCK;RPIA;POMC;GALNS;GALE;CYP24A1;GLB1;MMACHC | 1655827;R‐HSA‐1655830;R‐HSA‐2162225;R‐HSA‐2162226;R‐HSA‐2162227;R‐HSA‐1655823;R‐HSA‐  1605632;R‐HSA‐2022919;R‐HSA‐5696074;R‐HSA‐193054;R‐HSA‐194079;R‐HSA‐3165230;R‐HSA‐  1678854;R‐HSA‐194083;R‐HSA‐9638064;R‐HSA‐212007;R‐HSA‐2090043;R‐HSA‐427555;R‐HSA‐  193060;R‐HSA‐8943279;R‐HSA‐192042;R‐HSA‐9638075;R‐HSA‐2090038;R‐HSA‐2090037;R‐HSA‐  193064;R‐HSA‐193065;R‐HSA‐193070;R‐HSA‐9638078;R‐HSA‐193068;R‐HSA‐9638076;R‐HSA‐  192051;R‐HSA‐193072;R‐HSA‐1655850;R‐HSA‐193073;R‐HSA‐192054;R‐HSA‐9650858;R‐HSA‐  1655843;R‐HSA‐1655847;R‐HSA‐1655846;R‐HSA‐2090079;R‐HSA‐192065;R‐HSA‐2408551;R‐HSA‐  73797;R‐HSA‐1605724;R‐HSA‐73798;R‐HSA‐193099;R‐HSA‐193101;R‐HSA‐9705713;R‐HSA‐  9705714;R‐HSA‐2408525;R‐HSA‐5661240;R‐HSA‐373867;R‐HSA‐2408532;R‐HSA‐2161745;R‐HSA‐  193143;R‐HSA‐373875;R‐HSA‐1630304;R‐HSA‐2408540;R‐HSA‐192123;R‐HSA‐1630306;R‐HSA‐  2090085;R‐HSA‐177784;R‐HSA‐9638127;R‐HSA‐9638125;R‐HSA‐1793182;R‐HSA‐3149494;R‐HSA‐  1638032;R‐HSA‐2105001;R‐HSA‐3322041;R‐HSA‐71306;R‐HSA‐71825;R‐HSA‐3322019;R‐HSA‐  2046093;R‐HSA‐3322016;R‐HSA‐2046087;R‐HSA‐1989765;R‐HSA‐3322025;R‐HSA‐1989762;R‐HSA‐  469659;R‐HSA‐1606789;R‐HSA‐5433074;R‐HSA‐2161792;R‐HSA‐165026;R‐HSA‐1889981;R‐HSA‐  3322003;R‐HSA‐198818;R‐HSA‐5433072;R‐HSA‐1793209;R‐HSA‐2161795;R‐HSA‐3322001;R‐HSA‐  1889978;R‐HSA‐3322005;R‐HSA‐2993802;R‐HSA‐71334;R‐HSA‐2993799;R‐HSA‐3322009;R‐HSA‐  1793207;R‐HSA‐3322014;R‐HSA‐1247935;R‐HSA‐1497784;R‐HSA‐1606312;R‐HSA‐2022063;R‐HSA‐  193713;R‐HSA‐76466;R‐HSA‐193719;R‐HSA‐2161814;R‐HSA‐1793186;R‐HSA‐2993814;R‐HSA‐  70333;R‐HSA‐1638053;R‐HSA‐1889955;R‐HSA‐139970;R‐HSA‐2161890;R‐HSA‐1497810;R‐HSA‐  70342;R‐HSA‐2160874;R‐HSA‐2161899;R‐HSA‐193737;R‐HSA‐3149563;R‐HSA‐9677917;R‐HSA‐  1497796;R‐HSA‐70355;R‐HSA‐3149539;R‐HSA‐76500;R‐HSA‐70361;R‐HSA‐70369;R‐HSA‐2025723;R‐ HSA‐3159253;R‐HSA‐453358;R‐HSA‐3159259;R‐HSA‐3204318;R‐HSA‐163568;R‐HSA‐193780;R‐HSA‐ 975608;R‐HSA‐2076392;R‐HSA‐193787;R‐HSA‐1855205;R‐HSA‐3322057;R‐HSA‐390393;R‐HSA‐  3149518;R‐HSA‐3149519;R‐HSA‐2426148;R‐HSA‐9603987;R‐HSA‐193792;R‐HSA‐2404134;R‐HSA‐  2404135;R‐HSA‐3076905;R‐HSA‐2426146;R‐HSA‐2426157;R‐HSA‐2426156;R‐HSA‐1676048;R‐HSA‐  3000103;R‐HSA‐2993447;R‐HSA‐499981;R‐HSA‐2426153;R‐HSA‐2426152;R‐HSA‐2404137;R‐HSA‐  2426155;R‐HSA‐381707;R‐HSA‐2046222;R‐HSA‐2426161;R‐HSA‐3000120;R‐HSA‐70420;R‐HSA‐  2426163;R‐HSA‐3000122;R‐HSA‐3000112;R‐HSA‐174367;R‐HSA‐5653878;R‐HSA‐174368;R‐HSA‐  1678650;R‐HSA‐199456;R‐HSA‐174374;R‐HSA‐2046265;R‐HSA‐5653873;R‐HSA‐3000074;R‐HSA‐ | 127 | 34.14% |
| 4 | R‐HSA‐162582 | Signal Transduction | YES | YES | 112 | 3028 | 0.199565 | 0.01654 | 0.066168 | 1005 | 2534 | 0.178501 | 9606 | Homo sapiens | ALK;GSK3B;B4GALT1;FLT3;CLTC;PTEN;MTR;IKBKB;PPP1CB;SHH;HHAT;CY P26B1;PNP;OPN1LW;KIF5B;KDR;AKT1;IKBKG;HRAS;PGM1;ABCG8;RNF43; PDGFRA;ABCG5;MAP2K1;MAP2K2;TPM4;TPM3;FBXW7;AXIN1;ALG3;TGF BR1;TGFBR2;BCR;BTD;MRAS;CYP26C1;OPN1MW;PIK3CA;PRKAR1A;ESRP 1;KIT;RDH5;MYH9;AVP;RAF1;TP53;SQSTM1;CFTR;VCL;AMER1;KANK1;N OTCH1;LRP5;RRBP1;CSF2RB;PIK3R1;KLC1;CBL;TRAK1;CSF2RA;EGFR;NRA S;BAG4;BCL2L11;ERBB2;MAPK1;SHOC2;RIPK1;SPTBN1;ST3GAL3;RANBP2  ;ABCA1;SMAD2;TCF7L2;SMAD4;SMAD3;GALNT3;FN1;BRAF;POMC;NFKBI  A;OPN1SW;APC;NF1;CTNNB1;KRAS;STRN;FGFR4;FGFR3;FGFR2;FGFR1 | HSA‐9793680;R‐HSA‐2187303;R‐HSA‐5654608;R‐HSA‐1562641;R‐HSA‐1562640;R‐HSA‐419841;R‐ HSA‐5654622;R‐HSA‐5654623;R‐HSA‐5654620;R‐HSA‐2187309;R‐HSA‐5654618;R‐HSA‐178189;R‐ HSA‐5654596;R‐HSA‐5668932;R‐HSA‐5654597;R‐HSA‐5654594;R‐HSA‐9021596;R‐HSA‐5654592;R‐ HSA‐9701507;R‐HSA‐5654607;R‐HSA‐9017488;R‐HSA‐9793679;R‐HSA‐5654605;R‐HSA‐2187325;R‐ HSA‐5654603;R‐HSA‐5654600;R‐HSA‐9660557;R‐HSA‐6803527;R‐HSA‐5654646;R‐HSA‐9021609;R‐ HSA‐5654647;R‐HSA‐8851619;R‐HSA‐5668978;R‐HSA‐5654643;R‐HSA‐5654640;R‐HSA‐5654641;R‐ HSA‐870437;R‐HSA‐9021601;R‐HSA‐178218;R‐HSA‐5654655;R‐HSA‐9021600;R‐HSA‐5654653;R‐HSA‐ 8943780;R‐HSA‐5654651;R‐HSA‐5654631;R‐HSA‐5654628;R‐HSA‐9021627;R‐HSA‐9013437;R‐HSA‐  3772430;R‐HSA‐5654625;R‐HSA‐5654637;R‐HSA‐5654634;R‐HSA‐870449;R‐HSA‐2187293;R‐HSA‐  5654633;R‐HSA‐870477;R‐HSA‐8943817;R‐HSA‐5672980;R‐HSA‐870479;R‐HSA‐5672978;R‐HSA‐  198731;R‐HSA‐2187368;R‐HSA‐8943811;R‐HSA‐2187375;R‐HSA‐5138433;R‐HSA‐5672966;R‐HSA‐  5672965;R‐HSA‐5138459;R‐HSA‐9021660;R‐HSA‐5672960;R‐HSA‐5672961;R‐HSA‐2187382;R‐HSA‐  198746;R‐HSA‐8980691;R‐HSA‐5672972;R‐HSA‐5672973;R‐HSA‐9603279;R‐HSA‐2187388;R‐HSA‐  5672969;R‐HSA‐5654582;R‐HSA‐2187330;R‐HSA‐5654578;R‐HSA‐198756;R‐HSA‐9654523;R‐HSA‐  5654591;R‐HSA‐9654521;R‐HSA‐5654586;R‐HSA‐5654587;R‐HSA‐5654584;R‐HSA‐9654525;R‐HSA‐  5654566;R‐HSA‐5654565;R‐HSA‐5654562;R‐HSA‐5654560;R‐HSA‐5654575;R‐HSA‐2187355;R‐HSA‐  5654573;R‐HSA‐5654571;R‐HSA‐5654569;R‐HSA‐2187358;R‐HSA‐5357776;R‐HSA‐9021451;R‐HSA‐  9021450;R‐HSA‐5357780;R‐HSA‐870538;R‐HSA‐5675206;R‐HSA‐204947;R‐HSA‐5654734;R‐HSA‐  4395227;R‐HSA‐5654730;R‐HSA‐209055;R‐HSA‐4395231;R‐HSA‐5654729;R‐HSA‐379044;R‐HSA‐  2187395;R‐HSA‐8941613;R‐HSA‐8945709;R‐HSA‐9652277;R‐HSA‐4395236;R‐HSA‐2187401;R‐HSA‐  2187405;R‐HSA‐379048;R‐HSA‐2316434;R‐HSA‐8941628;R‐HSA‐8941618;R‐HSA‐8863804;R‐HSA‐  9652264;R‐HSA‐8941623;R‐HSA‐5654679;R‐HSA‐5654677;R‐HSA‐3299569;R‐HSA‐5654672;R‐HSA‐  5654673;R‐HSA‐5654684;R‐HSA‐5654662;R‐HSA‐5654663;R‐HSA‐205008;R‐HSA‐5654658;R‐HSA‐  5654659;R‐HSA‐8874079;R‐HSA‐5654669;R‐HSA‐9691215;R‐HSA‐5654667;R‐HSA‐9658445;R‐HSA‐  5654664;R‐HSA‐9027670;R‐HSA‐5654709;R‐HSA‐9701488;R‐HSA‐157926;R‐HSA‐8982637;R‐HSA‐  5654705;R‐HSA‐5675198;R‐HSA‐5654717;R‐HSA‐2179274;R‐HSA‐5654714;R‐HSA‐5675194;R‐HSA‐  5357757;R‐HSA‐2179276;R‐HSA‐9693282;R‐HSA‐5654692;R‐HSA‐5654690;R‐HSA‐2169046;R‐HSA‐  9013361;R‐HSA‐5634221;R‐HSA‐8943728;R‐HSA‐9701485;R‐HSA‐8982640;R‐HSA‐2179291;R‐HSA‐  5654701;R‐HSA‐2169050;R‐HSA‐2179293;R‐HSA‐5654697;R‐HSA‐5357904;R‐HSA‐2160931;R‐HSA‐ | 92 | 24.73% |
| 5 | R‐HSA‐392499 | Metabolism of proteins | YES | YES | 94 | 2214 | 0.145917 | 6.14E‐04 | 0.004911 | 144 | 812 | 0.057199 | 9606 | Homo sapiens | GALNT12;POMT2;DPAGT1;B4GALT1;WDR48;POMT1;MPI;PTEN;BRCA1;C 1GALT1C1;TFG;KIF5B;IKBKG;LARGE1;SLC34A2;SFTPB;ALG8;DAXX;ALG9;A LG6;FBXW7;SLC34A1;SFTPC;GAA;SLC35C1;ALG2;AXIN1;ALG14;ALG3;MO GS;LMO7;ALG12;ALG1;ALG11;POMGNT1;TGFBR1;TGFBR2;DHDDS;DPM 1;EEF1G;DPM2;F8;DPM3;F9;TRAF3;RFT1;MAN1B1;CARS1;TP53;SQSTM1  ;CFTR;GNE;AMER1;SFTPA2;DCTN1;CSF2RB;CSF2RA;NEU1;TPR;SLC17A5; RIPK1;MGAT2;ARSB;SPTBN1;ST3GAL3;MPDU1;SEC31A;RANBP2;SMAD2; BARD1;TCF7L2;SLC35A1;NPM1;SMAD4;SMAD3;GALNT3;CDKN2A;ABCA3  ;PMM2;SRD5A3;GFPT1;FN1;DOLK;PALB2;CP;POMC;NFKBIA;APC;GLB1;G  OLGB1;CTNNB1 | R‐HSA‐8956040;R‐HSA‐446215;R‐HSA‐5686359;R‐HSA‐1614362;R‐HSA‐688136;R‐HSA‐446212;R‐ HSA‐446208;R‐HSA‐913675;R‐HSA‐5689950;R‐HSA‐5696605;R‐HSA‐446218;R‐HSA‐446216;R‐HSA‐ 6791016;R‐HSA‐8955289;R‐HSA‐4086059;R‐HSA‐4085028;R‐HSA‐3640872;R‐HSA‐975919;R‐HSA‐  6807877;R‐HSA‐4085021;R‐HSA‐6781764;R‐HSA‐5689973;R‐HSA‐9668023;R‐HSA‐6807875;R‐HSA‐  5696627;R‐HSA‐5684862;R‐HSA‐870437;R‐HSA‐5683836;R‐HSA‐428585;R‐HSA‐3000348;R‐HSA‐  159796;R‐HSA‐4570493;R‐HSA‐159803;R‐HSA‐4084999;R‐HSA‐5615637;R‐HSA‐9638097;R‐HSA‐  870479;R‐HSA‐390470;R‐HSA‐5691411;R‐HSA‐9624789;R‐HSA‐4568846;R‐HSA‐5686301;R‐HSA‐  532549;R‐HSA‐265301;R‐HSA‐5683714;R‐HSA‐4656914;R‐HSA‐5686286;R‐HSA‐9638090;R‐HSA‐  2997616;R‐HSA‐8873946;R‐HSA‐159836;R‐HSA‐381799;R‐HSA‐3000399;R‐HSA‐5685296;R‐HSA‐  5689649;R‐HSA‐8952289;R‐HSA‐5686335;R‐HSA‐5694527;R‐HSA‐5694522;R‐HSA‐8932327;R‐HSA‐  4615987;R‐HSA‐4086088;R‐HSA‐3000411;R‐HSA‐5690159;R‐HSA‐9638120;R‐HSA‐5694418;R‐HSA‐  4551616;R‐HSA‐4615872;R‐HSA‐5694417;R‐HSA‐5685201;R‐HSA‐5694431;R‐HSA‐5690843;R‐HSA‐  427656;R‐HSA‐5685208;R‐HSA‐5228508;R‐HSA‐4755411;R‐HSA‐5690827;R‐HSA‐5694409;R‐HSA‐  8956200;R‐HSA‐901036;R‐HSA‐8952618;R‐HSA‐901039;R‐HSA‐5228525;R‐HSA‐5687284;R‐HSA‐  162721;R‐HSA‐8952620;R‐HSA‐5228523;R‐HSA‐4551649;R‐HSA‐162730;R‐HSA‐901024;R‐HSA‐  6781897;R‐HSA‐449718;R‐HSA‐5694439;R‐HSA‐936381;R‐HSA‐449715;R‐HSA‐6781905;R‐HSA‐  5694446;R‐HSA‐4551679;R‐HSA‐4655355;R‐HSA‐5690856;R‐HSA‐5694441;R‐HSA‐742345;R‐HSA‐  1964505;R‐HSA‐203973;R‐HSA‐4084984;R‐HSA‐5685649;R‐HSA‐4419979;R‐HSA‐6809006;R‐HSA‐  4419978;R‐HSA‐6809003;R‐HSA‐265160;R‐HSA‐532678;R‐HSA‐5684868;R‐HSA‐6809011;R‐HSA‐  6809010;R‐HSA‐4085992;R‐HSA‐5683840;R‐HSA‐5684864;R‐HSA‐5684865;R‐HSA‐5617037;R‐HSA‐  5685902;R‐HSA‐975829;R‐HSA‐901074;R‐HSA‐2997709;R‐HSA‐8955241;R‐HSA‐2997706;R‐HSA‐  446191;R‐HSA‐446188;R‐HSA‐379887;R‐HSA‐446189;R‐HSA‐204008;R‐HSA‐156910;R‐HSA‐446187;R‐ HSA‐446198;R‐HSA‐5683879;R‐HSA‐156913;R‐HSA‐727807;R‐HSA‐446195;R‐HSA‐981497;R‐HSA‐ 446207;R‐HSA‐2179291;R‐HSA‐446202;R‐HSA‐6782106;R‐HSA‐446201 | 91 | 24.46% |
| 6 | R‐HSA‐168256 | Immune System | YES | YES | 82 | 2624 | 0.172939 | 0.44198 | 0.441975 | 302 | 1659 | 0.116864 | 9606 | Homo sapiens | B4GALT1;FLT3;HEXB;CLTC;HEXA;PTEN;IKBKB;PNP;KIF5B;AKT1;IKBKG;LRR FIP1;GUSB;HRAS;GYG1;PGM1;HGSNAT;MAP2K1;FBXW7;GAA;TALDO1;L MO7;TICAM1;APRT;PPM1B;PIK3CA;BIN2;TRAF3;SERPING1;MYH9;RAF1; TP53;SQSTM1;TLR3;VCL;SFTPA2;MAOA;DCTN1;UNC93B1;CSF2RB;PIK3R 1;KLC1;CBL;CSF2RA;GNS;NRAS;BCL2L11;RNF213;NEU1;TPR;MAPK1;RIPK  1;ATG7;ARSB;SEC31A;RANBP2;SMAD3;CBLIF;IDH1;FN1;MSN;GALNS;PO  MC;NFKBIA;GLB1;CTNNB1;KRAS;GSDME | HSA‐9013895;R‐HSA‐9604738;R‐HSA‐8863895;R‐HSA‐9606784;R‐HSA‐936475;R‐HSA‐2130486;R‐ HSA‐203797;R‐HSA‐9607301;R‐HSA‐936985;R‐HSA‐5668417;R‐HSA‐177690;R‐HSA‐9605259;R‐HSA‐ 9607304;R‐HSA‐450074;R‐HSA‐177694;R‐HSA‐913424;R‐HSA‐177692;R‐HSA‐2317314;R‐HSA‐  389159;R‐HSA‐389158;R‐HSA‐2076220;R‐HSA‐2562564;R‐HSA‐9705137;R‐HSA‐913451;R‐HSA‐  741411;R‐HSA‐5668454;R‐HSA‐2317332;R‐HSA‐8951498;R‐HSA‐9013963;R‐HSA‐937032;R‐HSA‐  9750226;R‐HSA‐5432852;R‐HSA‐451649;R‐HSA‐198731;R‐HSA‐937029;R‐HSA‐921155;R‐HSA‐  6800426;R‐HSA‐9733316;R‐HSA‐1678921;R‐HSA‐9013979;R‐HSA‐9013978;R‐HSA‐6800434;R‐HSA‐  1678927;R‐HSA‐8854736;R‐HSA‐198746;R‐HSA‐9630923;R‐HSA‐1168459;R‐HSA‐9733323;R‐HSA‐  9013974;R‐HSA‐5676598;R‐HSA‐9013992;R‐HSA‐9699574;R‐HSA‐1112690;R‐HSA‐5676596;R‐HSA‐  5676597;R‐HSA‐983147;R‐HSA‐5684275;R‐HSA‐5676595;R‐HSA‐198756;R‐HSA‐5676593;R‐HSA‐  9013985;R‐HSA‐5668414;R‐HSA‐983140;R‐HSA‐9013986;R‐HSA‐9699578;R‐HSA‐9699579;R‐HSA‐  9013990;R‐HSA‐9606887;R‐HSA‐168053;R‐HSA‐9008894;R‐HSA‐2559568;R‐HSA‐389756;R‐HSA‐  914036;R‐HSA‐983157;R‐HSA‐983156;R‐HSA‐198270;R‐HSA‐5684267;R‐HSA‐937075;R‐HSA‐  983153;R‐HSA‐9773803;R‐HSA‐202365;R‐HSA‐983152;R‐HSA‐9009910;R‐HSA‐912527;R‐HSA‐  6786789;R‐HSA‐9606160;R‐HSA‐2424480;R‐HSA‐2424482;R‐HSA‐392835;R‐HSA‐8852481;R‐HSA‐  1295516;R‐HSA‐1678998;R‐HSA‐9680385;R‐HSA‐933532;R‐HSA‐933530;R‐HSA‐5609665;R‐HSA‐  9749505;R‐HSA‐9680389;R‐HSA‐9680388;R‐HSA‐933526;R‐HSA‐2130619;R‐HSA‐166041;R‐HSA‐  933523;R‐HSA‐168092;R‐HSA‐209063;R‐HSA‐209061;R‐HSA‐9609274;R‐HSA‐9607224;R‐HSA‐  933539;R‐HSA‐2213017;R‐HSA‐2424477;R‐HSA‐9014320;R‐HSA‐390329;R‐HSA‐209087;R‐HSA‐  9008692;R‐HSA‐9645137;R‐HSA‐9695831;R‐HSA‐9695828;R‐HSA‐2029273;R‐HSA‐2029271;R‐HSA‐  168140;R‐HSA‐9014343;R‐HSA‐9014342;R‐HSA‐847070;R‐HSA‐9674816;R‐HSA‐9761344;R‐HSA‐  9645126;R‐HSA‐450259;R‐HSA‐918232;R‐HSA‐8950367;R‐HSA‐5668481;R‐HSA‐388830;R‐HSA‐  2201338;R‐HSA‐918229;R‐HSA‐9645134;R‐HSA‐918227;R‐HSA‐918225;R‐HSA‐5668534;R‐HSA‐  388832;R‐HSA‐209125;R‐HSA‐5668543;R‐HSA‐1168637;R‐HSA‐1168636;R‐HSA‐1168638;R‐HSA‐  2130640;R‐HSA‐2130641;R‐HSA‐5624486;R‐HSA‐912629;R‐HSA‐5624492;R‐HSA‐168184;R‐HSA‐  9698408;R‐HSA‐5624494;R‐HSA‐912627;R‐HSA‐6785895;R‐HSA‐975119;R‐HSA‐2130725;R‐HSA‐  202500;R‐HSA‐914182;R‐HSA‐2130731;R‐HSA‐879362;R‐HSA‐5621573;R‐HSA‐1168641;R‐HSA‐  1168640;R‐HSA‐1168643;R‐HSA‐450325;R‐HSA‐1168642;R‐HSA‐199959;R‐HSA‐5362500;R‐HSA‐  9758604;R‐HSA‐166175;R‐HSA‐879917;R‐HSA‐199456;R‐HSA‐202534;R‐HSA‐450337;R‐HSA‐ | 68 | 18.28% |
| 7 | R‐HSA‐382551 | Transport of small molecules | YES | YES | 81 | 969 | 0.063863 | 3.33E‐16 | 3.53E‐14 | 110 | 454 | 0.031981 | 9606 | Homo sapiens | CLTC;SLC2A1;SLC40A1;SLC2A2;SLC4A1;ABCA12;SLC6A2;SLC4A4;SLC6A3; SLC22A12;SLC22A18;SLC12A6;ABCD1;SLC39A4;SLC34A3;SLC34A2;CUBN; ABCG8;SLC6A19;AMN;ABCG5;SLC36A2;SLC6A18;ABCC2;SLC2A10;SLC34 A1;SLC35C1;SLC11A2;ABCC6;ABCC9;SLC5A7;SLC7A7;SLC9A6;SLC7A9;PRK AR1A;SLC9A9;SLCO2A1;AVP;RAF1;CFTR;SLC29A3;SLC27A4;SLC24A1;SLC2 6A2;SLC24A4;SLC22A5;SLC20A2;SLC35D1;ABCB6;ABCB4;SLC1A1;SLC3A1; SLC1A3;SLC5A1;SLC5A2;SLC5A5;SLCO1B1;SLCO1B3;SLC17A5;RIPK1;SLC1 7A8;ABCA1;SLC35A2;SLC12A3;SLC35A1;SLC35A3;SLC16A1;ABCA3;ABCA  4;SLC33A1;SLC12A1;ERLIN2;CP;SLC6A5;SLC2A9;CLCN6;RHAG;SLC26A4;SL  C26A3;SLC24A5 | R‐HSA‐9728150;R‐HSA‐379393;R‐HSA‐444416;R‐HSA‐442368;R‐HSA‐425482;R‐HSA‐429591;R‐HSA‐ 428052;R‐HSA‐379415;R‐HSA‐189208;R‐HSA‐429594;R‐HSA‐194079;R‐HSA‐429094;R‐HSA‐194083;R‐ HSA‐174368;R‐HSA‐5678706;R‐HSA‐427555;R‐HSA‐8951727;R‐HSA‐450095;R‐HSA‐450092;R‐HSA‐ 9701055;R‐HSA‐429613;R‐HSA‐379432;R‐HSA‐450088;R‐HSA‐428585;R‐HSA‐442422;R‐HSA‐  265783;R‐HSA‐111925;R‐HSA‐9645220;R‐HSA‐432188;R‐HSA‐5682285;R‐HSA‐1237038;R‐HSA‐  741450;R‐HSA‐392263;R‐HSA‐432195;R‐HSA‐1454916;R‐HSA‐427605;R‐HSA‐5683714;R‐HSA‐  1467457;R‐HSA‐5678863;R‐HSA‐443997;R‐HSA‐1467466;R‐HSA‐1237069;R‐HSA‐426086;R‐HSA‐  9631987;R‐HSA‐375405;R‐HSA‐8866542;R‐HSA‐3295579;R‐HSA‐382575;R‐HSA‐561253;R‐HSA‐  8866551;R‐HSA‐904830;R‐HSA‐8866546;R‐HSA‐597628;R‐HSA‐427645;R‐HSA‐8866553;R‐HSA‐  9701141;R‐HSA‐6784738;R‐HSA‐264834;R‐HSA‐917891;R‐HSA‐1247645;R‐HSA‐427656;R‐HSA‐  216723;R‐HSA‐382613;R‐HSA‐435349;R‐HSA‐216727;R‐HSA‐264848;R‐HSA‐426130;R‐HSA‐427666;R‐ HSA‐5626316;R‐HSA‐9725706;R‐HSA‐1247665;R‐HSA‐917933;R‐HSA‐8866851;R‐HSA‐879528;R‐HSA‐ 426155;R‐HSA‐375473;R‐HSA‐216757;R‐HSA‐6784735;R‐HSA‐549297;R‐HSA‐6784729;R‐HSA‐  742345;R‐HSA‐727759;R‐HSA‐2682349;R‐HSA‐425678;R‐HSA‐727749;R‐HSA‐5627802;R‐HSA‐  8981574;R‐HSA‐8878664;R‐HSA‐383190;R‐HSA‐5339524;R‐HSA‐879575;R‐HSA‐8855131;R‐HSA‐  727767;R‐HSA‐8855130;R‐HSA‐735702;R‐HSA‐351963;R‐HSA‐444120;R‐HSA‐5682103;R‐HSA‐  5682101;R‐HSA‐5678261;R‐HSA‐428015;R‐HSA‐429036;R‐HSA‐425965;R‐HSA‐879585;R‐HSA‐  879584;R‐HSA‐2730692;R‐HSA‐5682084;R‐HSA‐727807;R‐HSA‐425983;R‐HSA‐1369065 | 80 | 21.51% |
| 8 | R‐HSA‐74160 | Gene expression (Transcription) | YES | YES | 63 | 1917 | 0.126343 | 0.29596 | 0.295962 | 392 | 1090 | 0.076782 | 9606 | Homo sapiens | RB1;GSK3B;NOTCH1;PTEN;BRCA1;EGFR;MECP2;BCL2L11;FIP1L1;ERBB2;T PR;PMS2;MAPK1;AKT1;RANBP2;SMAD2;BARD1;TCF7L2;DAXX;NPM1;SM AD4;SMAD3;CPSF6;CDKN2A;FBXW7;GAA;AXIN1;MLH1;G6PC1;GCK;POM C;BTD;MSH2;KIT;CTNNB1;KRAS;TP53 | HSA‐6791285;R‐HSA‐6805620;R‐HSA‐8878237;R‐HSA‐2187325;R‐HSA‐6791291;R‐HSA‐6799431;R‐ HSA‐8951977;R‐HSA‐9615536;R‐HSA‐8878243;R‐HSA‐9022625;R‐HSA‐870437;R‐HSA‐69685;R‐HSA‐ 6799441;R‐HSA‐870449;R‐HSA‐2187293;R‐HSA‐870477;R‐HSA‐9615570;R‐HSA‐9615571;R‐HSA‐  6798374;R‐HSA‐870479;R‐HSA‐9615554;R‐HSA‐6799409;R‐HSA‐3239014;R‐HSA‐2187388;R‐HSA‐  6799418;R‐HSA‐6799416;R‐HSA‐6801415;R‐HSA‐2187330;R‐HSA‐9018594;R‐HSA‐6800396;R‐HSA‐  6801637;R‐HSA‐6805730;R‐HSA‐870538;R‐HSA‐6805740;R‐HSA‐6801641;R‐HSA‐8985627;R‐HSA‐  6791409;R‐HSA‐209055;R‐HSA‐6805755;R‐HSA‐9622579;R‐HSA‐2187395;R‐HSA‐9006122;R‐HSA‐  6791363;R‐HSA‐8985644;R‐HSA‐9009208;R‐HSA‐9731111;R‐HSA‐9023549;R‐HSA‐8878143;R‐HSA‐  9023538;R‐HSA‐6791387;R‐HSA‐9622572;R‐HSA‐3700984;R‐HSA‐6814885;R‐HSA‐9733207;R‐HSA‐  9733203;R‐HSA‐3700981;R‐HSA‐9625693;R‐HSA‐9020504;R‐HSA‐6791349;R‐HSA‐6791348;R‐HSA‐  9622604;R‐HSA‐9023592;R‐HSA‐6805638;R‐HSA‐6791302;R‐HSA‐8878178;R‐HSA‐9020513;R‐HSA‐  9017441;R‐HSA‐2179274;R‐HSA‐9733247;R‐HSA‐2179276;R‐HSA‐8878186;R‐HSA‐6791306;R‐HSA‐  9017447;R‐HSA‐6805640;R‐HSA‐2106579;R‐HSA‐9733216;R‐HSA‐6805650;R‐HSA‐6791327;R‐HSA‐  2106586;R‐HSA‐6791323;R‐HSA‐2106591;R‐HSA‐5693609;R‐HSA‐6796647;R‐HSA‐6796649;R‐HSA‐  8853911;R‐HSA‐9022872;R‐HSA‐9021851;R‐HSA‐9006490;R‐HSA‐8853915;R‐HSA‐9021847;R‐HSA‐  9022870;R‐HSA‐8952232;R‐HSA‐9006507;R‐HSA‐9006508;R‐HSA‐8853921;R‐HSA‐8853920;R‐HSA‐  8952226;R‐HSA‐9615801;R‐HSA‐9006503;R‐HSA‐8939963;R‐HSA‐3700992;R‐HSA‐9617838;R‐HSA‐  3700997;R‐HSA‐6803801;R‐HSA‐6797606;R‐HSA‐8853956;R‐HSA‐9615829;R‐HSA‐9021888;R‐HSA‐  8853965;R‐HSA‐6792491;R‐HSA‐2186607;R‐HSA‐5629189;R‐HSA‐9022941;R‐HSA‐1484099;R‐HSA‐  9021919;R‐HSA‐5629187;R‐HSA‐9022942;R‐HSA‐6797616;R‐HSA‐5633295;R‐HSA‐9022935;R‐HSA‐  9022934;R‐HSA‐6803719;R‐HSA‐9021935;R‐HSA‐6801675;R‐HSA‐9006585;R‐HSA‐9021945;R‐HSA‐  6798615;R‐HSA‐9021944;R‐HSA‐9021946;R‐HSA‐9021949;R‐HSA‐6798611;R‐HSA‐9021948;R‐HSA‐  9006588;R‐HSA‐9021951;R‐HSA‐9021950;R‐HSA‐5633314;R‐HSA‐4396347;R‐HSA‐9007605;R‐HSA‐  6804762;R‐HSA‐6805785;R‐HSA‐9007606;R‐HSA‐9022728;R‐HSA‐2318752;R‐HSA‐9022734;R‐HSA‐  212356;R‐HSA‐6803946;R‐HSA‐8952081;R‐HSA‐6799815;R‐HSA‐6797766;R‐HSA‐6800836;R‐HSA‐  6797763;R‐HSA‐9022764;R‐HSA‐6803917;R‐HSA‐173481;R‐HSA‐6803914;R‐HSA‐8952101;R‐HSA‐  8986937;R‐HSA‐173488;R‐HSA‐8986939;R‐HSA‐2186643;R‐HSA‐6793685;R‐HSA‐8986940;R‐HSA‐  8986943;R‐HSA‐6803935;R‐HSA‐2127257;R‐HSA‐9022770;R‐HSA‐6799777;R‐HSA‐3222006;R‐HSA‐  8952128;R‐HSA‐2176491;R‐HSA‐9008475;R‐HSA‐2321904;R‐HSA‐5633414;R‐HSA‐2186741;R‐HSA‐ | 37 | 9.95% |

|  | A | B | C | D | E | F | G | H | I | J | K | L | M | N | O | P | Q | R |
| --- | --- | --- | --- | --- | --- | --- | --- | --- | --- | --- | --- | --- | --- | --- | --- | --- | --- | --- |
| 9 | R‐HSA‐8953897 | Cellular responses to stimuli | YES | YES | 37 | 1025 | 0.067554 | 0.17262 | 0.172615 | 73 | 481 | 0.033883 | 9606 | Homo sapiens | AMER1;RB1;GSK3B;NOTCH1;DCTN1;BRCA1;BAG4;TPR;LMNA;MAPK1;AK T1;SEC31A;RANBP2;CDKN2A;IDH1;GAA;GFPT1;EIF2AK3;TALDO1;MSN;P ALB2;GCLC;BTD;SQSTM1;TP53 | R‐HSA‐9766677;R‐HSA‐1791128;R‐HSA‐8979082;R‐HSA‐1791133;R‐HSA‐9766687;R‐HSA‐9761822;R‐ HSA‐3857329;R‐HSA‐3209151;R‐HSA‐3229089;R‐HSA‐9766532;R‐HSA‐3223200;R‐HSA‐4793819;R‐ HSA‐450325;R‐HSA‐9766656;R‐HSA‐3371435;R‐HSA‐5252041;R‐HSA‐381087;R‐HSA‐381086;R‐HSA‐ 3229102;R‐HSA‐9761844;R‐HSA‐9762100;R‐HSA‐9759157;R‐HSA‐9762102;R‐HSA‐9759158;R‐HSA‐  3209109;R‐HSA‐9759154;R‐HSA‐3209111;R‐HSA‐3200023;R‐HSA‐3209114;R‐HSA‐3786258;R‐HSA‐  381111;R‐HSA‐9761830;R‐HSA‐3209096;R‐HSA‐9761836;R‐HSA‐3371531;R‐HSA‐9762094;R‐HSA‐  3225867;R‐HSA‐3209098;R‐HSA‐5252079;R‐HSA‐9759147;R‐HSA‐182594;R‐HSA‐9760081;R‐HSA‐  3225851;R‐HSA‐9759172;R‐HSA‐9759169;R‐HSA‐9761858;R‐HSA‐3240295;R‐HSA‐3239014;R‐HSA‐  198746;R‐HSA‐8932309;R‐HSA‐9766645;R‐HSA‐6804998;R‐HSA‐6804996;R‐HSA‐3229138;R‐HSA‐  6804879;R‐HSA‐3209177;R‐HSA‐5333051;R‐HSA‐1791088;R‐HSA‐4647594;R‐HSA‐9760122;R‐HSA‐  3132737;R‐HSA‐8932327;R‐HSA‐1791092;R‐HSA‐4647593;R‐HSA‐3222593;R‐HSA‐5618080;R‐HSA‐  3223236;R‐HSA‐9760099;R‐HSA‐3209160;R‐HSA‐9761900;R‐HSA‐9645672;R‐HSA‐4647613;R‐HSA‐  5693609 | 25 | 6.72% |
| 10 | R‐HSA‐1266738 | Developmental Biology | YES | YES | 32 | 1313 | 0.086535 | 0.92918 | 0.929182 | 91 | 607 | 0.042759 | 9606 | Homo sapiens | GSK3B;NOTCH1;CLTC;SLC2A2;PIK3R1;EGFR;NRAS;SHH;ERBB2;MAPK1;AK T1;HRAS;SPTBN1;SMAD2;TCF7L2;MAP2K1;SMAD4;MAP2K2;SMAD3;ALG 3;MSN;GCK;PIK3CA;CTNNB1;MYH9;KRAS;FGFR1 | R‐HSA‐9756948;R‐HSA‐9756439;R‐HSA‐443779;R‐HSA‐452353;R‐HSA‐1535903;R‐HSA‐445071;R‐ HSA‐445069;R‐HSA‐374677;R‐HSA‐445079;R‐HSA‐211346;R‐HSA‐1980047;R‐HSA‐445077;R‐HSA‐ 3928616;R‐HSA‐9793921;R‐HSA‐9796225;R‐HSA‐211476;R‐HSA‐374680;R‐HSA‐9759882;R‐HSA‐  9756724;R‐HSA‐4093339;R‐HSA‐109860;R‐HSA‐109862;R‐HSA‐445089;R‐HSA‐392749;R‐HSA‐  392748;R‐HSA‐9793852;R‐HSA‐392751;R‐HSA‐374696;R‐HSA‐4093331;R‐HSA‐1225919;R‐HSA‐  452392;R‐HSA‐376117;R‐HSA‐555065;R‐HSA‐448951;R‐HSA‐9758501;R‐HSA‐1112609;R‐HSA‐  376119;R‐HSA‐173488;R‐HSA‐449200;R‐HSA‐448958;R‐HSA‐1181355;R‐HSA‐391868;R‐HSA‐  391871;R‐HSA‐448957;R‐HSA‐376121;R‐HSA‐391865;R‐HSA‐391866;R‐HSA‐9823959;R‐HSA‐  9823958;R‐HSA‐9823952;R‐HSA‐399951;R‐HSA‐9823836;R‐HSA‐9793887;R‐HSA‐9793375;R‐HSA‐  416588;R‐HSA‐9823966;R‐HSA‐480204;R‐HSA‐9824472;R‐HSA‐416594;R‐HSA‐9796300;R‐HSA‐  9754191;R‐HSA‐9823950;R‐HSA‐170847;R‐HSA‐211164;R‐HSA‐419033;R‐HSA‐9823989;R‐HSA‐  452838;R‐HSA‐375140;R‐HSA‐9823729;R‐HSA‐9754480;R‐HSA‐9796209;R‐HSA‐198756;R‐HSA‐  437230;R‐HSA‐3928656;R‐HSA‐452331;R‐HSA‐9793400;R‐HSA‐9823992;R‐HSA‐373739;R‐HSA‐  392053;R‐HSA‐9793383;R‐HSA‐9793382;R‐HSA‐373750;R‐HSA‐392054;R‐HSA‐3928654;R‐HSA‐  392051;R‐HSA‐9815789;R‐HSA‐419197;R‐HSA‐9823849;R‐HSA‐9823977;R‐HSA‐480378;R‐HSA‐  8854905 | 27 | 7.26% |
| 11 | R‐HSA‐1640170 | Cell Cycle | YES | YES | 25 | 733 | 0.048309 | 0.32044 | 0.32044 | 98 | 451 | 0.03177 | 9606 | Homo sapiens | RB1;BARD1;RANBP2;GSK3B;DAXX;NPM1;CDKN2A;DCTN1;GAA;ATRX;BR CA1;MLH1;BRCA2;PPP1CB;EML4;TPR;LMNA;CNTRL;AKAP9;MAPK1;AKT1; TP53;CEP43 | R‐HSA‐69891;R‐HSA‐9648017;R‐HSA‐375302;R‐HSA‐8852354;R‐HSA‐2990880;R‐HSA‐2990882;R‐ HSA‐9647746;R‐HSA‐69140;R‐HSA‐912408;R‐HSA‐69142;R‐HSA‐69144;R‐HSA‐8853405;R‐HSA‐ 188191;R‐HSA‐912429;R‐HSA‐380455;R‐HSA‐9018017;R‐HSA‐9686969;R‐HSA‐8853419;R‐HSA‐  176175;R‐HSA‐75820;R‐HSA‐187948;R‐HSA‐68914;R‐HSA‐69685;R‐HSA‐68916;R‐HSA‐6803801;R‐ HSA‐9659820;R‐HSA‐182594;R‐HSA‐5683735;R‐HSA‐9670101;R‐HSA‐606287;R‐HSA‐912458;R‐HSA‐ 9648089;R‐HSA‐912450;R‐HSA‐9686980;R‐HSA‐606289;R‐HSA‐380508;R‐HSA‐912470;R‐HSA‐  912467;R‐HSA‐113503;R‐HSA‐6803719;R‐HSA‐9648114;R‐HSA‐68960;R‐HSA‐141409;R‐HSA‐69227;R‐ HSA‐174441;R‐HSA‐141422;R‐HSA‐174445;R‐HSA‐9670114;R‐HSA‐174451;R‐HSA‐2484822;R‐HSA‐ 380272;R‐HSA‐141431;R‐HSA‐176250;R‐HSA‐912503;R‐HSA‐912496;R‐HSA‐141439;R‐HSA‐380283;R‐ HSA‐606326;R‐HSA‐380294;R‐HSA‐6799332;R‐HSA‐380303;R‐HSA‐176264;R‐HSA‐1638803;R‐HSA‐ 380311;R‐HSA‐9670149;R‐HSA‐3002811;R‐HSA‐380316;R‐HSA‐2468287;R‐HSA‐2995376;R‐HSA‐  2422927;R‐HSA‐5244669;R‐HSA‐6793685;R‐HSA‐1638821;R‐HSA‐9007926;R‐HSA‐2288097;R‐HSA‐  2467809;R‐HSA‐5229194;R‐HSA‐2467811;R‐HSA‐2993898;R‐HSA‐8941895;R‐HSA‐3000319;R‐HSA‐  198613;R‐HSA‐2574845;R‐HSA‐3000310;R‐HSA‐2172666;R‐HSA‐6803388;R‐HSA‐2574840;R‐HSA‐  188386;R‐HSA‐5633460;R‐HSA‐188390;R‐HSA‐176101;R‐HSA‐6804879;R‐HSA‐6799246;R‐HSA‐  113643;R‐HSA‐8852337;R‐HSA‐264435;R‐HSA‐8852351;R‐HSA‐5693609 | 23 | 6.18% |
| 12 | R‐HSA‐5653656 | Vesicle‐mediated transport | YES | YES | 24 | 828 | 0.054571 | 0.63929 | 0.639288 | 70 | 252 | 0.017751 | 9606 | Homo sapiens | HIP1;DCTN1;CLTC;AP3B1;GCC2;KLC1;CBL;GNS;EGFR;GOLGA4;F8;TFG;CU X1;KIF5B;GOLGB1;MYH9;TRIP11;AKT1;AVP;CFTR;SPTBN1;SEC31A | R‐HSA‐8868230;R‐HSA‐5694418;R‐HSA‐8847875;R‐HSA‐5694417;R‐HSA‐5694431;R‐HSA‐8868236;R‐ HSA‐1445144;R‐HSA‐8847635;R‐HSA‐8871196;R‐HSA‐8871194;R‐HSA‐8871193;R‐HSA‐5694409;R‐ HSA‐6807877;R‐HSA‐6807875;R‐HSA‐6814671;R‐HSA‐8867756;R‐HSA‐8868651;R‐HSA‐8867754;R‐ HSA‐8868648;R‐HSA‐5694439;R‐HSA‐8868661;R‐HSA‐8868660;R‐HSA‐196017;R‐HSA‐8868659;R‐ HSA‐8868658;R‐HSA‐190519;R‐HSA‐6814674;R‐HSA‐432688;R‐HSA‐196026;R‐HSA‐5694446;R‐HSA‐ 8869438;R‐HSA‐6814682;R‐HSA‐8847544;R‐HSA‐5694441;R‐HSA‐6811431;R‐HSA‐421831;R‐HSA‐  8849350;R‐HSA‐432706;R‐HSA‐432707;R‐HSA‐6811426;R‐HSA‐203973;R‐HSA‐6809006;R‐HSA‐  421836;R‐HSA‐5333658;R‐HSA‐6809003;R‐HSA‐421835;R‐HSA‐8933446;R‐HSA‐8849353;R‐HSA‐  432712;R‐HSA‐421833;R‐HSA‐8862280;R‐HSA‐6809011;R‐HSA‐6809010;R‐HSA‐8866269;R‐HSA‐  8866268;R‐HSA‐8868071;R‐HSA‐8866279;R‐HSA‐8866277;R‐HSA‐2316352;R‐HSA‐8874979;R‐HSA‐  5694527;R‐HSA‐8856813;R‐HSA‐204008;R‐HSA‐8866283;R‐HSA‐5694522;R‐HSA‐6814091;R‐HSA‐  8868072;R‐HSA‐190829;R‐HSA‐8856808;R‐HSA‐6811423 | 22 | 5.91% |
| 13 | R‐HSA‐109582 | Hemostasis | YES | YES | 23 | 803 | 0.052923 | 0.65963 | 0.659629 | 56 | 338 | 0.02381 | 9606 | Homo sapiens | SLC16A1;F12;FN1;PIK3R1;KLC1;NRAS;F8;SLC7A7;F9;PIK3CA;SLC7A9;PRKA R1A;KIF5B;SERPING1;MAPK1;AKT1;KRAS;RAF1;TP53;HRAS;VCL | R‐HSA‐9670673;R‐HSA‐418176;R‐HSA‐429441;R‐HSA‐443402;R‐HSA‐5607002;R‐HSA‐984733;R‐HSA‐ 140823;R‐HSA‐983194;R‐HSA‐158357;R‐HSA‐354066;R‐HSA‐354073;R‐HSA‐418200;R‐HSA‐354087;R‐ HSA‐210977;R‐HSA‐158118;R‐HSA‐377644;R‐HSA‐443439;R‐HSA‐9661625;R‐HSA‐377641;R‐HSA‐ 437162;R‐HSA‐377640;R‐HSA‐377643;R‐HSA‐443831;R‐HSA‐992696;R‐HSA‐5607023;R‐HSA‐  9650473;R‐HSA‐158137;R‐HSA‐158399;R‐HSA‐158278;R‐HSA‐354124;R‐HSA‐992708;R‐HSA‐  372693;R‐HSA‐482772;R‐HSA‐983259;R‐HSA‐5607043;R‐HSA‐158164;R‐HSA‐372697;R‐HSA‐  158300;R‐HSA‐375131;R‐HSA‐354149;R‐HSA‐202723;R‐HSA‐429415;R‐HSA‐372705;R‐HSA‐432110;R‐ HSA‐481007;R‐HSA‐418158;R‐HSA‐158313;R‐HSA‐204392;R‐HSA‐9670014;R‐HSA‐983266;R‐HSA‐ 354165;R‐HSA‐418163;R‐HSA‐437118;R‐HSA‐418170;R‐HSA‐204798;R‐HSA‐158333 | 21 | 5.65% |
| 14 | R‐HSA‐73894 | DNA Repair | YES | YES | 19 | 380 | 0.025044 | 0.02745 | 0.082361 | 165 | 339 | 0.02388 | 9606 | Homo sapiens | BARD1;WDR48;OGG1;GAA;BRCA1;MLH1;BRCA2;PALB2;MSH6;NEIL3;MS H2;NTHL1;MSH3;PMS2;NEIL1;TP53;MUTYH | R‐HSA‐69891;R‐HSA‐5684052;R‐HSA‐5655892;R‐HSA‐5656148;R‐HSA‐5653840;R‐HSA‐5656158;R‐ HSA‐5685341;R‐HSA‐5651805;R‐HSA‐5687640;R‐HSA‐5683801;R‐HSA‐9635996;R‐HSA‐5649734;R‐ HSA‐5358912;R‐HSA‐110352;R‐HSA‐9700998;R‐HSA‐110355;R‐HSA‐110354;R‐HSA‐5358919;R‐HSA‐ 5685838;R‐HSA‐5653838;R‐HSA‐110363;R‐HSA‐9701000;R‐HSA‐6786171;R‐HSA‐110364;R‐HSA‐  9701003;R‐HSA‐110368;R‐HSA‐110371;R‐HSA‐5690997;R‐HSA‐5686642;R‐HSA‐9629365;R‐HSA‐  5693564;R‐HSA‐9629369;R‐HSA‐9629372;R‐HSA‐5693561;R‐HSA‐5691001;R‐HSA‐9629373;R‐HSA‐  5693542;R‐HSA‐5684071;R‐HSA‐5690213;R‐HSA‐5693539;R‐HSA‐5651809;R‐HSA‐5690990;R‐HSA‐  5693551;R‐HSA‐5690991;R‐HSA‐5690988;R‐HSA‐9636008;R‐HSA‐9629358;R‐HSA‐5683735;R‐HSA‐  5653780;R‐HSA‐5685011;R‐HSA‐6788385;R‐HSA‐5358619;R‐HSA‐5653786;R‐HSA‐9629918;R‐HSA‐  9709273;R‐HSA‐5655835;R‐HSA‐5651992;R‐HSA‐6788392;R‐HSA‐9629149;R‐HSA‐5653766;R‐HSA‐  5649671;R‐HSA‐5358597;R‐HSA‐5358599;R‐HSA‐5649664;R‐HSA‐5653770;R‐HSA‐5649673;R‐HSA‐  6782211;R‐HSA‐6782208;R‐HSA‐5649726;R‐HSA‐5649724;R‐HSA‐5649725;R‐HSA‐5649723;R‐HSA‐  9629154;R‐HSA‐5652005;R‐HSA‐5649701;R‐HSA‐6782227;R‐HSA‐6782224;R‐HSA‐5649711;R‐HSA‐  5649708;R‐HSA‐5652009;R‐HSA‐5656105;R‐HSA‐5649705;R‐HSA‐5358545;R‐HSA‐110208;R‐HSA‐  110211;R‐HSA‐110213;R‐HSA‐5686483;R‐HSA‐110212;R‐HSA‐9629470;R‐HSA‐110224;R‐HSA‐  110227;R‐HSA‐5686469;R‐HSA‐110226;R‐HSA‐110229;R‐HSA‐5687758;R‐HSA‐9629195;R‐HSA‐  110235;R‐HSA‐6782204;R‐HSA‐5654989;R‐HSA‐5654986;R‐HSA‐110237;R‐HSA‐110236;R‐HSA‐  5654985;R‐HSA‐5649655;R‐HSA‐5358579;R‐HSA‐110243;R‐HSA‐6785732;R‐HSA‐110244;R‐HSA‐  9629492;R‐HSA‐110246;R‐HSA‐9629499;R‐HSA‐5653756;R‐HSA‐9629497;R‐HSA‐5685242;R‐HSA‐  5653754;R‐HSA‐5649658;R‐HSA‐5649657;R‐HSA‐9629216;R‐HSA‐5689317;R‐HSA‐5685985;R‐HSA‐  9629483;R‐HSA‐5685994;R‐HSA‐9707051;R‐HSA‐5684887;R‐HSA‐5693589;R‐HSA‐5684882;R‐HSA‐  5693584;R‐HSA‐5686685;R‐HSA‐5655965;R‐HSA‐5444511;R‐HSA‐5693593;R‐HSA‐5659781;R‐HSA‐  9763137;R‐HSA‐5686657;R‐HSA‐5693580;R‐HSA‐5684108;R‐HSA‐6782141;R‐HSA‐5686410;R‐HSA‐  5684875;R‐HSA‐6782138;R‐HSA‐5358513;R‐HSA‐9656947;R‐HSA‐5652151;R‐HSA‐5358512;R‐HSA‐  5693620;R‐HSA‐110307;R‐HSA‐110308;R‐HSA‐110311;R‐HSA‐5358519;R‐HSA‐5358518;R‐HSA‐  110317;R‐HSA‐5358525;R‐HSA‐110316;R‐HSA‐5655483;R‐HSA‐110319;R‐HSA‐5683385;R‐HSA‐  5655481;R‐HSA‐5685156;R‐HSA‐5444516;R‐HSA‐5444523;R‐HSA‐5655466;R‐HSA‐5693608;R‐HSA‐  5686440;R‐HSA‐5358510;R‐HSA‐5693609 | 17 | 4.57% |
| 15 | R‐HSA‐9748784 | Drug ADME | YES | YES | 14 | 239 | 0.015752 | 0.01727 | 0.069084 | 18 | 121 | 0.008524 | 9606 | Homo sapiens | SLC16A1;ABCC2;TPMT;ACY1;UGT1A1;ABCB4;SLCO1B1;PNP;SLCO1B3;UG T1A4;HPRT1;GGT1;SLC29A3 | R‐HSA‐2161538;R‐HSA‐9748951;R‐HSA‐9748983;R‐HSA‐9754929;R‐HSA‐9757010;R‐HSA‐9757139;R‐ HSA‐9748979;R‐HSA‐9759454;R‐HSA‐9753278;R‐HSA‐9753944;R‐HSA‐9755044;R‐HSA‐158546;R‐ HSA‐9759461;R‐HSA‐174931;R‐HSA‐9659680;R‐HSA‐9750656;R‐HSA‐9753634;R‐HSA‐9645220 | 13 | 3.49% |
| 16 | R‐HSA‐112316 | Neuronal System | YES | YES | 14 | 490 | 0.032294 | 0.64156 | 0.641555 | 17 | 221 | 0.015568 | 9606 | Homo sapiens | ABCC8;MAOA;SLC1A1;SLC1A3;ABCC9;SLC6A3;SLC5A7;NRAS;PPFIBP1;PR KAR1A;MAPK1;KRAS;HRAS | R‐HSA‐141186;R‐HSA‐9714580;R‐HSA‐210439;R‐HSA‐379393;R‐HSA‐379395;R‐HSA‐8951727;R‐HSA‐ 210404;R‐HSA‐442732;R‐HSA‐1296024;R‐HSA‐1369017;R‐HSA‐9677917;R‐HSA‐9619843;R‐HSA‐  379382;R‐HSA‐111925;R‐HSA‐374909;R‐HSA‐429594;R‐HSA‐388824 | 13 | 3.49% |
| 17 | R‐HSA‐9709957 | Sensory Perception | YES | YES | 14 | 1262 | 0.083174 | 1 | 0.999999 | 20 | 143 | 0.010073 | 9606 | Homo sapiens | SLC24A1;ABCA4;MSN;RBP4;OPN1SW;OPN1MW;RDH12;OPN1LW;RDH5; MYH9;LRAT;SLC17A8;SPTBN1;STRA6 | R‐HSA‐2466085;R‐HSA‐2465924;R‐HSA‐2453863;R‐HSA‐2404134;R‐HSA‐2404135;R‐HSA‐9662096;R‐ HSA‐2454081;R‐HSA‐9659380;R‐HSA‐9656891;R‐HSA‐2404137;R‐HSA‐2514891;R‐HSA‐9656893;R‐ HSA‐9663363;R‐HSA‐2453876;R‐HSA‐2464822;R‐HSA‐975608;R‐HSA‐2466749;R‐HSA‐2465917;R‐  HSA‐2453855;R‐HSA‐1467466 | 14 | 3.76% |
| 18 | R‐HSA‐5357801 | Programmed Cell Death | YES | YES | 12 | 237 | 0.01562 | 0.06462 | 0.129235 | 49 | 193 | 0.013595 | 9606 | Homo sapiens | BCL2L11;APC;CDKN2A;LMNA;MAPK1;AKT1;CTNNB1;RIPK1;TICAM1;TP53  ;GSDME | R‐HSA‐4331331;R‐HSA‐9647632;R‐HSA‐508163;R‐HSA‐5218891;R‐HSA‐508162;R‐HSA‐9710354;R‐ HSA‐9693978;R‐HSA‐139913;R‐HSA‐139918;R‐HSA‐139919;R‐HSA‐4331340;R‐HSA‐9688832;R‐HSA‐ 5218906;R‐HSA‐5675456;R‐HSA‐5218905;R‐HSA‐9686922;R‐HSA‐9688456;R‐HSA‐9686920;R‐HSA‐  9710263;R‐HSA‐2562564;R‐HSA‐264865;R‐HSA‐9793444;R‐HSA‐139952;R‐HSA‐140214;R‐HSA‐  5357927;R‐HSA‐9710254;R‐HSA‐6805981;R‐HSA‐5620975;R‐HSA‐9793451;R‐HSA‐9647660;R‐HSA‐  202947;R‐HSA‐9627089;R‐HSA‐9687638;R‐HSA‐198347;R‐HSA‐3371360;R‐HSA‐5357828;R‐HSA‐  5213466;R‐HSA‐5213464;R‐HSA‐5213462;R‐HSA‐202969;R‐HSA‐9687625;R‐HSA‐3465448;R‐HSA‐  3465429;R‐HSA‐141159;R‐HSA‐9710323;R‐HSA‐9645694;R‐HSA‐114284;R‐HSA‐9645692;R‐HSA‐  9710306 | 11 | 2.96% |
| 19 | R‐HSA‐1474165 | Reproduction | YES | YES | 6 | 122 | 0.008041 | 0.17477 | 0.174765 | 9 | 24 | 0.001691 | 9606 | Homo sapiens | B4GALT1;GAA;LMNA;BRCA1;MLH1;BRCA2 | R‐HSA‐912429;R‐HSA‐912408;R‐HSA‐912458;R‐HSA‐1297338;R‐HSA‐912503;R‐HSA‐912470;R‐HSA‐  912496;R‐HSA‐912467;R‐HSA‐912450 | 6 | 1.61% |
| 20 | R‐HSA‐8953854 | Metabolism of RNA | YES | YES | 6 | 830 | 0.054702 | 1 | 0.999999 | 26 | 189 | 0.013314 | 9606 | Homo sapiens | RANBP2;CPSF6;FIP1L1;TPR;AKT1 | R‐HSA‐8849157;R‐HSA‐159046;R‐HSA‐450499;R‐HSA‐159050;R‐HSA‐111439;R‐HSA‐77587;R‐HSA‐ 191825;R‐HSA‐158481;R‐HSA‐77589;R‐HSA‐191830;R‐HSA‐77591;R‐HSA‐158484;R‐HSA‐77590;R‐ HSA‐77593;R‐HSA‐75097;R‐HSA‐77592;R‐HSA‐75096;R‐HSA‐77594;R‐HSA‐75098;R‐HSA‐6783483;R‐  HSA‐72231;R‐HSA‐158441;R‐HSA‐158447;R‐HSA‐72180;R‐HSA‐72185;R‐HSA‐450490 | 5 | 1.34% |
| 21 | R‐HSA‐1500931 | Cell‐Cell communication | YES | YES | 5 | 134 | 0.008831 | 0.39208 | 0.392078 | 4 | 60 | 0.004227 | 9606 | Homo sapiens | SFTPA2;PIK3CA;CTNNB1;PIK3R1;SPTBN1 | R‐HSA‐391155;R‐HSA‐451758;R‐HSA‐419002;R‐HSA‐451403 | 5 | 1.34% |
| 22 | R‐HSA‐397014 | Muscle contraction | YES | YES | 5 | 232 | 0.01529 | 0.83889 | 0.838888 | 11 | 53 | 0.003733 | 9606 | Homo sapiens | TPM4;TPM3;AKAP9;ABCC9;VCL | R‐HSA‐390597;R‐HSA‐445700;R‐HSA‐5678261;R‐HSA‐390598;R‐HSA‐390593;R‐HSA‐445699;R‐HSA‐  390595;R‐HSA‐9691566;R‐HSA‐445704;R‐HSA‐5577050;R‐HSA‐445705 | 5 | 1.34% |
| 23 | R‐HSA‐1852241 | Organelle biogenesis and  maintenance | YES | YES | 5 | 338 | 0.022276 | 0.97792 | 0.977915 | 9 | 86 | 0.006058 | 9606 | Homo sapiens | DCTN1;CNTRL;AKAP9;TRIP11;CEP43 | R‐HSA‐5626228;R‐HSA‐5617828;R‐HSA‐5626227;R‐HSA‐5626220;R‐HSA‐5626223;R‐HSA‐5626681;R‐  HSA‐5617816;R‐HSA‐5626699;R‐HSA‐5638009 | 5 | 1.34% |
| 24 | R‐HSA‐8963743 | Digestion and absorption | YES | YES | 4 | 71 | 0.004679 | 0.17555 | 0.175553 | 8 | 30 | 0.002113 | 9606 | Homo sapiens | SI;SLC2A2;SLC5A1;LCT | R‐HSA‐5659861;R‐HSA‐8932955;R‐HSA‐189062;R‐HSA‐191108;R‐HSA‐189242;R‐HSA‐191101;R‐HSA‐  189053;R‐HSA‐189069 | 4 | 1.08% |
| 25 | R‐HSA‐400253 | Circadian Clock | YES | YES | 3 | 105 | 0.00692 | 0.62469 | 0.624694 | 3 | 59 | 0.004156 | 9606 | Homo sapiens | PPP1CB;AVP | R‐HSA‐400382;R‐HSA‐879782;R‐HSA‐5663165 | 2 | 0.54% |
| 26 | R‐HSA‐9612973 | Autophagy | YES | YES | 3 | 166 | 0.01094 | 0.88387 | 0.883865 | 37 | 108 | 0.007608 | 9606 | Homo sapiens | SQSTM1;ATG7;CFTR | R‐HSA‐9020616;R‐HSA‐9646354;R‐HSA‐9646679;R‐HSA‐9641111;R‐HSA‐9664855;R‐HSA‐5682896;R‐ HSA‐9641109;R‐HSA‐9631065;R‐HSA‐9624158;R‐HSA‐5682011;R‐HSA‐9646685;R‐HSA‐9631068;R‐ HSA‐5205663;R‐HSA‐9646347;R‐HSA‐5681999;R‐HSA‐5682893;R‐HSA‐9626060;R‐HSA‐5205649;R‐ HSA‐5683593;R‐HSA‐9646348;R‐HSA‐9664881;R‐HSA‐9664880;R‐HSA‐9646390;R‐HSA‐5205673;R‐ HSA‐5683583;R‐HSA‐5681980;R‐HSA‐9622840;R‐HSA‐5681981;R‐HSA‐9664892;R‐HSA‐9641127;R‐ HSA‐9625188;R‐HSA‐9620197;R‐HSA‐9615721;R‐HSA‐9646383;R‐HSA‐9622831;R‐HSA‐9625196;R‐ HSA‐9625197 | 3 | 0.81% |
| 27 | R‐HSA‐9609507 | Protein localization | YES | YES | 3 | 170 | 0.011204 | 0.89329 | 0.893286 | 7 | 53 | 0.003733 | 9606 | Homo sapiens | IDH1;ABCD1 | R‐HSA‐1268022;R‐HSA‐9033233;R‐HSA‐9603784;R‐HSA‐9033235;R‐HSA‐9603775;R‐HSA‐9033236;R‐  HSA‐9603804 | 2 | 0.54% |
| 28 | R‐HSA‐1474244 | Extracellular matrix organization | YES | YES | 2 | 328 | 0.021617 | 0.99957 | 0.999565 | 16 | 319 | 0.022471 | 9606 | Homo sapiens | KDR;FN1 | R‐HSA‐202723;R‐HSA‐2327746;R‐HSA‐3788061;R‐HSA‐2537665;R‐HSA‐4088281;R‐HSA‐2545196;R‐  HSA‐2731141;R‐HSA‐3785684;R‐HSA‐216050;R‐HSA‐1566981;R‐HSA‐2396337;R‐HSA‐2327733;R‐ HSA‐2533950;R‐HSA‐2681681;R‐HSA‐349593;R‐HSA‐2533944 | 2 | 0.54% |
| 29 | R‐HSA‐69306 | DNA Replication | YES | YES | 1 | 168 | 0.011072 | 0.99436 | 0.994357 | 6 | 58 | 0.004086 | 9606 | Homo sapiens | GAA | R‐HSA‐68960;R‐HSA‐68914;R‐HSA‐68916;R‐HSA‐69140;R‐HSA‐69142;R‐HSA‐69144 | 1 | 0.27% |
| 30 | R‐HSA‐4839726 | Chromatin organization | YES | YES | 1 | 254 | 0.01674 | 0.99961 | 0.99961 | 4 | 85 | 0.005988 | 9606 | Homo sapiens | KDM7A | R‐HSA‐4754187;R‐HSA‐4724284;R‐HSA‐5661121;R‐HSA‐5661115 | 1 | 0.27% |
