# Supplementary material for: Pathway-based, reaction-specific annotation of disease variants for elucidation of molecular phenotypes
Source: Database (Oxford). 2024 May 7;2024:baae031. doi: 10.1093/database/baae031 (PMC11184451; doi:10.1093/database/baae031)
Supplement: baae031_Supp [file baae031_supp.zip › suppl_data/Database_Orlic_Milacic_Reactome_of_Disease_Variants_SupplementaryTable3.docx]

|  | A | B | C |
| --- | --- | --- | --- |
| 1 | UniqueDiseases_DOID | DiseaseTerm_DOID | VariantEWASs |
| 2 | DOID:0001816 | angiosarcoma | 1 |
| 3 | DOID:0050129 | secretory diarrhea | 4 |
| 4 | DOID:0050156 | idiopathic pulmonary fibrosis | 2 |
| 5 | DOID:0050328 | congenital hypothyroidism | 6 |
| 6 | DOID:0050336 | hypophosphatemia | 2 |
| 7 | DOID:0050424 | familial adenomatous polyposis | 30 |
| 8 | DOID:0050445 | X‐linked dominant hypophosphatemic rickets | 5 |
| 9 | DOID:0050450 | Gitelman syndrome | 6 |
| 10 | DOID:0050458 | juvenile myelomonocytic leukemia | 3 |
| 11 | DOID:0050459 | hyperphosphatemia | 4 |
| 12 | DOID:0050469 | Costello syndrome | 6 |
| 13 | DOID:0050524 | maturity‐onset diabetes of the young | 6 |
| 14 | DOID:0111162 | epidermal nevus | 1 |
| 15 | DOID:0050534 | congenital stationary night blindness | 1 |
| 16 | DOID:0050544 | hypermethioninemia | 5 |
| 17 | DOID:0050546 | congenital adrenal insufficiency | 3 |
| 18 | DOID:0050557 | congenital muscular dystrophy | 4 |
| 19 | DOID:0050564 | autosomal dominant nonsyndromic deafness | 1 |
| 20 | DOID:0050570 | congenital disorder of glycosylation type I | 74 |
| 21 | DOID:0050571 | congenital disorder of glycosylation type II | 19 |
| 22 | DOID:0050572 | cone‐rod dystrophy | 1 |
| 23 | DOID:0050579 | glycogen storage disease XV | 1 |
| 24 | DOID:0050581 | brachydactyly | 4 |
| 25 | DOID:0050588 | muscular dystrophy‐dystroglycanopathy type B1 | 16 |
| 26 | DOID:0050590 | severe congenital neutropenia | 2 |
| 27 | DOID:0050593 | primary congenital glaucoma | 5 |
| 28 | DOID:0050605 | acrodermatitis enteropathica | 8 |
| 29 | DOID:0050632 | oculocutaneous albinism | 2 |
| 30 | DOID:0050641 | Rh deficiency syndrome | 6 |
| 31 | DOID:0050642 | hypochromic microcytic anemia | 5 |
| 32 | DOID:0050645 | arterial tortuosity syndrome | 6 |
| 33 | DOID:0050646 | distal arthrogryposis | 2 |
| 34 | DOID:0050650 | familial atrial fibrillation | 1 |
| 35 | DOID:0050657 | Bannayan‐Riley‐Ruvalcaba syndrome | 1 |
| 36 | DOID:0050660 | Beare‐Stevenson cutis gyrata syndrome | 4 |
| 37 | DOID:0050679 | blue cone monochromacy | 4 |
| 38 | DOID:0050711 | aceruloplasminemia | 4 |
| 39 | DOID:0050715 | methylmalonic aciduria and homocystinuria type cblC | 5 |
| 40 | DOID:0050716 | methylmalonic aciduria and homocystinuria type cblD | 7 |
| 41 | DOID:0050718 | vitamin metabolic disorder | 21 |
| 42 | DOID:0050732 | methylmalonic aciduria and homocystinuria type cblE | 3 |
| 43 | DOID:0050733 | methylmalonic aciduria and homocystinuria type cblG | 6 |
| 44 | DOID:0050737 | autosomal recessive disease | 5 |

|  | A | B | C |
| --- | --- | --- | --- |
| 45 | DOID:0050744 | anaplastic large cell lymphoma | 34 |
| 46 | DOID:0050745 | diffuse large B‐cell lymphoma | 15 |
| 47 | DOID:0050746 | mantle cell lymphoma | 5 |
| 48 | DOID:0050749 | peripheral T‐cell lymphoma | 1 |
| 49 | DOID:0050771 | pheochromocytoma | 15 |
| 50 | DOID:0050773 | paraganglioma | 16 |
| 51 | DOID:0050775 | schneckenbecken dysplasia | 4 |
| 52 | DOID:0050866 | oral squamous cell carcinoma | 14 |
| 53 | DOID:0050873 | follicular lymphoma | 1 |
| 54 | DOID:0050888 | syndromic intellectual disability | 5 |
| 55 | DOID:0050891 | adrenal cortical adenoma | 1 |
| 56 | DOID:0050892 | adrenal gland pheochromocytoma | 1 |
| 57 | DOID:0050904 | salivary gland carcinoma | 3 |
| 58 | DOID:0050905 | inflammatory myofibroblastic tumor | 16 |
| 59 | DOID:0050908 | myelodysplastic syndrome | 16 |
| 60 | DOID:0050913 | large intestine adenocarcinoma | 13 |
| 61 | DOID:0050921 | pharynx squamous cell carcinoma | 17 |
| 62 | DOID:0050927 | duodenum adenoma | 1 |
| 63 | DOID:0050929 | mucosal melanoma | 5 |
| 64 | DOID:0050937 | retroperitoneal neuroblastoma | 2 |
| 65 | DOID:0050938 | breast lobular carcinoma | 1 |
| 66 | DOID:0060002 | C1 inhibitor deficiency | 43 |
| 67 | DOID:0060037 | developmental disorder of mental health | 5 |
| 68 | DOID:0060041 | autism spectrum disorder | 1 |
| 69 | DOID:870 | neuropathy | 5 |
| 70 | DOID:0060058 | lymphoma | 10 |
| 71 | DOID:0060060 | non‐Hodgkin lymphoma | 6 |
| 72 | DOID:0060075 | estrogen‐receptor positive breast cancer | 3 |
| 73 | DOID:0060081 | triple‐receptor negative breast cancer | 8 |
| 74 | DOID:0050902 | medulloblastoma | 14 |
| 75 | DOID:0060106 | brain meningioma | 3 |
| 76 | DOID:0060108 | brain glioma | 47 |
| 77 | DOID:0060119 | pharynx cancer | 15 |
| 78 | DOID:0060158 | acquired metabolic disease | 5 |
| 79 | DOID:0060233 | cardiofaciocutaneous syndrome | 30 |
| 80 | DOID:0060350 | adenine phosphoribosyltransferase deficiency | 3 |
| 81 | DOID:0060643 | primary sclerosing cholangitis | 2 |
| 82 | DOID:0070004 | myeloid neoplasm | 3 |
| 83 | DOID:0080001 | bone disease | 4 |
| 84 | DOID:0080006 | bone development disease | 42 |
| 85 | DOID:0080027 | spondyloepimetaphyseal dysplasia | 10 |
| 86 | DOID:0080041 | hypochondroplasia | 7 |
| 87 | DOID:0080055 | achondrogenesis type IB | 4 |
| 88 | DOID:0080372 | epithelioid inflammatory myofibroblastic sarcoma | 4 |
| 89 | DOID:0080522 | thyroid gland anaplastic carcinoma | 13 |
| 90 | DOID:0080534 | myxofibrosarcoma | 1 |

|  | A | B | C |
| --- | --- | --- | --- |
| 91 | DOID:0080674 | luminal breast carcinoma B | 1 |
| 92 | DOID:0111147 | angioimmunoblastic T‐cell lymphoma | 1 |
| 93 | DOID:10003 | sensorineural hearing loss | 7 |
| 94 | DOID:10211 | cholelithiasis | 4 |
| 95 | DOID:10283 | prostate cancer | 31 |
| 96 | DOID:10286 | prostate carcinoma | 6 |
| 97 | DOID:1035 | aggressive NK‐cell leukemia | 1 |
| 98 | DOID:1040 | chronic lymphocytic leukemia | 2 |
| 99 | DOID:10534 | stomach cancer | 72 |
| 100 | DOID:10584 | retinitis pigmentosa | 2 |
| 101 | DOID:10588 | adrenoleukodystrophy | 6 |
| 102 | DOID:1060 | Hartnup disease | 5 |
| 103 | DOID:10603 | glucose intolerance | 7 |
| 104 | DOID:10604 | lactose intolerance | 2 |
| 105 | DOID:10609 | rickets | 9 |
| 106 | DOID:10629 | microphthalmia | 8 |
| 107 | DOID:10652 | Alzheimer's disease | 3 |
| 108 | DOID:1067 | open‐angle glaucoma | 1 |
| 109 | DOID:1070 | primary open angle glaucoma | 1 |
| 110 | DOID:10816 | duodenum adenocarcinoma | 1 |
| 111 | DOID:10991 | basal ganglia cerebrovascular disease | 6 |
| 112 | DOID:1107 | esophageal carcinoma | 11 |
| 113 | DOID:11105 | fundus albipunctatus | 5 |
| 114 | DOID:1115 | sarcoma | 2 |
| 115 | DOID:11193 | syndactyly | 2 |
| 116 | DOID:11661 | blue color blindness | 3 |
| 117 | DOID:11717 | neonatal diabetes | 7 |
| 118 | DOID:11984 | hypertrophic cardiomyopathy | 16 |
| 119 | DOID:12003 | trachea squamous cell carcinoma | 1 |
| 120 | DOID:1206 | Rett syndrome | 23 |
| 121 | DOID:12117 | pulmonary alveolar microlithiasis | 6 |
| 122 | DOID:12120 | pulmonary alveolar proteinosis | 3 |
| 123 | DOID:12134 | factor VIII deficiency | 78 |
| 124 | DOID:12176 | goiter | 5 |
| 125 | DOID:12190 | descending colon cancer | 2 |
| 126 | DOID:12192 | sigmoid colon cancer | 3 |
| 127 | DOID:12259 | hemophilia B | 30 |
| 128 | DOID:12308 | Dubin‐Johnson syndrome | 4 |
| 129 | DOID:12388 | neurohypophyseal diabetes insipidus | 7 |
| 130 | DOID:12678 | hypercalcemia | 6 |
| 131 | DOID:12716 | newborn respiratory distress syndrome | 15 |
| 132 | DOID:12798 | mucopolysaccharidosis | 14 |
| 133 | DOID:12799 | mucopolysaccharidosis II | 4 |
| 134 | DOID:12800 | mucopolysaccharidosis VI | 6 |
| 135 | DOID:12801 | mucopolysaccharidosis III | 8 |
| 136 | DOID:12802 | mucopolysaccharidosis I | 3 |

|  | A | B | C |
| --- | --- | --- | --- |
| 137 | DOID:12803 | Sly syndrome | 5 |
| 138 | DOID:12804 | mucopolysaccharidosis IV | 3 |
| 139 | DOID:12849 | autistic disorder | 1 |
| 140 | DOID:1289 | neurodegenerative disease | 6 |
| 141 | DOID:12930 | dilated cardiomyopathy | 2 |
| 142 | DOID:12960 | acrocephalosyndactylia | 10 |
| 143 | DOID:12971 | hereditary spherocytosis | 2 |
| 144 | DOID:1324 | lung cancer | 127 |
| 145 | DOID:13317 | hyperinsulinemic hypoglycemia | 4 |
| 146 | DOID:13359 | Ehlers‐Danlos syndrome | 13 |
| 147 | DOID:13382 | megaloblastic anemia | 5 |
| 148 | DOID:13481 | thanatophoric dysplasia | 15 |
| 149 | DOID:13636 | Fanconi anemia | 10 |
| 150 | DOID:1380 | endometrial cancer | 157 |
| 151 | DOID:1388 | Tangier disease | 6 |
| 152 | DOID:13909 | red‐green color blindness | 1 |
| 153 | DOID:13910 | red color blindness | 1 |
| 154 | DOID:14219 | renal tubular acidosis | 14 |
| 155 | DOID:14252 | dystrophies primarily involving the retinal pigment epithelium | 2 |
| 156 | DOID:14283 | primary hypertrophic osteoarthropathy | 5 |
| 157 | DOID:14291 | Noonan syndrome with multiple lentigines | 4 |
| 158 | DOID:14365 | systemic primary carnitine deficiency disease | 6 |
| 159 | DOID:14448 | name:46,XY sex reversal | 1 |
| 160 | DOID:14705 | Pfeiffer syndrome | 5 |
| 161 | DOID:14735 | hereditary angioedema | 8 |
| 162 | DOID:14749 | methylmalonic acidemia | 19 |
| 163 | DOID:14764 | Larsen syndrome | 1 |
| 164 | DOID:14791 | Leber congenital amaurosis | 5 |
| 165 | DOID:1485 | cystic fibrosis | 8 |
| 166 | DOID:150 | disease of mental health | 1 |
| 167 | DOID:1519 | cecum carcinoma | 7 |
| 168 | DOID:1520 | colon carcinoma | 1 |
| 169 | DOID:1540 | parathyroid carcinoma | 1 |
| 170 | DOID:1542 | head and neck carcinoma | 1 |
| 171 | DOID:1612 | breast cancer | 270 |
| 172 | DOID:1618 | breast fibroadenoma | 1 |
| 173 | DOID:162 | cancer | 3491 |
| 174 | DOID:1631 | benign breast phyllodes tumor | 12 |
| 175 | DOID:1682 | congenital heart disease | 1 |
| 176 | DOID:1686 | glaucoma | 2 |
| 177 | DOID:0060655 | autosomal recessive congenital ichthyosis | 9 |
| 178 | DOID:1701 | steroid inherited metabolic disorder | 7 |
| 179 | DOID:1752 | ocular melanoma | 1 |
| 180 | DOID:1781 | thyroid gland cancer | 36 |
| 181 | DOID:1785 | pituitary cancer | 4 |
| 182 | DOID:1788 | peritoneal mesothelioma | 1 |

|  | A | B | C |
| --- | --- | --- | --- |
| 183 | DOID:1793 | pancreatic cancer | 50 |
| 184 | DOID:1799 | islet cell tumor | 17 |
| 185 | DOID:1826 | epilepsy | 2 |
| 186 | DOID:1852 | intrahepatic cholestasis | 10 |
| 187 | DOID:1907 | malignant fibrous histiocytoma | 4 |
| 188 | DOID:1909 | melanoma | 199 |
| 189 | DOID:1919 | Lesch‐Nyhan syndrome | 3 |
| 190 | DOID:1967 | leiomyosarcoma | 19 |
| 191 | DOID:1996 | rectum adenocarcinoma | 7 |
| 192 | DOID:201 | connective tissue cancer | 3 |
| 193 | DOID:206 | hereditary multiple exostoses | 11 |
| 194 | DOID:2101 | vulva squamous cell carcinoma | 4 |
| 195 | DOID:2154 | nephroblastoma | 2 |
| 196 | DOID:218 | ascending colon cancer | 1 |
| 197 | DOID:2187 | amelogenesis imperfecta | 3 |
| 198 | DOID:2226 | myeloproliferative neoplasm | 16 |
| 199 | DOID:225 | syndrome | 1 |
| 200 | DOID:2256 | osteochondrodysplasia | 13 |
| 201 | DOID:2339 | Crouzon syndrome | 10 |
| 202 | DOID:234 | colon adenocarcinoma | 55 |
| 203 | DOID:2340 | craniosynostosis | 7 |
| 204 | DOID:2352 | hemochromatosis | 6 |
| 205 | DOID:2355 | anemia | 4 |
| 206 | DOID:2394 | ovarian cancer | 62 |
| 207 | DOID:2433 | epidermal appendage tumor | 7 |
| 208 | DOID:2452 | thrombophilia | 1 |
| 209 | DOID:2476 | hereditary spastic paraplegia | 15 |
| 210 | DOID:2477 | motor peripheral neuropathy | 1 |
| 211 | DOID:0050709 | early infantile epileptic encephalopathy | 8 |
| 212 | DOID:2513 | basal cell carcinoma | 8 |
| 213 | DOID:2526 | prostate adenocarcinoma | 34 |
| 214 | DOID:2531 | hematologic cancer | 43 |
| 215 | DOID:2565 | macular corneal dystrophy | 8 |
| 216 | DOID:2596 | larynx cancer | 23 |
| 217 | DOID:263 | kidney cancer | 14 |
| 218 | DOID:2679 | dysembryoplastic neuroepithelial tumor | 1 |
| 219 | DOID:2738 | pseudoxanthoma elasticum | 1 |
| 220 | DOID:2741 | bilirubin metabolic disorder | 3 |
| 221 | DOID:2747 | glycogen storage disease | 8 |
| 222 | DOID:2749 | glycogen storage disease I | 5 |
| 223 | DOID:2750 | glycogen storage disease IV | 2 |
| 224 | DOID:2752 | glycogen storage disease II | 2 |
| 225 | DOID:285 | hairy cell leukemia | 1 |
| 226 | DOID:2861 | congenital nonspherocytic hemolytic anemia | 3 |
| 227 | DOID:2871 | endometrial carcinoma | 10 |
| 228 | DOID:2876 | laryngeal squamous cell carcinoma | 1 |

|  | A | B | C |
| --- | --- | --- | --- |
| 229 | DOID:2891 | thyroid adenoma | 1 |
| 230 | DOID:2978 | carbohydrate metabolic disorder | 7 |
| 231 | DOID:2994 | germ cell cancer | 3 |
| 232 | DOID:2998 | testicular cancer | 7 |
| 233 | DOID:3001 | female reproductive endometrioid cancer | 24 |
| 234 | DOID:3007 | breast ductal carcinoma | 21 |
| 235 | DOID:3012 | Li‐Fraumeni syndrome | 2 |
| 236 | DOID:3039 | cecum adenocarcinoma | 3 |
| 237 | DOID:305 | carcinoma | 5 |
| 238 | DOID:3068 | glioblastoma | 64 |
| 239 | DOID:3069 | malignant astrocytoma | 180 |
| 240 | DOID:3071 | gliosarcoma | 6 |
| 241 | DOID:3082 | interstitial lung disease | 8 |
| 242 | DOID:3113 | papillary carcinoma | 2 |
| 243 | DOID:3146 | lipid metabolism disorder | 11 |
| 244 | DOID:3149 | keratoacanthoma | 1 |
| 245 | DOID:3151 | skin squamous cell carcinoma | 47 |
| 246 | DOID:3162 | malignant spindle cell melanoma | 2 |
| 247 | DOID:3165 | skin benign neoplasm | 2 |
| 248 | DOID:3187 | brain oligodendroglioma | 4 |
| 249 | DOID:3211 | lysosomal storage disease | 5 |
| 250 | DOID:3247 | rhabdomyosarcoma | 15 |
| 251 | DOID:3264 | subacute leukemia | 18 |
| 252 | DOID:3275 | thymoma | 1 |
| 253 | DOID:3277 | thymus cancer | 5 |
| 254 | DOID:3284 | thymic carcinoma | 1 |
| 255 | DOID:3302 | chordoma | 1 |
| 256 | DOID:3307 | teratoma | 3 |
| 257 | DOID:3314 | angiomyolipoma | 2 |
| 258 | DOID:3321 | GM2 gangliosidosis | 4 |
| 259 | DOID:3322 | GM1 gangliosidosis | 1 |
| 260 | DOID:3347 | osteosarcoma | 18 |
| 261 | DOID:3355 | fibrosarcoma | 2 |
| 262 | DOID:3369 | Ewing sarcoma | 2 |
| 263 | DOID:3371 | chondrosarcoma | 5 |
| 264 | DOID:3382 | liposarcoma | 2 |
| 265 | DOID:3405 | histiocytosis | 6 |
| 266 | DOID:3429 | inclusion body myositis | 2 |
| 267 | DOID:3449 | penis carcinoma | 7 |
| 268 | DOID:3459 | breast carcinoma | 28 |
| 269 | DOID:6457 | Cowden syndrome | 2 |
| 270 | DOID:3490 | Noonan syndrome | 55 |
| 271 | DOID:3498 | pancreatic ductal adenocarcinoma | 87 |
| 272 | DOID:350 | mastocytosis | 2 |
| 273 | DOID:3500 | gallbladder adenocarcinoma | 17 |
| 274 | DOID:3571 | liver cancer | 6 |

|  | A | B | C |
| --- | --- | --- | --- |
| 275 | DOID:3602 | toxic encephalopathy | 5 |
| 276 | DOID:3608 | appendix adenocarcinoma | 2 |
| 277 | DOID:3620 | central nervous system cancer | 3 |
| 278 | DOID:3635 | congenital myasthenic syndrome | 4 |
| 279 | DOID:3659 | sialuria | 3 |
| 280 | DOID:3664 | mast cell neoplasm | 16 |
| 281 | DOID:3702 | cervical adenocarcinoma | 6 |
| 282 | DOID:3711 | bladder adenocarcinoma | 3 |
| 283 | DOID:3717 | gastric adenocarcinoma | 13 |
| 284 | DOID:3742 | bladder squamous cell carcinoma | 3 |
| 285 | DOID:3744 | cervical squamous cell carcinoma | 23 |
| 286 | DOID:3748 | esophagus squamous cell carcinoma | 53 |
| 287 | DOID:3765 | pseudohermaphroditism | 4 |
| 288 | DOID:3803 | Crigler‐Najjar syndrome | 7 |
| 289 | DOID:3829 | pituitary adenoma | 3 |
| 290 | DOID:3907 | lung squamous cell carcinoma | 83 |
| 291 | DOID:3908 | lung non‐small cell carcinoma | 106 |
| 292 | DOID:3910 | lung adenocarcinoma | 156 |
| 293 | DOID:3948 | adrenocortical carcinoma | 5 |
| 294 | DOID:3953 | adrenal gland cancer | 3 |
| 295 | DOID:3962 | thyroid gland follicular carcinoma | 4 |
| 296 | DOID:3963 | thyroid gland carcinoma | 30 |
| 297 | DOID:3965 | Merkel cell carcinoma | 17 |
| 298 | DOID:3969 | thyroid gland papillary carcinoma | 5 |
| 299 | DOID:3996 | urinary system cancer | 17 |
| 300 | DOID:4000 | ovary transitional cell carcinoma | 1 |
| 301 | DOID:4001 | ovarian carcinoma | 10 |
| 302 | DOID:4007 | bladder carcinoma | 124 |
| 303 | DOID:4118 | colon neuroendocrine neoplasm | 1 |
| 304 | DOID:417 | autoimmune disease | 1 |
| 305 | DOID:420 | hypertrichosis | 6 |
| 306 | DOID:4202 | brain stem glioma | 1 |
| 307 | DOID:423 | myopathy | 3 |
| 308 | DOID:4231 | histiocytoma | 3 |
| 309 | DOID:4362 | cervical cancer | 24 |
| 310 | DOID:4415 | fibrous histiocytoma | 3 |
| 311 | DOID:4448 | macular degeneration | 4 |
| 312 | DOID:445 | Bartter disease | 6 |
| 313 | DOID:4450 | renal cell carcinoma | 4 |
| 314 | DOID:4465 | papillary renal cell carcinoma | 2 |
| 315 | DOID:4467 | clear cell renal cell carcinoma | 22 |
| 316 | DOID:447 | renal tubular transport disease | 20 |
| 317 | DOID:4471 | chromophobe renal cell carcinoma | 2 |
| 318 | DOID:4480 | achondroplasia | 2 |
| 319 | DOID:4556 | lung large cell carcinoma | 1 |
| 320 | DOID:4607 | biliary tract cancer | 9 |

|  | A | B | C |
| --- | --- | --- | --- |
| 321 | DOID:4621 | holoprosencephaly | 39 |
| 322 | DOID:4715 | gastric neuroendocrine neoplasm | 1 |
| 323 | DOID:4810 | cerebrotendinous xanthomatosis | 4 |
| 324 | DOID:4840 | sebaceous carcinoma | 6 |
| 325 | DOID:4851 | pilocytic astrocytoma | 11 |
| 326 | DOID:4866 | salivary gland adenoid cystic carcinoma | 2 |
| 327 | DOID:4896 | bile duct adenocarcinoma | 4 |
| 328 | DOID:4897 | bile duct carcinoma | 56 |
| 329 | DOID:4907 | small intestine carcinoma | 21 |
| 330 | DOID:4914 | esophagus adenocarcinoma | 26 |
| 331 | DOID:4919 | renal pelvis carcinoma | 1 |
| 332 | DOID:4921 | eccrine sweat gland cancer | 1 |
| 333 | DOID:4926 | bronchiolo‐alveolar adenocarcinoma | 1 |
| 334 | DOID:4931 | nasal cavity carcinoma | 3 |
| 335 | DOID:4944 | gastroesophageal junction adenocarcinoma | 2 |
| 336 | DOID:4947 | cholangiocarcinoma | 7 |
| 337 | DOID:4948 | gallbladder carcinoma | 3 |
| 338 | DOID:4971 | myelofibrosis | 1 |
| 339 | DOID:4972 | myelodysplastic/myeloproliferative neoplasm | 29 |
| 340 | DOID:4989 | pancreatitis | 1 |
| 341 | DOID:5032 | pineal gland cancer | 1 |
| 342 | DOID:5176 | renal Wilms' tumor | 7 |
| 343 | DOID:5212 | congenital disorder of glycosylation | 5 |
| 344 | DOID:5241 | hemangioblastoma | 1 |
| 345 | DOID:5409 | lung small cell carcinoma | 114 |
| 346 | DOID:5410 | pulmonary neuroendocrine tumor | 1 |
| 347 | DOID:5485 | synovial sarcoma | 1 |
| 348 | DOID:5511 | dysgerminoma of ovary | 1 |
| 349 | DOID:5517 | stomach carcinoma | 6 |
| 350 | DOID:5520 | head and neck squamous cell carcinoma | 61 |
| 351 | DOID:5557 | testicular germ cell cancer | 1 |
| 352 | DOID:5585 | Ferguson‐Smith tumor | 9 |
| 353 | DOID:5598 | fallopian tube serous adenocarcinoma | 1 |
| 354 | DOID:9952 | acute lymphoblastic leukemia | 49 |
| 355 | DOID:5621 | histiocytic and dendritic cell cancer | 2 |
| 356 | DOID:5636 | cervical adenosquamous carcinoma | 1 |
| 357 | DOID:5667 | sweat gland carcinoma | 1 |
| 358 | DOID:5672 | large intestine cancer | 205 |
| 359 | DOID:5683 | hereditary breast ovarian cancer syndrome | 6 |
| 360 | DOID:5742 | pancreatic acinar cell adenocarcinoma | 4 |
| 361 | DOID:5744 | ovary serous adenocarcinoma | 45 |
| 362 | DOID:5777 | rectum neuroendocrine neoplasm | 1 |
| 363 | DOID:5813 | purine nucleoside phosphorylase deficiency | 3 |
| 364 | DOID:5828 | endometrioid ovary carcinoma | 2 |
| 365 | DOID:583 | hemolytic anemia | 5 |
| 366 | DOID:5842 | testis seminoma | 16 |

|  | A | B | C |
| --- | --- | --- | --- |
| 367 | DOID:585 | nephrolithiasis | 2 |
| 368 | DOID:5940 | malignant peripheral nerve sheath tumor | 1 |
| 369 | DOID:612 | primary immunodeficiency disease | 16 |
| 370 | DOID:6212 | ovarian endometrial cancer | 14 |
| 371 | DOID:627 | severe combined immunodeficiency | 1 |
| 372 | DOID:630 | genetic disease | 7 |
| 373 | DOID:6477 | invasive bladder transitional cell carcinoma | 20 |
| 374 | DOID:6498 | seborrheic keratosis | 5 |
| 375 | DOID:655 | inherited metabolic disorder | 30 |
| 376 | DOID:6740 | cervix small cell carcinoma | 1 |
| 377 | DOID:684 | hepatocellular carcinoma | 60 |
| 378 | DOID:686 | liver carcinoma | 8 |
| 379 | DOID:687 | hepatoblastoma | 1 |
| 380 | DOID:7045 | basaloid lung carcinoma | 1 |
| 381 | DOID:7141 | prostate small cell carcinoma | 4 |
| 382 | DOID:5603 | T‐cell acute lymphoblastic leukemia | 51 |
| 383 | DOID:734 | urethra cancer | 3 |
| 384 | DOID:7474 | malignant pleural mesothelioma | 3 |
| 385 | DOID:7497 | brain ependymoma | 2 |
| 386 | DOID:768 | retinoblastoma | 50 |
| 387 | DOID:769 | neuroblastoma | 38 |
| 388 | DOID:8161 | thyroid gland Hurthle cell carcinoma | 1 |
| 389 | DOID:8162 | thyroid Hurthle cell adenoma | 1 |
| 390 | DOID:83 | cataract | 2 |
| 391 | DOID:8541 | Sezary's disease | 2 |
| 392 | DOID:8552 | chronic myeloid leukemia | 26 |
| 393 | DOID:8577 | ulcerative colitis | 1 |
| 394 | DOID:8584 | Burkitt lymphoma | 1 |
| 395 | DOID:8602 | gum cancer | 2 |
| 396 | DOID:8618 | oral cavity cancer | 8 |
| 397 | DOID:8649 | tongue cancer | 1 |
| 398 | DOID:8691 | mycosis fungoides | 1 |
| 399 | DOID:8692 | myeloid leukemia | 4 |
| 400 | DOID:8712 | neurofibromatosis | 6 |
| 401 | DOID:8850 | salivary gland cancer | 27 |
| 402 | DOID:8923 | skin melanoma | 47 |
| 403 | DOID:9036 | parotid gland cancer | 1 |
| 404 | DOID:9119 | acute myeloid leukemia | 178 |
| 405 | DOID:9206 | Barrett's esophagus | 7 |
| 406 | DOID:9252 | amino acid metabolic disorder | 12 |
| 407 | DOID:9253 | gastrointestinal stromal tumor | 94 |
| 408 | DOID:9256 | colorectal cancer | 220 |
| 409 | DOID:9261 | nasopharynx carcinoma | 17 |
| 410 | DOID:9263 | homocystinuria | 2 |
| 411 | DOID:9266 | cystinuria | 10 |
| 412 | DOID:9281 | phenylketonuria | 1 |

|  | A | B | C |
| --- | --- | --- | --- |
| 413 | DOID:936 | brain disease | 4 |
| 414 | DOID:9538 | multiple myeloma | 16 |
| 415 | DOID:9553 | adrenal gland disease | 19 |
| 416 | DOID:963 | episodic ataxia | 2 |
| 417 | DOID:9655 | oral mucosa leukoplakia | 3 |
| 418 | DOID:9868 | intestinal disaccharidase deficiency | 3 |
| 419 | DOID:9869 | hereditary fructose intolerance syndrome | 2 |
| 420 | DOID:9870 | galactosemia | 9 |
| 421 | DOID:9970 | obesity | 3 |
| 422 | DOID:999 | hypereosinophilic syndrome | 14 |
